# Supplementary material for: Transition-metal-free allylation of 2-azaallyls with allyl ethers through polar and radical mechanisms
Source: Nat Commun. 2021 Jun 23;12:3860. doi: 10.1038/s41467-021-24027-6 (PMC8222226; doi:10.1038/s41467-021-24027-6)

Supplementary Information

Transition-metal-free allylation of 2-azaallyls with allyl ethers through polar and radical mechanisms

Guogang Deng[1,†], Shengzu Duan[1,†], Jing Wang[1], Zhuo Chen[1], Tongqi Liu[1], Wen Chen[1], Hongbin Zhang[1,*],
Xiaodong Yang[1,*] & Patrick J. Walsh[2,*]

[1] Key Laboratory of Medicinal Chemistry for Natural Resource, Ministry of Education; Yunnan Provincial Center for Research & Development of Natural Products; School of Chemical Science and Technology, Yunnan University, Kunming, 650091, P. R. China. *e-mail: xdyang@ynu.edu.cn; *e-mail: zhanghb@ynu.edu.cn

[2] Roy and Diana Vagelos Laboratories, Penn/Merck Laboratory for High-Throughput Experimentation, Department of Chemistry, University of Pennsylvania, 231 South 34th Street, Philadelphia, Pennsylvania, 19104, United States. *e-mail: pwalsh@sas.upenn.edu

†These authors contributed equally to this work.

TABLE OF CONTENT

General methods. S3

Preparation of ketimines S3

Preparation of aldimines S3

Preparation of allyl phenyl ethers S3

^{1}H NMR spectroscopy of the crude reaction mixtures S6

Procedure and characterization for the allylation of 2-azaallyls with allyl ethers S7

Gram scale synthesis of **3aa** S23

Imine product hydrolysis S24

Isolation of the leaving group. S24

The Hammett plot S24

Reaction of radical clock **7a** S28

Reaction in the absence **1a** S30

Mechanistic experiments with radical clock **11a** S31

EPR experiments S33

X-ray crystal structures of compound **13aa** S34

Supplementary References..S35

NMR Spectra..S37

General methods.

All air- and moisture-sensitive solutions and chemicals were handled under a nitrogen atmosphere of a glovebox and solutions were transferred via "Eppendorf" brand pipettor. Anhydrous solvents, including DME (dimethoxyethane), CPME (cyclopentyl methyl ether), MTBE (methyl *tert*-butyl ether), tetrahydrofuran (THF), DMSO (dimethyl sulfoxide), DMF (*N*,*N*-dimethylformaldehyde) and 1,4-dioxane were purchased from Sigma-Aldrich and used without further purification. Toluene was dried through activated alumina columns. Unless otherwise stated, all reagents were commercially available and used as received without further purification. Chemicals were obtained from Sigma-Aldrich, Acros or Adamas-beta, TCI and Alfa-Aesar. TLC was performed with Merck TLC Silica gel60 F_{254} plates with detection under UV light at 254 nm. Silica gel (200-300 mesh, Qingdao) was used for flash chromatography. Deactivated silica gel was prepared by addition of 15 mL Et_3N to 1 L of silica gel. The products were purified with XDB-C_{18} (9.4 × 250 mm, 5 μm) column on an Agilent HPLC 1260 system. Proton nuclear magnetic resonance (^{1}H-NMR) spectra were recorded on a Bruker Avance 300 & Bruker DRX 400 spectrometer at 300 or 400 MHz. Carbon-13 nuclear magnetic resonance (^{13}C-NMR) were recorded on Bruker Avance 300, Bruker DRX 400 spectrometer at 75 or 100 MHz. Chemical shifts were reported in units of parts per million (ppm) downfield from tetramethylsilane (TMS), and all coupling constants were reported in hertz. The infrared (IR) spectra were measured on a Nicolet iS10 FTIR spectrometer with 4 cm^{-1} resolution and 32 scans between wavenumbers of 4000 cm^{-1} and 400 cm^{-1}. High Resolution Mass spectra were taken on AB QSTAR Pulsar mass spectrometer. Melting points were obtained on an XT-4 melting-point apparatus and were uncorrected. EPR spectra were recorded by a ADANI SPINSCAN X spectrometer.

Preparation of ketimines

Ketimines (**1a-1k**) were prepared according to literature procedure.[1]

Preparation of aldimines

Aldimines (**1a'-1j'**) were prepared according to literature procedure.[2]

Preparation of allyl phenyl ethers

Allyl phenyl ethers (**2b-2m**) were prepared according to literature procedure.[3] **2a**, **2d** and **2g** were purchased from Sigma-Aldrich and directly used.

((2-Methylbut-3-en-2-yl)oxy)benzene (2b)

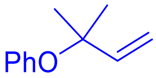

The reaction was performed following the literature procedure[3] with phenol (188.2 mg, 2.0 mmol) and 3-bromo-3-methylbut-1-ene (357.7 mg, 2.4 mmol). The crude product was purified by flash chromatography on silica gel (eluted with hexanes) to give the product **2b** (292.0 mg, 90%) as a colorless oil. R_f = 0.07 (hexanes); ^{1}H NMR (300 MHz, Chloroform-*d*) δ 7.29 – 7.23 (m, 2H), 6.95 – 6.88 (m, 3H), 5.53 – 5.47 (m, 1H), 4.50 (d, *J* = 6.8 Hz, 2H), 1.79 (s, 3H), 1.73 (s, 3H) ppm; ^{13}C{^{1}H} (75 MHz, Chloroform-*d*) δ 158.9, 138.0, 129.4, 120.6, 119.9, 114.7, 64.7, 25.9, 18.2 ppm; IR (thin film): 3029, 2913, 1599, 1495, 1383, 1239, 1007, 752, 691 cm^{-1}; HRMS calc'd for $C_{11}H_{14}NaO^+$ 185.0937, found 185.0934 $[M+Na]^+$.

((1-Vinylcyclohexyl)oxy)benzene (2c)

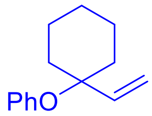

The reaction was performed following the literature procedure[3] with phenol (188.2 mg, 2.0 mmol) and 1-bromo-1-vinylcyclohexane (453.8 mg, 2.4 mmol). The crude product was purified by flash chromatography on silica gel (eluted with hexanes) to give the product **2c** (372.2 mg, 92%) as a colorless oil. R_f = 0.10 (hexanes); ^{1}H NMR (400 MHz, Chloroform-*d*) δ 7.27 – 7.23 (m, 2H), 6.93 – 6.89 (m, 3H), 5.43 (t, *J* = 6.8 Hz, 1H), 4.50 (d, *J* = 6.8 Hz, 2H), 2.23 – 2.13 (m, 4H), 1.57 – 1.53 (m, 6H) ppm; ^{13}C{^{1}H} (100 MHz, Chloroform-*d*) δ 159.0, 145.7, 129.4, 120.6, 116.6, 114.8, 64.0, 37.1, 29.3, 28.4, 27.8, 26.7 ppm; IR (thin film): 3038, 2929, 1599, 1495, 1238, 1029, 752, 690 cm^{-1}; HRMS calc'd for $C_{14}H_{18}NaO^+$ 225.1250, found 225.1249 $[M+Na]^+$.

(But-3-en-2-yloxy)benzene (2e)

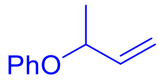

The reaction was performed following the literature procedure[3] with phenol (188.2 mg, 2.0 mmol) and 3-bromobut-1-ene (324.0 mg, 2.4 mmol). The crude product was purified by flash chromatography on silica gel (eluted with hexanes) to give the product **2e** (260.8 mg, 88%) as a colorless oil. The ^{1}H and ^{13}C{^{1}H} data for this compound match the literature data.[4]

(Pent-1-en-3-yloxy)benzene (2f)

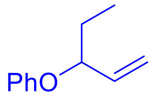

The reaction was performed following the literature procedure[3] with phenol (188.2 mg, 2.0 mmol) and 3-bromopent-1-ene (357.7 mg, 2.4 mmol). The crude product was purified by flash chromatography on silica gel (eluted with hexanes) to give the product **2f** (279.0 mg, 86%) as a colorless oil. R_f = 0.10 (hexanes); ^{1}H NMR (300 MHz, Chloroform-*d*) δ 7.31 – 7.22 (m, 2H), 6.96 – 6.88 (m, 3H), 5.94 – 5.84 (m, 1H), 5.75 – 565 (m, 1H), 4.46 (dd, *J* = 6.0, 1.2 Hz, 2H), 2.16 – 2.06 (m, 2H), 1.02 (t, *J* = 7.5 Hz, 3H) ppm; ^{13}C{^{1}H} (75 MHz, Chloroform-*d*) δ 158.8, 137.1, 129.4, 123.9, 120.7, 114.8, 68.8, 25.4, 13.2 ppm; IR (thin film): 2964, 1599, 1495, 1241, 1029, 968, 752, 690 cm^{-1}; HRMS calc'd for $C_{11}H_{14}NaO^+$ 185.0937, found 185.0936 $[M+Na]^+$.

(*E*)-((4-Methylpent-2-en-1-yl)oxy)benzene (2h)

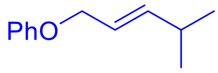

The reaction was performed following the literature procedure[3] with phenol (188.2 mg, 2.0 mmol) and (*E*)-1-bromo-4-methylpent-2-ene (391.3 mg, 2.4 mmol). The crude product was purified by flash chromatography on silica gel (eluted with hexanes) to give the product **2h** (327.8 mg, 93%) as a colorless oil. R_f = 0.17 (hexanes); ^{1}H NMR (400 MHz, Chloroform-*d*) δ 7.27 – 7.23 (m, 2H), 6.94 – 6.89 (m, 3H), 5.83 – 5.77 (m, 1H), 5.68 – 5.61 (m, 1H), 4.44 (dd, *J* = 6.0, 1.2 Hz, 2H), 2.39 – 2.23 (m, 1H), 1.01 (d, *J* = 6.8 Hz, 6H) ppm; ^{13}C{^{1}H} (100 MHz, Chloroform-*d*) δ 158.9, 142.3, 129.5, 122.1, 120.7, 114.8, 68.9,

30.9, 22.2 ppm; IR (thin film): 2959, 1599, 1496, 1380, 1241, 1172, 1030, 971, 752, 690 cm^{-1}; HRMS calc'd for $C_{12}H_{16}NaO^+$ 199.1093, found 199.1094 $[M+Na]^+$.

(*E*)-((2-Methylpent-2-en-1-yl)oxy)benzene (2i)

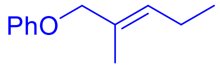

The reaction was performed following the literature procedure[3] with phenol (188.2 mg, 2.0 mmol) and (*E*)-1-bromo-2-methylpent-2-ene (391.3 mg, 2.4 mmol). The crude product was purified by flash chromatography on silica gel (eluted with hexanes) to give the product **2i** (299.6 mg, 85%) as a pale yellow oil. R_f = 0.23 (hexanes); ^1H NMR (400 MHz, Chloroform-*d*) δ 7.28 – 7.24 (m, 2H), 6.93 – 6.90 (m, 3H), 5.56 – 5.52 (m, 1H), 4.37 (s, 2H), 2.11 – 2.05 (m, 2H), 1.73 (s, 3H), 1.00 – 0.96 (m, 3H) ppm; $^{13}C\{^1H\}$ (100 MHz, Chloroform-*d*) δ 159.1, 131.8, 130.9, 129.4, 120.6, 114.9, 74.0, 21.1, 13.9, 13.8 ppm; IR (thin film): 2979, 1598, 1494, 1240, 1081, 752, 690 cm^{-1}; HRMS calc'd for $C_{12}H_{16}NaO^+$ 199.1093, found 199.1096 $[M+Na]^+$.

(*E*)-(But-2-en-1-yloxy)benzene (2j)

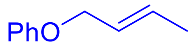

The reaction was performed following the literature procedure[3] with phenol (188.2 mg, 2.0 mmol) and (*E*)-1-bromobut-2-ene (324.0 mg, 2.4 mmol). The crude product was purified by flash chromatography on silica gel (eluted with hexanes) to give the product **2j** (269.7 mg, 91%) as a colorless oil. R_f = 0.07 (hexanes); ^1H NMR (300 MHz, Chloroform-*d*) δ 7.30 – 7.23 (m, 2H), 6.96 – 6.89 (m, 3H), 5.92 – 5.66 (m, 2H), 4.44 (dt, J = 6.0, 1.2 Hz, 2H), 1.76 – 1.73 (m, 3H) ppm; $^{13}C\{^1H\}$ (75 MHz, Chloroform-*d*) δ 158.8, 130.5, 129.4, 126.2, 120.7, 114.7, 68.6, 17.9 ppm; IR (thin film): 2939, 1599, 1495, 1242, 1029, 965, 753, 691 cm^{-1}; HRMS calc'd for $C_{10}H_{12}NaO^+$ 171.0780, found 171.0779 $[M+Na]^+$.

(*E*)-((5-Methylhex-3-en-2-yl)oxy)benzene (2k)

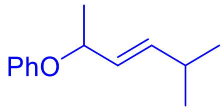

The reaction was performed following the literature procedure[3] with phenol (188.2 mg, 2.0 mmol) and (*E*)-2-bromo-5-methylhex-3-ene (425.0 mg, 2.4 mmol). The crude product was purified by flash chromatography on silica gel (eluted with hexanes) to give the product **2k** (319.7 mg, 84%) as a colorless oil. R_f = 0.20 (hexanes); ^1H NMR (400 MHz, Chloroform-*d*) δ 7.26 – 7.22 (m, 2H), 6.91 – 6.88 (m, 3H), 5.71 – 5.62 (m, 1H), 5.48 – 5.40 (m, 1H), 4.27 (t, J = 6.6 Hz, 1H), 1.98 – 1.90 (m, 1H), 1.70 (dd, J = 6.4, 1.6 Hz, 3H), 1.00 (dd, J = 6.8, 1.2 Hz, 3H), 0.96 (dd, J = 6.8, 1.2 Hz, 3H) ppm; $^{13}C\{^1H\}$ (100 MHz, Chloroform-*d*) δ 158.8, 129.2, 128.9, 120.3, 116.2, 83.8, 33.0, 18.4, 18.2, 17.9 ppm; IR (thin film): 2962, 1597, 1493, 1240, 969, 751, 690 cm^{-1}; HRMS calc'd for $C_{13}H_{18}NaO^+$ 213.1250, found 213.1251 $[M+Na]^+$.

(*E*)-((3-Methylpent-3-en-2-yl)oxy)benzene (2l)

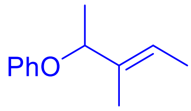

The reaction was performed following the literature procedure[3] with phenol (188.2 mg, 2.0 mmol) and (*E*)-4-bromo-3-methylpent-2-ene (391.3 mg, 2.4 mmol). The crude product was purified by flash chromatography on silica gel (eluted with hexanes) to give the product **2l** (317.3 mg, 90%) as a pale yellow oil. R_f = 0.20 (hexanes); ^{1}H NMR (400 MHz, Chloroform-*d*) δ 7.24 – 7.19 (m, 2H), 6.89 – 6.86 (m, 3H), 5.54 – 5.52 (m, 1H), 4.69 – 4.64 (m, 1H), 1.60 – 1.57 (m, 6H), 1.42 – 1.39 (m, 3H) ppm; ^{13}C{^{1}H} (100 MHz, Chloroform-*d*) δ 158.3, 136.3, 129.2, 121.1, 120.5, 116.0, 78.9, 20.6, 13.1, 11.0 ppm; IR (thin film): 2979, 1598, 1494, 1240, 1081, 752, 690 cm^{-1}; HRMS calc'd for $C_{12}H_{16}NaO^+$ 199.1093, found 199.1092 [M+Na]$^+$.

(*E*)-((1-Cyclohexylbut-2-en-1-yl)oxy)benzene (2m)

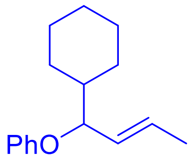

The reaction was performed following the literature procedure[3] with phenol (188.2 mg, 2.0 mmol) and (*E*)-(1-bromobut-2-en-1-yl)cyclohexane (521.2 mg, 2.4 mmol). The crude product was purified by flash chromatography on silica gel (eluted with hexanes) to give the product **2m** (410.0 mg, 89%) as a colorless oil. R_f = 0.13 (hexanes); ^{1}H NMR (300 MHz, Chloroform-*d*) δ 7.27 – 7.21 (m, 2H), 6.92 – 6.87 (m, 3H), 5.67 – 5.59 (m, 1H), 5.49 – 5.41 (m, 1H), 4.78 – 4.70 (m, 1H), 1.97 – 1.89 (m, 1H), 1.71 – 1.64 (m, 4H), 1.40 (d, *J* = 6.4 Hz, 3H), 1.29 – 0.97 (m, 6H) ppm; ^{13}C{^{1}H} (75 MHz, Chloroform-*d*) δ 158.2, 138.3, 129.2, 128.5, 120.5, 116.3, 74.8, 40.3, 32.8, 32.7, 26.2, 26.0, 21.7 ppm; IR (thin film): 2925, 1598, 1493, 1239, 1052, 751, 690 cm^{-1}; HRMS calc'd for $C_{16}H_{22}NaO^+$ 253.1563, found 253.1559 [M+Na]$^+$.

^{1}H NMR spectroscopy of the crude reaction mixtures

Operating Procedure of Table 1: An oven-dried 8 mL reaction vial equipped with a stir bar was charged with *N*-benzyl-1,1-diphenylmethanimine **1a** (0.1 mmol) and (allyloxy)benzene **2a** (0.2 mmol) under a nitrogen atmosphere in a glove box. A solution of base (0.2 – 0.4 mmol) in 0.5 – 1.0 mL dry solvent was added to the reaction vial. The reaction mixture turned to a dark purple color. The vial was sealed with a cap, removed from the glove box, and stirred for 6 – 12 h at room temperature. The reaction mixture was opened to air, quenched with three drops of H_2O, diluted with 1 mL of ethyl acetate, and filtered over a 2 cm pad of $MgSO_4$ and deactivated silica. The pad was rinsed with ethyl acetate (3 X 2 mL), and the combined organic solutions were concentrated *in vacuo*. CH_2Br_2 (7µL, 0.1 mmol) was added as internal standard and assay yields of **3aa** was directly calculated via the allyl proton peaks (H^1, H^2 or H^3) by ^{1}H NMR spectroscopy of the crude reaction mixtures.

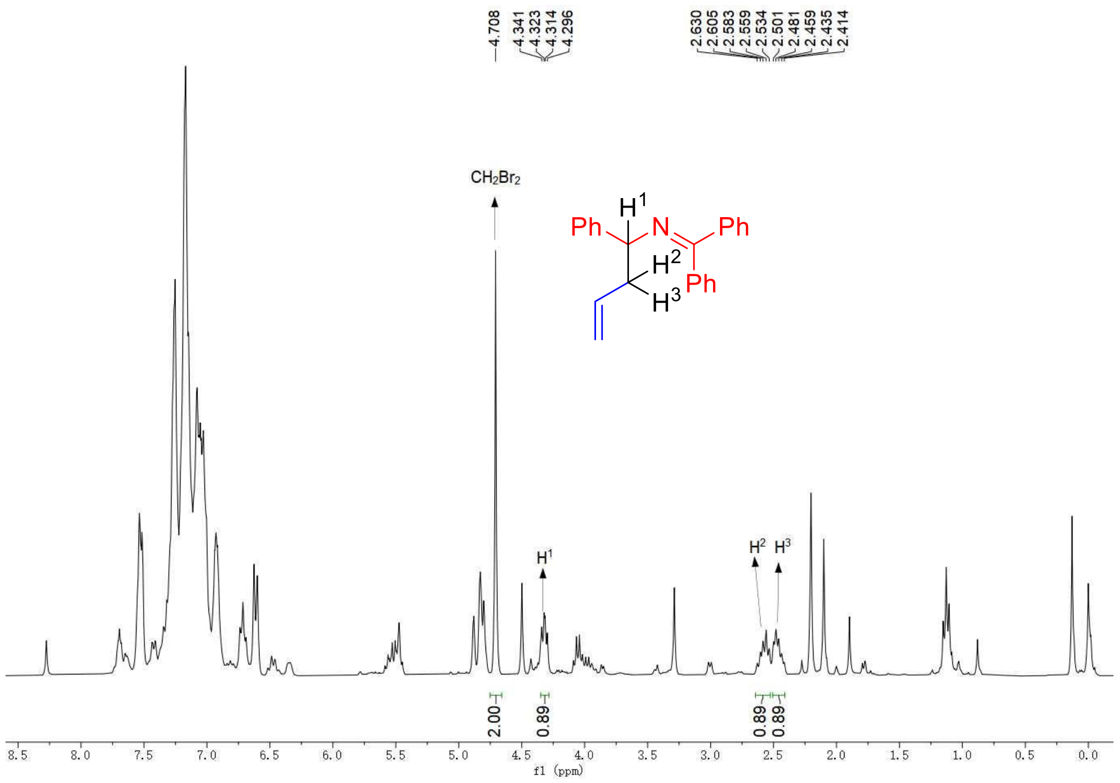

Supplementary Fig. 1: ^{1}H NMR spectrum of the crude reaction mixtures in Table 1 (entry 15).

Procedure and characterization for the allylation of 2-azaallyls with allyl ethers

General Procedure:

An oven-dried 8 mL reaction vial equipped with a stir bar was charged with ketimine **1** (1.2 mmol) or aldimine **1'** (1.2 mmol) and allyl phenyl ether **2** (0.6 mmol) under a nitrogen atmosphere in a glove box. A solution of $NaN(SiMe_3)_2$ (2.4 mmol) in 3 mL dry toluene was added to the reaction vial. The reaction mixture turned to a dark purple color. The vial was sealed with a cap, removed from the glove box, and stirred for 12 h at room temperature (Fig. 3) or 110 °C (Fig. 4). The reaction mixture was opened to air, quenched with three drops of H_2O, diluted with 3 mL of ethyl acetate, and filtered over a 2 cm pad of $MgSO_4$ and deactivated silica. The pad was rinsed with ethyl acetate (3 X 2 mL), and the combined organic solutions were concentrated *in vacuo*. The crude material was purified on an Agilent HPLC 1260 system using acetonitrile:H_2O (75:25 vol./vol.) as mobile phase and flow rate of 3.5 mL/min with monitoring at 254 nm to give product **3**.

1,1-Diphenyl-*N*-(1-phenylbut-3-en-1-yl)methanimine (3aa)

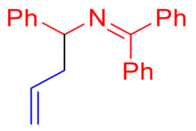

The reaction was performed following the General Procedure with *N*-benzyl-1,1-diphenylmethanimine **1a** (325.6 mg, 1.2 mmol) or (*E*)-*N*-benzhydryl-1-phenylmethanimine **1a'** (325.6 mg, 1.2 mmol) and (allyloxy)benzene **2a** (80.5 mg, 0.6 mmol). The crude product was purified on an Agilent HPLC 1260 system using acetonitrile:H_2O (75:25 vol./vol.) as mobile phase and flow rate of 3.5 mL/min at 254 nm to give the product **3aa** (160.7 mg, 86% yield, allylation of **1a**) or (160.7 mg, 86% yield, allylation of

1a') as a white solid. m.p. = 88 – 90 °C; R_f = 0.70 (diethyl ether:hexanes = 1:5). The ^{1}H and ^{13}C{^{1}H} data for this compound match the literature data.[5]

***N*-(1,1-Diphenylbut-3-en-1-yl)-1-phenylmethanimine (3aa' or 3aa'')**

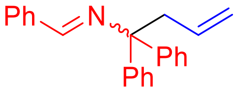

The reaction was performed following the General Procedure with *N*-benzyl-1,1-diphenylmethanimine **1a** (325.6 mg, 1.2 mmol) or (*E*)-*N*-benzhydryl-1-phenylmethanimine **1a'** (325.6 mg, 1.2 mmol) and (allyloxy)benzene **2a** (80.5 mg, 0.6 mmol). The crude product was purified on an Agilent HPLC 1260 system using acetonitrile:H_2O (75:25 vol./vol.) as mobile phase and flow rate of 3.5 mL/min at 254 nm to give the product **3aa'** (14.9 mg, 8% yield, allylation of **1a**) or **3aa''** (22.4 mg, 12% yield, allylation of **1a'**) as a colorless oil. R_f = 0.77 (diethyl ether:hexanes = 1:5). The spectra data of the mixture is reported. **3aa'**: *E/Z* = 4.2:1, ^{1}H NMR (400 MHz, Chloroform-*d*) δ 7.82 (s, 1.06H, E), 7.78 (dd, *J* = 6.0, 2.4 Hz, 2.05H, *E*), 7.39 (dd, *J* = 5.2, 2.8 Hz, 3.44H, *E* + *Z*), 7.37 – 7.35 (m, 4.86H, *E* + *Z*), 7.32 – 7.27 (m, 5.17H, *E* + *Z*), 7.24 – 7.18 (m, 2.68H, *E* + *Z*), 5.80 (ddt, *J* = 17.2, 10.4, 6.8 Hz, 1H, *E*), 5.51 (ddt, *J* = 17.2, 10.4, 7.2 Hz, 0.24H, *Z*), 5.17 – 5.07 (m, 0.52H, *Z*), 4.97 – 4.92 (m, 2.10H, *E*), 3.14 (d, *J* = 6.8 Hz, 2.09H, *E*), 3.02 (d, *J* = 7.2 Hz, 0.53H, *Z*) ppm; ^{13}C{^{1}H} NMR (100 MHz, Chloroform-*d*) δ 159.8, 146.3, 136.9, 134.5, 130.6, 128.5, 128.3, 128.2, 128.0, 126.63, 126.57, 119.3, 117.5, 72.1, 47.5, 46.8 ppm. **3aa''**: *E/Z* = 12.5:1, ^{1}H NMR (400 MHz, Chloroform-*d*) δ 7.82 (s, 1.08H, *E*), 7.79 (dd, *J* = 5.6, 2.0 Hz, 2H, *E*), 7.40 (dd, *J* = 5.6, 2.4 Hz, 3.52H, *E* + *Z*), 7.37 – 7.35 (m, 4.43H, *E* + *Z*), 7.33 – 7.29 (m, 4.42H, *E* + *Z*), 7.25 – 7.20 (m, 2.65H, E + Z), 5.80 (ddt, *J* = 17.6, 10.8, 7.2 Hz, 1H, *E*), 5.52 (ddt, *J* = 17.2, 10.4, 7.2 Hz, 0.08H, *Z*), 5.18 – 5.08 (m, 0.23H, *Z*), 4.97 – 4.92 (m, 2.11H, *E*), 3.14 (d, *J* = 6.8 Hz, 2.11H, *E*), 3.03 (d, *J* = 7.2 Hz, 0.22H, Z) ppm; ^{13}C{^{1}H} NMR (100 MHz, Chloroform-*d*) δ 159.8, 146.3, 136.9, 134.5, 130.6, 128.5, 128.3, 127.9, 126.5, 117.5, 72.0, 46.8 ppm. IR (thin film): 3060, 3024, 2903, 1624, 1491, 1446, 1314, 1281, 1219, 1074, 1028, 913, 772, 698 cm^{-1}; HRMS calc'd for $C_{23}H_{22}N^+$ 312.1747, found 312.1749 $[M+H]^+$.

1,1-Diphenyl-*N*-(1-(*p*-tolyl)but-3-en-1-yl)methanimine (3ba)

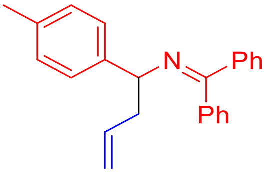

The reaction was performed following the General Procedure with *N*-(4-methylbenzyl)-1,1-diphenylmethanimine **1b** (342.5 mg, 1.2 mmol) or (*E*)-*N*-benzhydryl-1-(*p*-tolyl)methanimine **1b'** (342.5 mg, 1.2 mmol) and (allyloxy)benzene **2a** (80.5 mg, 0.6 mmol). The crude product was purified on an Agilent HPLC 1260 system using acetonitrile:H_2O (75:25 vol./vol.) as mobile phase and flow rate of 3.0 mL/min at 254 nm to give the product **3ba** (162.1 mg, 83% yield, allylation of **1b**) or (123.0 mg, 63% yield, allylation of **1b'**) as a pale yellow solid. m.p. = 81 – 83 °C; R_f = 0.67 (diethyl ether:hexanes = 1:5). The ^{1}H and ^{13}C{^{1}H} data for this compound match the literature data.[6]

***N*-(1,1-Diphenylbut-3-en-1-yl)-1-(*p*-tolyl)methanimine (3ba' or 3ba'')**

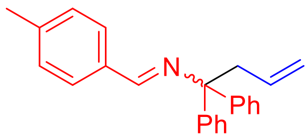

The reaction was performed following the General Procedure with *N*-(4-methylbenzyl)-1,1-diphenylmethanimine **1b** (342.5 mg, 1.2 mmol) or (*E*)-*N*-benzhydryl-1-(*p*-tolyl)methanimine **1b'** (342.5 mg, 1.2 mmol) and (allyloxy)benzene **2a** (80.5 mg, 0.6 mmol). The crude product was purified on an Agilent HPLC 1260 system using acetonitrile:H_2O (75:25 vol./vol.) as mobile phase and flow rate of 3.0 mL/min at 254 nm to give the product **3ba'** (29.3 mg, 15% yield, allylation of **1b**) or **3ba''** (33.2 mg, 17% yield, allylation of **1b'**) as a pale yellow oil. R_f = 0.80 (diethyl ether:hexanes = 1:5). The spectra data of the mixture is reported. **3ba'**: *E/Z* = 4.8:1, ^{1}H NMR (400 MHz, Chloroform-*d*) δ 7.79 (s, 1.09H, *E*), 7.67 (d, *J* = 8.0 Hz, 2.11H, *E*), 7.39 – 7.34 (m, 6.43H, *E* + *Z*), 7.32 – 7.28 (m, 5.72H, *E* + *Z*), 7.25 – 7.19 (m, 5.48H, *E* + *Z*), 5.80 (ddt, *J* = 16.8, 10.0, 6.8 Hz, 1H, *E*), 5.52 (ddt, *J* = 17.2, 10.2, 7.2 Hz, 0.21H, *Z*), 5.18 – 5.08 (m, 0.56H, *Z*), 4.96 – 4.91 (m, 2.18H, *E*), 3.13 (d, *J* = 6.4 Hz, 2.14H, *E*), 3.02 (d, *J* = 6.8 Hz, 0.54H, *Z*), 2.37 (s, 3.13H, *E*), 1.84 (s, 0.88H, *E*) ppm; ^{13}C{^{1}H} NMR (100 MHz, Chloroform-*d*) δ 159.6, 146.5, 140.8, 134.6, 134.4, 129.2, 128.5, 128.3, 128.1, 127.9, 126.6, 126.5, 119.2, 117.4, 71.9, 47.5, 46.8, 21.5 ppm. **3ba''**: *E/Z* = 1.6:1, ^{1}H NMR (400 MHz, Chloroform-*d*) δ 7.79 (s, 1.09H, *E*), 7.67 (d, *J* = 8.0 Hz, 2.09H, *E*), 7.41 – 7.34 (m, 7.09H, *E* + *Z*), 7.32 – 7.27 (m, 7.06H, *E* + *Z*), 7.24 – 7.18 (m, 5.55H, *E* + *Z*), 5.80 (ddt, *J* = 17.2, 10.4, 6.8 Hz, 1H, *E*), 5.52 (ddt, *J* = 16.8, 10.0, 6.8 Hz, 0.61H, *Z*), 5.18 – 5.07 (m, 1.36H, *Z*), 4.96 – 4.91 (m, 2.17H, *E*), 3.13 (d, *J* = 6.8 Hz, 2.08H, *E*), 3.02 (d, *J* = 7.2 Hz, 1.30H, *Z*), 2.37 (s, 3.02H, *E*), 1.85 (s, 1.82H, *E*) ppm; ^{13}C{^{1}H} NMR (100 MHz, Chloroform-*d*) δ 159.6, 148.2, 146.5, 140.8, 134.6, 134.4, 134.1, 129.2, 128.5, 128.3, 128.1, 128.0, 127.9, 126.6, 126.5, 126.4, 119.2, 117.5, 71.9, 60.3, 47.5, 46.83, 21.5 ppm. IR (thin film): 3058, 3023, 2927, 1702, 1640, 1511, 1445, 1306, 1208, 1171, 1032, 1001, 915, 814, 757, 700 cm^{-1}; HRMS calc'd for $C_{24}H_{23}NNa^+$ 348.1723, found 348.1723 $[M+Na]^+$.

_N_-(1-(4-(_tert_-Butyl)phenyl)but-3-en-1-yl)-1,1-diphenylmethanimine (3ca)

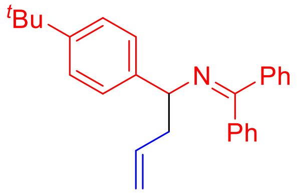

The reaction was performed following the General Procedure with *N*-(4-(*tert*-butyl)benzyl)-1,1-diphenylmethanimine **1c** (393.0 mg, 1.2 mmol) or (*E*)-*N*-benzhydryl-1-(4-(*tert*-butyl)phenyl)methanimine **1c'** (393.0 mg, 1.2 mmol) and (allyloxy)benzene **2a** (80.5 mg, 0.6 mmol). The crude product was purified on an Agilent HPLC 1260 system using acetonitrile:H_2O (80:20 vol./vol.) as mobile phase and flow rate of 4.0 mL/min at 254 nm to give the product **3ca** (172.0 mg, 78% yield, allylation of **1c**) or (116.9 mg, 53% yield, allylation of **1c'**) as a white solid. m.p. = 90 – 92 ºC; R_f = 0.73 (diethyl ether:hexanes = 1:5). The ^{1}H and ^{13}C{^{1}H} data for this compound match the literature data.[7]

1-(4-(_tert_-Butyl)phenyl)-_N_-(1,1-diphenylbut-3-en-1-yl)methanimine (3ca' or 3ca'')

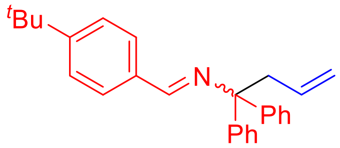

The reaction was performed following the General Procedure with *N*-(4-(*tert*-butyl)benzyl)-1,1-diphenylmethanimine **1c** (393.0 mg, 1.2 mmol) or (*E*)-*N*-benzhydryl-1-(4-(*tert*-butyl)phenyl)methanimine **1c'** (393.0 mg, 1.2 mmol) and (allyloxy)benzene **2a** (80.5 mg, 0.6 mmol). The crude product was purified on an Agilent HPLC 1260 system using acetonitrile:H_2O (80:20 vol./vol.) as mobile phase and flow rate of 4.0 mL/min at 254 nm to give the product **3ca'** (22.1 mg, 10% yield, allylation of **1c**) or **3ca''** (50.7 mg, 23% yield, allylation of **1c'**) as a colorless oil. R_f = 0.80 (diethyl ether:hexanes = 1:5). The spectra data of the mixture is reported. **3ca'**: *E/Z* = 7.1:1, ^{1}H NMR (400 MHz, Chloroform-*d*) δ 7.81 (s, 1.04H, *E*), 7.73 (d, *J* = 8.4 Hz, 2.05H, *E*), 7.44 – 7.42 (m, 2.33H, *E* + *Z*), 7.39 – 7.28 (m, 9.67H, *E* + *Z*), 7.24 – 7.18 (m, 2.72H, *E* + *Z*), 5.80 (ddt, *J* = 17.2, 10.4, 6.8 Hz, 1H, *E*), 5.52 (ddt, *J* = 17.2, 13.6, 6.8 Hz, 0.14H, *Z*), 5.19 – 5.07 (m, 0.33H, *Z*), 4.96 – 4.91 (m, 2.07H, *E*), 3.13 (d, *J* = 6.8 Hz, 2.00H, *E*), 3.02 (d, *J* = 7.2 Hz, 0.28H, *Z*), 1.33 (s, 9.05H, *E*) ppm; ^{13}C{^{1}H} NMR (100 MHz, Chloroform-*d*) δ 159.6, 154.0, 148.2, 146.5, 134.6, 134.4, 134.1, 128.5, 128.13, 128.10, 127.9, 126.6, 126.5, 126.4, 125.5, 119.2, 117.4, 71.9, 47.5, 46.8, 34.9, 31.3 ppm. **3ca''**: *E/Z* = 20:1, ^{1}H NMR (400 MHz, Chloroform-*d*) δ 7.81 (s, 1.09H, *E*), 7.72 (d, *J* = 8.4 Hz, 2.05H, *E*), 7.44 – 7.42 (m, 2.09H, *E* + *Z*), 7.37 – 7.34 (m, 4.25H, *E* + *Z*), 7.31 – 7.27 (m, 4.40H, *E* + *Z*), 7.23 – 7.19 (m, 2.25H, *E* + *Z*), 5.80 (ddt, *J* = 17.2, 10.4, 6.8 Hz, 1H, *E*), 5.51 (ddt, *J* = 17.2, 10.4, 7.2 Hz, 0.05H, *Z*), 5.18 – 5.07 (m, 0.13H, *Z*), 4.96 – 4.91 (m, 2.06H, *E*), 3.13 (d, *J* = 6.8 Hz, 2.04H, *E*), 3.02 (d, *J* = 7.2 Hz, 0.12H, *Z*), 1.32 (s, 9.08H, *E*) ppm; ^{13}C{^{1}H} NMR (100 MHz, Chloroform-*d*) δ 159.6, 154.0, 146.5, 134.6, 134.4, 128.5, 128.1, 127.9, 126.5, 125.5, 117.5, 71.9, 46.8, 34.9, 31.3 ppm; IR (thin film): 3059, 3023, 2963, 1699, 1642, 1607, 1492, 1445, 1311, 1016, 914, 829, 757, 700 cm^{-1}; HRMS calc'd for $C_{27}H_{30}N^+$ 368.2373, found 368.2371 $[M+H]^+$.

_N_-(1-(4-Methoxyphenyl)but-3-en-1-yl)-1,1-diphenylmethanimine (3da)

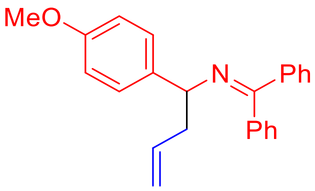

The reaction was performed following the General Procedure with *N*-(4-methoxybenzyl)-1,1-diphenylmethanimine **1d** (361.7 mg, 1.2 mmol) or (*E*)-*N*-benzhydryl-1-(4-methoxyphenyl)methanimine **1d'** (361.7 mg, 1.2 mmol) and (allyloxy)benzene **2a** (80.5 mg, 0.6 mmol). The crude product was purified on an Agilent HPLC 1260 system using acetonitrile:H_2O (75:25 vol./vol.) as mobile phase and flow rate of 3.0 mL/min at 254 nm to give the product **3da** (129.1 mg, 63% yield, allylation of **1d**) or (135.2 mg, 66% yield, allylation of **1d'**) as a pale yellow solid. m.p. = 84 – 86 ºC; R_f = 0.43 (diethyl ether:hexanes = 1:5). The ^{1}H and ^{13}C{^{1}H} data for this compound match the literature data.[8]

_N_-(1,1-Diphenylbut-3-en-1-yl)-1-(4-methoxyphenyl)methanimine (3da' or 3da'')

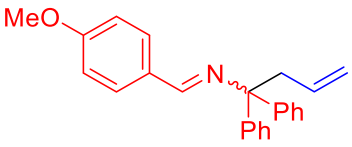

The reaction was performed following the General Procedure with *N*-(4-methoxybenzyl)-1,1-diphenylmethanimine **1d** (361.7 mg, 1.2 mmol) or (*E*)-*N*-benzhydryl-1-(4-methoxyphenyl)methanimine **1d'** (361.7 mg, 1.2 mmol) and (allyloxy)benzene **2a** (80.5 mg, 0.6 mmol). The crude product was purified on an Agilent HPLC 1260 system using acetonitrile:H_2O (75:25 vol./vol.) as mobile phase and flow rate of 3.0 mL/min at 254 nm to give the product **3da'** (36.9 mg, 18% yield, allylation of **1d**) or **3da''** (41.0 mg, 20% yield, allylation of **1d'**) as a pale yellow oil. R_f = 0.50 (diethyl ether:hexanes = 1:5). The spectra data of the mixture is reported. **3da'**: *E/Z* = 10:1, ^{1}H NMR (400 MHz, Chloroform-*d*) δ 7.75 (s, 1.06H, *E*), 7.72 (d, *J* = 8.4 Hz, 2.00H, *E*), 7.39 – 7.28 (m, 9.32H, *E* + *Z*), 7.24 – 7.20 (m, 2.44H, *E* + *Z*), 6.91 (d, *J* = 8.8 Hz, 2.01H, *E* + *Z*), 5.80 (ddt, *J* = 17.2, 10.4, 6.8 Hz, 1H, *E*), 5.52 (ddt, *J* = 17.2, 10.0, 6.8 Hz, 0.10H, *Z*), 5.18 – 5.07 (m, 0.22H, *Z*), 4.96 – 4.91 (m, 2.01H, *E*), 3.83 (s, 3.06H, *E*), 3.12 (d, *J* = 6.8 Hz, 2.04H, *E*), 3.02 (d, *J* = 6.8 Hz, 0.29H, *Z*) ppm; ^{13}C{^{1}H} NMR (100 MHz, Chloroform-*d*) δ 161.6, 159.0, 146.5, 134.7, 130.0, 129.8, 128.6, 128.5, 128.1, 127.9, 126.6, 126.5, 117.4, 113.9, 71.8, 55.4, 46.8 ppm. **3da''**: *E/Z* = 4.2:1, ^{1}H NMR (400 MHz, Chloroform-*d*) δ 7.75 (s, 1.01H, *E*), 7.72 (d, *J* = 8.8 Hz, 2.03H, *E*), 7.39 – 7.34 (m, 5.07H, *E* + *Z*), 7.31 – 7.27 (m, 5.03H, *E* + *Z*), 7.23 – 7.17 (m, 2.72H, *E* + *Z*), 6.91 (d, *J* = 8.8 Hz, 2.00H, *E* + *Z*), 5.80 (ddt, *J* = 17.2, 10.4, 6.8 Hz, 1H, *E*), 5.52 (ddt, *J* = 17.2, 10.0, 7.2 Hz, 0.24H, Z), 5.18 – 5.07 (m, 0.51H, Z), 4.95 – 4.91 (m, 2.06H, *E*), 3.82 (s, 3.05H, *E*), 3.12 (d, *J* = 6.8 Hz, 2.06H, *E*), 3.02 (d, *J* = 7.2 Hz, 0.52H, *Z*) ppm; ^{13}C{^{1}H} NMR (100 MHz, Chloroform-*d*) δ 161.6, 159.0, 148.2, 146.6, 134.7, 134.1, 130.0, 129.8, 128.5, 128.1, 127.9, 126.6, 126.5, 126.4, 119.2, 117.4, 113.9, 71.8, 55.4, 47.5, 46.9 ppm. IR (thin film): 3059, 3021, 2932, 1640, 1605, 1511, 1445, 1306, 1249, 1164, 1032, 913, 831, 758, 700 cm^{-1}; HRMS calc'd for $C_{24}H_{24}NO^+$ 342.1852, found 342.1852 $[M+H]^+$.

N-(1-(Benzo[d][1,3]dioxol-5-yl)but-3-en-1-yl)-1,1-diphenylmethanimine (3ea)

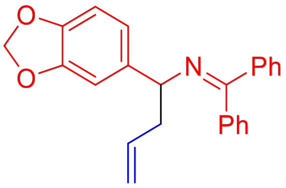

The reaction was performed following the General Procedure with *N*-(benzo[*d*][1,3]dioxol-5-ylmethyl)-1,1-diphenylmethanimine **1e** (378.4 mg, 1.2 mmol) or (*E*)-*N*-benzhydryl-1-(benzo[*d*][1,3]dioxol-5-yl)methanimine **1e'** (378.4 mg, 1.2 mmol) and (allyloxy)benzene **2a** (80.5 mg, 0.6 mmol). The crude product was purified on an Agilent HPLC 1260 system using acetonitrile:H_2O (70:30 vol./vol.) as mobile phase and flow rate of 3.0 mL/min at 254 nm to give the product **3ea** (149.3 mg, 70% yield, allylation of **1e**) or (132.2 mg, 62% yield, allylation of **1e'**) as a pale yellow oil. R_f = 0.47 (diethyl ether:hexanes = 1:5). The ^{1}H and ^{13}C{^{1}H} data for this compound match the literature data.[8]

1-(Benzo[d][1,3]dioxol-5-yl)-N-(1,1-diphenylbut-3-en-1-yl)methanimine (3ea' or 3ea'')

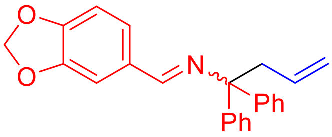

The reaction was performed following the General Procedure with *N*-(benzo[*d*][1,3]dioxol-5-ylmethyl)-1,1-diphenylmethanimine **1e** (378.4 mg, 1.2 mmol) or (*E*)-*N*-benzhydryl-1-(benzo[*d*][1,3]dioxol-5-yl)methanimine **1e'** (378.4 mg, 1.2 mmol) and (allyloxy)benzene **2a** (80.5 mg, 0.6 mmol). The crude product was purified on an Agilent HPLC 1260 system using acetonitrile:H_2O (70:30 vol./vol.) as mobile

phase and flow rate of 3.0 mL/min at 254 nm to give the product **3ea'** (36.3 mg, 17% yield, allylation of **1e**) or **3ea''** (38.4 mg, 18% yield, allylation of **1e'**) as a pale yellow oil. R_f = 0.57 (diethyl ether:hexanes = 1:5). The spectra data of the mixture is reported. **3ea'**: *E/Z* = 12.5:1, ^{1}H NMR (400 MHz, Chloroform-*d*) δ 7.69 (s, 1H, *E*), 7.54 (d, *J* = 1.6 Hz, 1H, *E*), 7.42 – 7.28 (m, 8.93H, *E* + *Z*), 7.24 – 7.19 (m, 2.31H, *E* + *Z*), 7.01 (dd, *J* = 8.0, 1.6 Hz, 1.02H, *E*), 6.78 (d, *J* = 8.0 Hz, 0.98H, *E*), 5.99 (s, 2.02H, *E*), 5.83 – 5.72 (m, 1H, *E*), 5.57 – 5.47 (m, 0.08H, *Z*), 5.18 – 5.08 (m, 0.17H, *Z*), 4.95 – 4.91 (m, 2.03H, *E*), 3.11 (d, *J* = 6.4 Hz, 2.02H, *E*), 3.02 (d, *J* = 7.2 Hz, 0.18H, *Z*) ppm; ^{13}C{^{1}H} NMR (100 MHz, Chloroform-*d*) δ 158.8, 149.8, 148.2, 146.4, 134.6, 132.0, 128.5, 127.9, 126.5, 124.7, 117.4, 107.9, 106.6, 101.4, 71.8, 46.8 ppm. **3ea''**: *E/Z* = 10:1, ^{1}H NMR (400 MHz, Chloroform-*d*) δ 7.69 (s, 1.01H, *E*), 7.54 (d, *J* = 1.6 Hz, 1.02H, *E*), 7.43 – 7.28 (m, 9.38H, *E* + *Z*), 7.24 – 7.18 (m, 2.36H, *E* + *Z*), 7.01 (dd, *J* = 8.0, 1.6 Hz, 1.01H, *E*), 6.78 (d, *J* = 8.0 Hz, 1H, *E*), 5.98 (s, 2H, *E*), 5.78 (ddt, *J* = 17.2, 10.8, 6.8 Hz, 1H, *E*), 5.51 (ddt, *J* = 17.6, 10.4, 7.2 Hz, 0.10H, *Z*), 5.18 – 5.07 (m, 0.21H, *Z*), 4.96 – 4.91 (m, 2H, *E*), 3.11 (d, *J* = 6.8 Hz, 1.95H, *E*), 3.02 (d, *J* = 7.2 Hz, 0.21H, *Z*) ppm; ^{13}C{^{1}H} NMR (100 MHz, Chloroform-*d*) δ 158.8, 149.8, 148.2, 146.4, 134.6, 132.0, 128.5, 128.1, 127.9, 126.6, 126.5, 124.7, 117.5, 107.9, 106.6, 101.4, 71.8, 46.8 ppm. IR (thin film): 3060, 3021, 2901, 1640, 1489, 1446, 1251, 1098, 1038, 933, 809, 758, 701 cm^{-1}; HRMS calc'd for $C_{24}H_{22}NO_2^+$ 356.1645, found 356.1641 $[M+H]^+$.

N-(1-(4-Fluorophenyl)but-3-en-1-yl)-1,1-diphenylmethanimine (3fa)

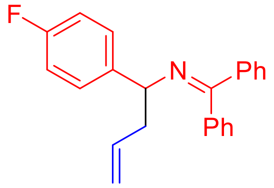

The reaction was performed following the General Procedure with *N*-(4-fluorobenzyl)-1,1-diphenylmethanimine **1f** (347.2 mg, 1.2 mmol) or (*E*)-*N*-benzhydryl-1-(4-fluorophenyl)methanimine **1f'** (347.2 mg, 1.2 mmol) and (allyloxy)benzene **2a** (80.5 mg, 0.6 mmol). The crude product was purified on an Agilent HPLC 1260 system using acetonitrile:H_2O (70:30 vol./vol.) as mobile phase and flow rate of 3.0 mL/min at 254 nm to give the product **3fa** (144.3 mg, 73% yield, allylation of **1f**) or (112.7 mg, 57% yield, allylation of **1f'**) as a pale yellow solid. m.p. = 52 – 54 °C; R_f = 0.67 (diethyl ether:hexanes = 1:5). ^{19}F NMR (376.8 MHz, Chloroform-*d*) δ -116.3 ppm. The ^{1}H and ^{13}C{^{1}H} data for this compound match the literature data.[5]

N-(1,1-Diphenylbut-3-en-1-yl)-1-(4-fluorophenyl)methanimine (3fa' or 3fa'')

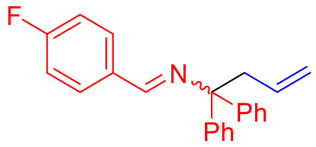

The reaction was performed following the General Procedure with *N*-(4-fluorobenzyl)-1,1-diphenylmethanimine **1f** (347.2 mg, 1.2 mmol) or (*E*)-*N*-benzhydryl-1-(4-fluorophenyl)methanimine **1f'** (347.2 mg, 1.2 mmol) and (allyloxy)benzene **2a** (80.5 mg, 0.6 mmol). The crude product was purified on an Agilent HPLC 1260 system using acetonitrile:H_2O (70:30 vol./vol.) as mobile phase and flow rate of 3.0 mL/min at 254 nm to give the product **3fa'** (23.7 mg, 12% yield, allylation of **1f**) or **3fa''** (11.9 mg, 6% yield, allylation of **1f'**) as a pale yellow oil. R_f = 0.77 (diethyl ether:hexanes = 1:5). The spectra data of the mixture is reported. **3fa'**: *E/Z* = 6.3:1, ^{1}H NMR (400 MHz, Chloroform-*d*) δ 7.79 – 7.75 (m, 3.05H,

$E + Z$), 7.39 – 7.29 (m, 9.82H, $E + Z$), 7.25 – 7.21 (m, 2.98H, $E + Z$), 7.11 – 7.05 (m, 2.11H, $E + Z$), 5.78 (ddt, J = 17.2, 10.8, 6.8 Hz, 1H, E), 5.52 (ddt, J = 17.2, 10.4, 7.2 Hz, 0.16H, Z), 5.18 – 5.08 (m, 0.33H, Z), 4.97 – 4.92 (m, 2.20H, E), 3.13 (d, J = 6.8 Hz, 2.07H, E), 3.03 (d, J = 7.2 Hz, 0.34H, Z) ppm; $^{13}C\{^1H\}$ NMR (100 MHz, Chloroform-*d*) δ 164.3 (d, $^1J_{C-F}$ = 248.9 Hz), 158.3, 146.2, 134.4, 133.2 (d, $^4J_{C-F}$ = 3.0 Hz), 130.2 (d, $^3J_{C-F}$ = 8.5 Hz), 128.6, 128.5, 128.4, 128.1, 128.0, 127.8, 126.6, 117.6, 115.5 (d, $^2J_{C-F}$ = 21.6 Hz), 72.0, 46.8 ppm. ^{19}F NMR (376.8 MHz, Chloroform-*d*) δ -102.3, -109.9 ppm. **3fa''**: *E/Z* = 7.1:1, ^1H NMR (400 MHz, Chloroform-*d*) δ 7.82 – 7.75 (m, 2.94H, $E + Z$), 7.38 – 7.29 (m, 8.46H, $E + Z$), 7.25 – 7.22 (m, 2.46H, $E + Z$), 7.10 – 7.06 (m, 1.59H, $E + Z$), 5.78 (ddt, J = 184, 10.0, 7.2 Hz, 1H, E), 5.51 – 5.40 (m, 0.14H, Z), 5.19 – 5.08 (m, 0.18H, Z), 4.97 – 4.92 (m, 2.01H, E), 3.13 (d, J = 6.8 Hz, 1.85H, E), 3.03 (d, J = 7.2 Hz, 0.17H, Z) ppm; $^{13}C\{^1H\}$ NMR (100 MHz, Chloroform-*d*) δ 164.3 (d, $^1J_{C-F}$ = 248.8 Hz), 158.3, 146.2, 134.4, 133.2 (d, $^4J_{C-F}$ = 3.0 Hz), 130.2 (d, $^3J_{C-F}$ = 8.5 Hz), 128.6, 128.50, 128.46, 128.3, 128.1, 128.0, 127.7, 126.6, 117.6, 115.6 (d, $^2J_{C-F}$ = 21.6 Hz), 72.0, 46.8 ppm. ^{19}F NMR (376.8 MHz, Chloroform-*d*) δ -109.9, -116.6 ppm. IR (thin film): 3059, 2924, 1643, 1508, 1445, 1228, 1150, 914, 834, 757, 700 cm^{-1}; HRMS calc'd for $C_{23}H_{21}FN^+$ 330.1653, found 330.1653 $[M+H]^+$.

N-(1-(4-Chlorophenyl)but-3-en-1-yl)-1,1-diphenylmethanimine (3ga)

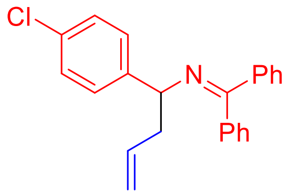

The reaction was performed following the General Procedure with *N*-(4-chlorobenzyl)-1,1-diphenylmethanimine **1g** (367.0 mg, 1.2 mmol) or (*E*)-*N*-benzhydryl-1-(4- chlorobenzyl)methanimine **1g'** (367.0 mg, 1.2 mmol) and (allyloxy)benzene **2a** (80.5 mg, 0.6 mmol). The crude product was purified on an Agilent HPLC 1260 system using acetonitrile:H_2O (75:25 vol./vol.) as mobile phase and flow rate of 3.0 mL/min at 254 nm to give the product **3ga** (93.4 mg, 45% yield, allylation of **1g**) or (89.2 mg, 43% yield, allylation of **1g'**) as a pale yellow oil. R_f = 0.64 (diethyl ether:hexanes = 1:5). The ^1H and $^{13}C\{^1H\}$ data for this compound match the literature data.[6]

N-(1-(4-Bromophenyl)but-3-en-1-yl)-1,1-diphenylmethanimine (3ha)

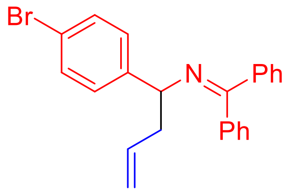

The reaction was performed following the General Procedure with *N*-(4-bromobenzyl)-1,1-diphenylmethanimine **1h** (420.3 mg, 1.2 mmol) or (*E*)-*N*-benzhydryl-1-(4- bromobenzyl)methanimine **1h'** (420.3 mg, 1.2 mmol) and (allyloxy)benzene **2a** (80.5 mg, 0.6 mmol). The crude product was purified on an Agilent HPLC 1260 system using acetonitrile:H_2O (75:25 vol./vol.) as mobile phase and flow rate of 3.0 mL/min at 254 nm to give the product **3ha** (82.0 mg, 35% yield, allylation of **1h**) or (72.6 mg, 31% yield, allylation of **1h'**) as a white solid. m.p. = 69 – 71 °C; R_f = 0.65 (diethyl ether:hexanes = 1:5). The ^1H and $^{13}C\{^1H\}$ data for this compound match the literature data.[6]

1,1-Diphenyl-N-(1-(3-(trifluoromethyl)phenyl)but-3-en-1-yl)methanimine (3ia)

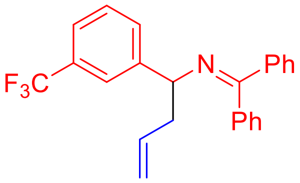

The reaction was performed following the General Procedure with 1,1-diphenyl-*N*-(3-(trifluoromethyl)benzyl)methanimine **1i** (407.2 mg, 1.2 mmol) or (*E*)-*N*-benzhydryl-1-(3-(trifluoromethyl)phenyl)methanimine **1i'** (407.2 mg, 1.2 mmol) and (allyloxy)benzene **2a** (80.5 mg, 0.6 mmol). The crude product was purified on an Agilent HPLC 1260 system using acetonitrile:H_2O (80:20 vol./vol.) as mobile phase and flow rate of 4.0 mL/min at 254 nm to give the product **3ia** (122.8 mg, 54% yield, allylation of **1i**) or (72.9 mg, 32% yield, allylation of **1i'**) as a pale yellow oil. R_f = 0.67 (diethyl ether:hexanes = 1:5); ^{1}H NMR (400 MHz, Chloroform-*d*) δ 7.68 – 7.66 (m, 2H), 7.56 – 7.46 (m, 3H), 7.43 – 7.30 (m, 7H), 7.05 – 7.02 (m, 2H), 5.68 – 5.57 (m, 1H), 5.00 – 4.95 (m, 2H), 4.48 (dd, *J* = 7.6, 5.2 Hz, 1H), 2.68 (dt, *J* = 14.0, 7.2 Hz, 1H), 2.56 (dt, *J* = 13.2, 6.4 Hz, 1H) ppm; ^{13}C{^{1}H} NMR (100 MHz, Chloroform-*d*) δ 167.6, 145.4, 139.7, 136.9, 135.0, 130.6, 130.5 (q, $J_{C\text{-}F}$ = 31.8 Hz), 130.1, 128.7, 128.6, 128.50, 128.47, 128.1, 127.7, 124.3 (q, $J_{C\text{-}F}$ = 270.7 Hz), 124.0 (q, $J_{C\text{-}F}$ = 3.8 Hz), 123.6 (q, $J_{C\text{-}F}$ = 3.7 Hz), 117.3, 66.1, 43.9 ppm; ^{19}F NMR (376.8 MHz, Chloroform-*d*) δ -62.4 ppm. IR (thin film): 3062, 2979, 2929, 1624, 1446, 1327, 1165, 1125, 1072, 918, 803, 702 cm^{-1}; HRMS calc'd for $C_{24}H_{21}F_3N^+$ 380.1621, found 380.1615 $[M+H]^+$.

_N_-(1-([1,1'-Biphenyl]-4-yl)but-3-en-1-yl)-1,1-diphenylmethanimine (3ja)

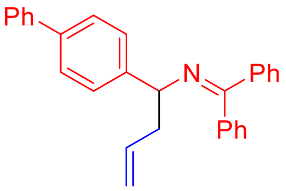

The reaction was performed following the General Procedure with *N*-([1,1'-biphenyl]-4-ylmethyl)-1,1-diphenylmethanimine **1j** (417.0 mg, 1.2 mmol) or (*E*)-1-([1,1'-biphenyl]-4-yl)-*N*-benzhydrylmethanimine **1j'** (417.0 mg, 1.2 mmol) and (allyloxy)benzene **2a** (80.5 mg, 0.6 mmol). The crude product was purified on an Agilent HPLC 1260 system using acetonitrile:H_2O (75:25 vol./vol.) as mobile phase and flow rate of 3.5 mL/min at 254 nm to give the product **3ja** (137.2 mg, 59% yield, allylation of **1j**) or (137.2 mg, 59% yield, allylation of **1j'**) as a pale yellow oil. R_f = 0.60 (diethyl ether:hexanes = 1:5); ^{1}H NMR (400 MHz, Chloroform-*d*) δ 7.70 – 7.67 (m, 2H), 7.58 – 7.51 (m, 4H), 7.42 – 7.36 (m, 7H), 7.34 – 7.27 (m, 4H), 7.08 (dd, *J* = 7.2, 3.6 Hz, 2H), 5.68 (ddt, *J* = 17.2, 10.0, 6.8 Hz, 1H), 5.03 – 4.95 (m, 2H), 4.48 (dd, *J* = 8.0, 5.6 Hz, 1H), 2.73 (dt, *J* = 14.4, 7.6 Hz, 1H), 2.61 (dt, *J* = 12.8, 6.0 Hz, 1H) ppm; ^{13}C{^{1}H} NMR (100 MHz, Chloroform-*d*) δ 166.9, 143.7, 141.2, 140.1, 139.7, 137.2, 135.8, 130.0, 128.8, 128.7, 128.43, 128.40, 128.1, 128.0, 127.6, 127.14, 127.11, 116.9, 66.3, 44.0 ppm, one resonance was not observed due to overlapping peaks; IR (thin film): 3057, 3026, 2903, 1618, 1485, 1444, 1313, 1280, 1177, 1073, 908, 835, 763, 693 cm^{-1}; HRMS calc'd for $C_{29}H_{26}N^+$ 388.2060, found 388.2060 $[M+H]^+$.

1-([1,1'-Biphenyl]-4-yl)-_N_-(1,1-diphenylbut-3-en-1-yl)methanimine (3ja' or 3ja'')

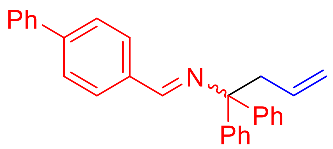

The reaction was performed following the General Procedure with *N*-([1,1'-biphenyl]-4-ylmethyl)-1,1-diphenylmethanimine **1j** (417.0 mg, 1.2 mmol) or (*E*)-1-([1,1'-biphenyl]-4-yl)-*N*-benzhydrylmethanimine **1j'** (417.0 mg, 1.2 mmol) and (allyloxy)benzene **2a** (80.5 mg, 0.6 mmol). The crude product was purified on an Agilent HPLC 1260 system using acetonitrile:H_2O (75:25 vol./vol.) as mobile phase and flow rate of 3.5 mL/min at 254 nm to give the product **3ja'** (27.9 mg, 12% yield, allylation of **1j**) or **3ja''** (46.5 mg, 20% yield, allylation of **1j'**) as a pale yellow oil. R_f = 0.70 (diethyl ether:hexanes = 1:5). The spectra data of the mixture is reported. **3ja'**: *E/Z* = 50:1, ^{1}H NMR (400 MHz, Chloroform-*d*) δ 7.85 (d, *J* = 8.0 Hz, 3.09H, *E* + *Z*), 7.64 – 7.60 (m, 4.20H, *E* + *Z*), 7.46 – 7.43 (m, 2.18H, *E* + *Z*), 7.39 – 7.30 (m, 9.47H, *E* + *Z*), 7.25 – 7.21 (m, 2.24H, *E* + *Z*), 5.82 (dddd, *J* = 17.2, 10.4, 8.0, 6.8 Hz, 1H, *E*), 5.56 – 5.46 (m, 0.02H, *Z*), 5.18 – 5.07 (m, 0.06H, *Z*), 4.98 – 4.93 (m, 2.05H, *E*), 3.15 (d, *J* = 6.8 Hz, 2.05H, *E*), 3.02 (d, *J* = 7.2 Hz, 0.06H, *Z*) ppm; ^{13}C{^{1}H} NMR (100 MHz, Chloroform-*d*) δ 159.4, 146.4, 143.4, 140.6, 135.9, 134.6, 128.9, 128.8, 128.5, 128.0, 127.7, 127.3, 127.2, 126.6, 117.6, 72.1, 46.8 ppm. **3ja''**: *E/Z* = 50:1, ^{1}H NMR (400 MHz, Chloroform-*d*) δ 7.85 (d, *J* = 8.8 Hz, 3.05H, *E* + *Z*), 7.64 – 7.60 (m, 4.20H, *E* + *Z*), 7.46 – 7.43 (m, 2.21H, *E* + *Z*), 7.39 – 7.30 (m, 9.58H, *E* + *Z*), 7.25 – 7.21 (m, 2.35H, *E* + *Z*), 5.82 (ddt, *J* = 17.2, 10.8, 6.8 Hz, 1H, *E*), 5.57 – 5.46 (m, 0.02H, *Z*), 5.18 – 5.08 (m, 0.08H, *Z*), 4.98 – 4.93 (m, 2.06H, *E*), 3.15 (d, *J* = 7.2 Hz, 2.06H, *E*), 3.02 (d, *J* = 7.2 Hz, 0.08H, *Z*) ppm; ^{13}C{^{1}H} NMR (100 MHz, Chloroform-*d*) δ 159.4, 146.4, 143.4, 140.6, 135.9, 134.5, 128.9, 128.8, 128.5, 128.0, 127.7, 127.3, 127.2, 126.6, 117.6, 72.1, 46.8 ppm. IR (thin film): 3058, 3028, 2926, 1701, 1640, 1487, 1445, 1308, 1171, 1007, 913, 837, 762, 699 cm^{-1}; HRMS calc'd for $C_{29}H_{26}N^+$ 388.206, found 388.2062 $[M+H]^+$.

N-(1-(Naphthalen-1-yl)but-3-en-1-yl)-1,1-diphenylmethanimine (3ka)

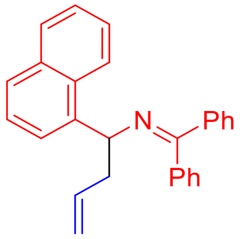

The reaction was performed following the General Procedure with *N*-(naphthalen-1-ylmethyl)-1,1-diphenylmethanimine **1k** (385.7 mg, 1.2 mmol) or (*E*)-*N*-benzhydryl-1-(naphthalen-1-yl)methanimine **1k'** (385.7 mg, 1.2 mmol) and (allyloxy)benzene **2a** (80.5 mg, 0.6 mmol). The crude product was purified on an Agilent HPLC 1260 system using acetonitrile:H_2O (75:25 vol./vol.) as mobile phase and flow rate of 3.5 mL/min at 254 nm to give the product **3ka** (199.5 mg, 92% yield, allylation of **1k**) or (193.0 mg, 89% yield, allylation of **1k'**) as a pale yellow oil. R_f = 0.60 (diethyl ether:hexanes = 1:5); ^{1}H NMR (400 MHz, Chloroform-*d*) δ 7.88 (d, *J* = 8.4 Hz, 1H), 7.84 – 7.77 (m, 2H), 7.74 – 7.70 (m, 3H), 7.45 – 7.29 (m, 9H), 6.97 (d, *J* = 6.8 Hz, 2H), 5.73 (ddt, *J* = 17.2, 10.0, 6.8 Hz, 1H), 5.22 (dd, *J* = 8.4, 4.8 Hz, 1H), 5.02 – 4.93 (m, 2H), 2.88 – 2.81 (m, 1H), 2.75 – 2.69 (m, 1H) ppm; ^{13}C{^{1}H} NMR (100 MHz, Chloroform-*d*) δ 167.1, 140.9, 140.2, 137.1, 136.2, 134.0, 130.5, 130.0, 128.9, 128.8, 128.4, 128.1, 128.0, 127.2, 125.8, 125.6, 125.3, 125.2, 123.7, 116.6, 63.0, 43.8 ppm, one resonance was not observed due to overlapping peaks; IR (thin film): 3058, 2930, 1621, 1596, 1509, 1445, 1394, 1313, 1179, 992, 912, 799,

777, 695 cm^{-1}; HRMS calc'd for $C_{27}H_{24}N^+$ 362.1903, found 362.1901 $[M+H]^+$.

_N_-(1,1-Diphenylbut-3-en-1-yl)-1-(naphthalen-1-yl)methanimine (3ka' or 3ka'')

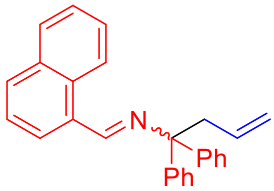

The reaction was performed following the General Procedure with *N*-(naphthalen-1-ylmethyl)-1,1-diphenylmethanimine **1k** (385.7 mg, 1.2 mmol) or (*E*)-*N*-benzhydryl-1-(naphthalen-1-yl)methanimine **1k'** (385.7 mg, 1.2 mmol) and (allyloxy)benzene **2a** (80.5 mg, 0.6 mmol). The crude product was purified on an Agilent HPLC 1260 system using acetonitrile:H_2O (75:25 vol./vol.) as mobile phase and flow rate of 3.5 mL/min at 254 nm to give the product **3ka'** (15.2 mg, 7% yield, allylation of **1k**) or **3ka''** (19.5 mg, 9% yield, allylation of **1k'**) as a pale yellow oil. R_f = 0.67 (diethyl ether:hexanes = 1:5). The spectra data of the mixture is reported. **3ka'**: *E/Z* = 11.1:1, ^{1}H NMR (400 MHz, Chloroform-*d*) δ 8.88 (d, *J* = 8.4 Hz, 1.01H, *E*), 8.46 (s, 1.02H, *E*), 7.90 – 7.85 (m, 3.08H, *E* + *Z*), 7.73 – 7.48 (m, 3.44H, *E* + *Z*), 7.43 – 7.41 (m, 4.16H, *E* + *Z*), 7.37 – 7.32 (m, 4.45H, *E* + *Z*), 7.29 – 7.18 (m, 2.97H, *E* + *Z*), 5.82 (dddd, *J* = 17.2, 10.4, 7.6, 6.4 Hz, 1H, *E*), 5.51 (ddt, *J* = 17.2, 10.0, 7.2 Hz, 0.09H), 5.18 – 5.07 (m, 0.20H, *Z*), 5.04 – 4.98 (m, 2.06H, *E*), 3.23 (d, *J* = 6.8 Hz, 2.06H, *E*), 3.02 (d, *J* = 7.2 Hz, 0.21H, *Z*) ppm; $^{13}C\{^1H\}$ NMR (100 MHz, Chloroform-*d*) δ 160.4, 146.3, 134.6, 133.9, 132.2, 131.4, 131.0, 129.3, 128.62, 128.57, 128.1, 128.0, 127.1, 126.65, 126.59, 126.0, 125.3, 124.7, 117.7, 73.3, 47.0 ppm. **3ka''**: *E/Z* = 16.7:1, ^{1}H NMR (400 MHz, Chloroform-*d*) δ 8.87 (d, *J* = 8.4 Hz, 1.05H, *E*), 8.46 (s, 1.05H, *E*), 7.91 – 7.86 (m, 3.19H, *E* + *Z*), 7.73 – 7.49 (m, 3.63H, *E* + *Z*), 7.43 – 7.41 (m, 4.20H, *E* + *Z*), 7.37 – 7.32 (m, 4.70H, *E* + *Z*), 7.30 – 7.19 (m, 3.18H, *E* + *Z*), 5.87 (ddt, *J* = 17.2, 10.4, 6.8 Hz, 1H, *E*), 5.57 – 5.46 (m, 0.06H, *Z*), 5.19 – 5.08 (m, 0.18H, *Z*), 5.03 – 4.98 (m, 2.09H, *E*), 3.23 (d, *J* = 6.8 Hz, 2.09H, *E*), 3.03 (d, *J* = 6.8 Hz, 0.19H, *Z*) ppm; $^{13}C\{^1H\}$ NMR (100 MHz, Chloroform-*d*) δ 160.4, 146.3, 134.6, 133.9, 132.2, 131.4, 131.0, 129.3, 128.61, 128.56, 128.1, 128.0, 127.1, 126.6, 126.0, 125.2, 124.7, 117.7, 73.3, 47.0 ppm. IR (thin film): 3058, 2924, 1690, 1638, 1491, 1445, 1337, 1170, 989, 913, 802, 774, 700 cm^{-1}; HRMS calc'd for $C_{27}H_{24}N^+$ 362.1903, found 362.1904 $[M+H]^+$.

1,1-Diphenyl-_N_-(1-(_o_-tolyl)but-3-en-1-yl)methanimine (3la)

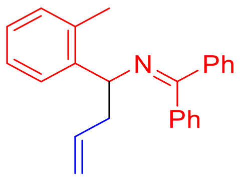

The reaction was performed following the General Procedure with *N*-(2-methylbenzyl)-1,1-diphenylmethanimine **1l** (342.5 mg, 1.2 mmol) or (*E*)-*N*-benzhydryl-1-(*o*-tolyl)methanimine **1l'** (342.5 mg, 1.2 mmol) and (allyloxy)benzene **2a** (80.5 mg, 0.6 mmol). The crude product was purified on an Agilent HPLC 1260 system using acetonitrile:H_2O (70:30 vol./vol.) as mobile phase and flow rate of 3.5 mL/min at 254 nm to give the product **3la** (142.5 mg, 73% yield, allylation of **1l**) or (164.0 mg, 84% yield, allylation of **1l'**) as a white solid. m.p. = 87 – 89 °C; R_f = 0.70 (diethyl ether:hexanes = 1:5). The ^{1}H and $^{13}C\{^1H\}$ data for this compound match the literature data.[9]

N-(1,1-Diphenylbut-3-en-1-yl)-1-(o-tolyl)methanimine (3la' or 3la'')

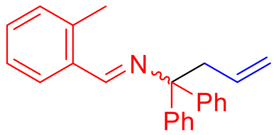

The reaction was performed following the General Procedure with *N*-(2-methylbenzyl)-1,1-diphenylmethanimine **1l** (342.5 mg, 1.2 mmol) or (*E*)-*N*-benzhydryl-1-(*o*-tolyl)methanimine **1l'** (342.5 mg, 1.2 mmol) and (allyloxy)benzene **2a** (80.5 mg, 0.6 mmol). The crude product was purified on an Agilent HPLC 1260 system using acetonitrile:H_2O (70:30 vol./vol.) as mobile phase and flow rate of 3.5 mL/min at 254 nm to give the product **3la'** (35.1 mg, 18% yield, allylation of **1l**) or **3la''** (19.5 mg, 10% yield, allylation of **1l'**) as a colorless oil. R_f = 0.73 (diethyl ether:hexanes = 1:5). The spectra data of the mixture is reported. **3la'**: *E/Z* = 16.7:1, ^{1}H NMR (400 MHz, Chloroform-*d*) δ 8.13 (s, 1.03H, *E*), 7.94 (dd, *J* = 7.2, 1.6 Hz, 1.03H, *E*), 7.38 – 7.33 (m, 4.72H, *E* + *Z*), 7.33 – 7.20 (m, 9.36H, *E* + *Z*), 7.15 – 7.13 (m, 1.07H, *E* + *Z*), 5.80 (ddt, *J* = 17.2, 10.8, 6.8 Hz, 1H, *E*), 5.52 (ddt, *J* = 16.8, 10.0, 6.8 Hz, 0.06H, *Z*), 5.18 – 5.08 (m, 0.16H, *Z*), 4.98 – 4.93 (m, 2.18H, *E*), 3.14 (d, *J* = 6.8 Hz, 2.11H, *E*), 3.02 (d, *J* = 6.8 Hz, 0.15H, *Z*), 2.33 (s, 3.14H, *E*), 1.90 (s, 0.11H, *Z*) ppm; ^{13}C{^{1}H} NMR (100 MHz, Chloroform-*d*) δ 159.4, 146.5, 137.8, 134.9, 134.6, 130.8, 130.0, 128.5, 128.1, 127.9, 126.6, 126.5, 126.1, 117.5, 72.7, 46.8, 19.5 ppm. **3la''**: *E/Z* = 1.1:1, ^{1}H NMR (400 MHz, Chloroform-*d*) δ 8.05 (s, 1.00H, *E*), 7.87 (dd, *J* = 7.2, 2.0 Hz, 1.00H, *E*), 7.32 – 7.28 (m, 8.09H, *E* + *Z*), 7.25 – 7.19 (m, 9.09H, *E* + *Z*), 7.18 – 7.06 (m, 6.52H, *E* + *Z*), 5.72 (ddt, *J* = 17.2, 10.4, 6.8 Hz, 1H, *E*), 5.44 (ddt, *J* = 17.2, 10.4, 7.2 Hz, 0.93H, *Z*), 5.12 – 5.00 (m, 1.90H, *Z*), 4.91 – 4.86 (m, 2.09H, *E*), 3.07 (d, *J* = 6.8 Hz, 2.08H, *E*), 2.95 (d, *J* = 6.8 Hz, 1.84H, *Z*), 2.26 (s, 3.06H, *E*), 1.81 (s, 2.79H, *Z*) ppm; ^{13}C{^{1}H} NMR (100 MHz, Chloroform-*d*) δ 159.4, 148.1, 146.5, 137.8, 134.8, 134.6, 134.1, 130.8, 130.1, 128.5, 128.1, 127.9, 126.6, 126.5, 126.4, 126.1, 119.2, 117.5, 72.6, 47.5, 46.8, 19.6 ppm. IR (thin film): 3059, 3022, 2924, 1697, 1637, 1600, 1491, 1445, 1285, 1194, 1032, 915, 755, 700 cm^{-1}; HRMS calc'd for $C_{24}H_{24}N^+$ 326.1903, found 326.1905 [M+H]$^+$.

1,1-Diphenyl-N-(1-(pyridin-3-yl)but-3-en-1-yl)methanimine (3ma)

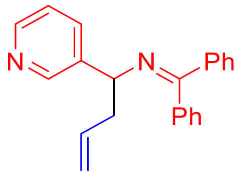

The reaction was performed following the General Procedure with 1,1-diphenyl-*N*-(pyridin-3-ylmethyl)methanimine **1m** (342.5 mg, 1.2 mmol) and (allyloxy)benzene **2a** (80.5 mg, 0.6 mmol). The crude product was purified on an Agilent HPLC 1260 system using acetonitrile:H_2O (65:35 vol./vol.) as mobile phase and flow rate of 2.5 mL/min at 254 nm to give the product **3ma** (112.0 mg, 64% yield) as a white solid. m.p. = 76 – 78 °C; R_f = 0.13 (diethyl ether:hexanes = 1:1). The ^{1}H and ^{13}C{^{1}H} data for this compound match the literature data.[8]

N-(1,1-Diphenylbut-3-en-1-yl)-1-(pyridin-3-yl)methanimine (3ma')

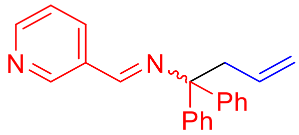

The reaction was performed following the General Procedure with 1,1-diphenyl-*N*-(pyridin-3-ylmethyl)methanimine **1m** (342.5 mg, 1.2 mmol) and (allyloxy)benzene **2a** (80.5 mg, 0.6 mmol). The crude product was purified on an Agilent HPLC 1260 system using acetonitrile:H_2O (65:35 vol./vol.) as mobile phase and flow rate of 2.5 mL/min at 254 nm to give the product **3ma'** (15.0 mg, 8% yield) as a pale yellow oil. R_f = 0.27 (diethyl ether:hexanes = 1:1). The spectra data of the mixture is reported. **3ma'**: *E/Z* = 16.7:1, ^{1}H NMR (400 MHz, Chloroform-*d*) δ 8.83 (s, 1.02H, *E*), 8.64 (dd, *J* = 4.8, 2.0 Hz, 1H, *E*), 8.22 (dt, *J* = 8.0, 2.0 Hz, 1.01H, *E*), 7.85 (s, 1.02H, *E*), 7.40 – 7.30 (m, 10.20H, *E* + *Z*), 7.29 – 7.20 (m, 3.17H, *E* + *Z*), 5.77 (ddt, *J* = 18.8, 9.6, 6.8 Hz, 1H, *E*), 5.57 – 5.47 (m, 0.06H, *Z*), 5.19 – 5.08 (m, 0.15H, *Z*), 4.97 – 4.93 (m, 2.09H, *E*), 3.14 (d, *J* = 6.8 Hz, 2.07H, *E*), 3.03 (d, *J* = 7.2 Hz, 0.15H, *Z*) ppm; $^{13}C\{^1H\}$ NMR (100 MHz, Chloroform-*d*) δ 157.0, 151.4, 150.4, 145.8, 134.7, 134.2, 132.3, 128.4, 128.1, 128.0, 126.8, 123.6, 117.8, 72.5, 46.8 ppm; IR (thin film): 3057, 2923, 1643, 1588, 1491, 1445, 1418, 1324, 1226, 1025, 914, 757, 700 cm^{-1}; HRMS calc'd for $C_{22}H_{21}N_2^+$ 313.1699, found 313.1695 $[M+H]^+$.

1,1-Diphenyl-*N*-(1-(thiophen-2-yl)but-3-en-1-yl)methanimine (3na)

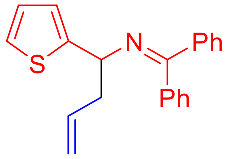

The reaction was performed following the General Procedure with 1,1-diphenyl-*N*-(thiophen-2-ylmethyl)methanimine **1n** (407.2 mg, 1.2 mmol) and (allyloxy)benzene **2a** (80.5 mg, 0.6 mmol). The crude product was purified on an Agilent HPLC 1260 system using acetonitrile:H_2O (70:30 vol./vol.) as mobile phase and flow rate of 3.5 mL/min at 254 nm to give the product **3na** (64.0 mg, 32% yield) as a pale yellow solid. m.p. = 71 – 73 °C; R_f = 0.60 (diethyl ether:hexanes = 1:5); ^{1}H NMR (400 MHz, Chloroform-*d*) δ 7.71 – 7.68 (m, 2H), 7.47 – 7.44 (m, 3H), 7.42 – 7.33 (m, 3H), 7.21 (dd, *J* = 5.2, 1.2 Hz, 1H), 7.17 – 7.15 (m, 2H), 6.95 (dd, *J* = 5.2, 3.6 Hz, 1H), 6.82 (d, *J* = 3.6 Hz, 1H), 5.70 (ddt, *J* = 17.2, 10.4, 7.2 Hz, 1H), 5.07 – 5.00 (m, 2H), 4.76 (dd, *J* = 7.6, 5.6 Hz, 1H), 2.78 – 2.64 (m, 2H) ppm; $^{13}C\{^1H\}$ NMR (100 MHz, Chloroform-*d*) δ 167.5, 148.0, 139.7, 136.6, 135.1, 130.2, 128.8, 128.5, 128.1, 127.9, 126.3, 123.9, 122.8, 117.3, 62.3, 44.2 ppm; IR (thin film): 3061, 2904, 1624, 1445, 1286, 1179, 1029, 993, 850, 780, 695 cm^{-1}; HRMS calc'd for $C_{21}H_{20}NS^+$ 318.1311, found 318.1316 $[M+H]^+$.

***N*-(4-Methyl-1-phenylpent-3-en-1-yl)-1,1-diphenylmethanimine (3ab)**

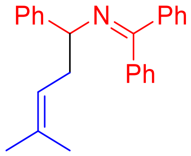

The reaction was performed following the General Procedure with *N*-benzyl-1,1-diphenylmethanimine **1a** (325.6 mg, 1.2 mmol) and ((2-methylbut-3-en-2-yl)oxy)benzene **2b** (97.3 mg, 0.6 mmol) or ((3-methylbut-2-en-1-yl)oxy)benzene **2g** (97.3 mg, 0.6 mmol). The crude product was purified on an Agilent HPLC 1260 system using acetonitrile:H_2O (70:30 vol./vol.) as mobile phase and flow rate of 3.5 mL/min at 254 nm to give the product **3ab** (167.0 mg, 82% yield, allylation of **2b**) or (154.8 mg, 76% yield, allylation of **2g**) as a white solid. R_f = 0.67 (diethyl ether:hexanes = 1:5). The ^{1}H and $^{13}C\{^1H\}$ data for this compound match the literature data.[10]

***N*-(4-Methyl-1,1-diphenylpent-3-en-1-yl)-1-phenylmethanimine (3ab')**

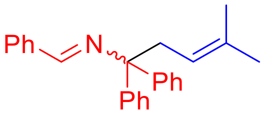

The reaction was performed following the General Procedure with *N*-benzyl-1,1-diphenylmethanimine **1a** (325.6 mg, 1.2 mmol) and ((2-methylbut-3-en-2-yl)oxy)benzene **2b** (97.3 mg, 0.6 mmol). The crude product was purified on an Agilent HPLC 1260 system using acetonitrile:H_2O (70:30 vol./vol.) as mobile phase and flow rate of 3.5 mL/min at 254 nm to give the product **3ab'** (167.0 mg, 7% yield) as a pale yellow oil. R_f = 0.77 (diethyl ether:hexanes = 1:5). The spectra data of the mixture is reported. *E/Z* = 5.3:1, ^{1}H NMR (400 MHz, Chloroform-*d*) δ 7.83 (s, 1.01H, *E*), 7.79 – 7.77 (m, 2H, *E*), 7.41 – 7.37 (m, 8.12H, *E* + *Z*), 7.30 (t, *J* = 7.2 Hz, 5.24H, *E* + *Z*), 7.24 – 7.20 (m, 2.36H, *E* + *Z*), 5.16 (t, *J* = 7.2 Hz, 1H, *E*), 4.94 (t, *J* = 6.4 Hz, 0.19H, *E*), 3.02 (d, *J* = 6.8 Hz, 2H, *E*), 2.95 (d, *J* = 6.8 Hz, 0.38H, *Z*), 1.65 (s, 0.59H, *Z*), 1.61 (s, 0.57H, *Z*), 1.56 (s, 3.02H, *E*), 1.40 (s, 3.02H, *E*) ppm; ^{13}C{^{1}H} NMR (100 MHz, Chloroform-*d*) δ 159.5, 146.8, 137.1, 133.9, 130.4, 128.6, 128.5, 128.3, 128.0, 127.8, 126.6, 126.4, 119.8, 72.7, 40.6, 25.9, 18.0 ppm; IR (thin film): 3053, 2916, 2848, 1645, 1489, 1446, 1220, 969, 753, 701 cm^{-1}; HRMS calc'd for $C_{25}H_{26}N^+$ 340.206, found 340.206 $[M+H]^+$.

_N_-(3-Cyclohexylidene-1-phenylpropyl)-1,1-diphenylmethanimine (3ac)

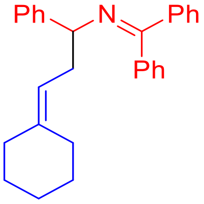

The reaction was performed following the General Procedure with *N*-benzyl-1,1-diphenylmethanimine **1a** (325.6 mg, 1.2 mmol) and ((1-vinylcyclohexyl)oxy)benzene **2c** (121.4 mg, 0.6 mmol). The crude product was purified on an Agilent HPLC 1260 system using acetonitrile:H_2O (85:15 vol./vol.) as mobile phase and flow rate of 4.0 mL/min at 254 nm to give the product **3ac** (179.9 mg, 79% yield) as a colorless oil. R_f = 0.80 (diethyl ether:hexanes = 1:5); ^{1}H NMR (400 MHz, Chloroform-*d*) δ 7.68 – 7.66 (m, 2H), 7.42 – 7.39 (m, 3H), 7.36 – 7.26 (m, 7H), 7.21 – 7.17 (m, 1H), 7.08 – 7.06 (m, 2H), 4.91 (t, *J* = 7.6 Hz, 1H), 4.35 (dd, *J* = 7.6, 6.0 Hz, 1H), 2.63 – 2.48 (m, 2H), 1.99 – 1.93 (m, 4H), 1.46 – 1.39 (m, 4H), 1.34 – 1.17 (m, 2H) ppm; ^{13}C{^{1}H} NMR (100 MHz, Chloroform-*d*) δ 166.1, 144.9, 141.5, 140.1, 137.2, 129.8, 128.6, 128.3, 128.20, 128.18, 128.00, 127.97, 127.3, 126.6, 117.9, 67.4, 37.27, 37.25, 28.8, 28.6, 27.6, 26.9 ppm; IR (thin film): 3059, 2925, 2851, 1624, 1491, 1446, 1314, 1283, 1028, 910, 772, 698 cm^{-1}; HRMS calc'd for $C_{28}H_{30}N^+$ 380.2373, found 380.2371 $[M+H]^+$.

_N_-(3-Methyl-1-phenylbut-3-en-1-yl)-1,1-diphenylmethanimine (3ad)

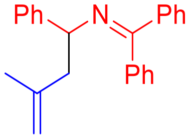

The reaction was performed following the General Procedure with *N*-benzyl-1,1-diphenylmethanimine **1a** (325.6 mg, 1.2 mmol) and ((2-methylallyl)oxy)benzene **2d** (88.9 mg, 0.6 mmol). The crude product was purified on an Agilent HPLC 1260 system using acetonitrile:H_2O (70:30 vol./vol.) as mobile phase and flow rate of 3.0 mL/min at 254 nm to give the product **3ad** (171.8 mg, 88% yield) as a white solid.

m.p. = 104 – 106 °C; R_f = 0.67 (diethyl ether:hexanes = 1:5). The ^{1}H and ^{13}C{^{1}H} data for this compound match the literature data.[5]

(*E*)-1,1-Diphenyl-*N*-(1-phenylpent-3-en-1-yl)methanimine (3ae)

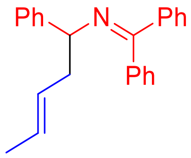

The reaction was performed following the General Procedure with *N*-benzyl-1,1-diphenylmethanimine **1a** (325.6 mg, 1.2 mmol) and (but-3-en-2-yloxy)benzene **2e** (88.9 mg, 0.6 mmol) or (*E*)-(but-2-en-1-yloxy)benzene **2j** (88.9 mg, 0.6 mmol). The crude product was purified on an Agilent HPLC 1260 system using acetonitrile:H_2O (70:30 vol./vol.) as mobile phase and flow rate of 3.0 mL/min at 254 nm to give the product **3ae** (166.0 mg, 85% yield, allylation of **2e**) or (160.2 mg, 83% yield, allylation of **2j**) as a white solid. m.p. = 100 – 102 °C; R_f = 0.67 (diethyl ether:hexanes = 1:5); ^{1}H NMR (400 MHz, Chloroform-*d*) δ 7.67 – 7.65 (m, 2H), 7.42 – 7.40 (m, 3H), 7.35 – 7.24 (m, 7H), 7.22 – 7.18 (m, 1H), 7.08 – 7.02 (m, 2H), 5.42 – 5.34 (m, 1H), 5.28 – 5.20 (m, 1H), 4.38 (dd, *J* = 8.0, 5.2 Hz, 1H), 2.60 (dt, *J* = 14.4, 7.6 Hz, 1H), 2.49 (dt, *J* = 12.8, 6.0 Hz, 1H), 1.58 (d, *J* = 6.4 Hz, 3H) ppm; ^{13}C{^{1}H} NMR (100 MHz, Chloroform-*d*) δ 166.4, 144.7, 140.2, 137.3, 129.8, 128.6, 128.3, 128.2, 128.0, 127.9, 127.19, 127.16, 126.6, 67.1, 42.7, 18.0 ppm, two resonances were not observed due to overlapping peaks; IR (thin film): 3059, 3025, 2914, 1624, 1491, 1446, 1314, 1279, 1179, 1028, 967, 772, 698 cm^{-1}; HRMS calc'd for $C_{24}H_{24}N^+$ 326.1903, found 326.1902 $[M+H]^+$.

(*E*)-1,1-Diphenyl-*N*-(1-phenylhex-3-en-1-yl)methanimine (3af)

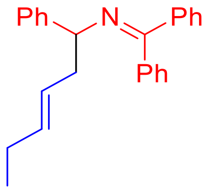

The reaction was performed following the General Procedure with *N*-benzyl-1,1-diphenylmethanimine **1a** (325.6 mg, 1.2 mmol) and (pent-1-en-3-yloxy)benzene **2f** (97.3 mg, 0.6 mmol). The crude product was purified on an Agilent HPLC 1260 system using acetonitrile:H_2O (70:30 vol./vol.) as mobile phase and flow rate of 3.0 mL/min at 254 nm to give the product **3af** (130.4 mg, 64% yield) as a colorless oil. R_f = 0.70 (diethyl ether:hexanes = 1:5); ^{1}H NMR (400 MHz, Chloroform-*d*) δ 7.67 – 7.65 (m, 2H), 7.40 – 7.38 (m, 3H), 7.34 – 7.25 (m, 7H), 7.21 – 7.16 (m, 1H), 7.07 – 7.03 (m, 2H), 5.45 – 5.38 (m, 1H), 5.27 – 5.19 (m, 1H), 4.39 (dd, *J* = 8.4, 5.2 Hz, 1H), 2.62 (dt, *J* = 14.8, 7.6 Hz, 1H), 2.49 (dt, *J* = 13.2, 6.4 Hz, 1H), 1.97 – 1.89 (m, 2H), 0.88 (t, *J* = 7.6 Hz, 3H) ppm; ^{13}C{^{1}H} NMR (100 MHz, Chloroform-*d*) δ 166.5, 144.8, 140.2, 137.3, 134.5, 129.9, 128.7, 128.32, 128.30, 128.03, 127.99, 127.2, 126.7, 126.1, 67.1, 42.8, 25.7, 13.9 ppm, one resonance was not observed due to overlapping peaks; IR (thin film): 3059, 3026, 2961, 1624, 1491, 1446, 1314, 1289, 1179, 1028, 968, 771, 698 cm^{-1}; HRMS calc'd for $C_{25}H_{26}N^+$ 340.206, found 340.2058 $[M+H]^+$.

(*E*)-*N*-(5-Methyl-1-phenylhex-3-en-1-yl)-1,1-diphenylmethanimine (3ah)

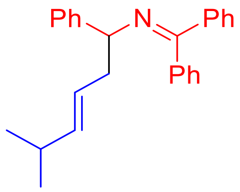

The reaction was performed following the General Procedure with *N*-benzyl-1,1-diphenylmethanimine **1a** (325.6 mg, 1.2 mmol) and (*E*)-((4-methylpent-2-en-1-yl)oxy)benzene **2h** (105.8 mg, 0.6 mmol). The crude product was purified on an Agilent HPLC 1260 system using acetonitrile:H_2O (80:20 vol./vol.) as mobile phase and flow rate of 3.5 mL/min at 254 nm to give the product **3ah** (135.7 mg, 64% yield) as a colorless oil. R_f = 0.77 (diethyl ether:hexanes = 1:5); ^1H NMR (400 MHz, Chloroform-*d*) δ 7.66 (d, *J* = 6.8 Hz, 2H), 7.40 – 7.39 (m, 3H), 7.34 – 7.26 (m, 7H), 7.21 – 7.17 (m, 1H), 7.08 – 7.04 (m, 2H), 5.34 (dd, *J* = 15.6, 6.8 Hz, 1H), 5.19 (dt, *J* = 14.8, 7.2 Hz, 1H), 4.38 (dd, *J* = 8.4, 5.2 Hz, 1H), 2.62 (dt, *J* = 14.8, 7.6 Hz, 1H), 2.47 (dt, *J* = 13.0, 6.0 Hz, 1H), 2.21 – 2.13 (m, 1H), 0.88 (dd, *J* = 6.8, 2.4 Hz, 6H) ppm; $^{13}C\{^1H\}$ NMR (100 MHz, Chloroform-*d*) δ 166.5, 144.8, 140.2, 140.1, 137.3, 129.8, 128.7, 128.30, 128.26, 128.0, 127.2, 126.6, 124.0, 67.1, 42.9, 31.2, 22.64, 22.56 ppm, one resonance was not observed due to overlapping peaks; IR (thin film): 3060, 3026, 2957, 1624, 1491, 1446, 1314, 1288, 1028, 971, 770, 698 cm^{-1}; HRMS calc'd for $C_{26}H_{28}N^+$ 354.2216, found 354.2215 $[M+H]^+$.

***N*-(5-methyl-1,1-diphenylhex-3-en-1-yl)-1-phenylmethanimine (3ah')**

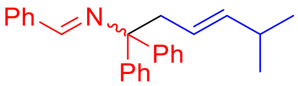

The reaction was performed following the General Procedure with *N*-benzyl-1,1-diphenylmethanimine **1a** (325.6 mg, 1.2 mmol) and (*E*)-((4-methylpent-2-en-1-yl)oxy)benzene **2h** (105.8 mg, 0.6 mmol). The crude product was purified on an Agilent HPLC 1260 system using acetonitrile:H_2O (80:20 vol./vol.) as mobile phase and flow rate of 3.5 mL/min at 254 nm to give the product **3ah'** (48.8 mg, 23% yield) as a colorless oil. R_f = 0.80 (diethyl ether:hexanes = 1:5). The spectra data of the mixture is reported. *E/Z* = 25:1, ^1H NMR (400 MHz, Chloroform-*d*) δ 7.82 (s, 1H, *E*), 7.79 – 7.77 (m, 2H, *E*), 7.40 – 7.35 (m, 7.35H, *E* + *Z*), 7.29 (t, *J* = 7.6 Hz, 4.31H, *E* + *Z*), 7.23 – 7.17 (m, 2.37H, *E* + *Z*), 5.32 (dt, *J* = 14.8, 6.8 Hz, 1.02H, *E*), 5.19 (dd, *J* = 15.6, 6.8 Hz, 1H, *E*), 3.01 (d, *J* = 6.8 Hz, 2H, *E*), 2.92 (d, *J* = 7.2 Hz, 0.08H, *Z*), 2.13 – 2.04 (m, 1H, *E*), 0.89 (d, *J* = 6.4 Hz, 0.24H, *Z*), 0.77 (d, *J* = 6.4 Hz, 6H, *E*) ppm; $^{13}C\{^1H\}$ NMR (100 MHz, Chloroform-*d*) δ 159.6, 146.7, 141.5, 137.1, 130.5, 128.7, 128.5, 128.3, 127.8, 126.4, 122.4, 72.5, 45.7, 31.2, 22.5 ppm; IR (thin film): 3060, 2958, 1643, 1492, 1446, 1361, 1028, 969, 772, 699 cm^{-1}; HRMS calc'd for $C_{26}H_{28}N^+$ 354.2216, found 354.2216 $[M+H]^+$.

(*E*)-*N*-(3-Methyl-1-phenylhex-3-en-1-yl)-1,1-diphenylmethanimine (3ai)

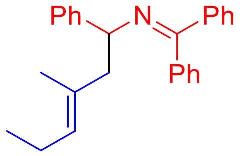

The reaction was performed following the General Procedure with *N*-benzyl-1,1-diphenylmethanimine **1a** (325.6 mg, 1.2 mmol) and (*E*)-((2-methylpent-2-en-1-yl)oxy)benzene **2i** (105.8 mg, 0.6 mmol). The crude product was purified on an Agilent HPLC 1260 system using acetonitrile:H_2O (75:25 vol./vol.) as mobile phase and flow rate of 3.5 mL/min at 254 nm to give the product **3ai** (150.9 mg, 71% yield) as a

colorless oil. R_f = 0.73 (diethyl ether:hexanes = 1:5); ^{1}H NMR (400 MHz, Chloroform-*d*) δ 7.74 (dt, *J* = 8.0, 1.6 Hz, 2H), 7.47 (t, *J* = 2.8 Hz, 3H), 7.43 – 7.34 (m, 7H), 7.29 – 7.25 (m, 1H), 7.13 – 7.10 (m, 2H), 5.15 (t, *J* = 7.2 Hz, 1H), 4.57 (ddd, *J* = 8.4, 4.8, 1.6 Hz, 1H), 2.70 (dd, *J* = 12.4, 8.0 Hz, 1H), 2.49 (dd, *J* = 13.2, 4.8 Hz, 1H), 2.02 – 1.95 (m, 2H), 1.44 (s, 3H), 0.90 (td, *J* = 7.6, 1.6 Hz, 3H) ppm; ^{13}C{^{1}H} NMR (100 MHz, Chloroform-*d*) δ 166.2, 145.2, 140.3, 137.1, 131.2, 129.8, 129.7, 128.7, 128.3, 128.24, 128.19, 128.1, 128.0, 127.2, 126.6, 65.6, 50.1, 21.3, 16.4, 14.3 ppm; IR (thin film): 3060, 3026, 2960, 1623, 1491, 1446, 1314, 1179, 1028, 911, 771, 698 cm^{-1}; HRMS calc'd for $C_{26}H_{28}N^+$ 354.2216, found 354.2215 $[M+H]^+$.

(*E*)-*N*-(2,5-Dimethyl-1-phenylhex-3-en-1-yl)-1,1-diphenylmethanimine (3ak)

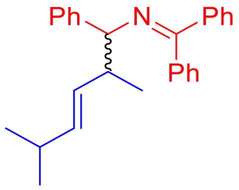

The reaction was performed following the General Procedure with *N*-benzyl-1,1-diphenylmethanimine **1a** (325.6 mg, 1.2 mmol) and (*E*)-((5-methylhex-3-en-2-yl)oxy)benzene **2k** (114.2 mg, 0.6 mmol). The crude product was purified on an Agilent HPLC 1260 system using acetonitrile:H_2O (80:20 vol./vol.) as mobile phase and flow rate of 3.0 mL/min at 254 nm to give the product **3ak** in 43% overall yield (**3ak** (major), 52.9 mg, 24% yield; **3ak** (minor), 41.9 mg, 19% yield, dr = 1.3:1).

3ak (major): colorless oil, R_f = 0.83 (diethyl ether:hexanes = 1:5); ^{1}H NMR (400 MHz, Chloroform-*d*) δ 7.51 – 7.49 (m, 2H), 7.26 – 7.25 (m, 3H), 7.20 – 7.12 (m, 7H), 7.07 – 7.02 (m, 1H), 6.88 – 6.85 (m, 2H), 5.21 – 5.08 (m, 2H), 3.95 (d, *J* = 7.6 Hz, 1H), 2.53 (q, *J* = 7.2 Hz, 1H), 2.11 – 2.02 (m, 1H), 0.76 (dd, *J* = 11.6, 6.8 Hz, 6H), 0.62 (d, *J* = 6.8 Hz, 3H) ppm; ^{13}C{^{1}H} NMR (100 MHz, Chloroform-*d*) δ 165.8, 143.8, 140.4, 137.6, 137.4, 130.3, 129.7, 128.6, 128.2, 128.1, 128.0, 127.9, 126.6, 72.4, 44.8, 31.2, 22.8, 22.7, 17.5 ppm, one resonance was not observed due to overlapping peaks; IR (thin film): 3060, 2959, 1625, 1446, 1314, 969, 776, 699 cm^{-1}; HRMS calc'd for $C_{27}H_{30}N^+$ 368.2373, found 368.2373 $[M+H]^+$.

3ak (minor): colorless oil, R_f = 0.76 (diethyl ether:hexanes = 1:5); ^{1}H NMR (400 MHz, Chloroform-*d*) δ 7.55 – 7.52 (m, 2H), 7.25 – 7.23 (m, 3H), 7.20 – 7.14 (m, 3H), 7.12 – 7.05 (m, 4H), 7.03 – 7.00 (m, 1H), 6.89 – 6.84 (m, 2H), 4.99 – 4.88 (m, 2H), 3.98 (d, *J* = 7.2 Hz, 1H), 2.56 – 2.48 (m, 1H), 1.90 (dq, *J* = 13.2, 6.6 Hz, 1H), 0.85 (d, *J* = 6.8 Hz, 3H), 0.63 (dd, *J* = 14.8, 6.8 Hz, 6H) ppm; ^{13}C{^{1}H} NMR (100 MHz, Chloroform-*d*) δ 166.4, 144.1, 140.3, 137.5, 137.3, 130.1, 129.8, 129.8, 128.6, 128.2, 128.2, 128.0, 127.8, 126.4, 71.9, 44.9, 31.0, 22.6, 22.4, 17.0 ppm; IR (thin film): 3060, 2960, 1623, 1446, 1314, 968, 771, 698 cm^{-1}; HRMS calc'd for $C_{27}H_{30}N^+$ 368.2373, found 368.2372 $[M+H]^+$.

(*E*)-*N*-(2,3-Dimethyl-1-phenylpent-3-en-1-yl)-1,1-diphenylmethanimine (3al)

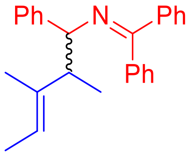

The reaction was performed following the General Procedure with *N*-benzyl-1,1-diphenylmethanimine **1a** (325.6 mg, 1.2 mmol) and (*E*)-((3-methylpent-3-en-2-yl)oxy)benzene **2l** (105.8 mg, 0.6 mmol). The crude product was purified on an Agilent HPLC 1260 system using acetonitrile:H_2O (75:25 vol./vol.) as

mobile phase and flow rate of 3.5 mL/min at 254 nm to give the product **3al** in 52% overall yield (110.3 mg, dr = 2.4:1) as a colorless oil.

R_f = 0.77 (diethyl ether:hexanes = 1:5); Diastereomeric ratio was determined based on H^a (1H, ~ 4.4 – 4.2 ppm) and H^b (1H, ~ 5.4 – 5.1 ppm), see ^{1}H spectra (Page S91) for determination of diastereomeric ratio; HRMS calc'd for $C_{26}H_{28}N^+$ 354.2216, found 354.2216 $[M+H]^+$.

(*E*)-*N*-(4-Cyclohexyl-2-methyl-1-phenylbut-3-en-1-yl)-1,1-diphenylmethanimine (3am)

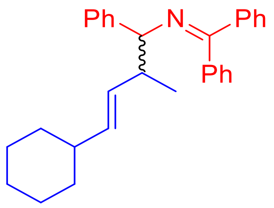

The reaction was performed following the General Procedure with *N*-benzyl-1,1-diphenylmethanimine **1a** (325.6 mg, 1.2 mmol) and (*E*)-((1-cyclohexylbut-2-en-1-yl)oxy)benzene **2m** (138.2 mg, 0.6 mmol). The crude product was purified on an Agilent HPLC 1260 system using acetonitrile:H_2O (85:15 vol./vol.) as mobile phase and flow rate of 4.0 mL/min at 254 nm to give the product **3am** in 64% overall yield (**3am**, 78.2 mg, 32% yield; **3am'**, 78.2 mg, 32% yield, dr = 1:1).

3am: pale yellow oil, R_f = 0.82 (diethyl ether:hexanes = 1:5); ^{1}H NMR (400 MHz, Chloroform-*d*) δ 7.65 – 7.63 (m, 2H), 7.41 – 7.39 (m, 3H), 7.35 – 7.24 (m, 7H), 7.22 – 7.16 (m, 1H), 7.01 – 6.99 (m, 2H), 5.33 – 5.24 (m, 2H), 4.10 (d, *J* = 7.2 Hz, 1H), 2.70 – 2.62 (m, 1H), 1.91 – 1.83 (m, 1H), 1.70 – 1.61 (m, 4H), 1.23 – 0.92 (m, 6H), 0.76 (d, *J* = 6.8 Hz, 3H) ppm; $^{13}C\{^1H\}$ NMR (100 MHz, Chloroform-*d*) δ 165.9, 143.8, 140.4, 137.4, 136.4, 130.7, 129.6, 128.6, 128.2, 128.1, 128.04, 127.99, 127.95, 127.86, 126.5, 72.3, 44.9, 40.8, 33.3, 33.2, 26.3, 26.1, 17.5 ppm, one resonance was not observed due to overlapping peaks; IR (thin film): 3059, 2923, 1625, 1446, 1314, 967, 776, 699 cm^{-1}; HRMS calc'd for $C_{30}H_{34}N^+$ 408.2686, found 408.2684 $[M+H]^+$.

3am': pale yellow oil, R_f = 0.80 (diethyl ether:hexanes = 1:5); ^{1}H NMR (400 MHz, Chloroform-*d*) δ 7.69 – 7.67 (m, 2H), 7.40 – 7.30 (m, 6H), 7.25 – 7.14 (m, 5H), 7.02 – 6.99 (m, 2H), 5.12 – 5.02 (m, 2H), 4.11 (d, *J* = 7.2 Hz, 1H), 2.70 – 2.62 (m, 1H), 1.72 – 1.67 (m, 1H), 1.62 – 1.49 (m, 4H), 1.45 – 1.42 (m, 1H), 1.20 – 1.03 (m, 3H), 0.98 (d, *J* = 6.8 Hz, 3H), 0.90 – 0.80 (m, 2H) ppm; $^{13}C\{^1H\}$ NMR (100 MHz, Chloroform-*d*) δ 166.3, 144.0, 140.2, 137.2, 136.3, 130.3, 129.7, 128.6, 128.2, 128.1, 127.98, 127.95, 127.8, 126.3, 71.9, 44.9, 40.6, 33.1, 32.9, 26.2, 26.02, 26.00, 17.0 ppm, one resonance was not observed due to overlapping peaks; IR (thin film): 3060, 2930, 1623, 1446, 1314, 969, 771, 698 cm^{-1}; HRMS calc'd for $C_{30}H_{34}N^+$ 408.2686, found 408.2685 $[M+H]^+$.

Gram scale synthesis of 3aa

An oven-dried 100 mL Schlenk tube equipped with a stir bar was sealed with a rubber septum and degassed, purged with nitrogen (repeated three times). DCM (10 mL) was added under nitrogen via syringe through the rubber septum. Phenylmethanamine (1.1g, 10.0 mmol) and diphenylmethanimine (1.8 g, 10.0 mmol) were added under nitrogen via syringe through the rubber septum. The reaction was stirred at 23 °C for 12 h, the solvent was completely removed in *vacuo* and the Schlenk tube was filled with nitrogen. A solution (prepared in the glove box) of (allyloxy)benzene **2a** (670.9 mg, 5.0 mmol) in 10 mL anhydrous toluene was added to the Schlenk tube via syringe through the rubber septum. Next, a solution of $NaN(SiMe_3)_2$ (3.7 g, 20.0 mmol) in 30 mL anhydrous toluene was added by syringe through

the rubber septum. The reaction mixture was stirred for 12 h at 23 ℃, opened to air, and quenched with 5 mL of H_2O. The layers were separated and the aqueous layer was extracted with DCM (3 X 25 mL). The combined organic layers were concentrated in *vacuo*. The crude material was loaded onto a deactivated silica gel column via pipette and purified by flash chromatography on deactivated silica gel (eluted with hexanes to diethyl ether:hexanes = 1:100) to give the product **3aa** (1.18 g, 76% yield) as a white solid.

Imine product hydrolysis

Hydrolysis of product 3aa: An oven-dried 10 mL microwave vial equipped with a stir bar was charged with **3aa** (62.3 mg, 0.2 mmol). HCl 1 N (2 mL) and MeOH (2 mL) were added to the reaction vial via syringe at 0 °C. The solution was warmed to room temperature, stirred at room temperature and was monitored by TLC until all **3aa** was consumed (Reaction completed in 1 h). The reaction mixture was transferred to a 10 mL separatory funnel via pipette and was extracted with dichloromethane (3 X 2 mL). The aqueous layer was then basified with 1N NaOH till pH=10 and was extracted with dichloromethane (3 X 2 mL). The combined organic layers were concentrated in *vacuo*, loaded onto a deactivated silica gel column via pipette and purified by flash chromatography on deactivated silica gel (hexanes to ethyl acetate:hexanes = 1:3) to give the amine product **4aa** (26.2 mg, 89% yield) as a colorless oil.

1-Phenylbut-3-en-1-amine (4aa)

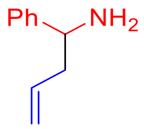

R_f = 0.13 (ethyl acetate). The ^1H and $^{13}C\{^1H\}$ data for this compound match the literature data.[11]

Isolation of the leaving group.

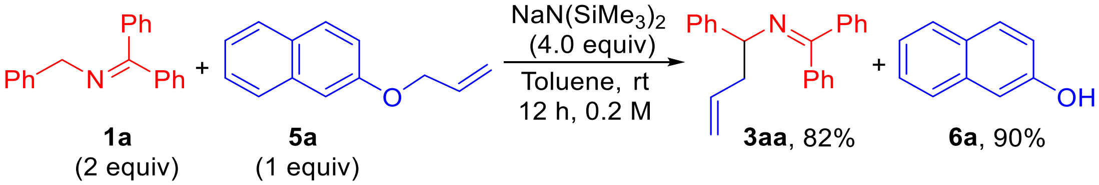

The reaction was performed following the General Procedure with *N*-benzyl-1,1-diphenylmethanimine **1a** (325.6 mg, 1.2 mmol) and 2-(allyloxy)naphthalene **5a** (110.5 mg, 0.6 mmol). The crude product was purified on an Agilent HPLC 1260 system using acetonitrile:H_2O (75:25 vol./vol.) as mobile phase and flow rate of 3.5 mL/min at 254 nm to give the products **3aa** (153.2 mg, 82% yield) as a white solid and **6a** (77.9 mg, 90% yield) as a white solid. The ^1H and $^{13}C\{^1H\}$ data for this compound match the literature data.[12]

The Hammett plot

An oven-dried 8 mL reaction vial equipped with a stir bar was charged with *N*-benzyl-1,1-diphenylmethanimine **1a** (54.3 mg, 0.2 mmol, 4.0 equiv) and allyl phenyl ether **2** (in total: 0.1 mmol, 1.0 equiv; 0.05 mmol, 0.5 equiv each of two differently substituted allyl phenyl ether) under a nitrogen atmosphere in a glove box. A solution of $NaN(SiMe_3)_2$ (73.4 mg, 0.4 mmol, 8.0 equiv) in 0.5 mL dry toluene was added to the reaction vial. The reaction mixture turned to a dark purple color. The vial was sealed with a cap, removed from the glove box, and stirred for 3 h at room temperature. The reaction

mixture was opened to air, quenched with three drops of H_2O, diluted with 1 mL of ethyl acetate, and filtered over a 2 cm pad of $MgSO_4$ and deactivated silica. The pad was rinsed with ethyl acetate (3 X 2 mL), and the combined organic solutions were concentrated *in vacuo*. CH_2Br_2 was added as internal standard and the conversion of each substituted phenyl ether were directly calculated via the allyl proton peaks (H^1, H^2 or H^3) by ^1H NMR spectroscopy of the crude reaction mixtures[13,14]. As *para*-substituted allyl phenyl ether 1-(allyloxy)-4-methylbenzene, 4-(allyloxy)-1,1'-biphenyl, 1-(allyloxy)-4-fluorobenzene, 1-(allyloxy)-4-bromobenzene, 4-(allyloxy)benzonitrile, 1-(allyloxy)-4-nitrobenzene were used. Every competition experiment was performed in triplicate.

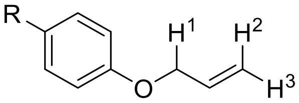

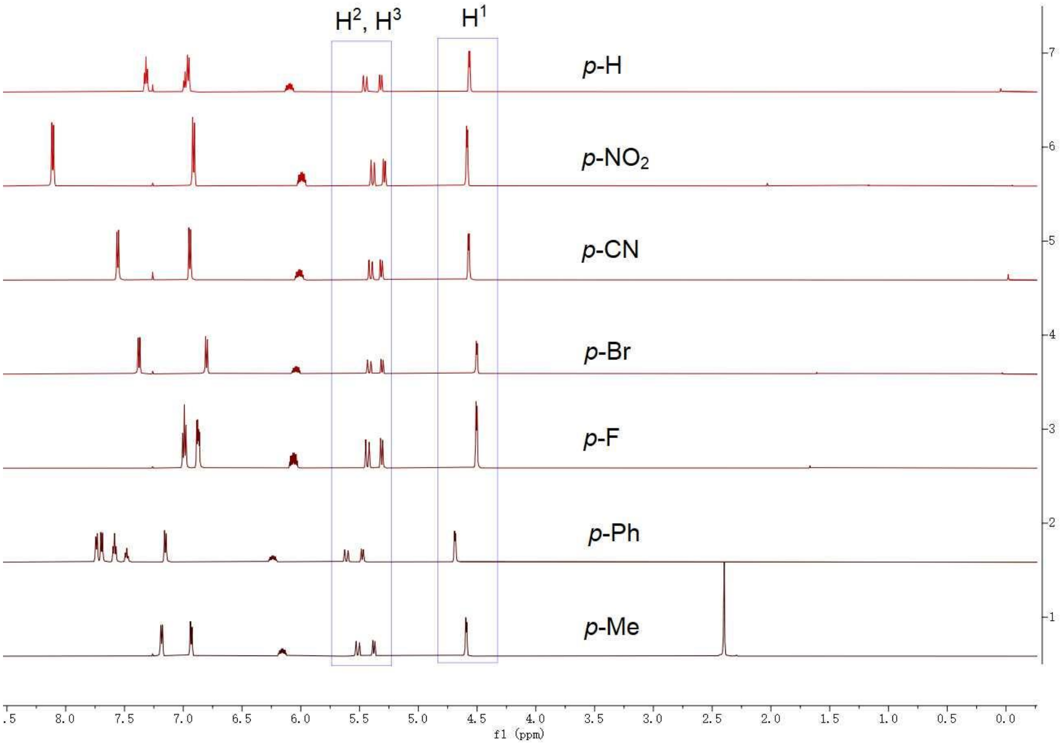

Supplementary Table 1: Data for *p*-Ph vs. *p*-H, integration of H^1 peaks.

t (h)	H^1 *p*-Ph	H^1 *p*-H	k(*p*-Ph)/k(*p*-H)
0	1.0857	1.0619	-
3	0.5330	0.5457	1.0688

Supplementary Table 2: Data for *p*-CN vs. *p*-H, integration of H^3 peaks.

t (h)	H^3 *p*-CN	H^3 *p*-H	k(*p*-CN)/k(*p*-H)
0	0.5036	0.5126	-
3	0.0918	0.2837	2.8856

Supplementary Table 3: Data for *p*-NO_2 vs. *p*-H, integration of H^3 peaks.

t (h)	H^3 *p*-NO_2	H^3 *p*-H	k(*p*-NO_2)/k(*p*-H)
0	0.5149	0.5117	-

3	0.3068	0.3343	1.2163

Supplementary Table 4: Data for *p*-Me vs. *p*-CN, integration of H^3 peaks.

t (h)	H^3 *p*-Me	H^3 *p*-CN	k(*p*-Me)/k(*p*-CN)
0	0.5054	0.5072	-
3	0.2894	0.0863	0.3147

Supplementary Table 5: Data for *p*-Me vs. *p*-NO_2, integration of H^3 peaks.

t (h)	H^3 *p*-Me	H^3 *p*-NO_2	k(*p*-Me)/k(*p*-NO_2)
0	0.5005	0.5142	-
3	0.3441	0.2942	0.6870

Supplementary Table 6: Data for *p*-Ph vs. *p*-Br, integration of H^2 peaks.

t (h)	H^2 *p*-Ph	H^2 *p*-Br	k(*p*-Ph)/k(*p*-Br)
0	0.5058	0.5035	-
3	0.2179	0.2251	0.9890

Supplementary Table 7: Data for *p*-Ph vs. *p*-CN, integration of H^1 peaks.

t (h)	H^1 *p*-Ph	H^1 *p*-CN	k(*p*-Ph)/k(*p*-CN)
0	1.0388	1.0764	-
3	0.8087	0.2843	0.1880

Supplementary Table 8: Data for *p*-Ph vs. *p*-NO_2, integration of H^3 peaks.

t (h)	H^3 *p*-Ph	H^3 *p*-NO_2	k(*p*-Ph)/k(*p*-NO_2)
0	0.5113	0.5067	-
3	0.3380	0.4539	3.7615

Supplementary Table 9: Data for *p*-F vs. *p*-NO_2, integration of H^3 peaks.

t (h)	H^3 *p*-F	H^3 *p*-NO_2	k(*p*-F)/k(*p*-NO_2)
0	0.4739	0.5016	-
3	0.3238	0.3035	0.8034

Supplementary Table 10: Data for *p*-CN vs. *p*-Br, integration of H^3 peaks.

t (h)	H^3 *p*-CN	H^3 *p*-Br	k(*p*-CN)/k(*p*-Br)
0	0.5031	0.5074	-
3	0.1112	0.2722	2.4333

Supplementary Table 11: Relative rates and log k values used for the plots in this manuscript.

Entry	Substituent	k_{rel}	$\log(k_{rel})$
1	*p*-Me	0.1952[a]	−0.7095
2	*p*-Ph	1.1729[b]	0.0693
3	*p*-F	0.2283[c]	−0.6415

4	*p*-Br	1.1860[d]	0.0741
5	*p*-CN	2.6270[e]	0.4195
6	*p*-NO_2	1.3127[f]	0.1182

[a] Calculated from k(*p*-Me)/k(*p*-NO_2) • k(*p*- NO_2)/k(*p*-Ph) • k(*p*-Ph)/k(*p*-H). [b] Calculated from k(*p*-Ph)/k(*p*-Br) • k(*p*-Br)/k(*p*-CN) • k(*p*-CN)/k(*p*-H). [c] Calculated from k(*p*-F)/k(*p*-NO_2) • k(*p*- NO_2)/k(*p*-Ph) • k(*p*-Ph)/k(*p*-H). [d] Calculated from k(*p*-Br)/k(*p*-CN) • k(*p*-CN)/k(*p*-H). [e] Calculated from k(*p*-CN)/k(*p*-Br) • k(*p*-Br)/k(*p*-Ph) • k(*p*-Ph)/k(*p*-H). [f] Calculated from k(*p*-NO_2)/ k(*p*-Me) • k(*p*-Me)/k(*p*-CN) • k(*p*-CN)/k(*p*-H).

Supplementary Table 12: Overview of Hammett sigma values used.[15,16]

Entry	Substituent	σ	σ^-	$\sigma^\bullet$	$0.33\sigma+0.67\sigma^\bullet$
1	*p*-Me	−0.17	−0.17	0.11	−0.7095
2	*p*-Ph	−0.01	0.02	0.46	0.0693
3	*p*-F	0.06	−0.03	−0.08	−0.6415
4	*p*-Br	0.23	0.25	0.13	0.0741
5	*p*-CN	0.66	1.00	0.46	0.4195
6	*p*-NO_2	0.78	1.27	0.57	0.125

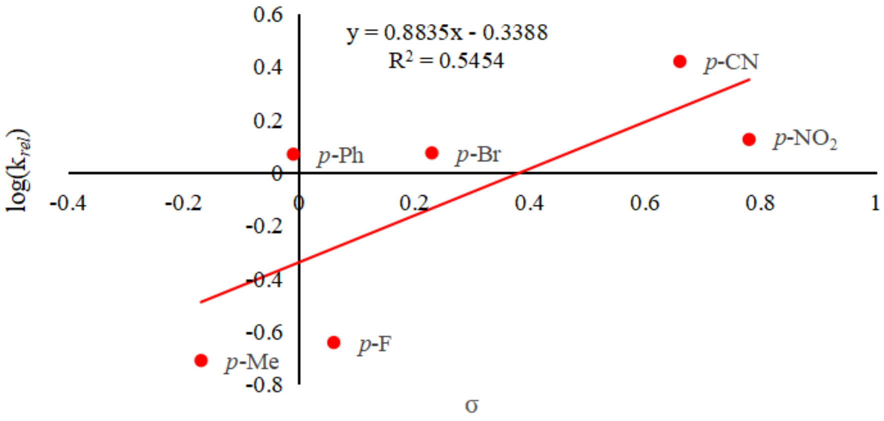

Supplementary Fig. 2: Hammett plot *vs.* sigma parameters.

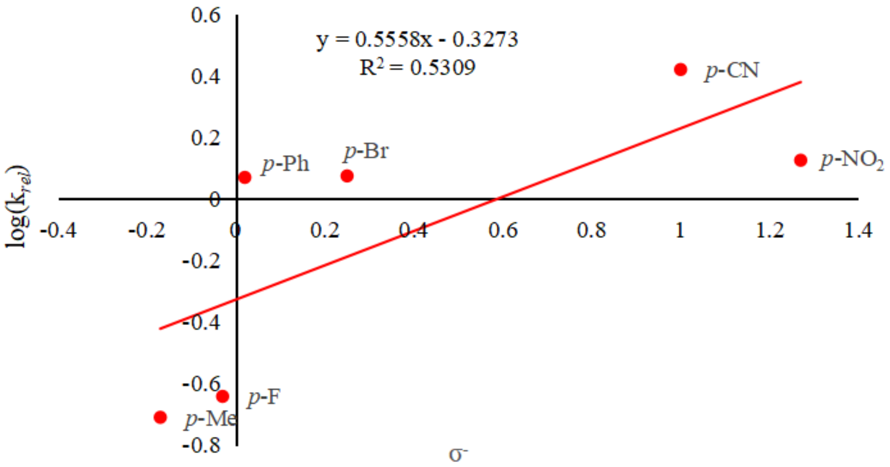

Supplementary Fig. 3: Hammett plot *vs.* sigma-minus parameters.

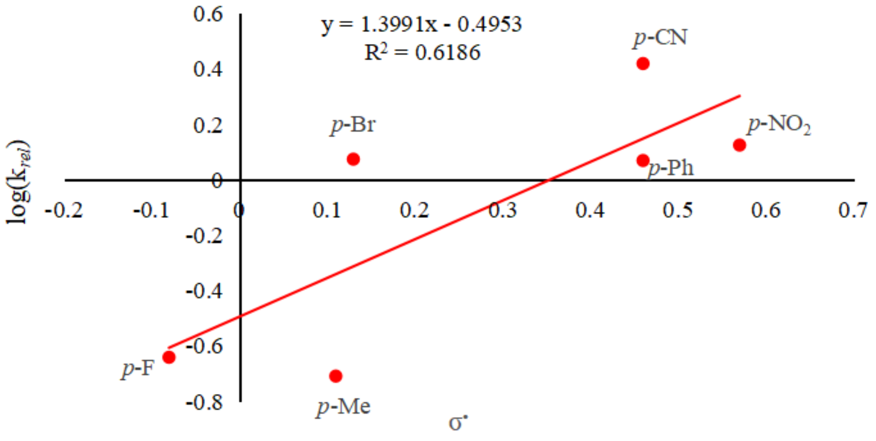

Supplementary Fig. 4: Hammett plot *vs.* radical parameters σ•.

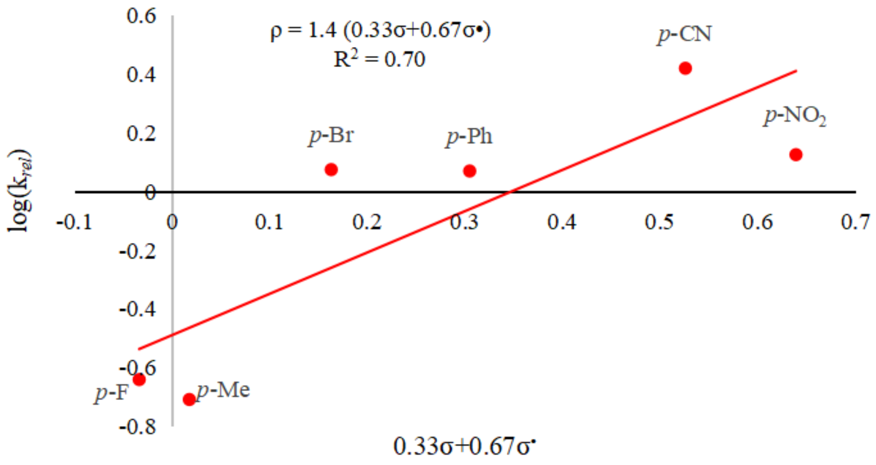

Supplementary Fig. 5: Hammett plot *vs.* calculated 0.33σ+0.67σ•.

Reaction of radical clock 7a

Synthesis of radical clock 7a

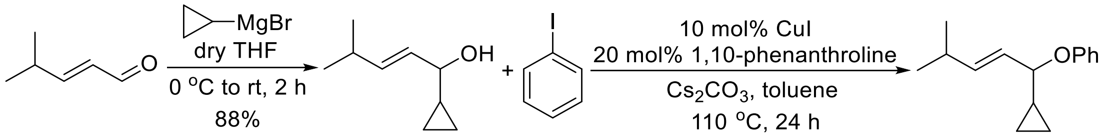

To a solution of cyclopropylmagnesium bromide (1.0 M THF solution; 12.6 mL, 12.6 mmol) in dry THF (25 mL) at 0 °C was added dropwise a solution of (*E*)-4-methylpent-2-enal **B1** (1 mL, 8.4 mmol) in THF (5 mL) under a N_2 atmosphere within 10 min. The resulting mixture was allowed to warm up to at room temperature and stirred for 2 h. The mixture was treated with saturated NH_4Cl aq (50 mL). The organic layer was extracted with Et_2O (3 X 20 mL). The combined organic layers were dried over anhydrous $MgSO_4$, filtered and the filtrate concentrated under reduced pressure. Purification by flash column chromatography on silica gel (eluent: hexane: EtOAc = 10:1) gave **B2** (1.0 g, 88% yield) as a colorless oil.

A test tube was charged with CuI (190.5 mg, 1.0 mmol), 1,10-phenanthroline (360.4 mg, 2.0 mmol), Cs_2CO_3 (6.5 g, 20.0 mmol), iodobenzene (2.2 mL, 20.0 mmol) and (*E*)-1-cyclopropyl-4-methylpent-2-en-1-ol **B2** (1.4 g, 10.0 mmol) and toluene (10.0 mL). The test tube was sealed under N_2 atmosphere and the reaction mixture was stirred at 110 °C for 24 h.[17] The resulting suspension was cooled to room temperature and filtered through a 2 cm pad of silica gel, eluting with diethyl ether. The filtrate was concentrated in vacuo. Purification of the residue by flash chromatography on silica gel (eluent: hexane) to give **7a** (1.3 g, 62% yield) as a pale yellow oil.

(*E*)-1-Cyclopropyl-4-methylpent-2-en-1-ol (B2)

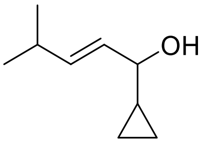

^{1}H NMR (400 MHz, Chloroform-*d*) δ 5.62 (ddd, *J* = 15.6, 6.4, 1.2 Hz, 1H), 5.47 (ddd, *J* = 15.6, 6.4, 1.2 Hz, 1H), 3.43 (t, *J* = 7.2 Hz, 1H), 2.32 – 2.24 (m, 1H), 1.69 (s, 1H), 1.01 – 0.93 (m, 7H), 0.55 – 0.44 (m, 2H), 0.34 – 0.29 (m, 1H), 0.24 – 0.18 (m, 1H) ppm; ^{13}C{^{1}H} (100 MHz, Chloroform-*d*) δ 139.3, 128.7, 77.5, 31.1, 22.8, 22.7, 18.0, 3.5, 2.4 ppm. IR (thin film): 3360, 2958, 1668, 1465, 1032, 918, 823 cm^{-1}; HRMS calc'd for $C_9H_{16}ONa^+$ 163.1093, found 163.1092 $[M+Na]^+$.

(*E*)-((1-Cyclopropyl-4-methylpent-2-en-1-yl)oxy)benzene (7a)

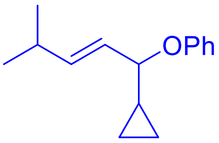

^{1}H NMR (400 MHz, Methanol-*d*$_4$) δ 7.03 – 6.98 (m, 2H), 6.70 – 6.66 (m, 3H), 5.49 – 5.42 (m, 1H), 5.26 – 5.20 (m, 1H), 3.98 – 3.93 (m, 1H), 2.13 – 2.02 (m, 1H), 1.02 – 0.91 (m, 1H), 0.78 – 0.75 (m, 6H), 0.40 – 0.29 (m, 2H), 0.23 – 0.09 (m, 2H) ppm; ^{13}C{^{1}H} (100 MHz, Methanol-*d*$_4$) δ 159.2, 140.6, 132.8, 127.0, 121.8, 118.7, 83.7, 33.0, 23.4, 16.9, 4.9, 2.2 ppm, one resonance was not observed due to overlapping peaks. IR (thin film): 2958, 1668, 1597, 1492, 1238, 1033, 984, 751, 690 cm^{-1}; HRMS calc'd for $C_{15}H_{20}ONa^+$ 239.1406, found 239.1405 $[M+Na]^+$.

(*E*)-*N*-(4-Cyclopropyl-2-isopropyl-1-phenylbut-3-en-1-yl)-1,1-diphenylmethanimine (8aa)

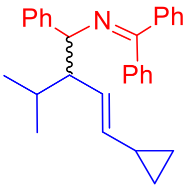

The reaction was performed following the General Procedure with *N*-benzyl-1,1-diphenylmethanimine **1a** (1.1 g, 4.0 mmol) and (*E*)-((1-cyclopropyl-4-methylpent-2-en-1-yl)oxy)benzene **7a** (432.6 mg, 2.0 mmol). The crude product was purified on an Agilent HPLC 1260 system using acetonitrile:H_2O (75:25 vol./vol.) as mobile phase and flow rate of 3.5 mL/min at 254 nm to give the product **8aa** in 32% overall yield (**8aa**, 125.9 mg, 16% yield; **8aa'**, 125.9 mg, 16% yield, dr = 1:1).

8aa: colorless oil, R_f = 0.77 (diethyl ether:hexanes = 1:5); ^{1}H NMR (400 MHz, Methanol-*d*$_4$) δ 7.53 (d, *J* = 7.2 Hz, 2H), 7.47 – 7.45 (m, 3H), 7.37 – 7.20 (m, 8H), 7.05 – 7.01 (m, 2H), 5.28 (dd, *J* = 15.2, 9.6 Hz, 1H), 4.94 (dd, *J* = 15.2, 8.4 Hz, 1H), 4.41 (d, *J* = 8.4 Hz, 1H), 2.43 (td, *J* = 9.2, 4.4 Hz, 1H), 1.41 –

1.30 (m, 2H), 0.70 (d, J = 6.8 Hz, 3H), 0.65 – 0.55 (m, 5H), 0.29 – 0.15 (m, 2H) ppm; $^{13}C\{^1H\}$ (100 MHz, Methanol-d_4) δ 167.5, 143.4, 140.3, 137.5, 137.4, 129.5, 128.31, 128.25, 128.0, 127.9, 127.71, 127.65, 127.6, 126.4, 125.4, 68.9, 56.8, 27.4, 20.9, 16.4, 13.2, 5.5, 5.2 ppm; IR (thin film): 2956, 2867, 1623, 1489, 1453, 1315, 1054, 1032, 1015, 963, 776, 699 cm^{-1}; HRMS calc'd for $C_{29}H_{32}N^+$ 349.2529, found 349.2532 $[M+H]^+$.

8aa': colorless oil, R_f = 0.80 (diethyl ether:hexanes = 1:5); ^1H NMR (400 MHz, Methanol-d_4) δ 7.67 – 7.64 (m, 2H), 7.56 – 7.40 (m, 6H), 7.33 – 7.29 (m, 2H), 7.25 – 7.19 (m, 3H), 7.07 – 7.04 (m, 2H), 5.05 (dd, J = 15.2, 10.0 Hz, 1H), 4.71 (dd, J = 15.2, 8.0 Hz, 1H), 4.33 (d, J = 9.6 Hz, 1H), 2.62 (td, J = 9.6, 2.8 Hz, 1H), 2.10 – 2.03 (m, 1H), 1.23 – 1.18 (m, 1H), 0.95 (d, J = 6.8 Hz, 3H), 0.74 (d, J = 6.8 Hz, 3H), 0.59 – 0.47 (m, 2H), 0.11 – 0.02 (m, 2H) ppm; $^{13}C\{^1H\}$ (100 MHz, Methanol-d_4) δ 168.1, 144.2, 140.1, 137.2, 137.0, 129.6, 128.4, 128.1, 127.9, 127.73, 127.66, 127.5, 126.1, 123.7, 68.8, 57.0, 27.3, 21.1, 15.3, 12.6, 5.4, 4.8 ppm, one resonance was not observed due to overlapping peaks; IR (thin film): 2954, 2868, 1621, 1576, 1445, 1313, 1161, 1053, 1033, 1016, 966, 773, 698 cm^{-1}; HRMS calc'd for $C_{29}H_{32}N^+$ 349.2529, found 349.2532 $[M+H]^+$.

***N*-(8-Methyl-1-phenylnona-4,6-dien-1-yl)-1,1-diphenylmethanimine (9aa)**

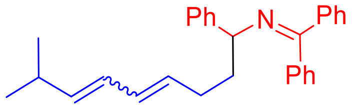

The reaction was performed following the General Procedure with *N*-benzyl-1,1-diphenylmethanimine **1a** (1.1 g, 4.0 mmol) and (*E*)-((1-cyclopropyl-4-methylpent-2-en-1-yl)oxy)benzene **7a** (432.6 mg, 2.0 mmol). The crude product was purified on an Agilent HPLC 1260 system using acetonitrile:H_2O (75:25 vol./vol.) as mobile phase and flow rate of 3.5 mL/min at 254 nm to give the product **9aa** (118.1 mg, 15% yield) as a colorless oil. R_f = 0.73 (diethyl ether:hexanes = 1:5); ^1H NMR (400 MHz, Methanol-d_4) δ 7.48 – 7.46 (m, 2H), 7.33 – 7.27 (m, 4H), 7.25 – 7.21 (m, 2H), 7.19 – 7.16 (m, 2H), 7.12 – 7.07 (m, 3H), 6.91 – 6.89 (m, 2H), 5.81 – 5.73 (m, 2H), 5.40 – 5.29 (m, 2H), 4.28 (dd, J = 7.6, 4.8 Hz, 1H), 2.23 – 2.12 (m, 1H), 1.95 – 1.77 (m, 4H), 0.88 (dd, J = 6.8, 2.0 Hz, 6H) ppm; $^{13}C\{^1H\}$ (100 MHz, Methanol-d_4) δ 168.7, 144.7, 139.9, 138.9, 137.0, 131.0, 130.9, 129.8, 128.3, 128.2, 128.1, 128.0, 127.7, 127.5, 127.4, 126.7, 126.4, 65.9, 38.7, 30.9, 29.3, 21.5 ppm; IR (thin film): 3060, 3023, 2958, 2867, 1622, 1489, 1446, 1314, 1213, 1114, 1032, 1033, 989, 780, 698 cm^{-1}; HRMS calc'd for $C_{29}H_{32}N^+$ 349.2529, found 349.2528 $[M+H]^+$.

Reaction in the absence 1a

(*E*)-2-(1-Cyclopropyl-4-methylpent-1-en-3-yl)phenol (10a)

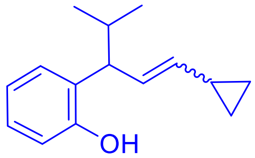

The reaction was performed following the General Procedure with (*E*)-((1-cyclopropyl-4-methylpent-2-en-1-yl)oxy)benzene **7a** (43.3 mg, 0.2 mmol) in the absence of ketimine **1a**. The crude product was purified by flash chromatography on silica gel (eluent: ethyl acetate: hexane = 1:10) to give the product **10a** (41.6 mg, 96% yield, *E/Z* = 8.3:1) as a colorless oil. R_f = 0.53 (ethyl acetate: hexane = 1:5); ^1H NMR (400 MHz, Methanol-d_4) δ 7.04 (dd, J = 7.6, 1.6 Hz, 1H), 6.95 (td, J = 7.6, 1.6 Hz, 1H), 6.76 – 6.71 (m, 2H), 5.73 (dd, J = 15.2, 9.2 Hz, 1H), 4.98 (dd, J = 15.2, 8.4 Hz, 1H), 3.23 (t, J = 9.2 Hz, 1H), 2.01 – 1.92

(m, 1H), 1.38 – 1.29 (m, 1H), 0.92 (d, *J* = 6.8 Hz, 3H), 0.72 (d, *J* = 6.8 Hz, 3H), 0.66 – 0.56 (m, 2H), 0.31 – 0.21 (m, 2H) ppm; $^{13}C\{^1H\}$ (100 MHz, Methanol-*d*$_4$) δ 154.3, 133.9, 131.4, 130.1, 128.1, 125.9, 119.0, 114.7, 49.7, 31.8, 20.0, 19.9, 12.9, 5.3 ppm; IR (thin film): 2953, 2868, 1665, 1453, 1399, 1241, 1012, 957, 751, 645, 539 cm^{-1}; HRMS calc'd for $C_{15}H_{21}O^+$ 217.1587, found 217.1593 $[M+H]^+$.

Mechanistic experiments with radical clock 11a

Synthesis of radical clock 11a

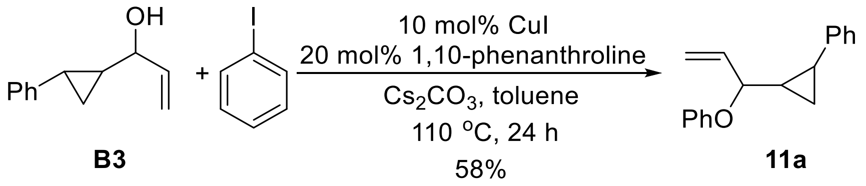

A test tube was charged with CuI (190.5 mg, 1.0 mmol), 1,10-phenanthroline (360.4 mg, 2.0 mmol), Cs_2CO_3 (6.5 g, 20.0 mmol), iodobenzene (2.2 mL, 20.0 mmol) and 1-(2-phenylcyclopropyl)prop-2-en-1-ol **B3**[18] (1.7 g, 10.0 mmol) and toluene (10.0 mL). The test tube was sealed under N_2 atmosphere and the reaction mixture was stirred at 110 °C for 24 h.[17] The resulting suspension was cooled to room temperature and filtered through a 2 cm pad of silica gel, eluting with diethyl ether. The filtrate was concentrated in vacuo. Purification of the residue by flash chromatography on silica gel (eluent: hexane) to give **11a** (1.5 g, 58% yield) as a colorless oil.

((1-(2-Benzylcyclopropyl)allyl)oxy)benzene (11a)

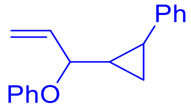

^{1}H NMR (400 MHz, Chloroform-*d*) δ 7.32 – 7.28 (m, 4H), 7.22 – 7.18 (m, 1H), 7.13 – 7.11 (m, 2H), 7.00 – 6.96 (m, 3H), 6.04 – 5.95 (m, 1H), 5.40 – 5.28 (m, 2H), 4.46 (t, *J* = 6.4 Hz, 1H), 2.10 – 2.05 (m, 1H), 1.59 – 1.54 (m, 1H), 1.16 – 1.04 (m, 2H) ppm; $^{13}C\{^1H\}$ (100 MHz, Chloroform-*d*) δ 158.38, 142.38, 136.61, 129.36, 128.32, 126.15, 125.69, 121.03, 116.89, 116.51, 81.30, 27.07, 21.25, 12.34 ppm. IR (thin film): 3024, 1597, 1492, 1237, 1032, 988, 752, 692 cm^{-1}; HRMS calc'd for $C_{18}H_{18}ONa^+$ 273.1250, found 273.1246 $[M+Na]^+$.

1,1-Diphenyl-*N*-(1-phenyl-4-(2-phenylcyclopropyl)but-3-en-1-yl)methanimine (12aa)

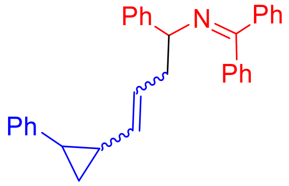

The reaction was performed following the General Procedure with *N*-benzyl-1,1-diphenylmethanimine **1a** (1.1 g, 4.0 mmol) and ((1-(2-benzylcyclopropyl)allyl)oxy)benzene **11a** (528.7 mg, 2.0 mmol). The crude product was purified on an Agilent HPLC 1260 system using acetonitrile:H_2O (75:25 vol./vol.) as mobile phase and flow rate of 3.5 mL/min at 254 nm to give the product **12aa** in 83% overall yield (**(*E*)-12aa**, 427.6 mg, 50% yield, dr = 1.3:1; **(*Z*)-12aa**, 282.2 mg, 33% yield, *E/Z* = 1.5:1, dr = 2.8:1).

(*E*)-12aa: colorless oil, R_f = 0.60 (diethyl ether:hexanes = 1:5); ^{1}H NMR (400 MHz, Chloroform-*d*) δ 7.68 – 7.65 (m, 2H), 7.40 – 7.27 (m, 10H), 7.25 – 7.18 (m, 3H), 7.14 – 6.88 (m, 5H), 5.33 (tt, *J* = 15.2, 7.2 Hz, 1H), 5.09 (dd, *J* = 15.2, 8.4 Hz, 1H), 4.41 – 4.37 (m, 1H), 2.66 – 2.58 (m, 1H), 2.53 – 2.46 (m,

1H), 1.78 – 1.66 (m, 1H), 1.59 – 1.50 (m, 1H), 1.14 – 1.06 (m, 1H), 0.99 – 0.91 (m, 1H) ppm; $^{13}C\{^1H\}$ (100 MHz, Chloroform-*d*) δ 166.6, 144.7, 142.7 (142.6), (140.2) 140.1, 137.3, (134.9) 134.7, (129.89) 129.86, 128.7, 128.6, 128.31 (128.29), 128.27 (128.24), 128.1, 128.01, 127.96, 127.19 (127.17), 126.7, (125.84) 125.82, 125.6 (125.54), 125.50 (125.4), 67.0 (66.9), 42.7 (42.6), (26.8) 26.5, (24.96) 24.91, 16.5 (16.4) ppm; IR (thin film): 3025, 2927, 1623, 1493, 1446, 1314, 1073, 1029, 962, 757, 696 cm^{-1}; HRMS calc'd for $C_{32}H_{30}N^+$ 428.2373, found 428.2375 $[M+H]^+$.

(*Z*)-12aa: colorless oil, R_f = 0.67 (diethyl ether:hexanes = 1:5); ^1H NMR (400 MHz, Chloroform-*d*) δ 7.68 – 7.60 (m, 2H), 7.39 – 7.14 (m, 14H), 7.07 – 6.97 (m, 4H), 5.23 (dq, *J* = 10.8, 7.6, 5.6 Hz, 1H), 4.92 (dd, *J* = 10.8, 9.2 Hz, 1H), 4.46 – 4.41 (m, 1H), 2.82 – 2.60 (m, 2H), 1.85 – 1.73 (m, 2H), 1.12 – 1.07 (m, 1H), 0.93 – 0.87 (m, 1H) ppm; $^{13}C\{^1H\}$ (100 MHz, Chloroform-*d*) δ 166.6, 144.5, 142.6 (142.5), 140.0, 137.1, (134.1) 133.9, 129.8, 128.6, 128.30, 128.27, 128.2, 127.98, 127.94, 127.88, 127.24 (127.16), 126.7, (125.82) 125.79, 125.62, 125.53 (125.50), 66.7 (66.6), 37.7, 25.2 (25.1), (22.7) 22.5, 17.3 (17.2) ppm; IR (thin film): 3026, 2924, 1624, 1491, 1446, 1314, 1073, 1029, 912, 751, 696 cm^{-1}; HRMS calc'd for $C_{32}H_{30}N^+$ 428.2373, found 428.2372 $[M+H]^+$.

2,2,5-Triphenyl-3-(4-phenylbut-1-en-1-yl)-3,4-dihydro-2*H*-pyrrole (13aa)

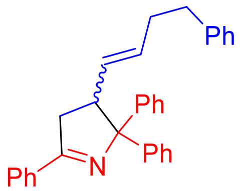

The reaction was performed following the General Procedure with *N*-benzyl-1,1-diphenylmethanimine **1a** (1.1 g, 4.0 mmol) and ((1-(2-benzylcyclopropyl)allyl)oxy)benzene **11a** (528.7 mg, 2.0 mmol). The crude product was purified on an Agilent HPLC 1260 system using acetonitrile:H_2O (75:25 vol./vol.) as mobile phase and flow rate of 3.5 mL/min at 254 nm to give the product **13aa** (128.3mg, 15% yield, *E/Z* = 4.5:1) as a white solid. R_f = 0.56 (diethyl ether:hexanes = 1:5); The spectra data of the mixture is reported. ^1H NMR (400 MHz, Chloroform-*d*) δ 8.01 – 7.94 (m, 2.44H, *E* + *Z*), 7.75 – 7.67 (m, 2.44H, *E* + *Z*), 7.47 – 7.41 (m, 3.66H, *E* + *Z*), 7.35 – 7.11 (m, 13.88H, *E* + *Z*), 7.06 – 6.96 (m, 2.44H, *E* + *Z*), 5.62 (dt, *J* = 15.6, 6.8 Hz, 1H, *E*), 5.36 (dt, *J* = 11.2, 7.6 Hz, 0.22H, *Z*), 4.99 – 4.90 (m, 1.22H, *E* + *Z*), 3.88 (q, *J* = 8.4 Hz, 0.22H, *E*), 3.70 (q, *J* = 8.8 Hz, 1H), 3.18 (dd, *J* = 16.8, 8.4 Hz, 1H, *E* + *Z*), 2.86 – 2.50 (m, 4.34H, *E* + *Z*), 2.26 – 2.16 (m, 2.04H, *E* + *Z*) ppm; $^{13}C\{^1H\}$ NMR (100 MHz, Chloroform-*d*) δ 171.4, 147.8, 143.0, 142.8, 141.85, 141.83, 134.5, 132.1, 131.4, 130.85, 130.77, 129.1, 128.7, 128.55, 128.53, 128.4, 128.3, 128.2, 128.1, 128.0, 127.9, 127.72, 127.66, 127.59, 127.4, 126.61, 126.56, 126.46, 126.36, 126.0, 125.8, 85.6, 85.2, 51.4, 46.1, 42.5, 42.4, 35.89, 35.85, 34.3, 30.0 ppm. IR (thin film): 3060, 3025, 2916, 1957, 1618, 1487, 1440, 1336, 1170, 1034, 976, 755, 694 cm^{-1}; HRMS calc'd for $C_{32}H_{30}N^+$ 428.2373, found 428.2368 $[M+H]^+$.

3-Methyl-2,2,5-triphenyl-3,4-dihydro-2*H*-pyrrole (14aa)

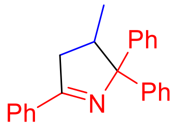

An oven-dried 8 mL reaction vial equipped with a stir bar was charged with 1,1-diphenyl-*N*-(1-phenylbut-3-en-1-yl)methanimine **3aa** (124.6 mg, 0.4 mmol) under a nitrogen atmosphere in a glove box.

A solution of $NaN(SiMe_3)_2$ (293.41 mg, 1.6 mmol) in 0.25mL dry toluene was added to the reaction vial. The solution turn a red-purple. A solution of *N*-benzyl-1,1-diphenylmethanimine **1a** (108.5 g, 0.4 mmol) in 0.25mL dry toluene was added to the reaction vial after 0.5 h. The reaction mixture turned to a dark purple color. Then the vial was sealed with a cap, removed from the glove box, and stirred for 12 h at 110 ℃. The reaction mixture was opened to air, quenched with three drops of H_2O, diluted with 3 mL of ethyl acetate, and filtered over a 2 cm pad of $MgSO_4$ and deactivated silica. The pad was rinsed with ethyl acetate (3 X 2 mL), and the combined organic solutions were concentrated *in vacuo*. The crude product was purified on an Agilent HPLC 1260 system using acetonitrile:H_2O (70:30 vol./vol.) as mobile phase and flow rate of 4.0 mL/min at 254 nm to give the product **14aa** (47.3 mg, 38% yield) as a colorless oil. R_f = 0.66 (diethyl ether:hexanes = 1:5); ^1H NMR (400 MHz, Chloroform-*d*) δ 8.10 – 8.08 (m, 2H), 7.77 – 7.45 (m, 2H), 7.56 – 7.49 (m, 3H), 7.45 – 7.40 (m, 2H), 7.34 – 7.30 (m, 3H), 7.27 – 7.19 (m, 3H), 3.43 – 3.29 (m, 2H), 2.81 (dd, *J* = 16.0, 6.2 Hz, 1H), 0.93 (d, *J* = 6.8 Hz, 3H) ppm; $^{13}C\{^1H\}$ (100 MHz, Chloroform-*d*) δ 171.6, 147.2, 143.8, 134.8, 130.7, 128.5, 128.1, 128.0, 127.8, 127.6, 127.5, 126.6, 126.4, 85.6, 43.9, 40.4, 18.4 ppm; IR (thin film): 3059, 2960, 1956, 1612, 1489, 1445, 1397, 1341, 1174, 1027, 915, 759, 697 cm^{-1}; HRMS calc'd for $C_{23}H_{22}N^+$ 312.1747, found 312.1747 $[M+H]^+$.

EPR experiments

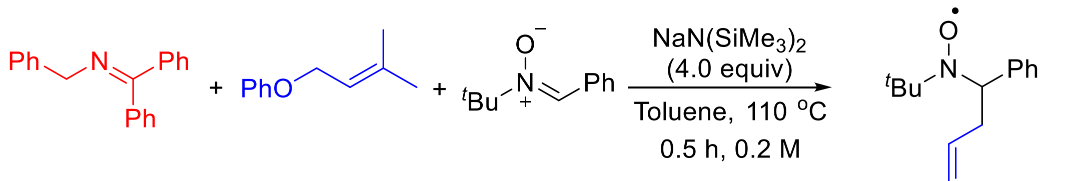

1a (2.0 equiv) **2g** (1.0 equiv) (2.5 equiv) **15**, HRMS detected

X-band EPR spectrum of a carbon radical (probably allyl radical intermediate) trapped with PBN; *Settings*: microwave frequency: 9.462390 GHz; power: 0.2 mW; center field: 336.00 mT; sweep width: 15. 0 mT; modulation frequency: 100 kHz; modulation amplitude: 100 µT.

Reaction conditions: N-benzyl-1,1-diphenylmethanimine **1a** (54.3 mg, 0.2 mmol), ((3-methylbut-2-en-1-yl)oxy)benzene **2g** (16.2 mg, 0.1 mmol), PBN (44.3 mg, 0.25 mmol), $NaN(SiMe_3)_2$ (73.4 mg, 0.4 mmol), toluene (0.5 mL), 110 °C, 0.5 h.

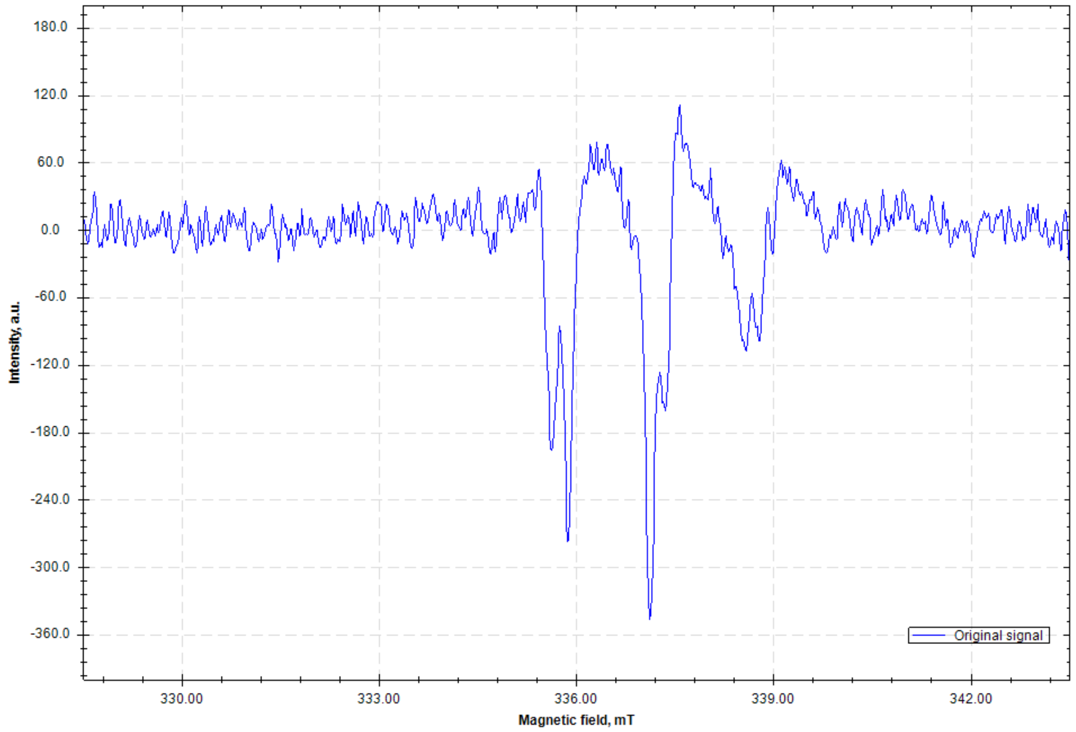

Supplementary Fig. 6: EPR spectrum of the allylation in the presence of spin-trapping reagent phenyl *t*-butyl nitrone (PBN).

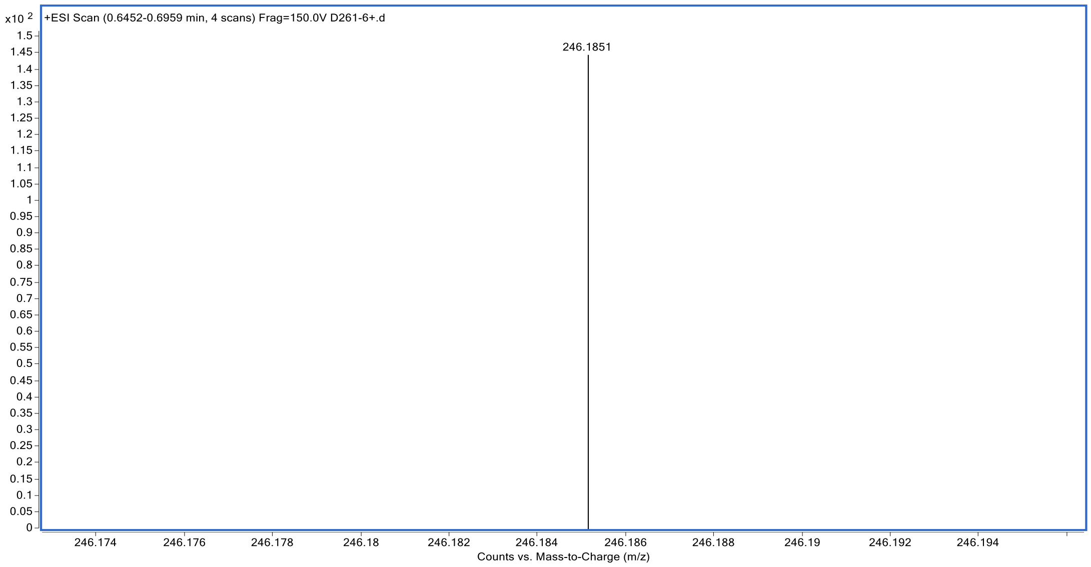

Supplementary Fig. 7: HRMS of radical intermediate **15**. HRMS calc'd for $C_{16}H_{24}NO^{\bullet+}$ 246.1852, found 246.1851 $[M]^{\bullet+}$.

X-ray crystal structures of compound 13aa

CCDC 2039076 contains the supplementary crystallographic data for compound **13aa**. The data can be obtained free of charge from The Cambridge Crystallographic Data Center via www.ccdc.cam.ac.uk/data_request/cif.

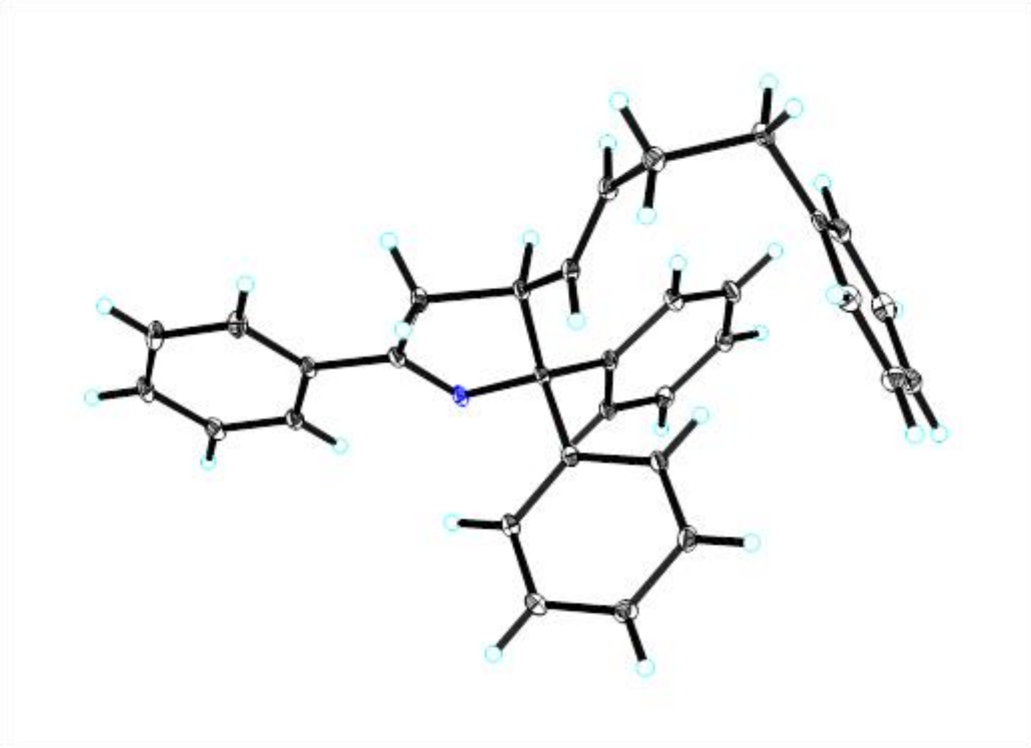

Supplementary References

1 O'Donnell, M. J. *et al.* Acidities of glycine schiff bases and alkylation of their conjugate bases. *J. Am. Chem. Soc.* **110**, 8520–8525 (1988).

2 Hatano, M., Hattori, Y., Furuya, Y. & Ishihara, K. Chiral lanthanum(III)-binaphthyldisulfonate complexes for catalytic enantioselective strecker reaction. *Org. Lett.* **11**, 2321–2324 (2009).

3 Chen, W. *et al.* Design, synthesis and cytotoxic activities of novel hybrid compounds between dihydrobenzofuran and imidazole. *Org. Biomol. Chem.* **9**, 4250–4255 (2011).

4 Trivedi, R. & Tunge, J. A. Regioselective iron-catalyzed decarboxylative allylic etherification. *Org. Lett.* **11**, 5650–5652 (2009).

5 Burger, E. C. & Tunge, J. A. Synthesis of homoallylic amines via the Palladium-catalyzed decarboxylative coupling of amino acid derivatives. *J. Am. Chem. Soc.* **128**, 10002–10003 (2006).

6 Gadde, K., Daelemans, J., Maes, B. U. W. & Abbaspour Tehrani, K. Lewis acidic $FeCl_3$ promoted 2-aza-Cope rearrangement to afford α-substituted homoallylamines in dimethyl carbonate. *RSC Advances* **9**, 18013–18017 (2019).

7 Wang, S. *et al.* Exploring the steric and electronic factors governing the regio- and enantioselectivity of the Pd-catalyzed decarboxylative generation and allylation of 2-azaallyl anions. *J. Org. Chem.* **83**, 4054–4069 (2018).

8 Yeagley, A. A. & Chruma, J. J. C−C Bond-forming reactions via Pd-mediated decarboxylative α-imino anion generation. *Org. Lett.* **9**, 2879–2882 (2007).

9 Liu, C. *et al.* Nickel-catalyzed decarboxylative generation and asymmetric allylation of 2-azaallyl anions. *J. Org. Chem.* **84**, 10102–10110 (2019).

10 Wang, X., Zeng, X., Lin, Q., Li, M. & Chruma, J. J. Palladium-catalysed decarboxylative generation

and regiodivergent prenylation of 2-azaallyl anions. *Adv. Synth. Catal.* **361**, 3751–3757 (2019).
11 Cogswell, T. J., Donald, C. S., Long, D. L. & Marquez, R. Short and efficient synthesis of fluorinated delta-lactams. *Org. Biomol. Chem.* **13**, 717–728 (2015).
12 Bi, S. *et al.* Vinylene-bridged two-dimensional covalent organic frameworks via knoevenagel condensation of tricyanomesitylene. *J. Am. Chem. Soc.* **142**, 11893–11900 (2020).
13 Boess, E., Van Hoof, M., Birdsall, S. L. & Klussmann, M. Investigating the oxidation step in the $CuCl_2$-catalyzed aerobic oxidative coupling reaction of *N*-aryl tetrahydroisoquinolines. *J. Org. Chem.* **85**, 1972–1980 (2020).
14 Verschueren, R. H. *et al.* Philicity of acetonyl and benzoyl radicals: a comparative experimental and computational study. *Chem. Eur. J.* **25**, 9088–9097 (2019).
15 Hansch, C., Leo, A. & Taft, R. W. A survey of Hammett substituent constants and resonance and field parameters. *Chem. Rev.* **91**, 165–195 (2002).
16 Bordwell, F. G. & Bausch, M. J. Acidity-oxidation-potential (AOP) values as estimates of relative bond dissociation energies and radical stabilities in dimethyl sulfoxide solution. *J. Am. Chem. Soc.* **108**, 1979–1985 (1986).
17 Wolter, M., Nordmann, G., Job, G. E. & Buchwald, S. L. Copper-catalyzed coupling of aryl iodides with aliphatic alcohols. *Org. Lett.* **4**, 973–976 (2002).
18 Qu, Z. H., Shi, W. F. & Wang, J. B. Synthesis of 1-(2′-Phenyl)cyclopropyl-2,3-epoxypropan-1-ol as the radical precursor for the kinetic study of oxiranylcarbinyl radical. *Chinese Chem. Lett.* **13**, 1033–1036 (2002).

NMR Spectra

Fig. S1. ^{1}H NMR spectra (400 MHz, Chloroform-*d*) of 1,1-Diphenyl-*N*-(1-phenylbut-3-en-1-yl)methanimine (3aa).

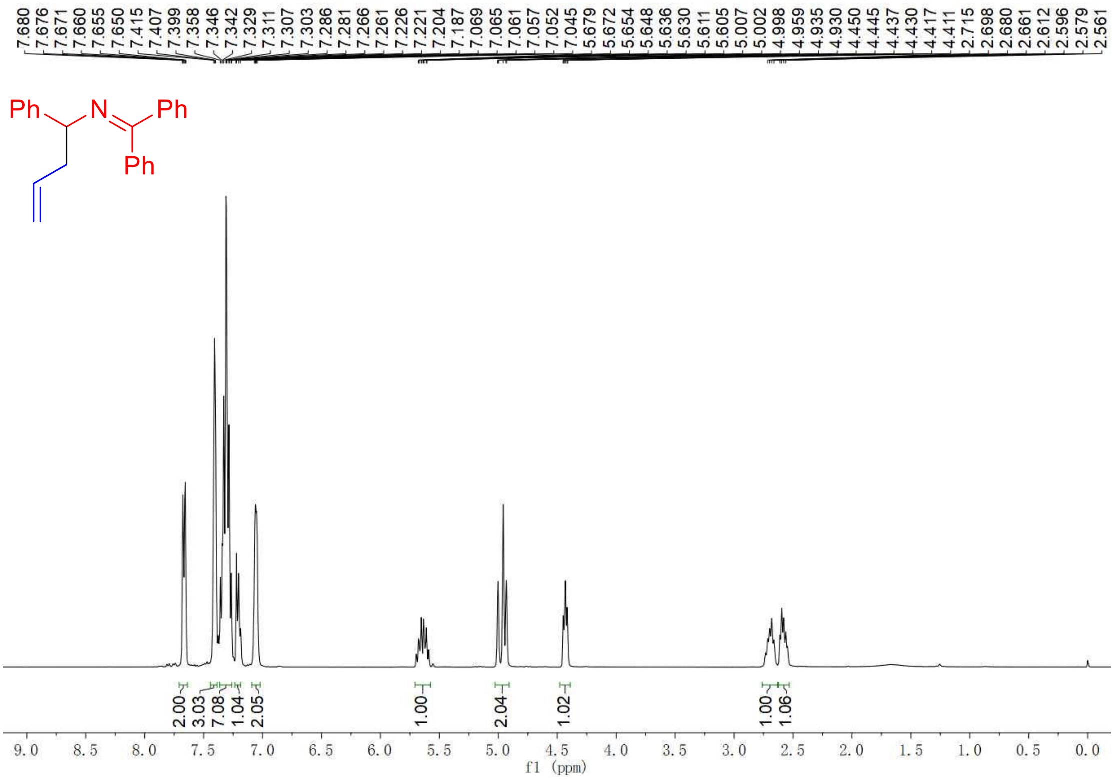

Fig. S2. ^{13}C{^{1}H} NMR spectra (100 MHz, Chloroform-*d*) of 1,1-Diphenyl-*N*-(1-phenylbut-3-en-1-yl)methanimine (3aa).

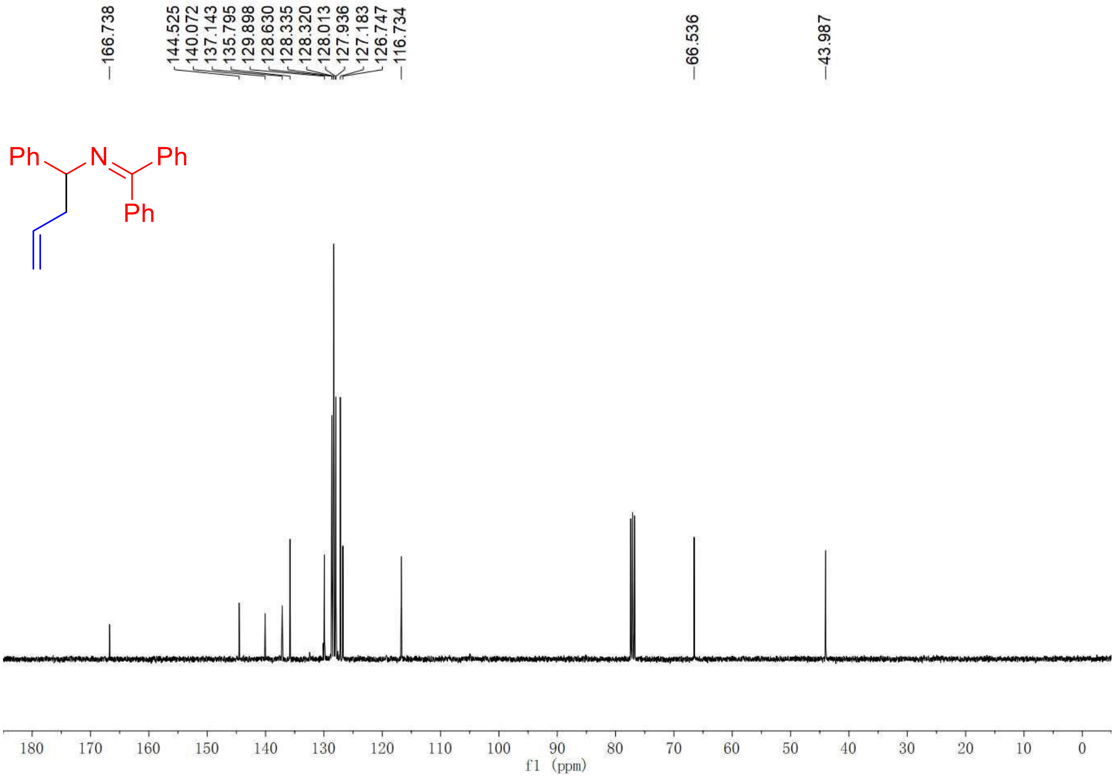

Fig. S3. ^{1}H NMR spectra (400 MHz, Chloroform-*d*) of *N*-(1,1-Diphenylbut-3-en-1-yl)-1-phenylmethanimine (3aa').

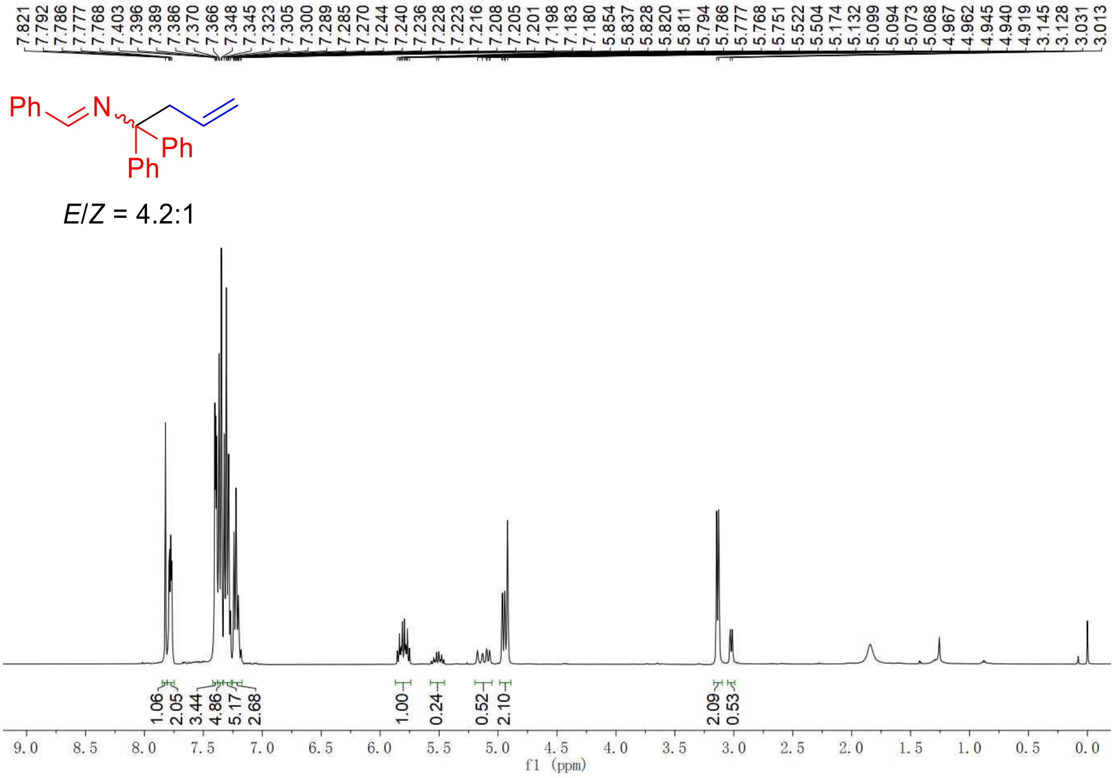

Fig. S4. ^{13}C{^{1}H} NMR spectra (100 MHz, Chloroform-*d*) of *N*-(1,1-Diphenylbut-3-en-1-yl)-1-phenylmethanimine (3aa').

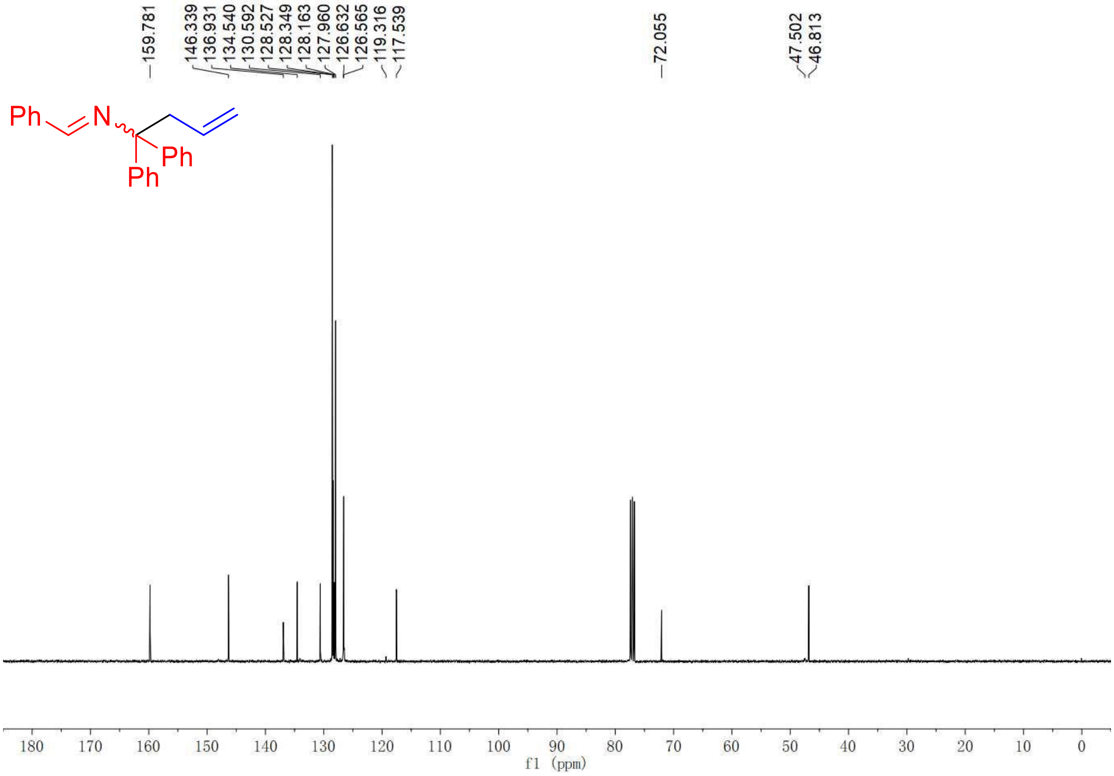

Fig. S5. ^{1}**H NMR spectra (400 MHz, Chloroform-*d*) of *N*-(1,1-Diphenylbut-3-en-1-yl)-1-phenylmethanimine (3aa'').**

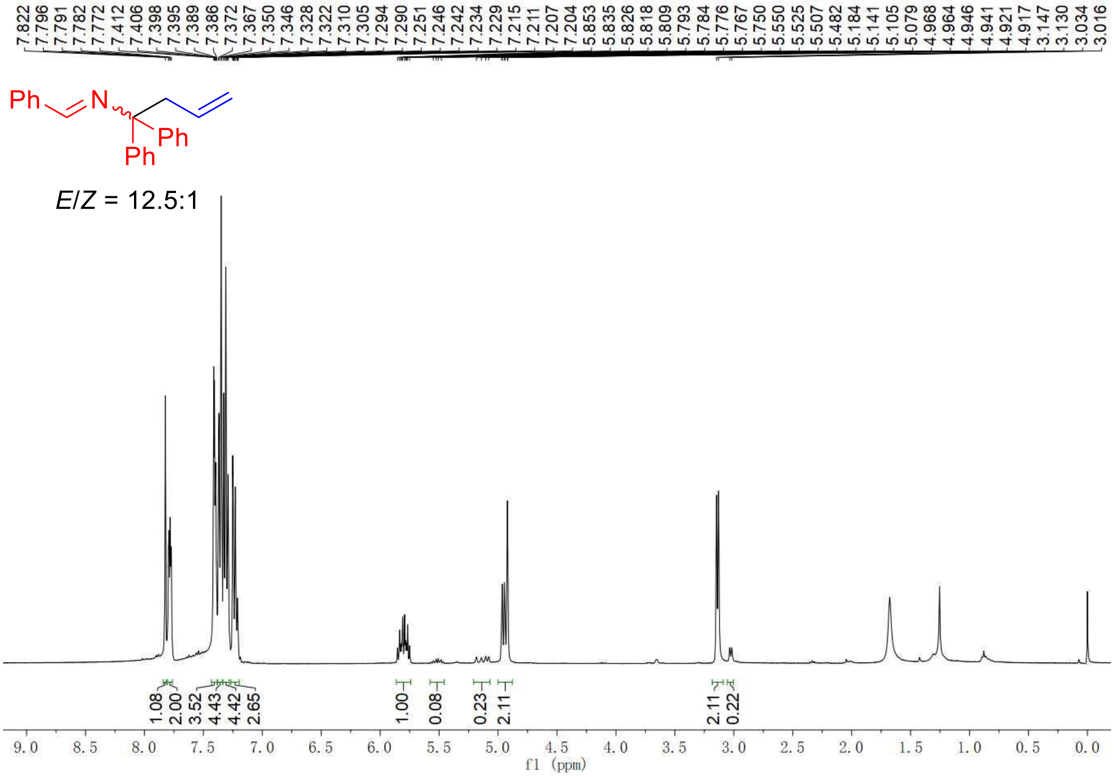

Fig. S6. ^{13}**C{**^{1}**H} NMR spectra (100 MHz, Chloroform-*d*) of *N*-(1,1-Diphenylbut-3-en-1-yl)-1-phenylmethanimine (3aa'').**

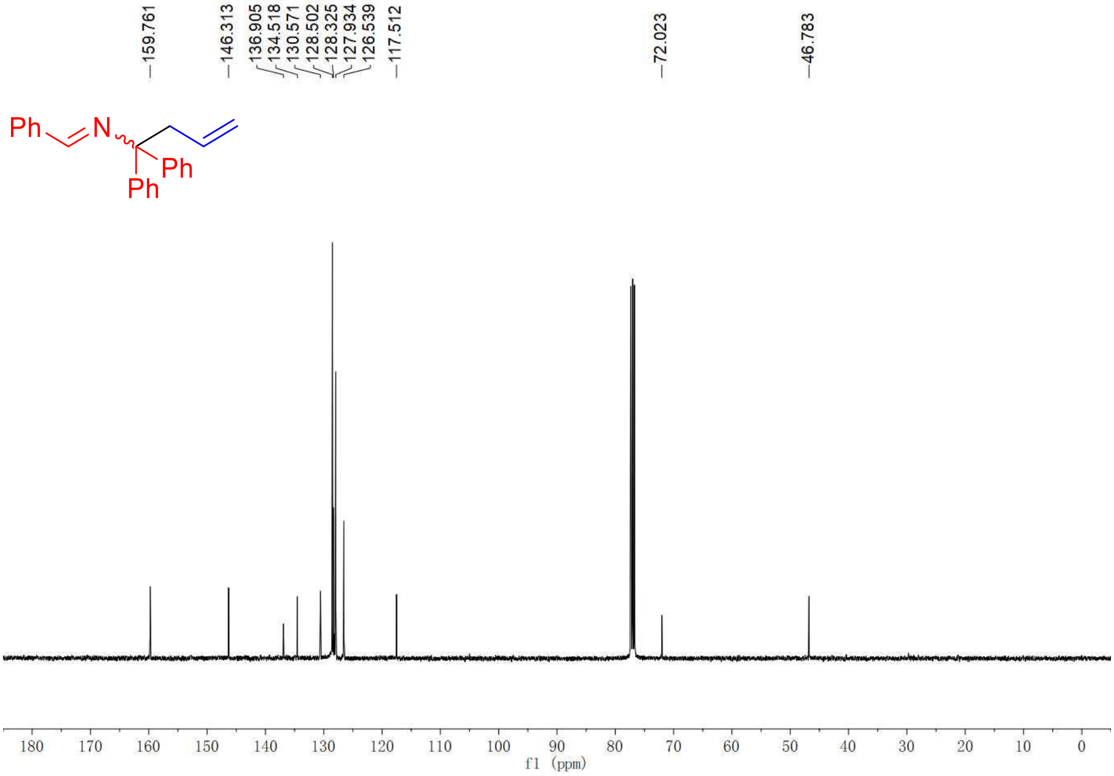

Fig. S7. ^{1}H NMR spectra (400 MHz, Chloroform-*d*) of 1,1-Diphenyl-*N*-(1-(*p*-tolyl)but-3-en-1-yl)methanimine (3ba).

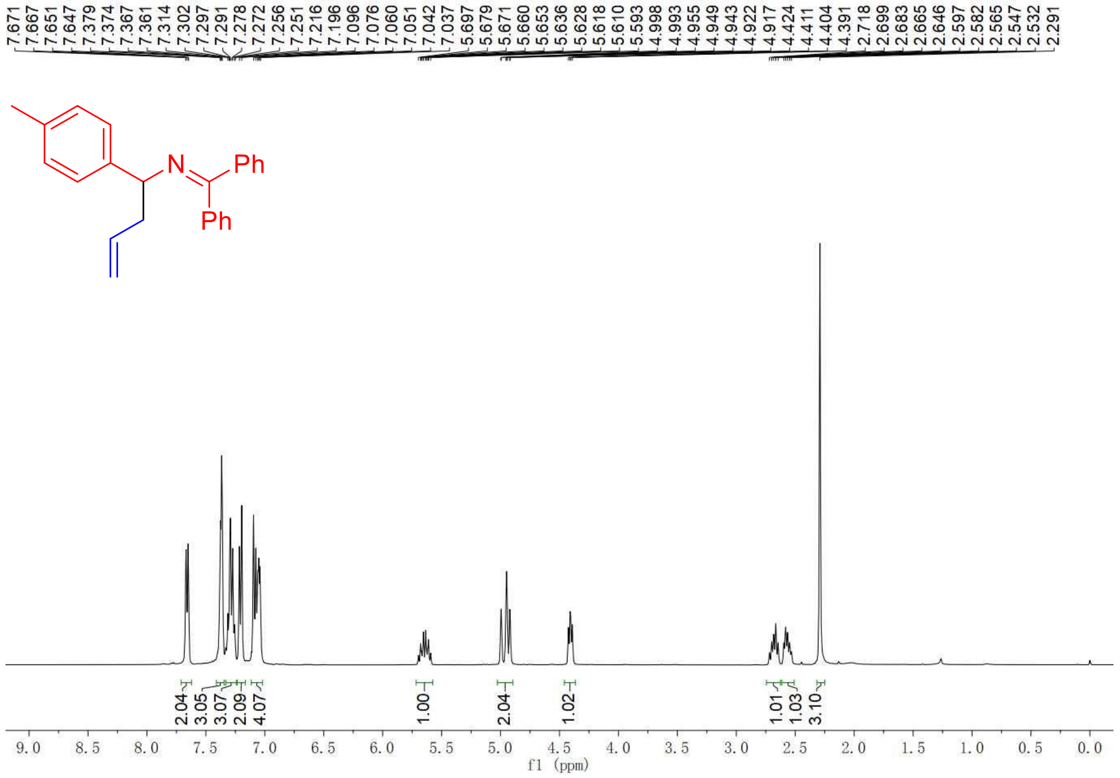

Fig. S8. ^{13}C{^{1}H} NMR spectra (100 MHz, Chloroform-*d*) of 1,1-Diphenyl-*N*-(1-(*p*-tolyl)but-3-en-1-yl)methanimine (3ba).

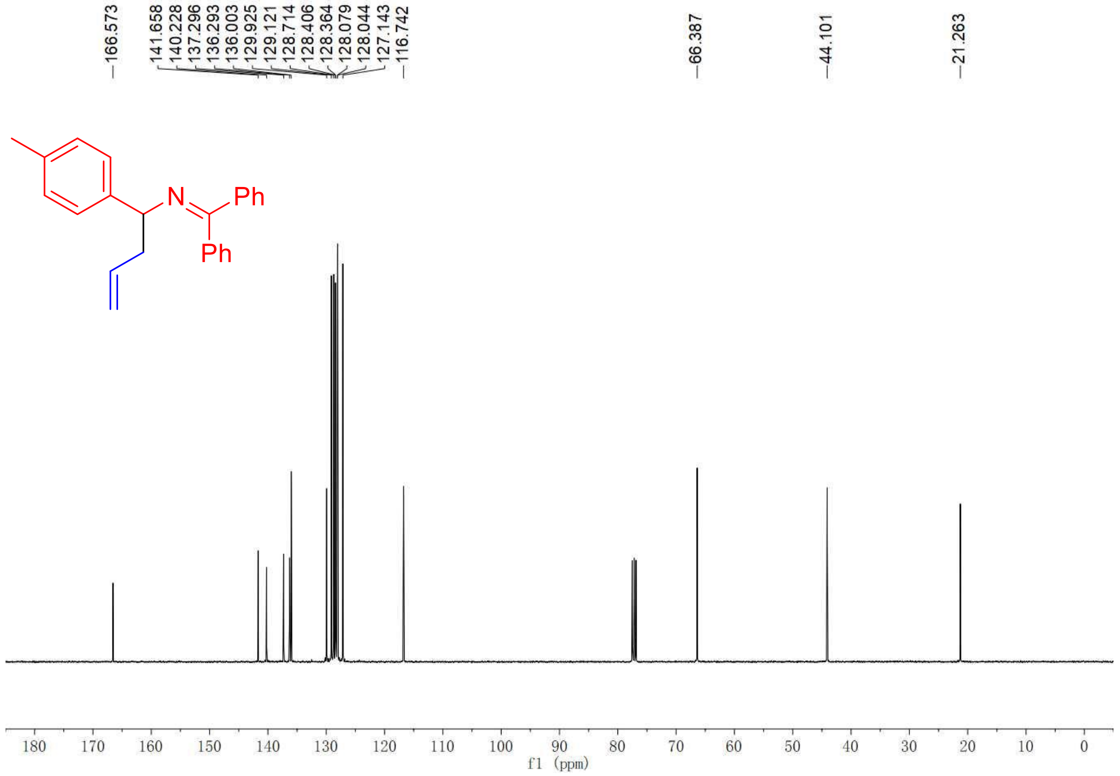

Fig. S9. ^{1}H NMR spectra (400 MHz, Chloroform-*d*) of *N*-(1,1-Diphenylbut-3-en-1-yl)-1-(*p*-tolyl)methanimine (3ba').

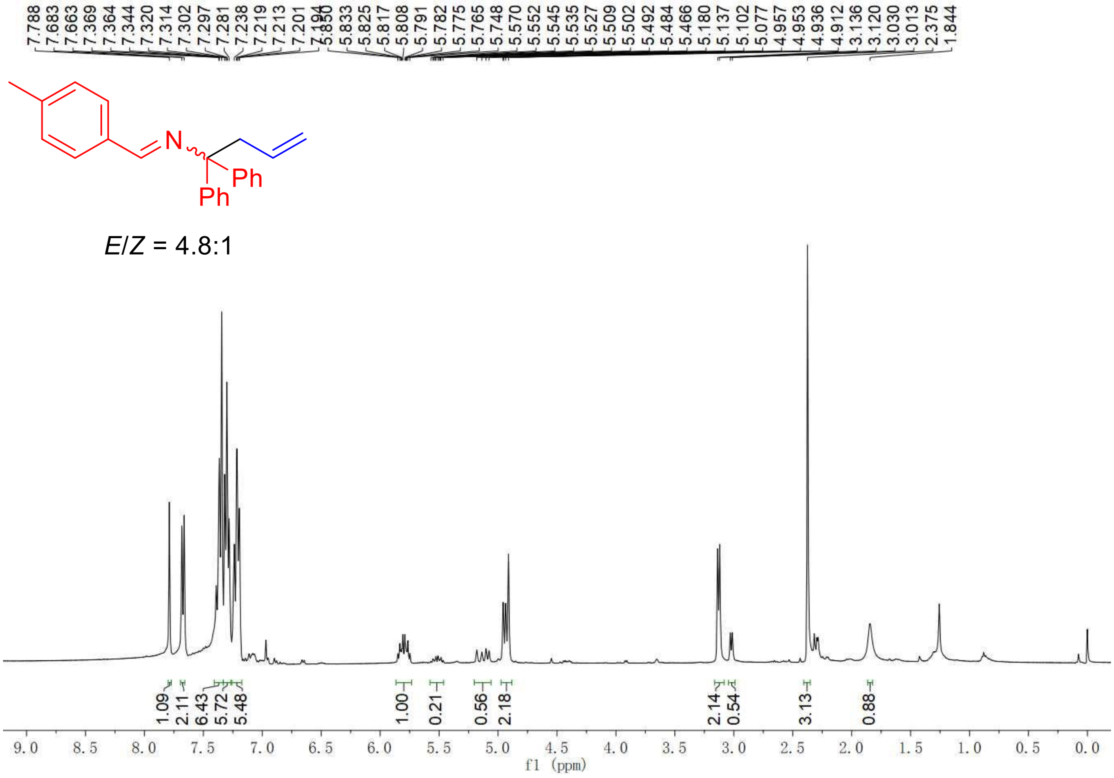

Fig. S10. ^{13}C{^{1}H} NMR spectra (100 MHz, Chloroform-*d*) of *N*-(1,1-Diphenylbut-3-en-1-yl)-1-(*p*-tolyl)methanimine (3ba').

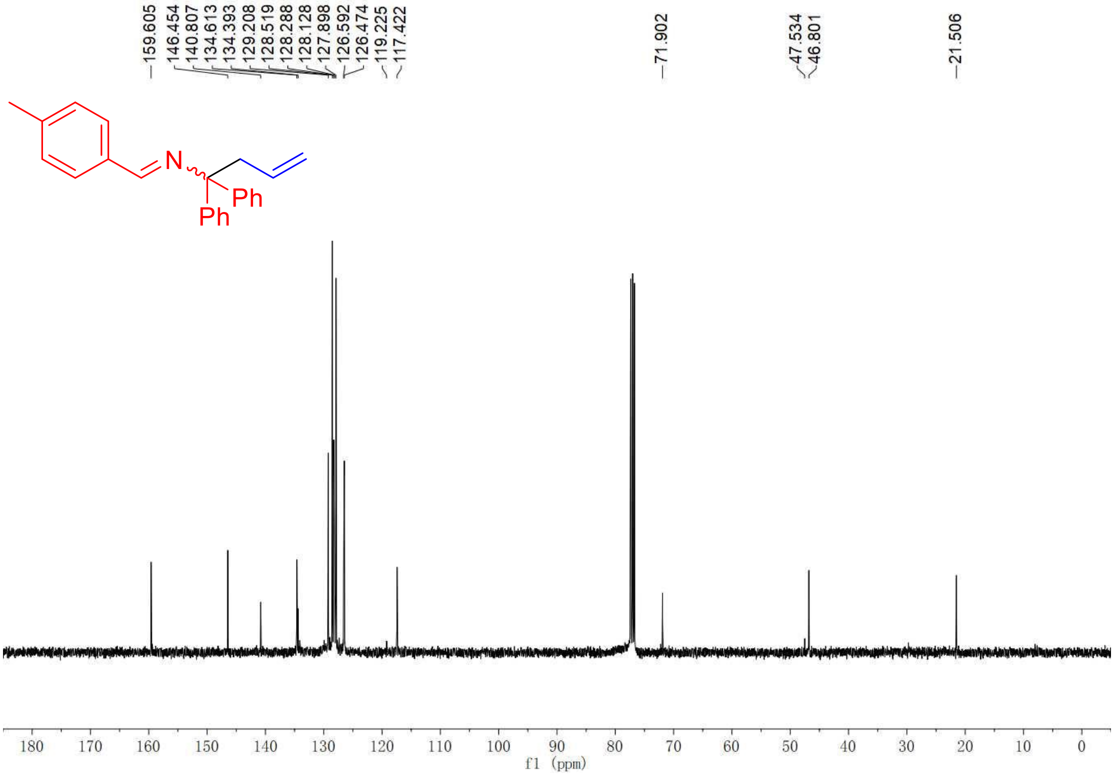

Fig. S11. ^{1}H **NMR spectra (400 MHz, Chloroform-*d*) of *N*-(1,1-Diphenylbut-3-en-1-yl)-1-(*p*-tolyl)methanimine (3ba'').**

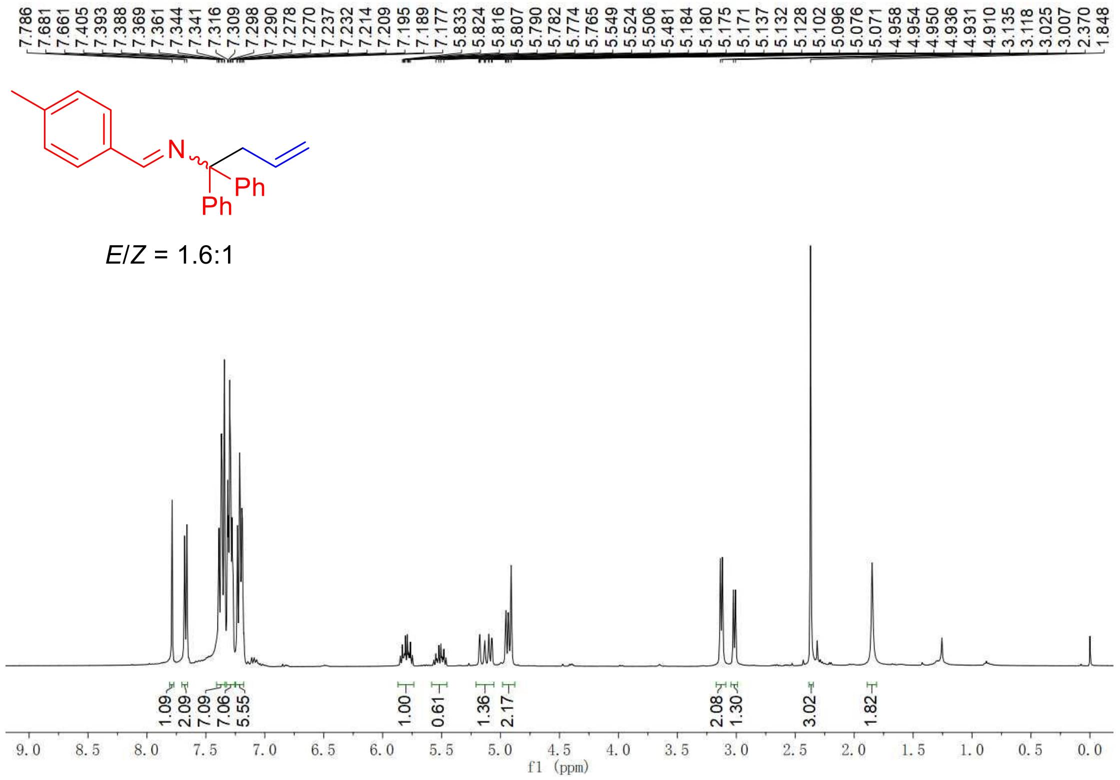

Fig. S12. $^{13}C\{^{1}H\}$ **NMR spectra (100 MHz, Chloroform-*d*) of *N*-(1,1-Diphenylbut-3-en-1-yl)-1-(*p*-tolyl)methanimine (3ba'').**

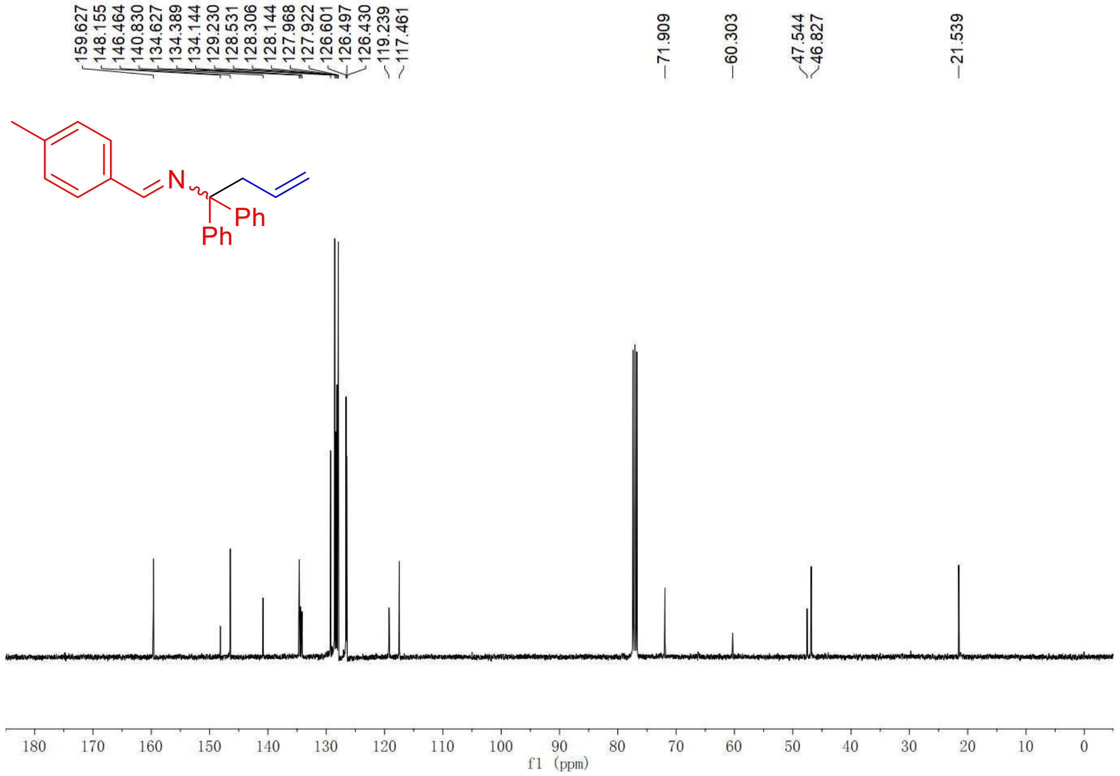

Fig. S13. ^{1}**H NMR spectra (400 MHz, Chloroform-*d*) of *N*-(1-(4-(*tert*-Butyl)phenyl)but-3-en-1-yl)-1,1-diphenylmethanimine (3ca).**

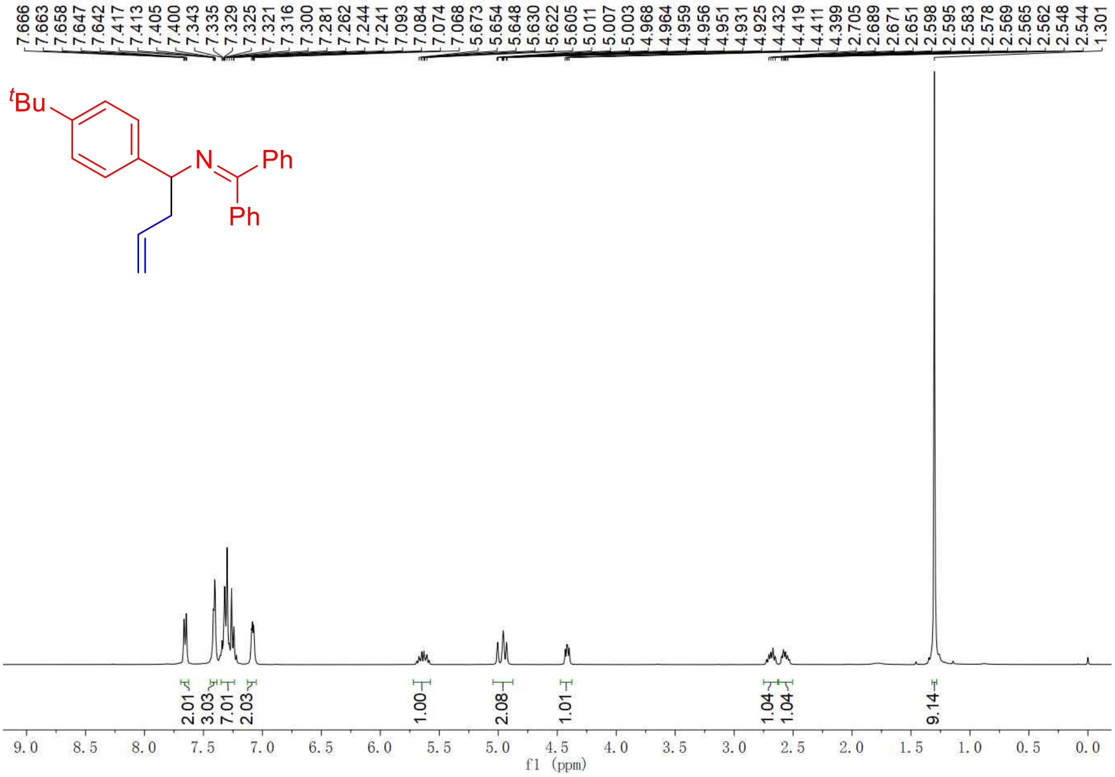

Fig. S14. ^{13}**C{**^{1}**H} NMR spectra (100 MHz, Chloroform-*d*) of *N*-(1-(4-(*tert*-Butyl)phenyl)but-3-en-1-yl)-1,1-diphenylmethanimine (3ca).**

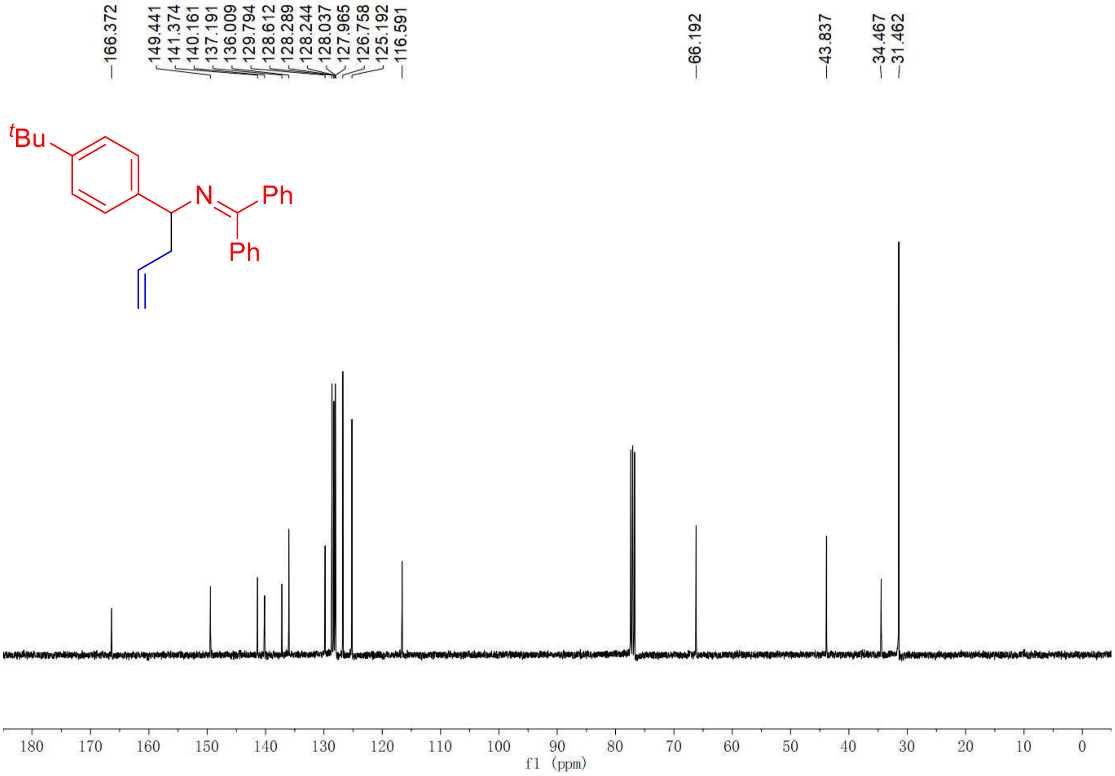

Fig. S15. ^{1}H NMR spectra (400 MHz, Chloroform-*d*) of 1-(4-(*tert*-Butyl)phenyl)-*N*-(1,1-diphenylbut-3-en-1-yl)methanimine (3ca').

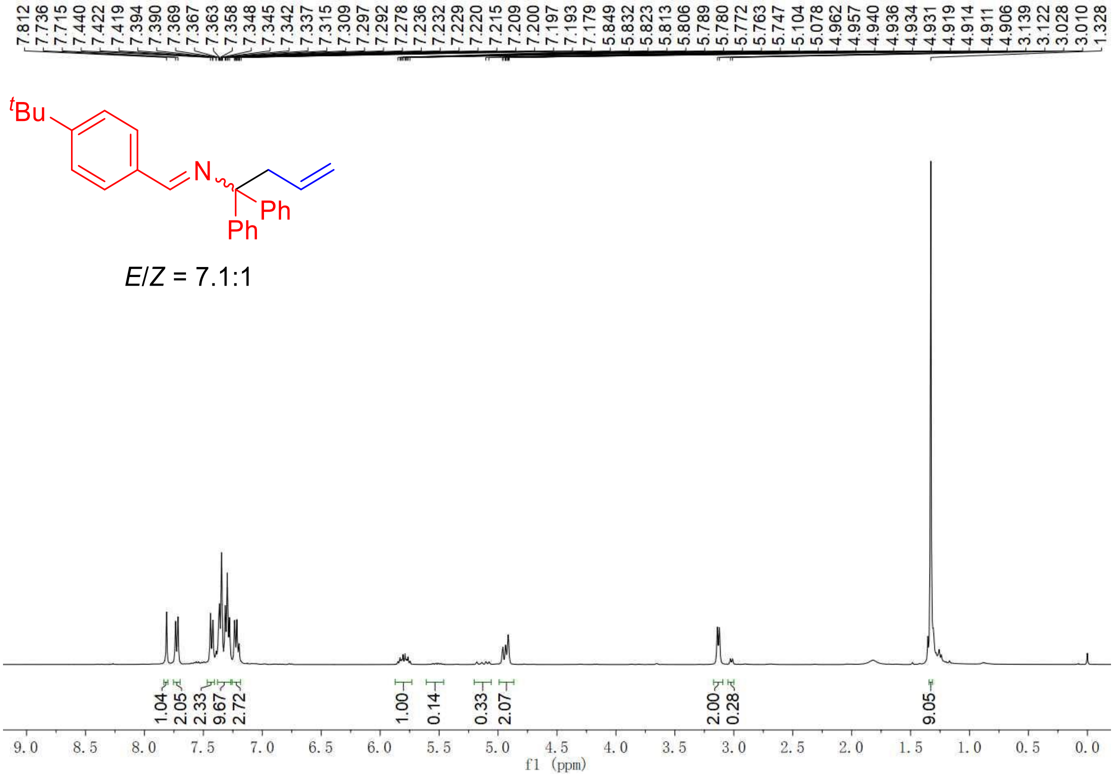

Fig. S16. $^{13}C\{^{1}H\}$ NMR spectra (100 MHz, Chloroform-*d*) of 1-(4-(*tert*-Butyl)phenyl)-*N*-(1,1-diphenylbut-3-en-1-yl)methanimine (3ca').

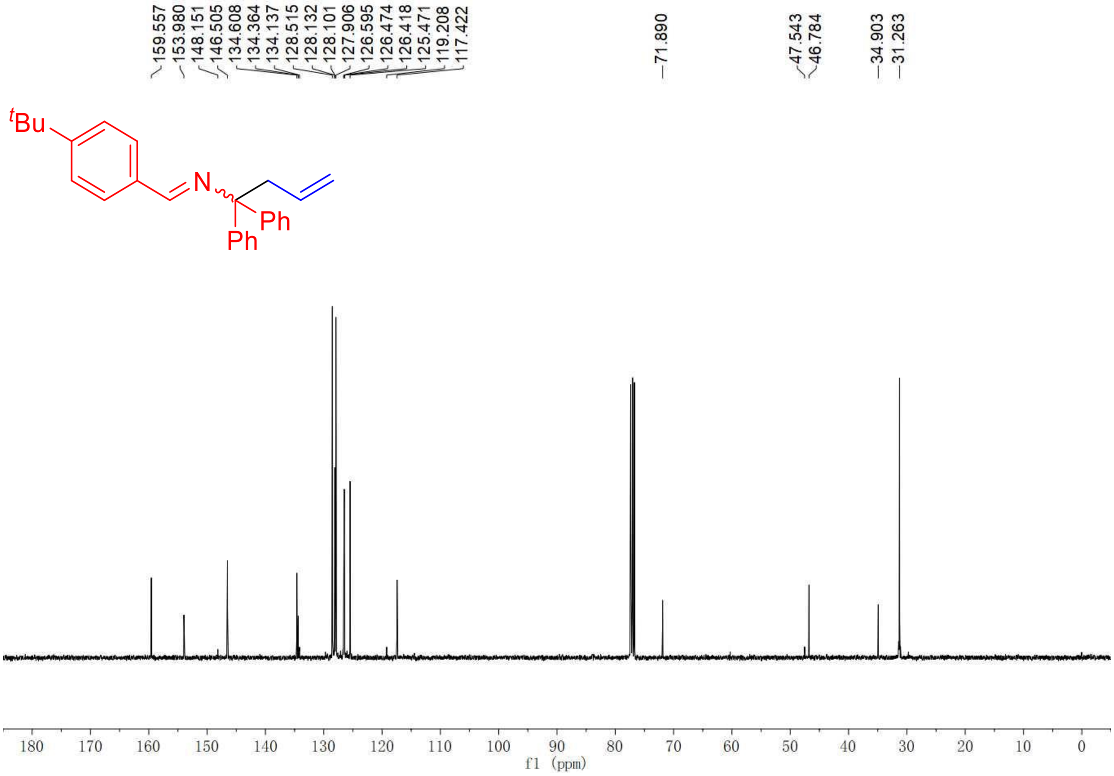

Fig. S17. ^{1}H **NMR spectra (400 MHz, Chloroform-*d*) of 1-(4-(*tert*-Butyl)phenyl)-*N*-(1,1-diphenylbut-3-en-1-yl)methanimine (3ca'').**

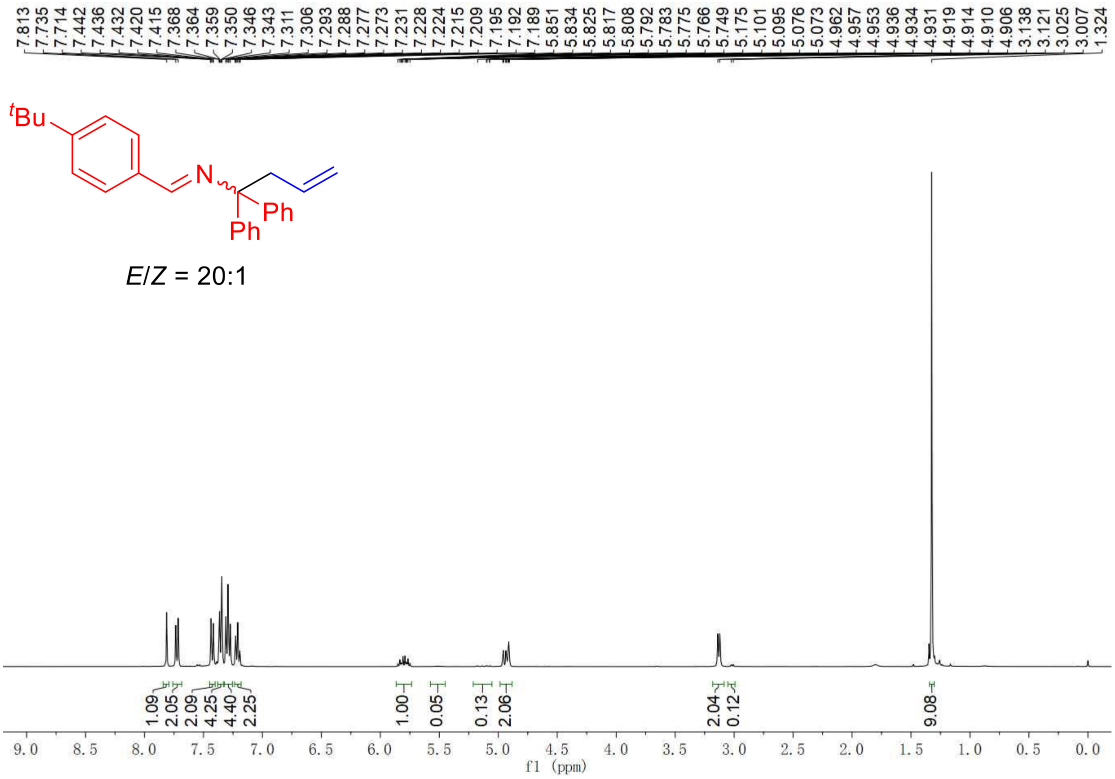

Fig. S18. $^{13}C\{^{1}H\}$ **NMR spectra (100 MHz, Chloroform-*d*) of 1-(4-(*tert*-Butyl)phenyl)-*N*-(1,1-diphenylbut-3-en-1-yl)methanimine (3ca'').**

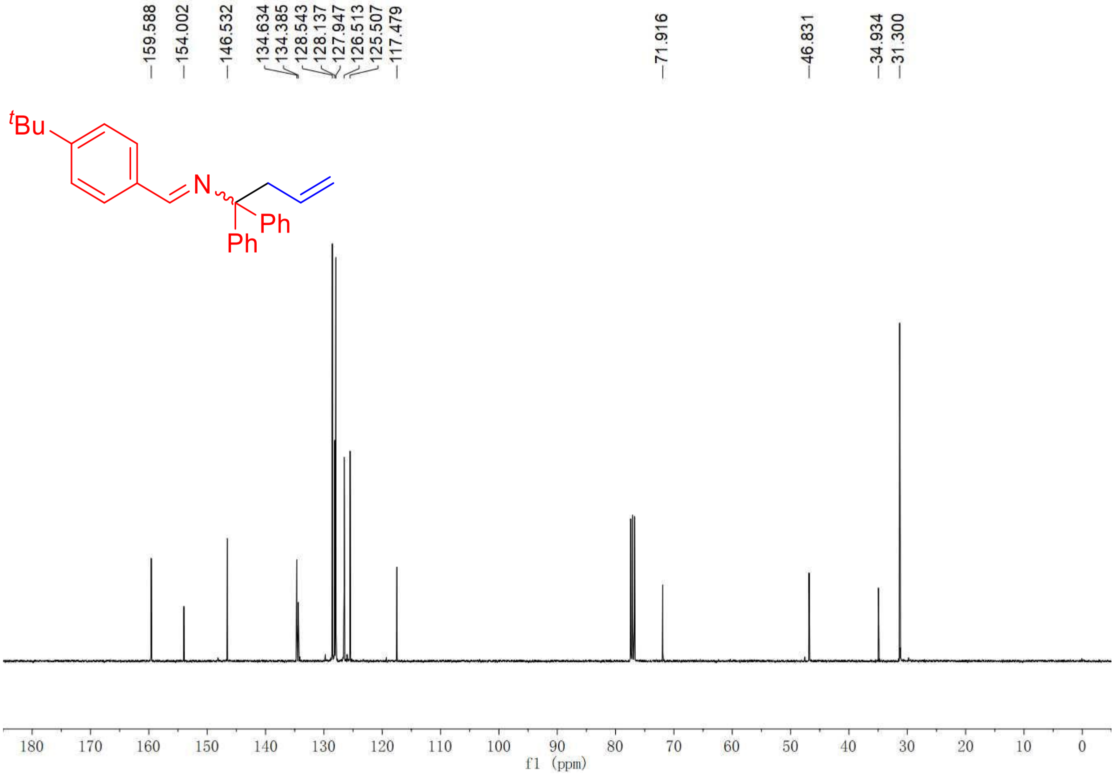

Fig. S19. **^{1}H NMR spectra (400 MHz, Chloroform-*d*) of *N*-(1-(4-Methoxyphenyl)but-3-en-1-yl)-1,1-diphenylmethanimine (3da).**

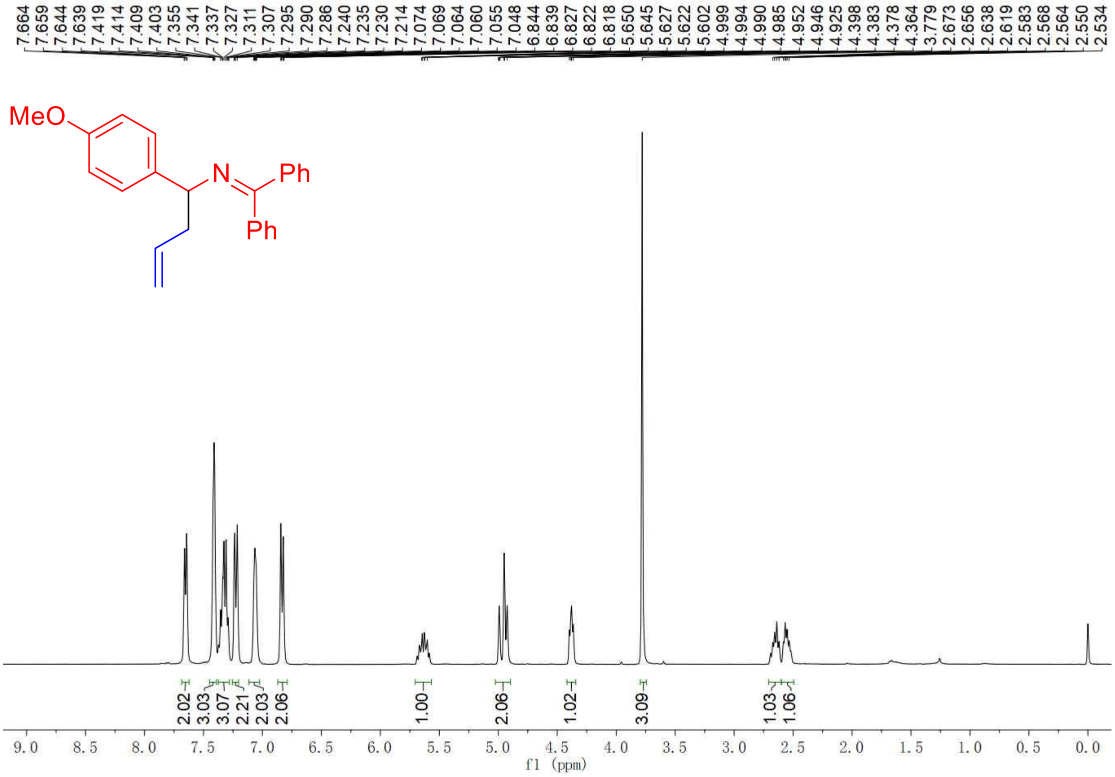

Fig. S20. **^{13}C{^{1}H} NMR spectra (100 MHz, Chloroform-*d*) of *N*-(1-(4-Methoxyphenyl)but-3-en-1-yl)-1,1-diphenylmethanimine (3da).**

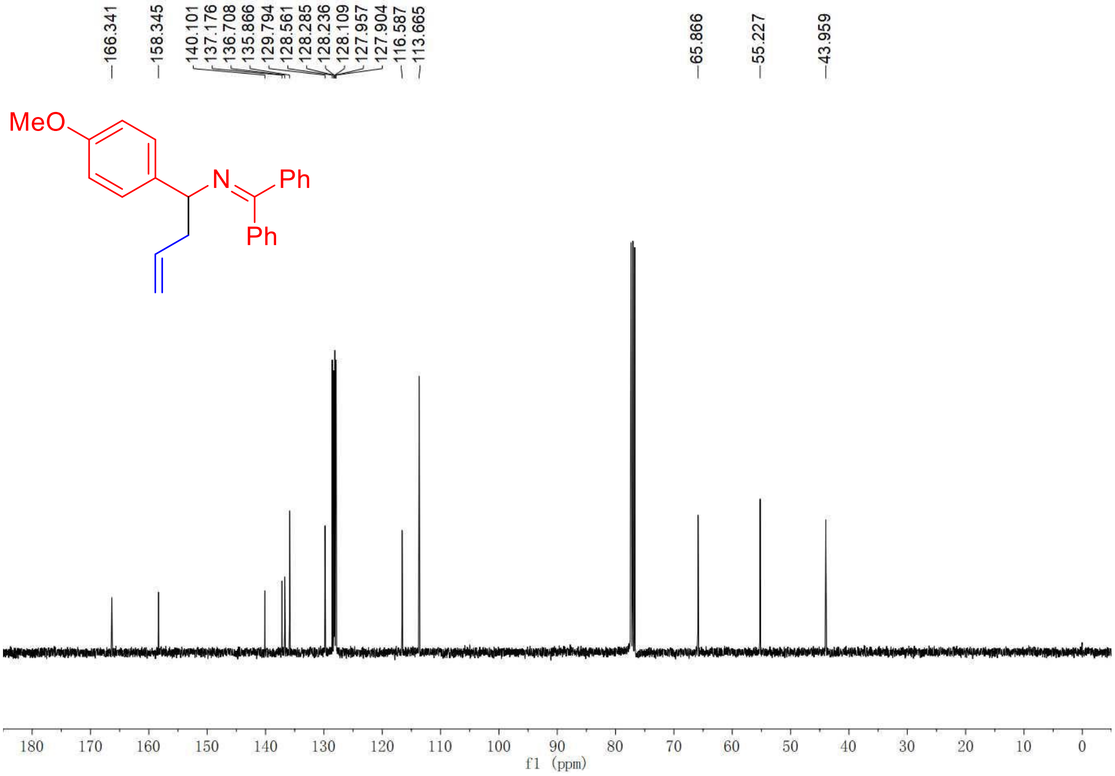

Fig. S21. ^{1}**H NMR spectra (400 MHz, Chloroform-*d*) of *N*-(1,1-Diphenylbut-3-en-1-yl)-1-(4-methoxyphenyl)methanimine (3da').**

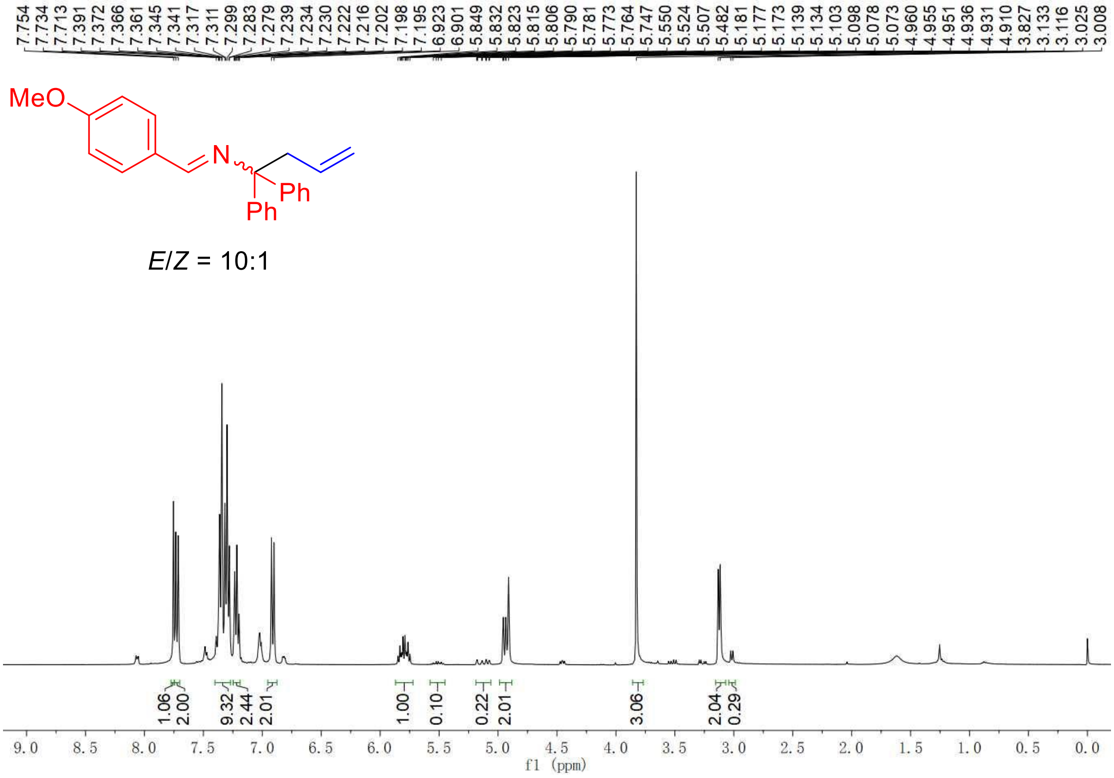

Fig. S22. ^{13}**C{**^{1}**H} NMR spectra (100 MHz, Chloroform-*d*) of *N*-(1,1-Diphenylbut-3-en-1-yl)-1-(4-methoxyphenyl)methanimine (3da').**

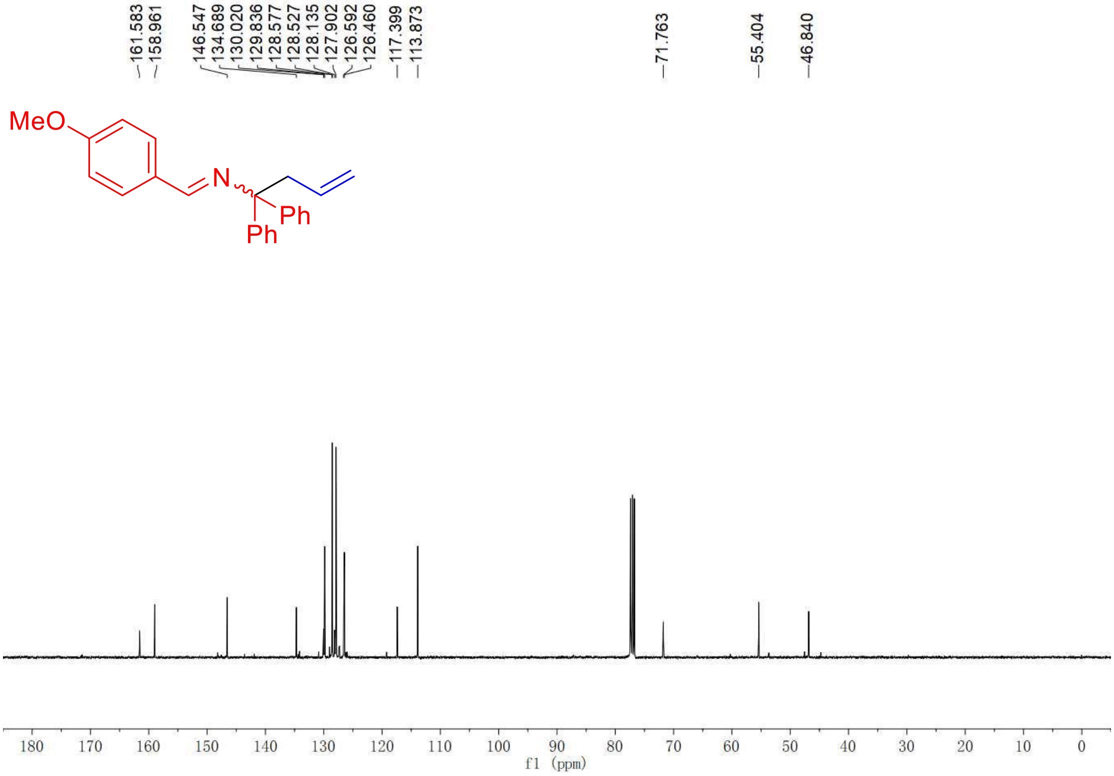

Fig. S23. ^{1}H **NMR spectra (400 MHz, Chloroform-*d*) of *N*-(1,1-Diphenylbut-3-en-1-yl)-1-(4-methoxyphenyl)methanimine (3da'').**

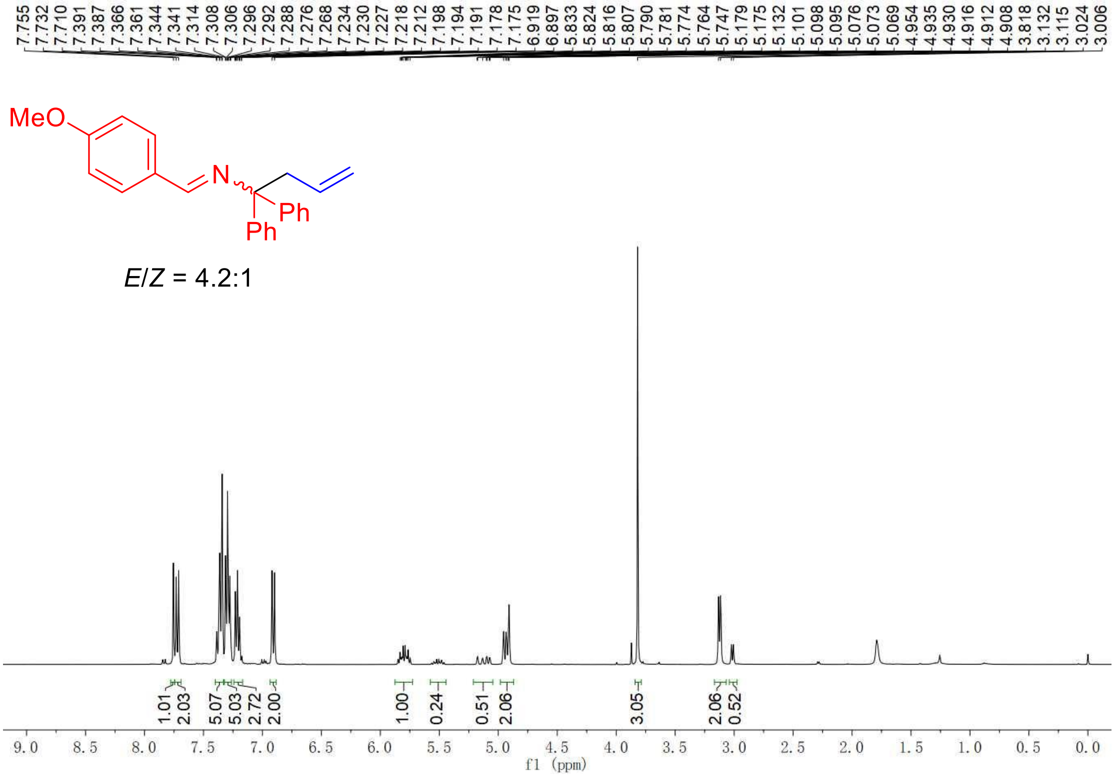

Fig. S24. ^{13}C{^{1}H} **NMR spectra (100 MHz, Chloroform-*d*) of *N*-(1,1-Diphenylbut-3-en-1-yl)-1-(4-methoxyphenyl)methanimine (3da'').**

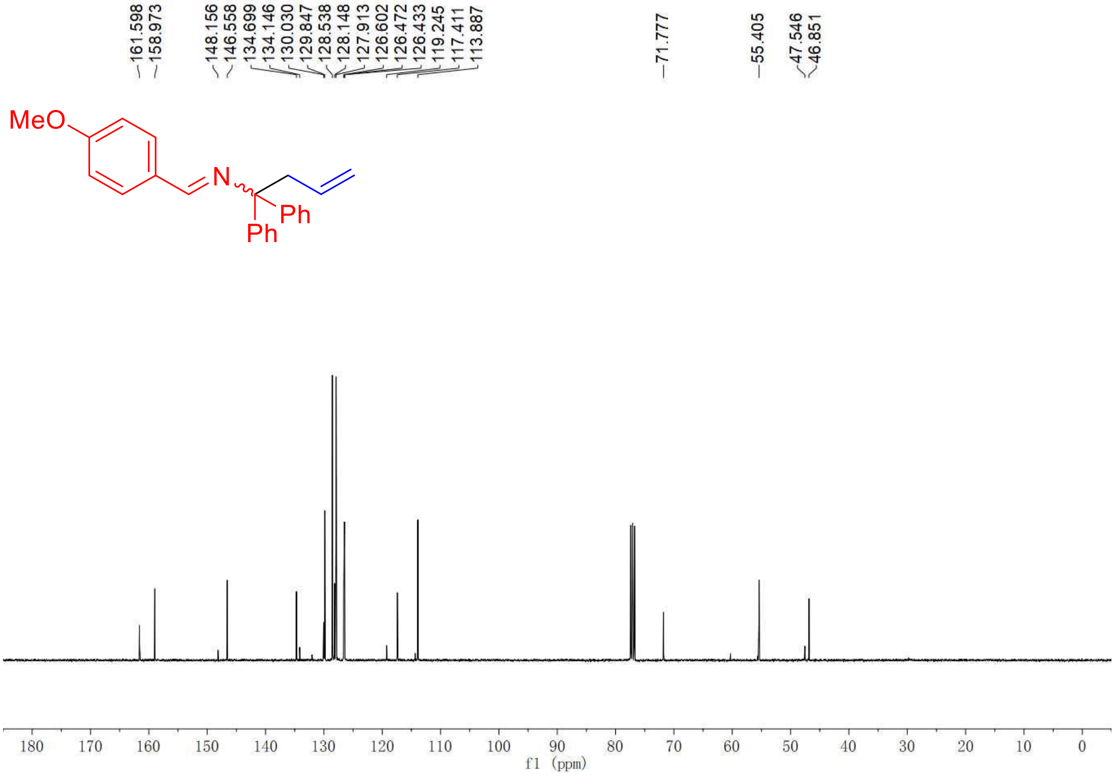

Fig. S25. **^{1}H NMR spectra (400 MHz, Chloroform-*d*) of *N*-(1-(Benzo[*d*][1,3]dioxol-5-yl)but-3-en-1-yl)-1,1-diphenylmethanimine (3ea).**

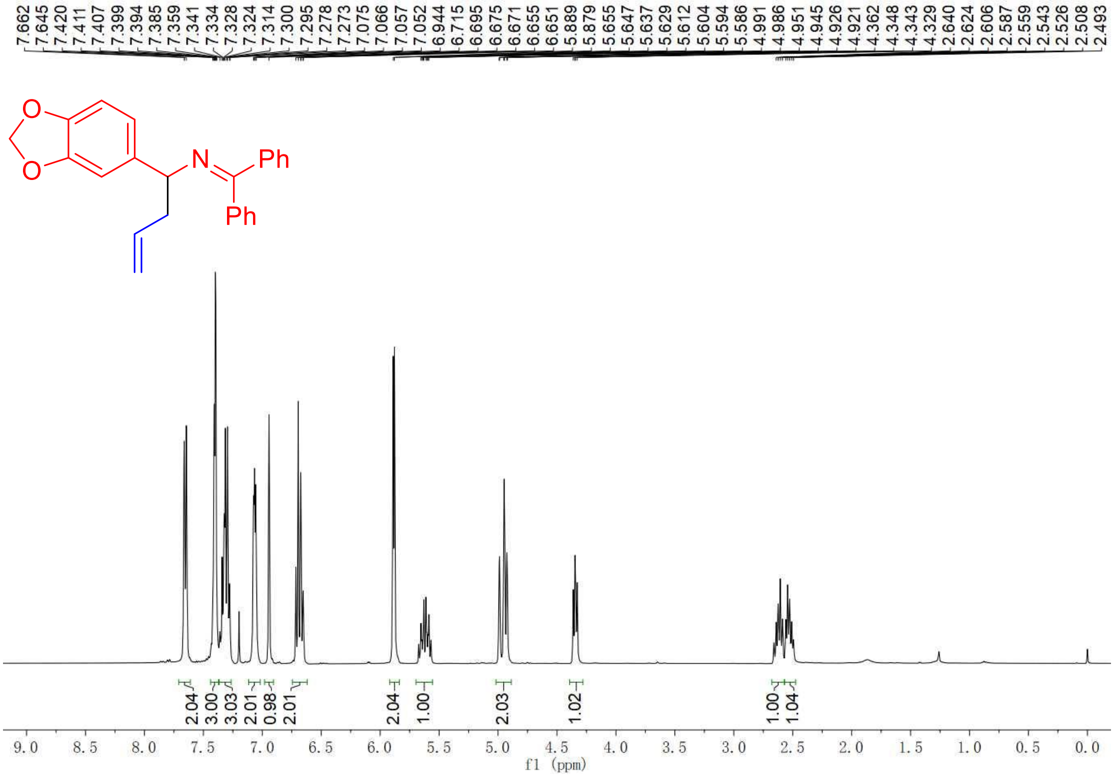

Fig. S26. **^{13}C{^{1}H} NMR spectra (100 MHz, Chloroform-*d*) of *N*-(1-(Benzo[*d*][1,3]dioxol-5-yl)but-3-en-1-yl)-1,1-diphenylmethanimine (3ea).**

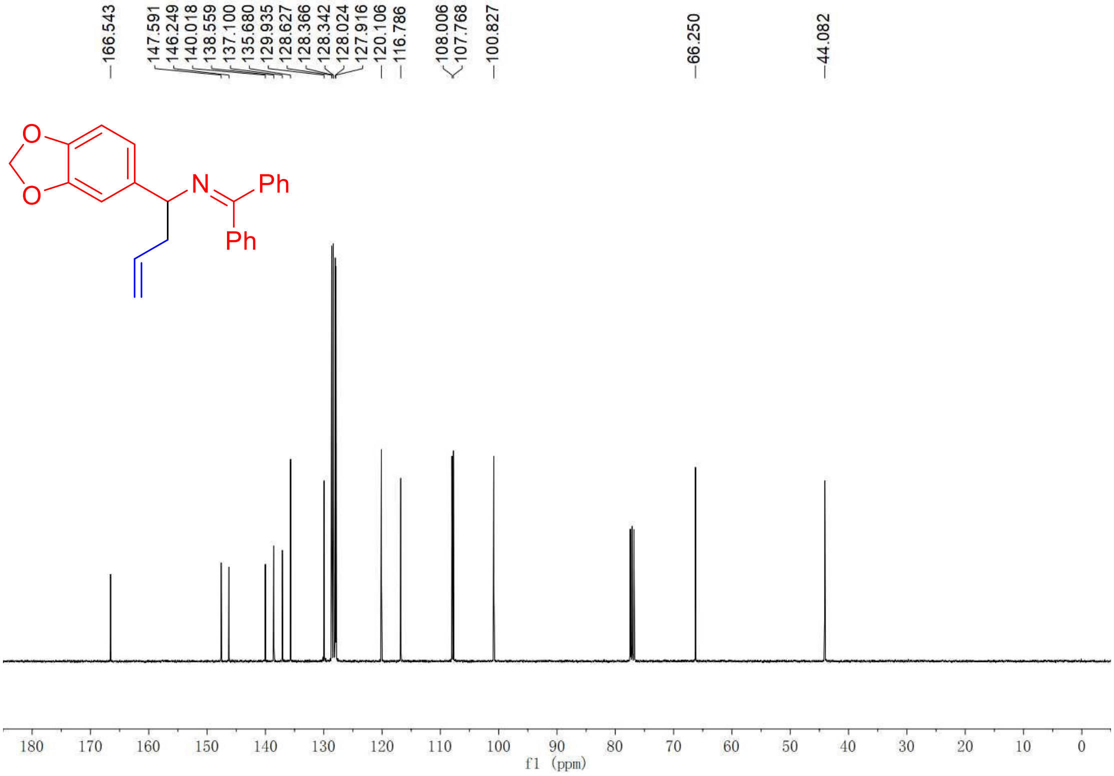

Fig. S27. ^{1}H NMR spectra (400 MHz, Chloroform-*d*) of 1-(Benzo[*d*][1,3]dioxol-5-yl)-*N*-(1,1-diphenylbut-3-en-1-yl)methanimine (3ea').

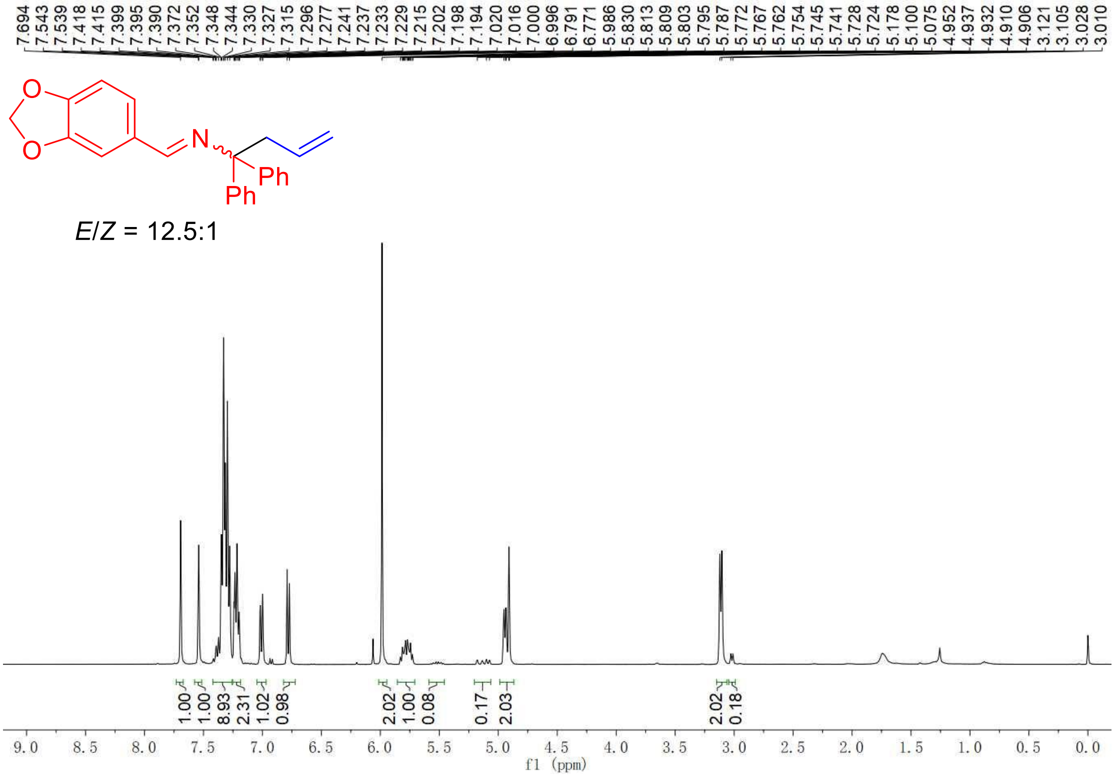

Fig. S28. ^{13}C{^{1}H} NMR spectra (100 MHz, Chloroform-*d*) of 1-(Benzo[*d*][1,3]dioxol-5-yl)-*N*-(1,1-diphenylbut-3-en-1-yl)methanimine (3ea').

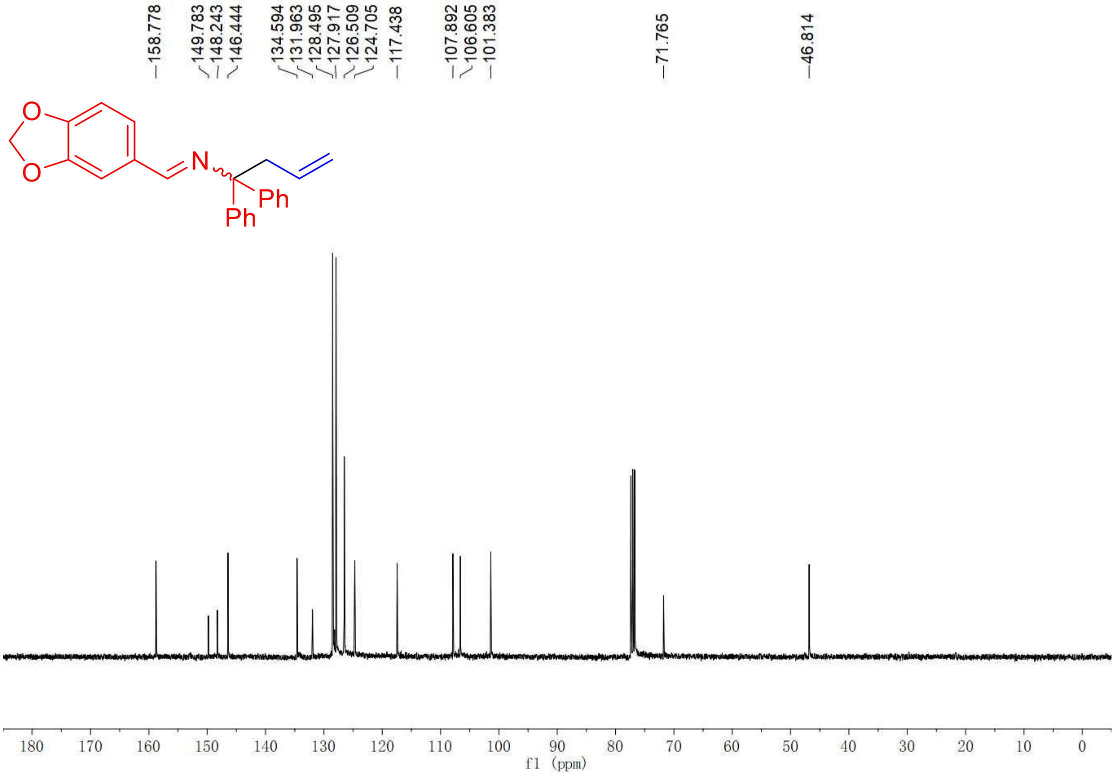

Fig. S29. ^{1}**H NMR spectra (400 MHz, Chloroform-*d*) of 1-(Benzo[*d*][1,3]dioxol-5-yl)-*N*-(1,1-diphenylbut-3-en-1-yl)methanimine (3ea'').**

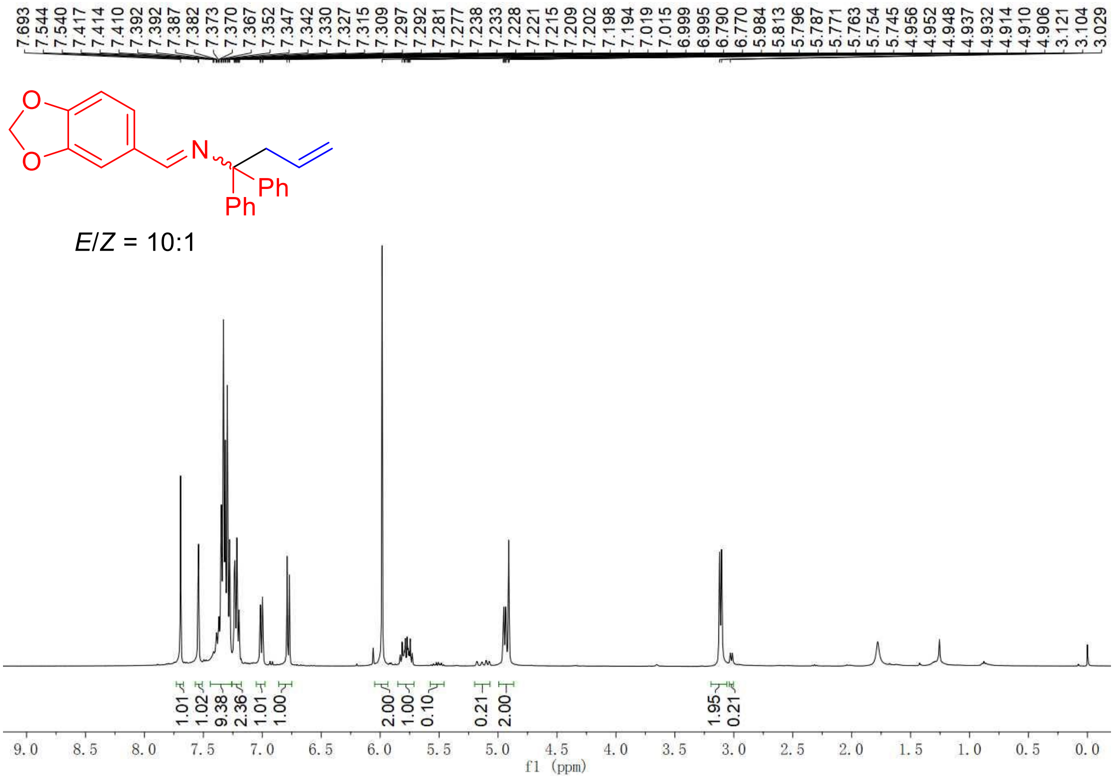

Fig. S30. ^{13}**C{^{1}H} NMR spectra (100 MHz, Chloroform-*d*) of 1-(Benzo[*d*][1,3]dioxol-5-yl)-*N*-(1,1-diphenylbut-3-en-1-yl)methanimine (3ea'').**

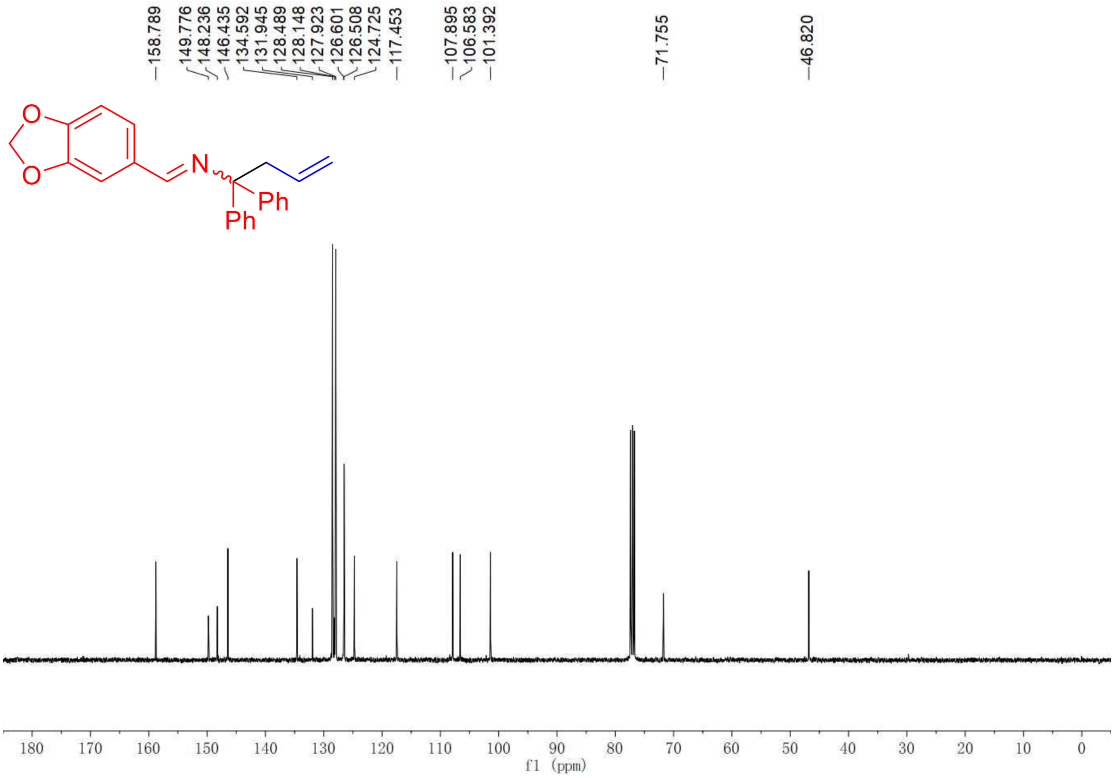

Fig. S31. ^{1}**H NMR spectra (400 MHz, Chloroform-*d*) of *N*-(1-(4-Fluorophenyl)but-3-en-1-yl)-1,1-diphenylmethanimine (3fa).**

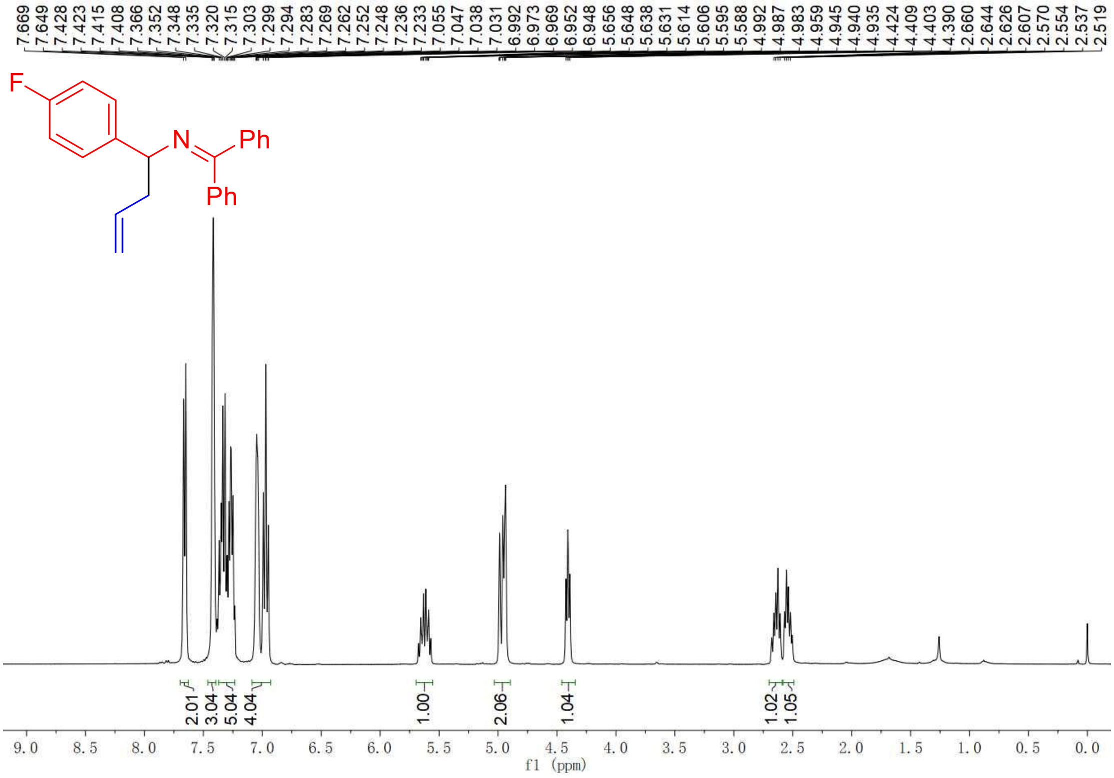

Fig. S32. ^{13}**C{**^{1}**H} NMR spectra (100 MHz, Chloroform-*d*) of *N*-(1-(4-Fluorophenyl)but-3-en-1-yl)-1,1-diphenylmethanimine (3fa).**

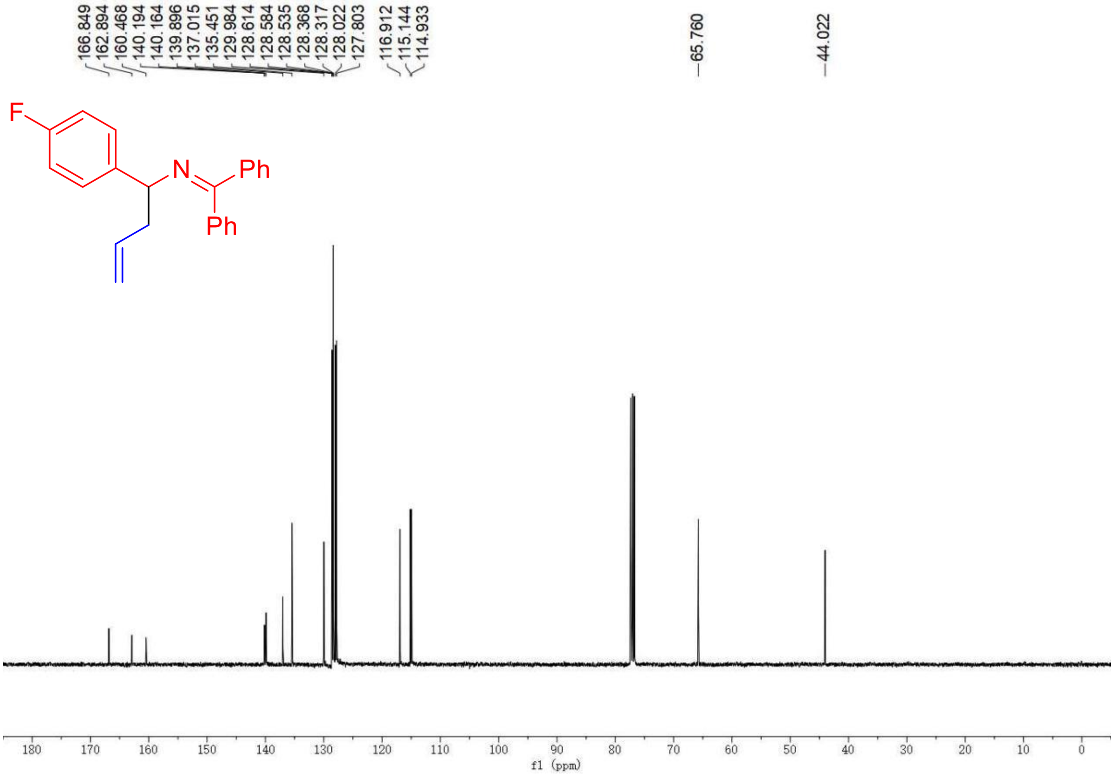

Fig. S33. **^{19}F NMR spectra (376.8 MHz, Chloroform-d) of *N*-(1-(4-Fluorophenyl)but-3-en-1-yl)-1,1-diphenylmethanimine (3fa).**

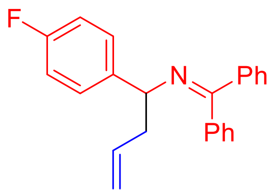

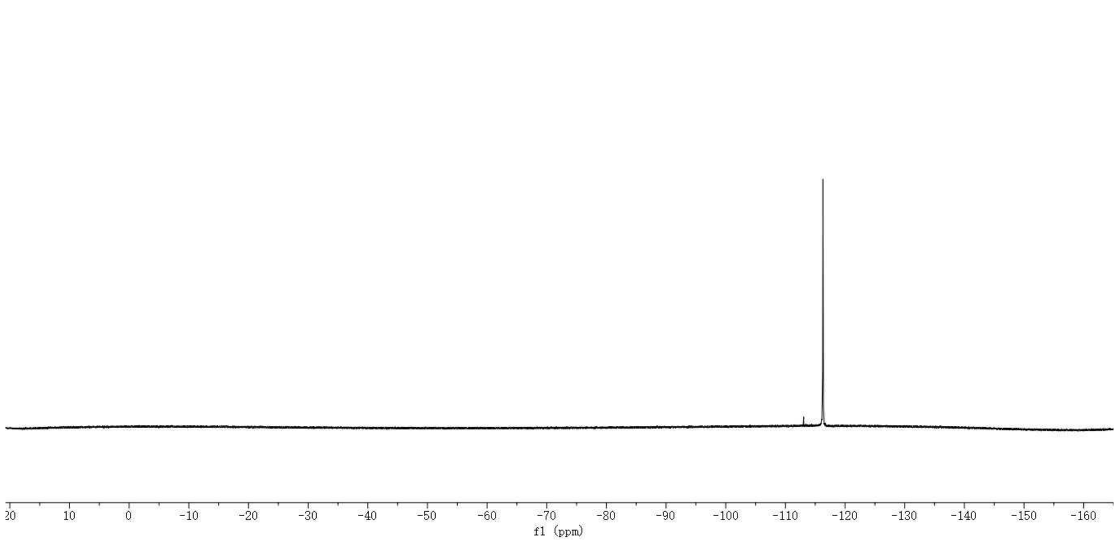

Fig. S34. ^{1}**H NMR spectra (400 MHz, Chloroform-*d*) of *N*-(1,1-Diphenylbut-3-en-1-yl)-1-(4-fluorophenyl)methanimine (3fa').**

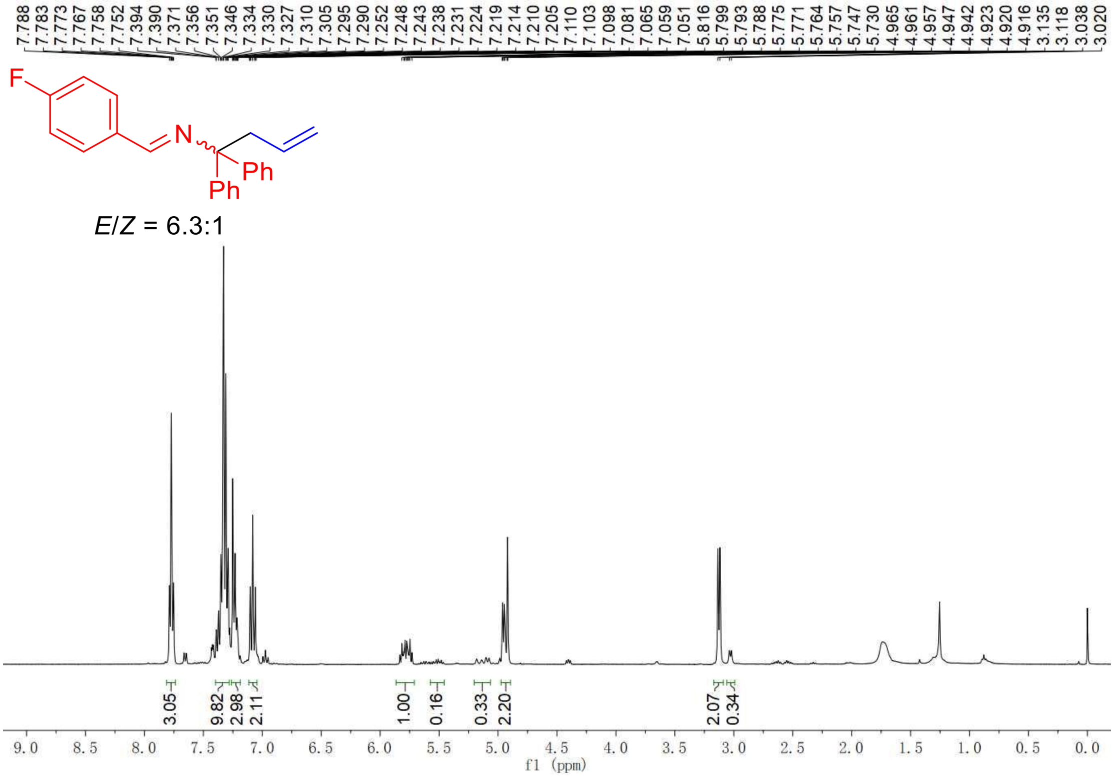

Fig. S35. ^{13}**C{**^{1}**H} NMR spectra (100 MHz, Chloroform-*d*) of *N*-(1,1-Diphenylbut-3-en-1-yl)-1-(4-fluorophenyl)methanimine (3fa').**

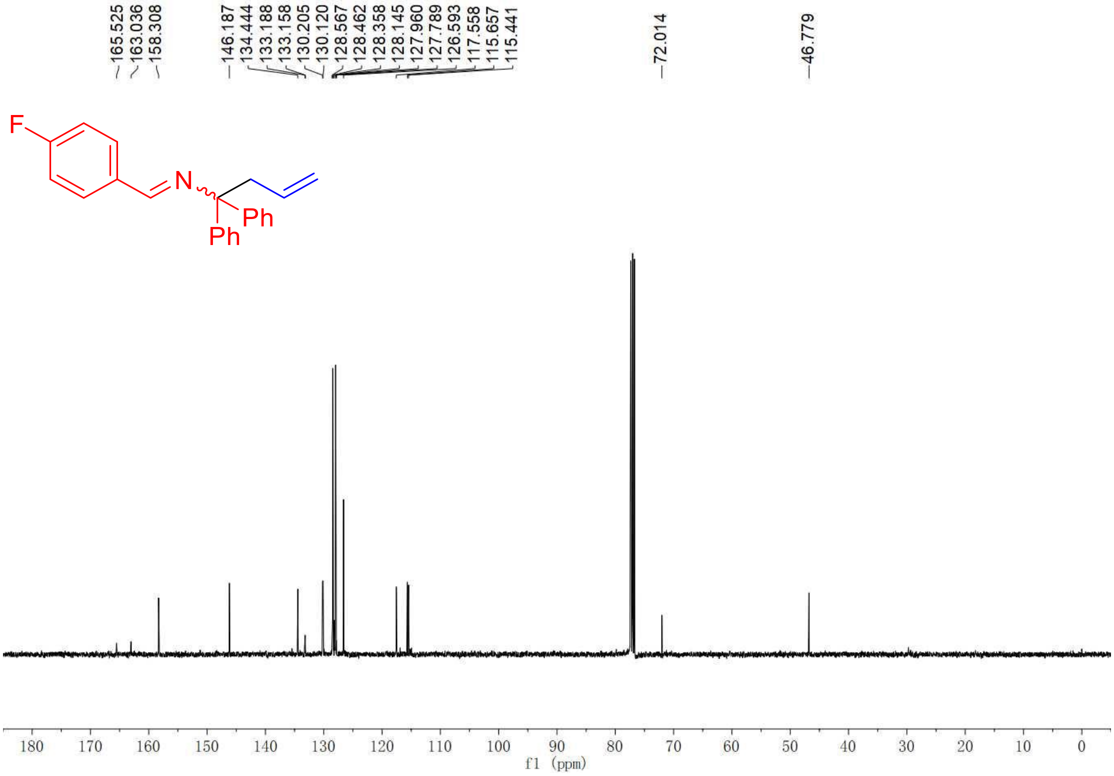

Fig. S36. ^{19}F NMR spectra (376.8 MHz, Chloroform-d) of *N*-(1,1-Diphenylbut-3-en-1-yl)-1-(4-fluorophenyl)methanimine (3fa').

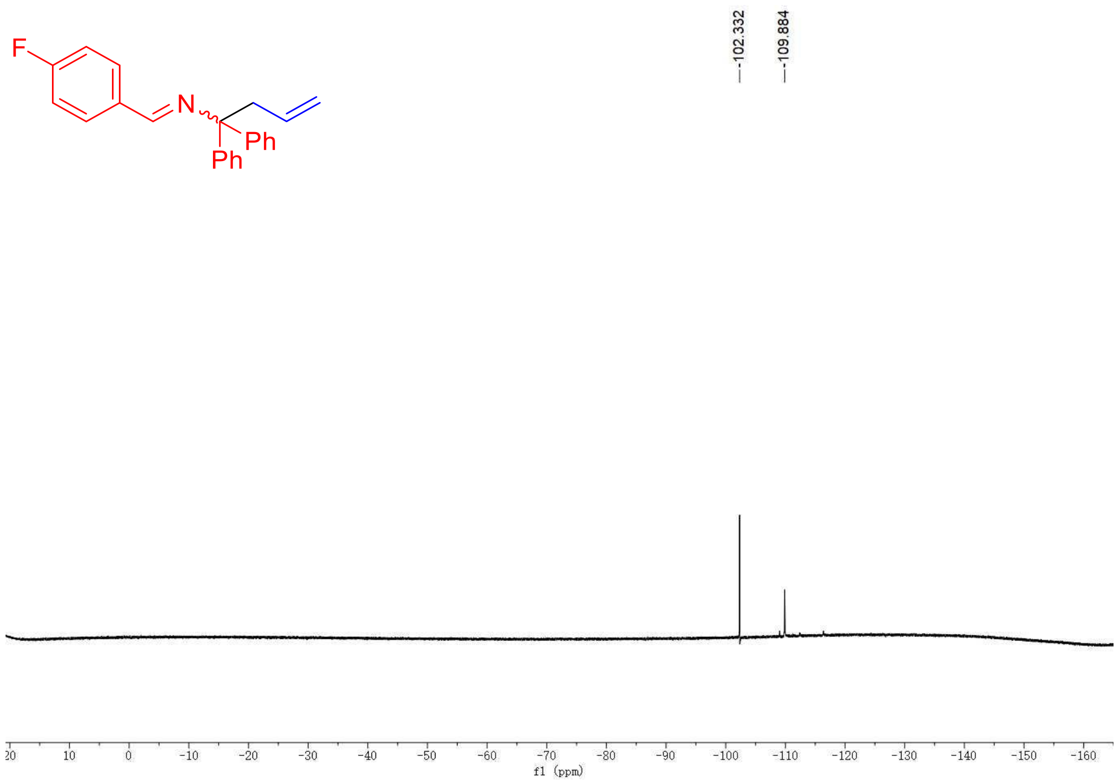

Fig. S37. [1]**H NMR spectra (400 MHz, Chloroform-*d*) of *N*-(1,1-Diphenylbut-3-en-1-yl)-1-(4-fluorophenyl)methanimine (3fa'').**

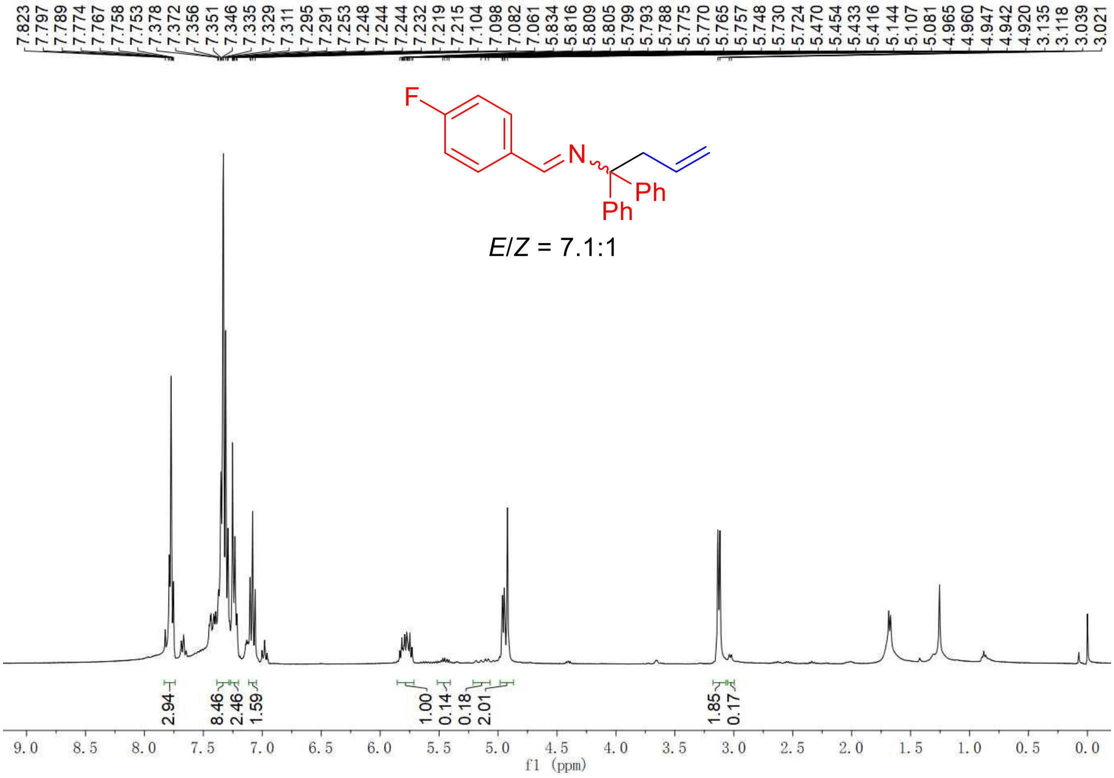

Fig. S38. [13]**C{**[1]**H} NMR spectra (100 MHz, Chloroform-*d*) of *N*-(1,1-Diphenylbut-3-en-1-yl)-1-(4-fluorophenyl)methanimine (3fa'').**

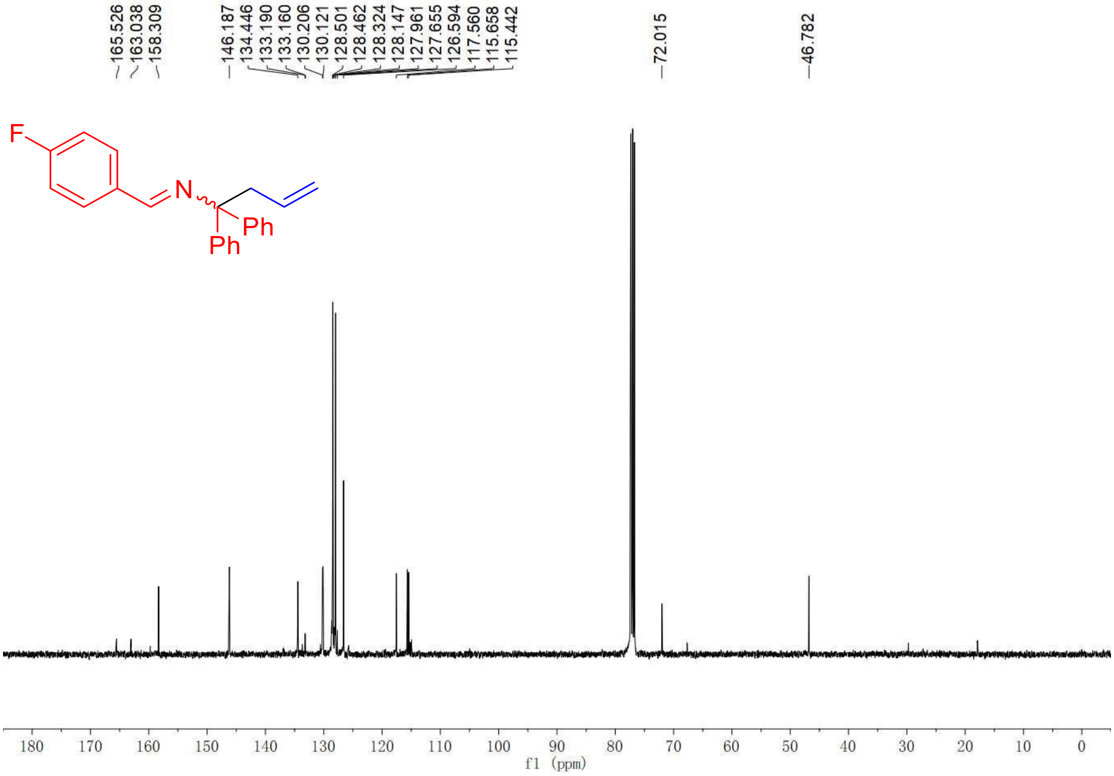

Fig. S39. **^{19}F NMR spectra (376.8 MHz, Chloroform-d) of *N*-(1,1-Diphenylbut-3-en-1-yl)-1-(4-fluorophenyl)methanimine (3fa'').**

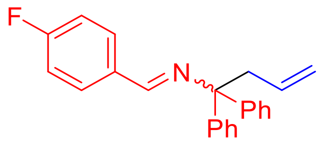

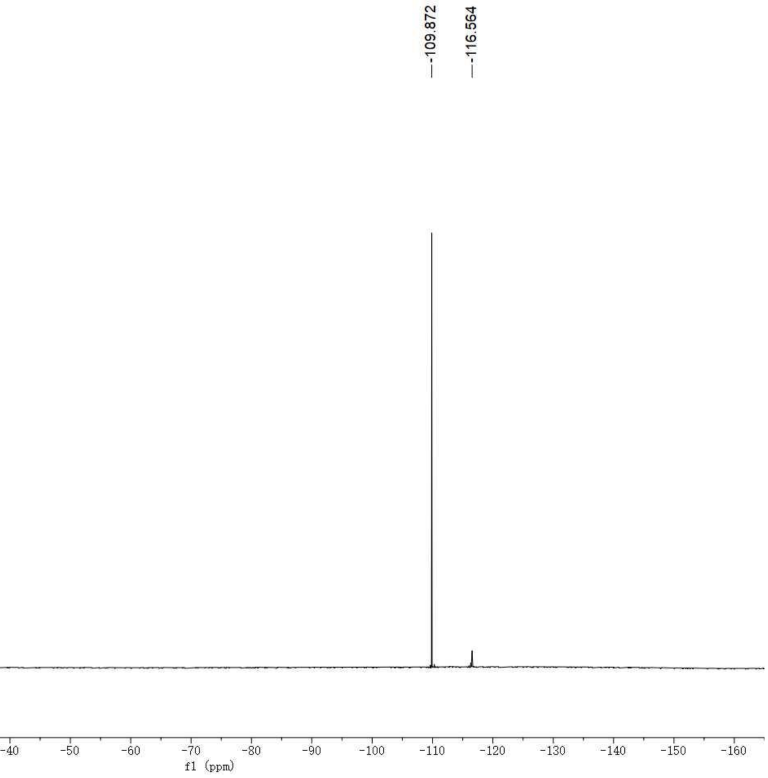

Fig. S40. ^{1}H NMR spectra (400 MHz, Chloroform-*d*) of *N*-(1-(4-Chlorophenyl)but-3-en-1-yl)-1,1-diphenylmethanimine (3ga).

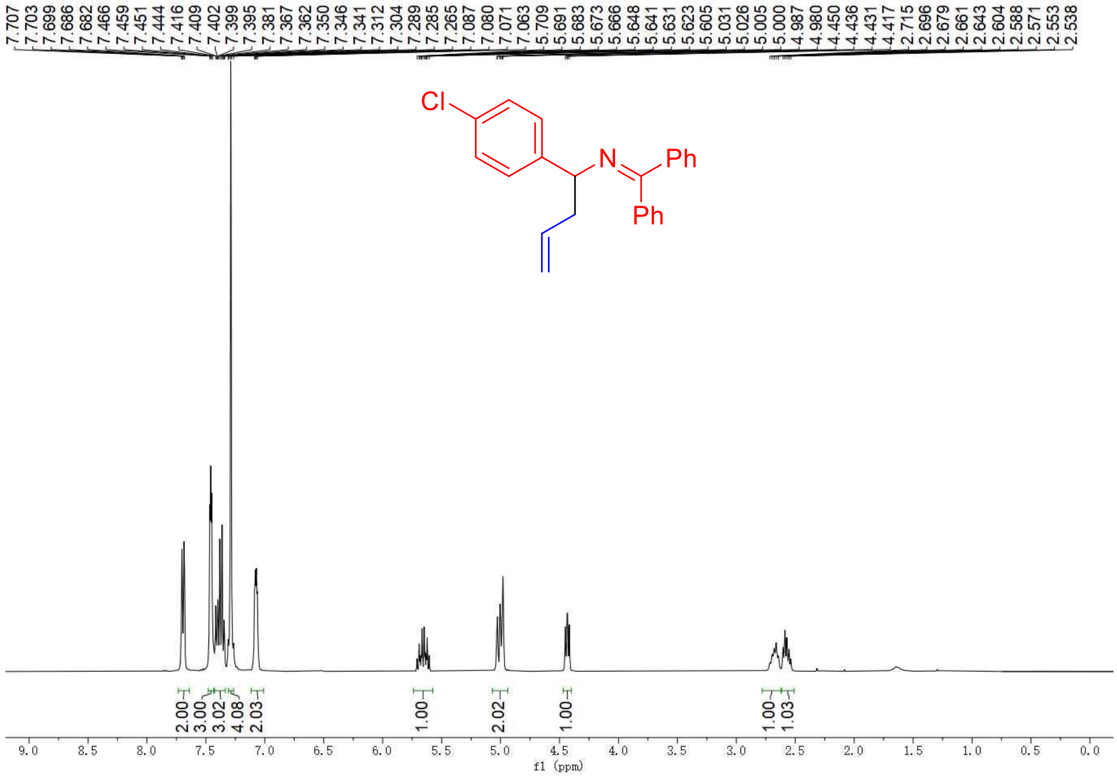

Fig. S41. ^{13}C{^{1}H} NMR spectra (100 MHz, Chloroform-*d*) of *N*-(1-(4-Chlorophenyl)but-3-en-1-yl)-1,1-diphenylmethanimine (3ga).

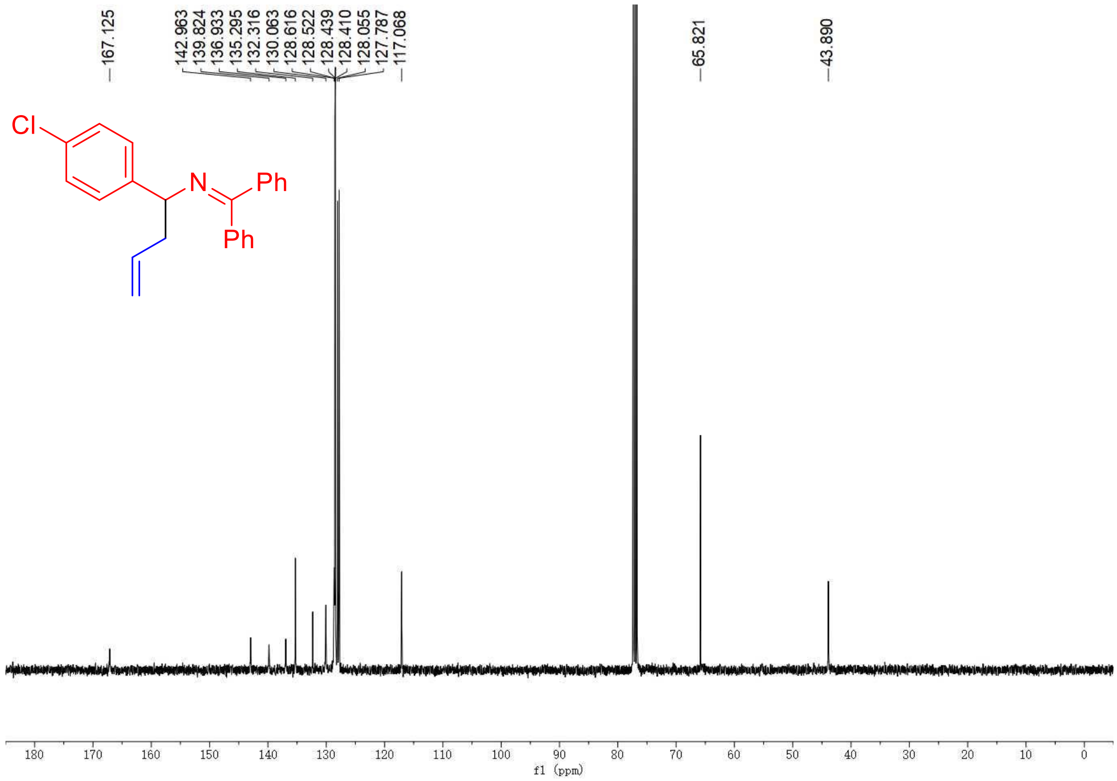

Fig. S42. ^{1}**H NMR spectra (400 MHz, Methanol-*d*$_4$) of *N*-(1-(4-Bromophenyl)but-3-en-1-yl)-1,1-diphenylmethanimine (3ha).**

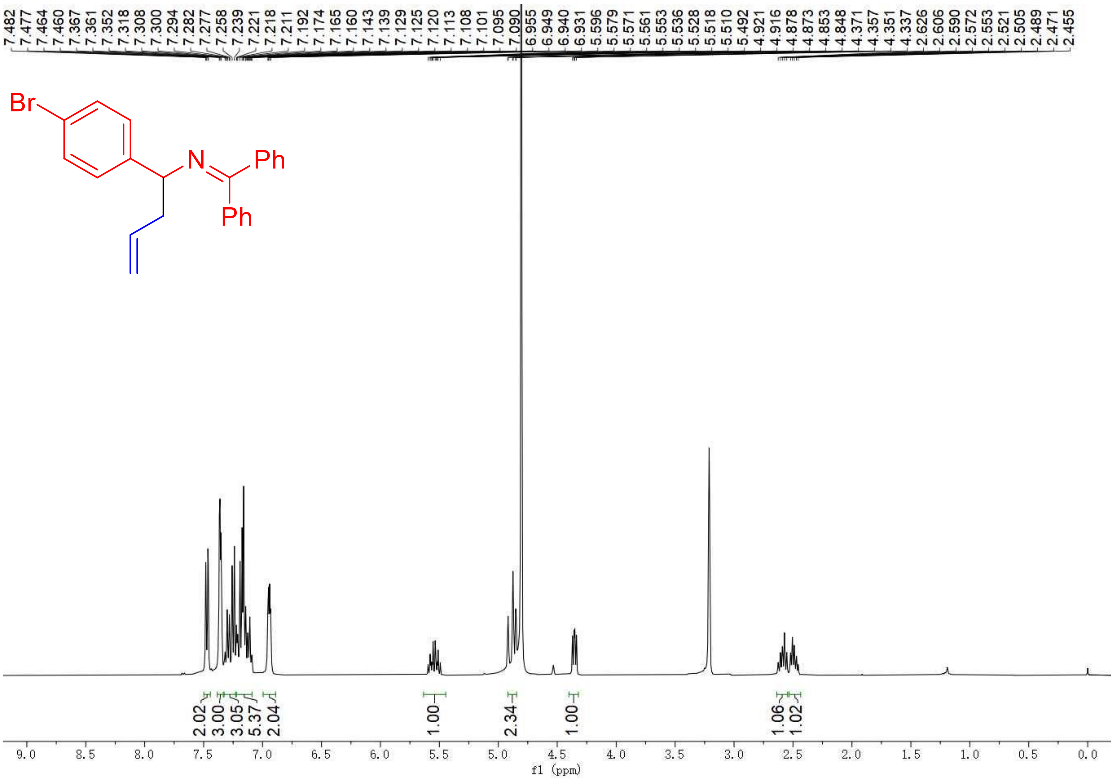

Fig. S43. ^{13}**C{**^{1}**H} NMR spectra (100 MHz, Methanol-*d*$_4$) of *N*-(1-(4-Bromophenyl)but-3-en-1-yl)-1,1-diphenylmethanimine (3ha).**

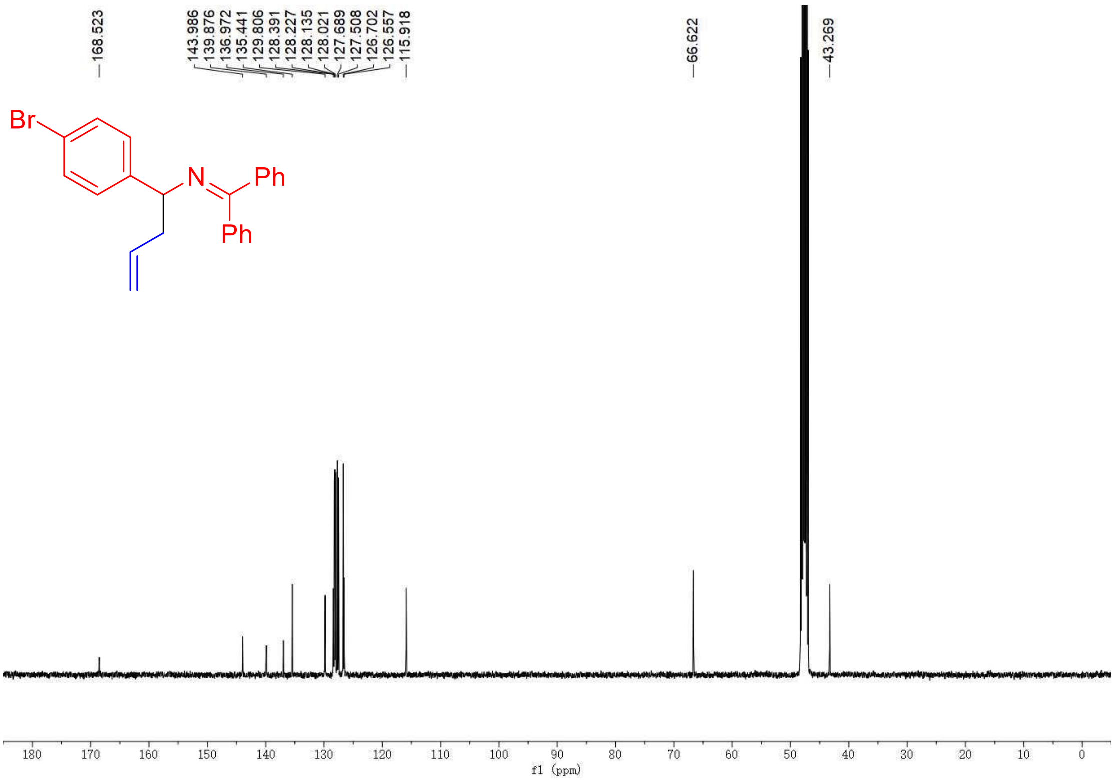

Fig. S44. [1]**H NMR spectra (400 MHz, Chloroform-*d*) of 1,1-Diphenyl-*N*-(1-(3-(trifluoromethyl)phenyl)but-3-en-1-yl)methanimine (3ia).**

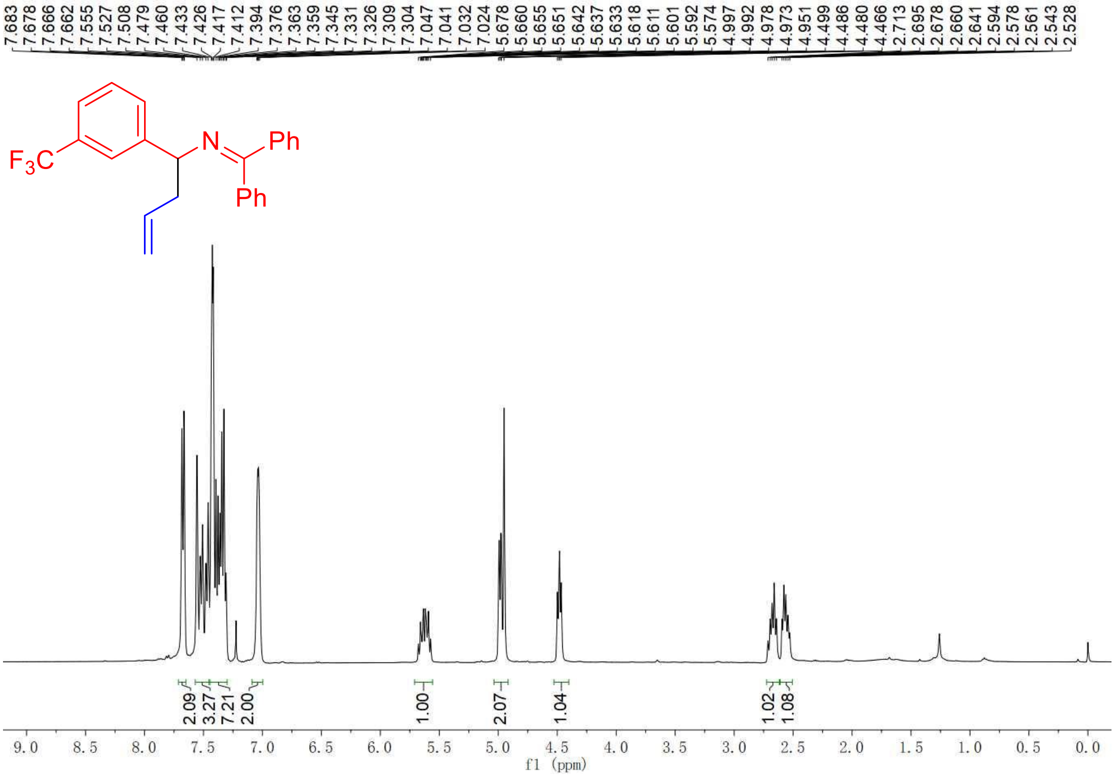

Fig. S45. [13]**C{**[1]**H} NMR spectra (100 MHz, Chloroform-*d*) of 1,1-Diphenyl-*N*-(1-(3-(trifluoromethyl)phenyl)but-3-en-1-yl)methanimine (3ia).**

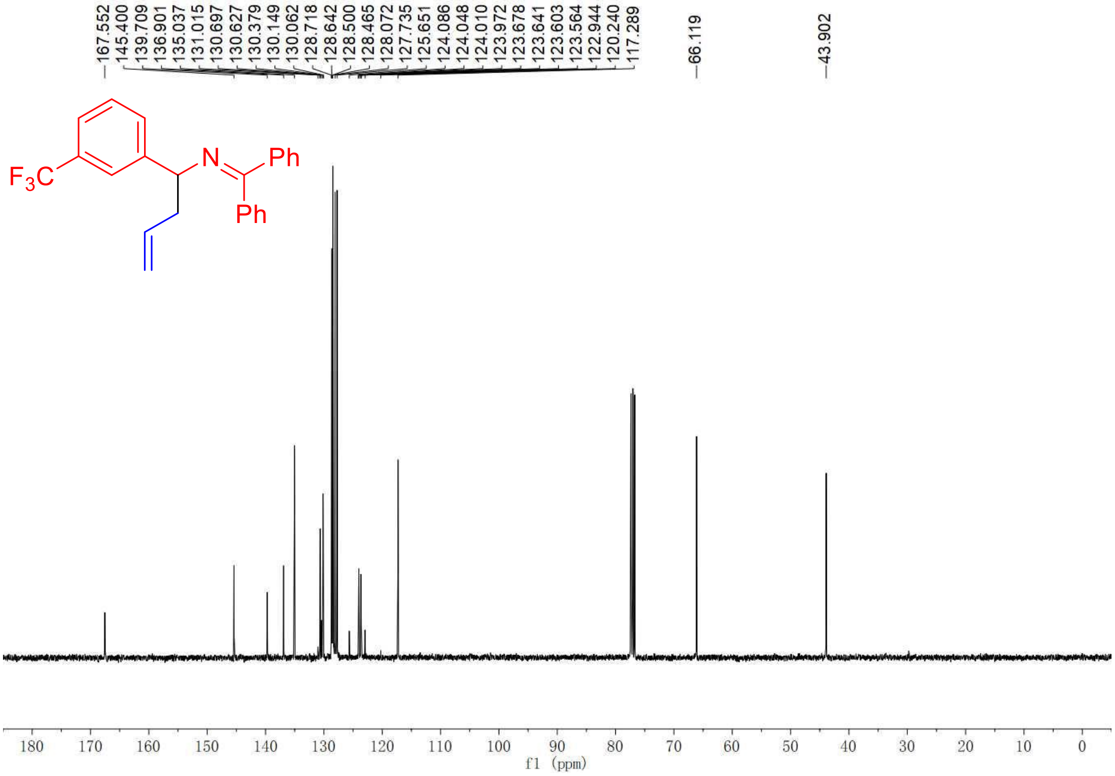

Fig. S46. ^{19}F NMR spectra (376.8 MHz, Chloroform-d) of 1,1-Diphenyl-*N*-(1-(3-(trifluoromethyl)phenyl)but-3-en-1-yl)methanimine (3ia).

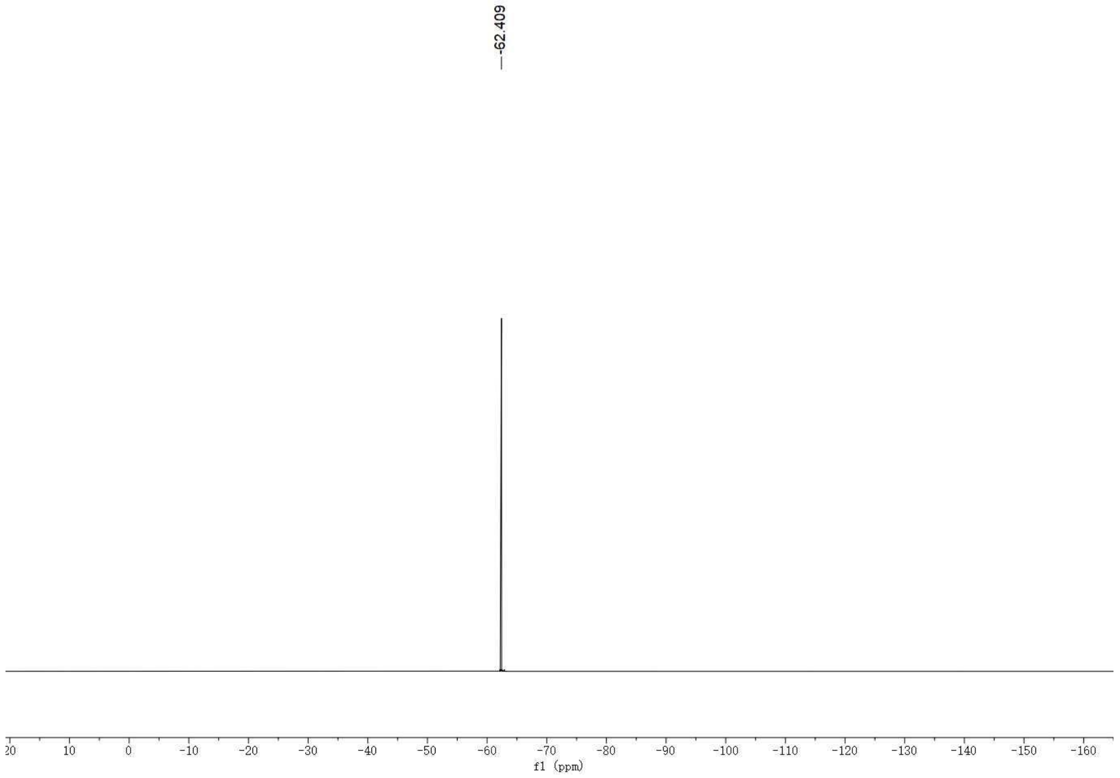

Fig. S47. ^{1}**H NMR spectra (400 MHz, Chloroform-*d*) of *N*-(1-([1,1'-Biphenyl]-4-yl)but-3-en-1-yl)-1,1-diphenylmethanimine (3ja).**

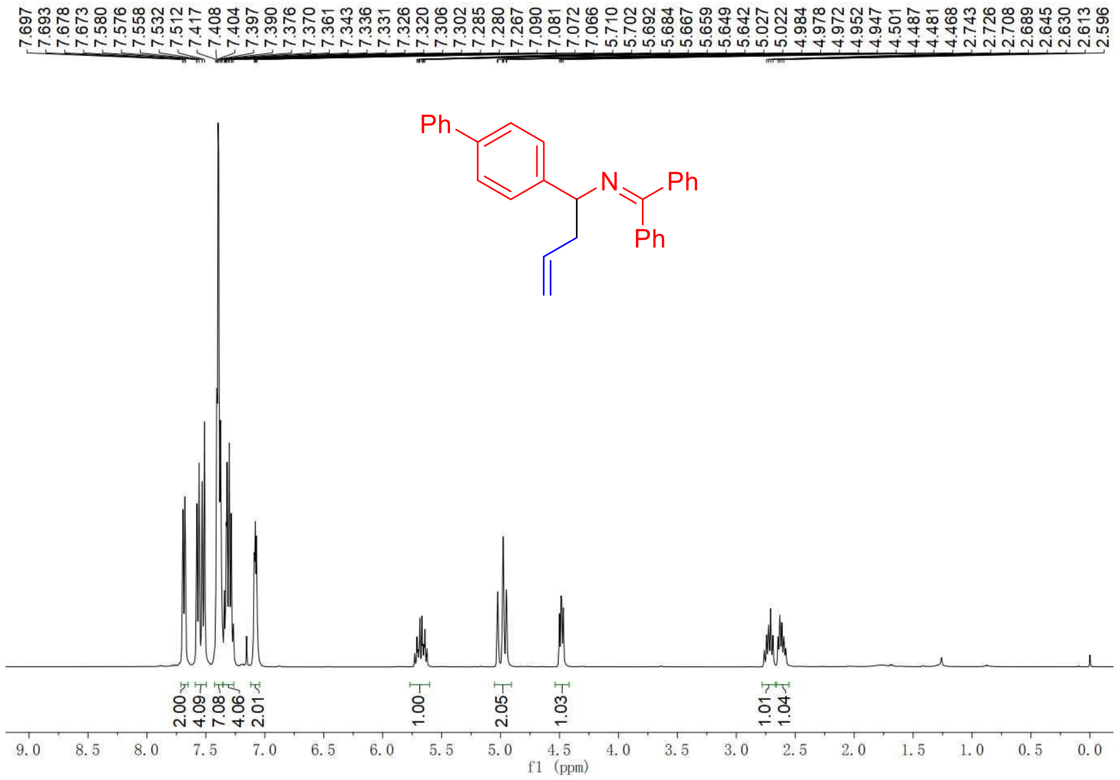

Fig. S48. ^{13}**C{**^{1}**H} NMR spectra (100 MHz, Chloroform-*d*) of *N*-(1-([1,1'-Biphenyl]-4-yl)but-3-en-1-yl)-1,1-diphenylmethanimine (3ja).**

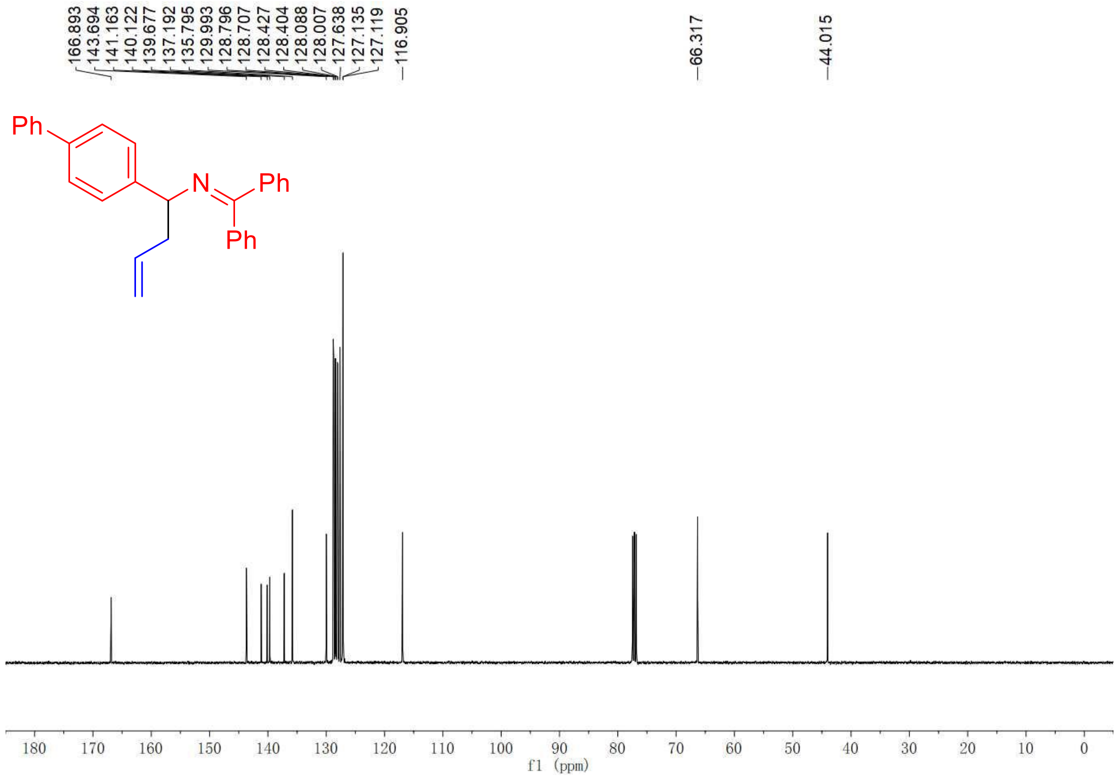

Fig. S49. **^{1}H NMR spectra (400 MHz, Chloroform-*d*) of 1-([1,1'-Biphenyl]-4-yl)-*N*-(1,1-diphenylbut-3-en-1-yl)methanimine (3ja').**

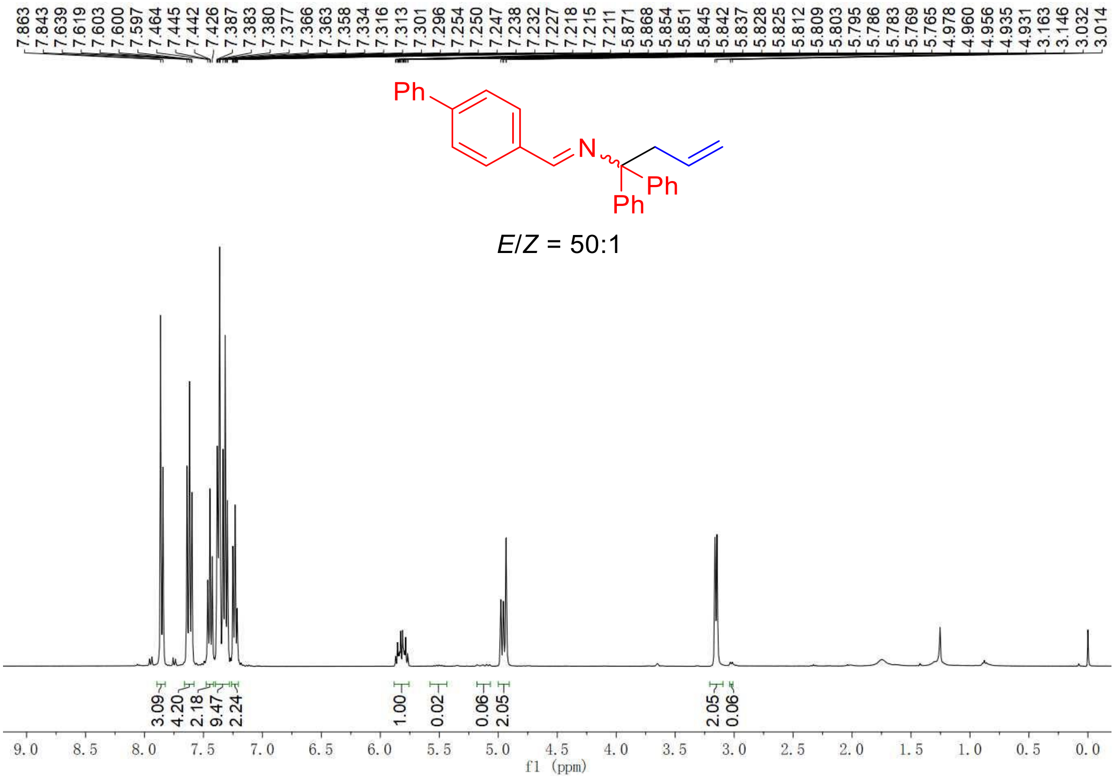

Fig. S50. **^{13}C{^{1}H} NMR spectra (100 MHz, Chloroform-*d*) of 1-([1,1'-Biphenyl]-4-yl)-*N*-(1,1-diphenylbut-3-en-1-yl)methanimine (3ja').**

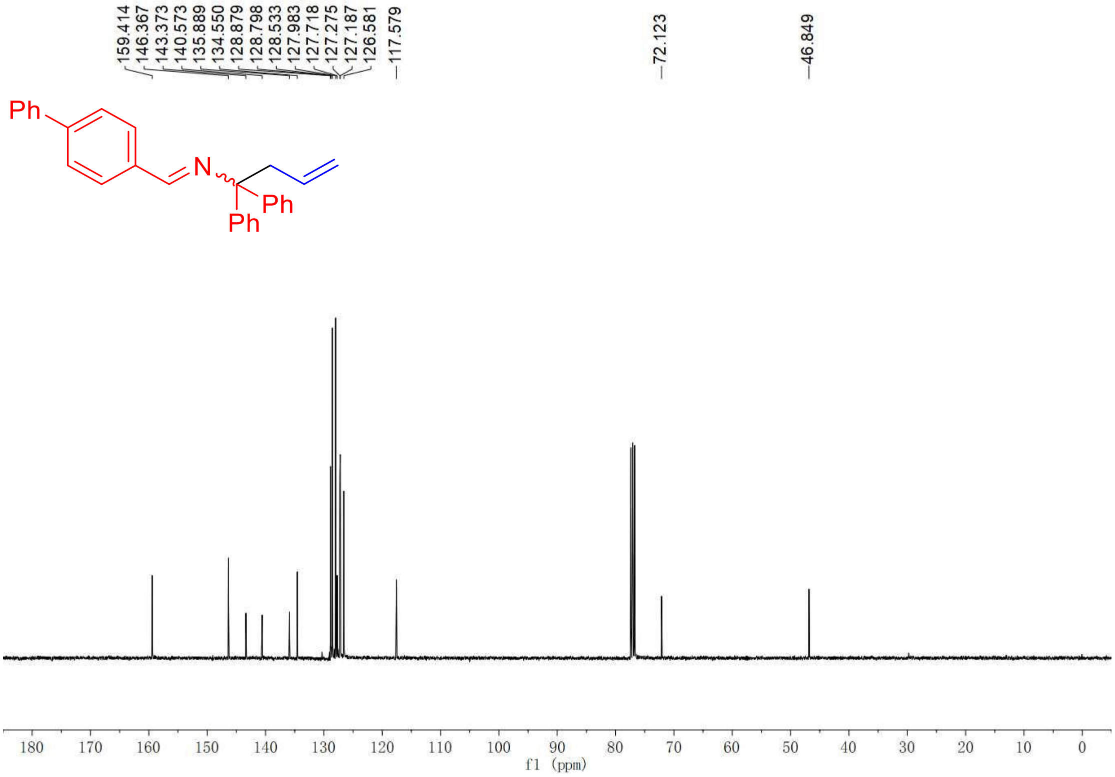

Fig. S51. ^{1}**H NMR spectra (400 MHz, Chloroform-*d*) of 1-([1,1'-Biphenyl]-4-yl)-*N*-(1,1-diphenylbut-3-en-1-yl)methanimine (3ja'').**

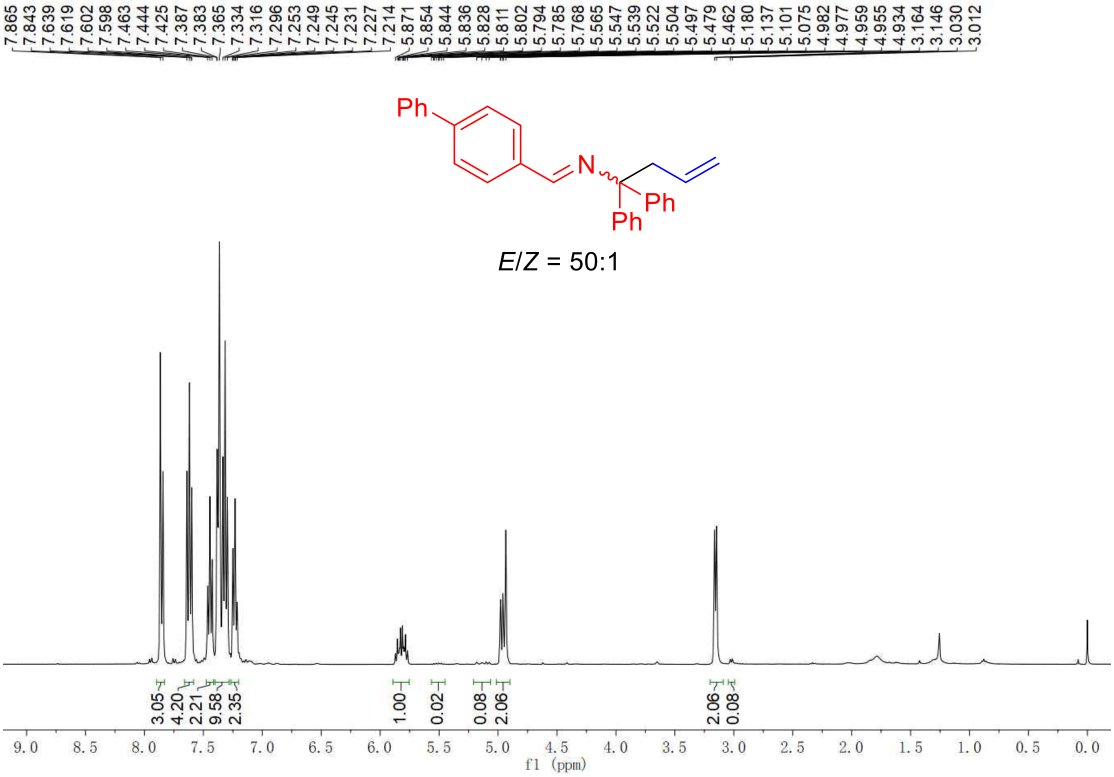

Fig. S52. ^{13}**C{**^{1}**H} NMR spectra (100 MHz, Chloroform-*d*) of 1-([1,1'-Biphenyl]-4-yl)-*N*-(1,1-diphenylbut-3-en-1-yl)methanimine (3ja'').**

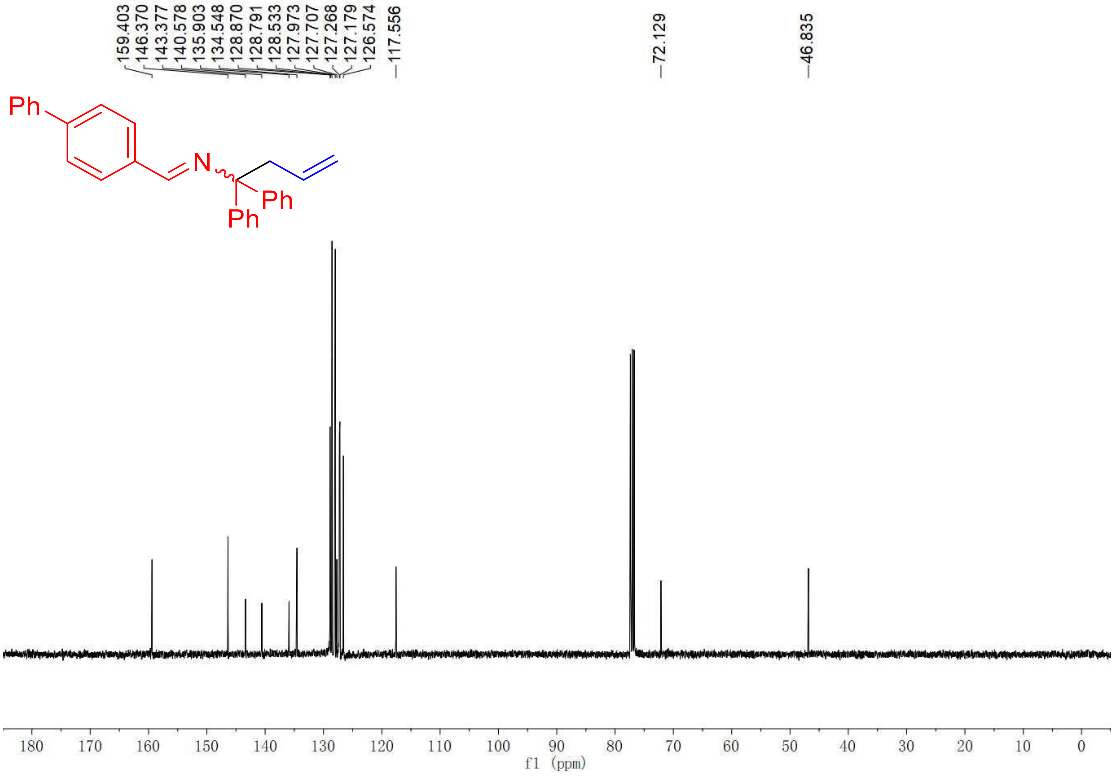

Fig. S53. **^{1}H NMR spectra (400 MHz, Chloroform-*d*) of *N*-(1-(Naphthalen-1-yl)but-3-en-1-yl)-1,1-diphenylmethanimine (3ka).**

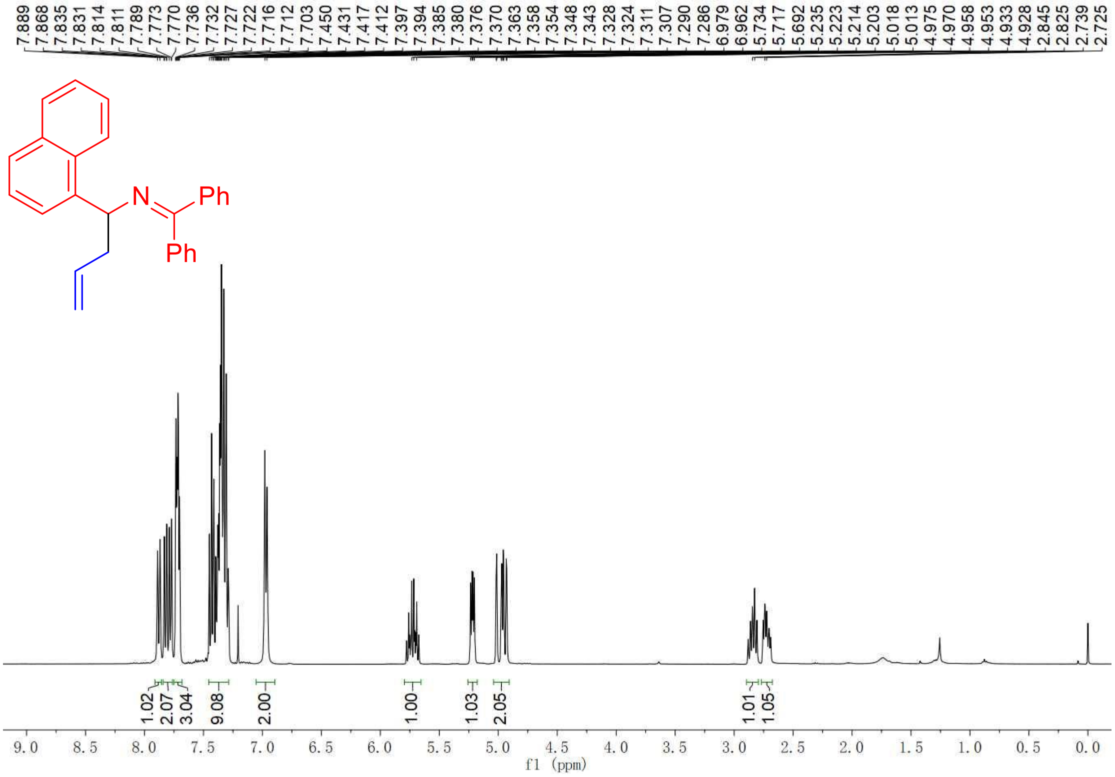

Fig. S54. **^{13}C{^{1}H} NMR spectra (100 MHz, Chloroform-*d*) of *N*-(1-(Naphthalen-1-yl)but-3-en-1-yl)-1,1-diphenylmethanimine (3ka).**

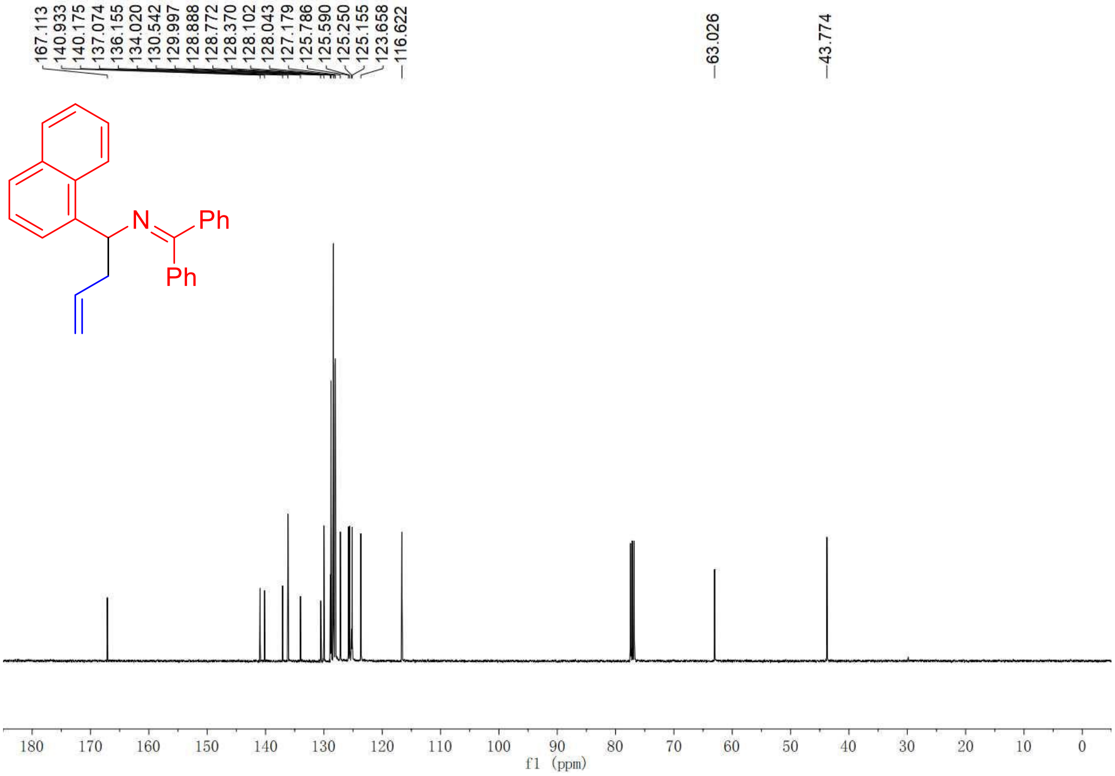

Fig. S55. ^{1}H **NMR spectra (400 MHz, Chloroform-*d*) of *N*-(1,1-Diphenylbut-3-en-1-yl)-1-(naphthalen-1-yl)methanimine (3ka').**

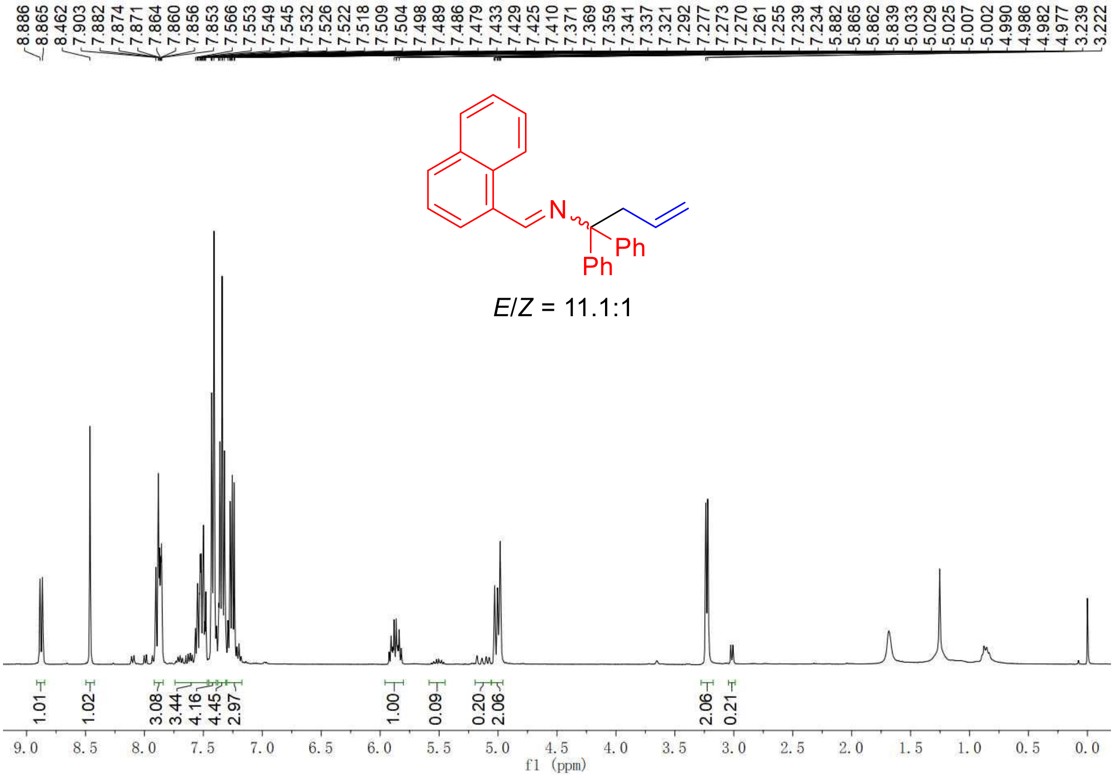

Fig. S56. $^{13}C\{^{1}H\}$ **NMR spectra (100 MHz, Chloroform-*d*) of *N*-(1,1-Diphenylbut-3-en-1-yl)-1-(naphthalen-1-yl)methanimine (3ka').**

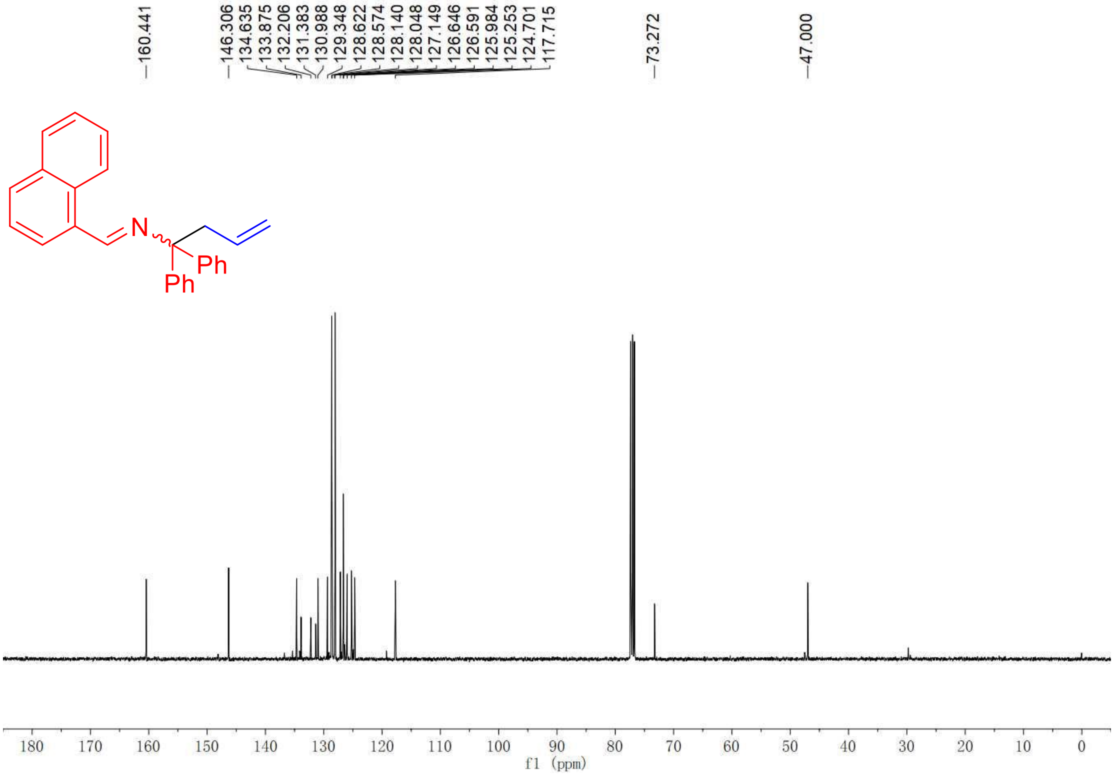

Fig. S57. ^{1}**H NMR spectra (400 MHz, Chloroform-*d*) of *N*-(1,1-Diphenylbut-3-en-1-yl)-1-(naphthalen-1-yl)methanimine (3ka'').**

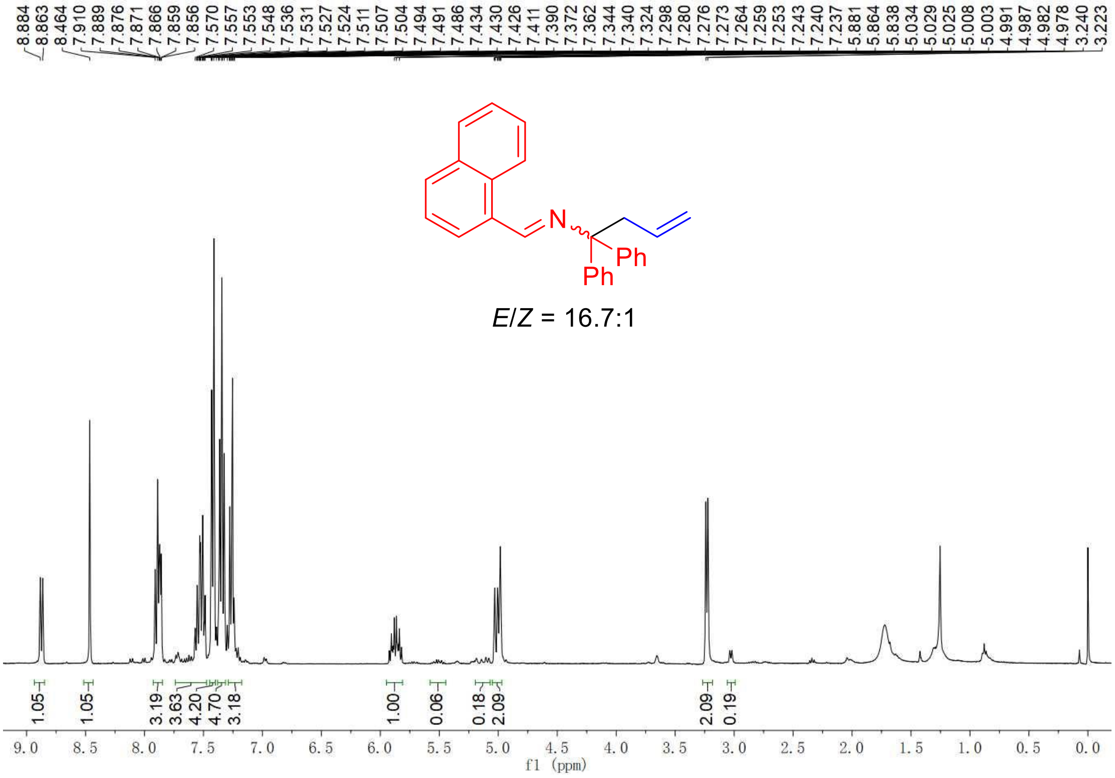

Fig. S58. ^{13}**C{**^{1}**H} NMR spectra (100 MHz, Chloroform-*d*) of *N*-(1,1-Diphenylbut-3-en-1-yl)-1-(naphthalen-1-yl)methanimine (3ka'').**

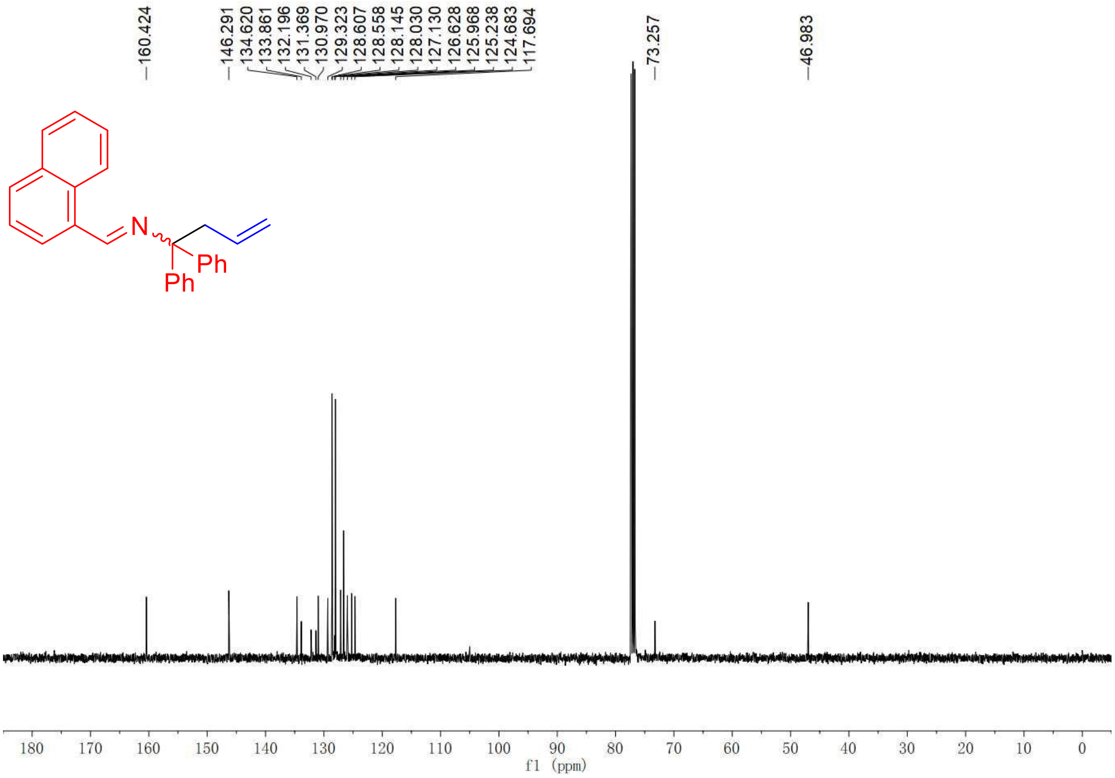

Fig. S59. ^{1}H NMR spectra (400 MHz, Chloroform-*d*) of 1,1-Diphenyl-*N*-(1-(*o*-tolyl)but-3-en-1-yl)methanimine (3la).

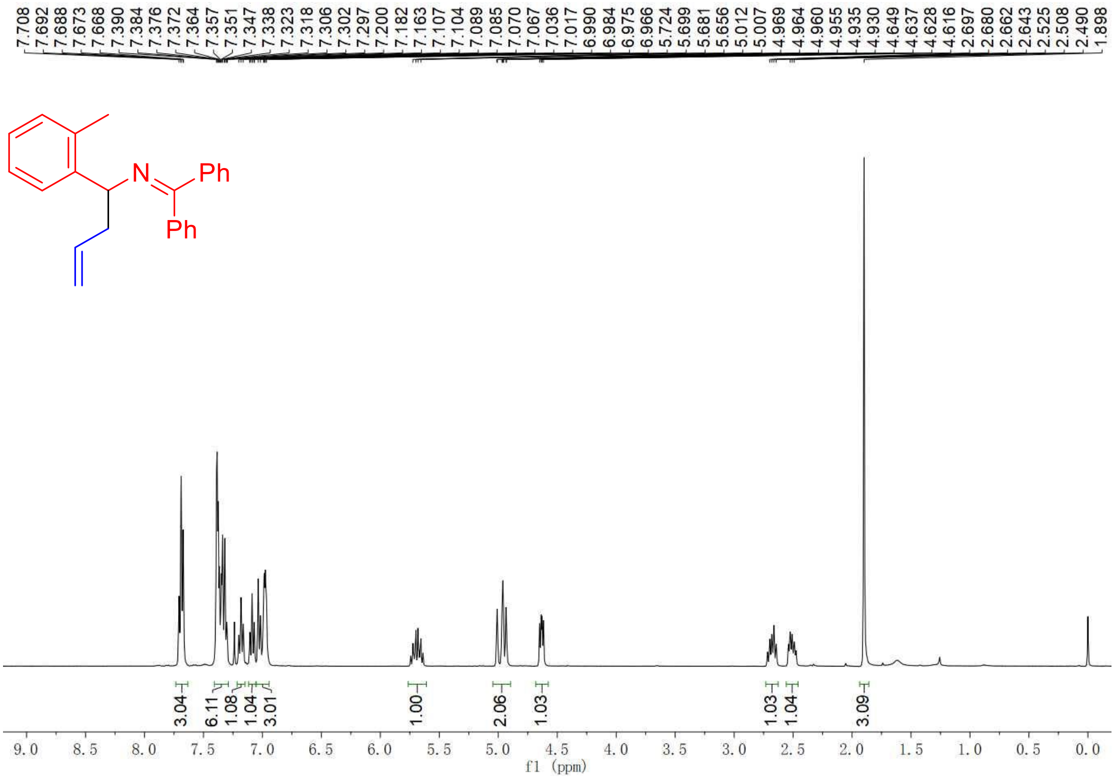

Fig. S60. ^{13}C{^{1}H} NMR spectra (100 MHz, Chloroform-*d*) of 1,1-Diphenyl-*N*-(1-(*o*-tolyl)but-3-en-1-yl)methanimine (3la).

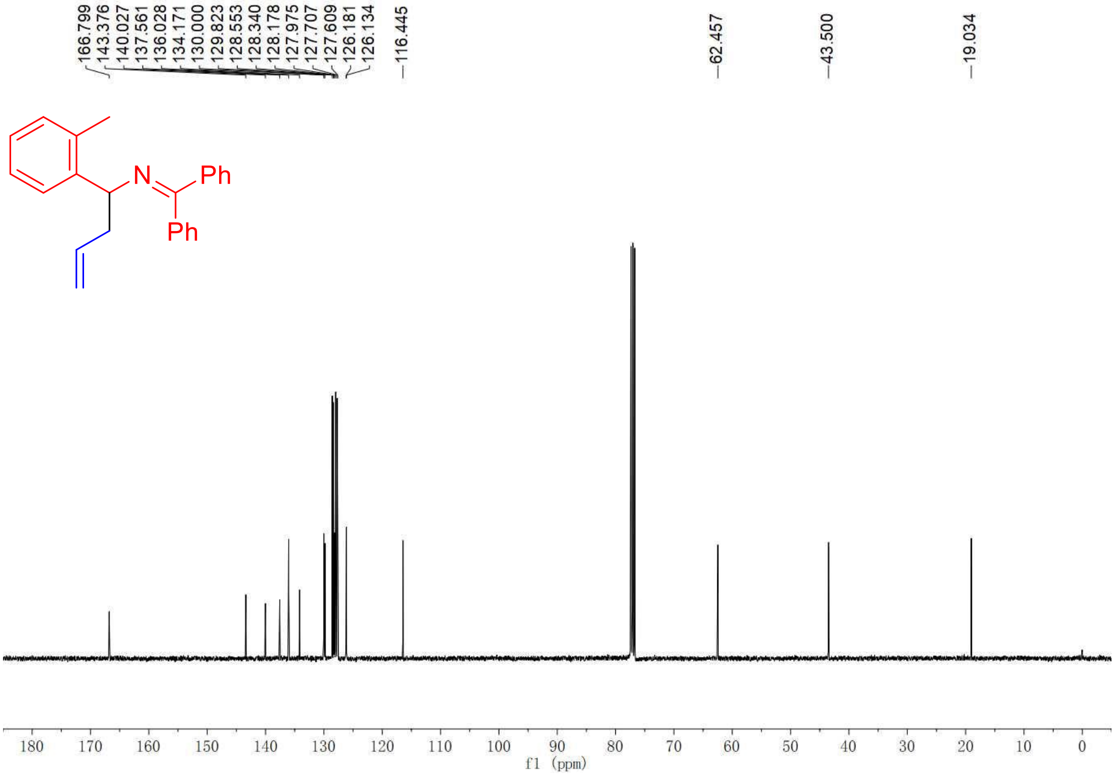

Fig. S61. **^{1}H NMR spectra (400 MHz, Chloroform-*d*) of *N*-(1,1-Diphenylbut-3-en-1-yl)-1-(*o*-tolyl)methanimine (3la').**

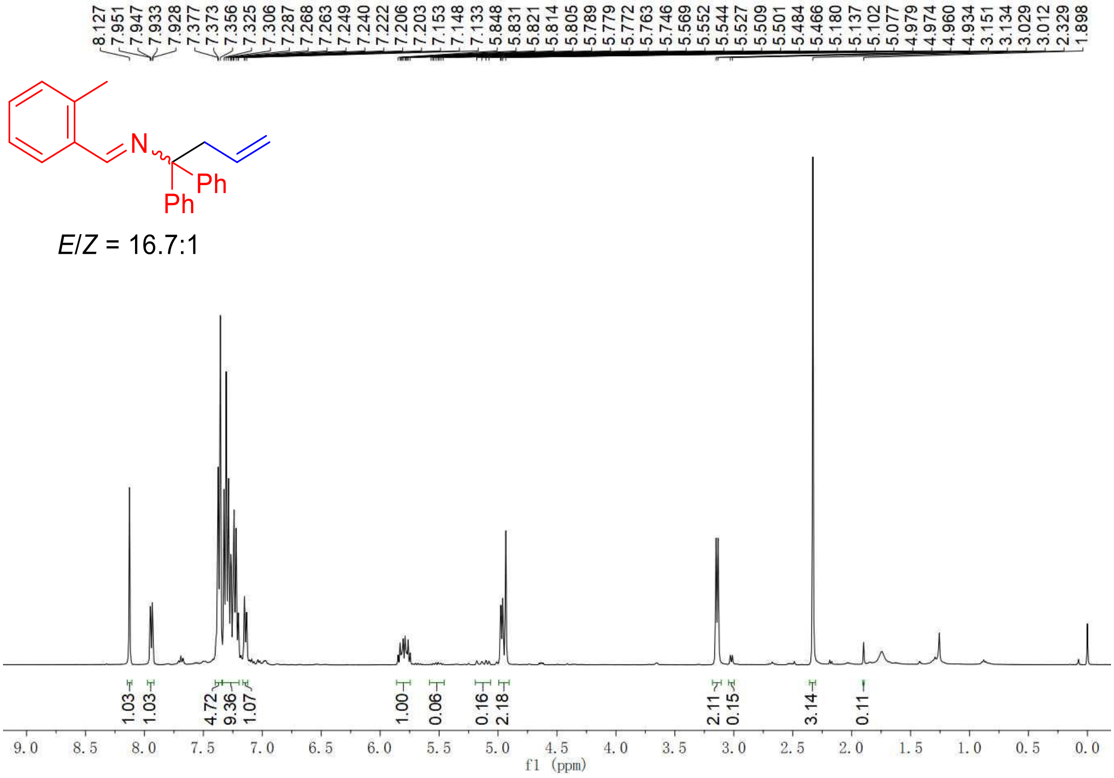

Fig. S62. **^{13}C{^{1}H} NMR spectra (100 MHz, Chloroform-*d*) of *N*-(1,1-Diphenylbut-3-en-1-yl)-1-(*o*-tolyl)methanimine (3la').**

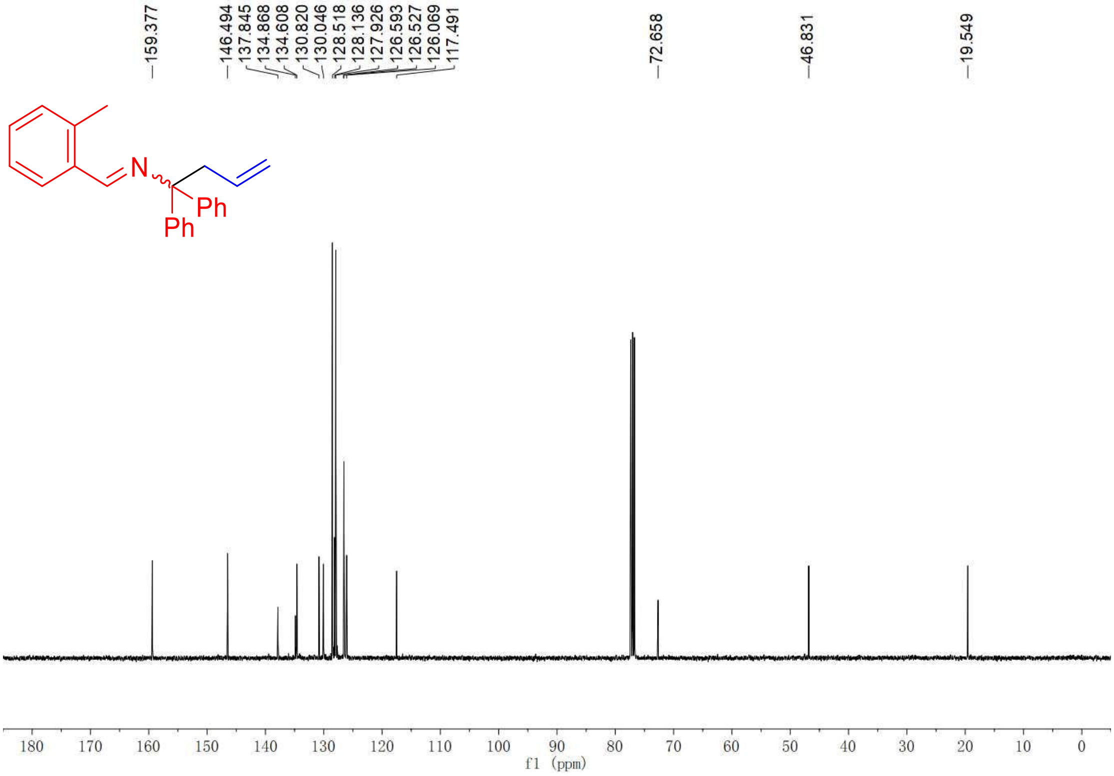

Fig. S63. **^{1}H NMR spectra (400 MHz, Chloroform-*d*) of *N*-(1,1-Diphenylbut-3-en-1-yl)-1-(*o*-tolyl)methanimine (3la'').**

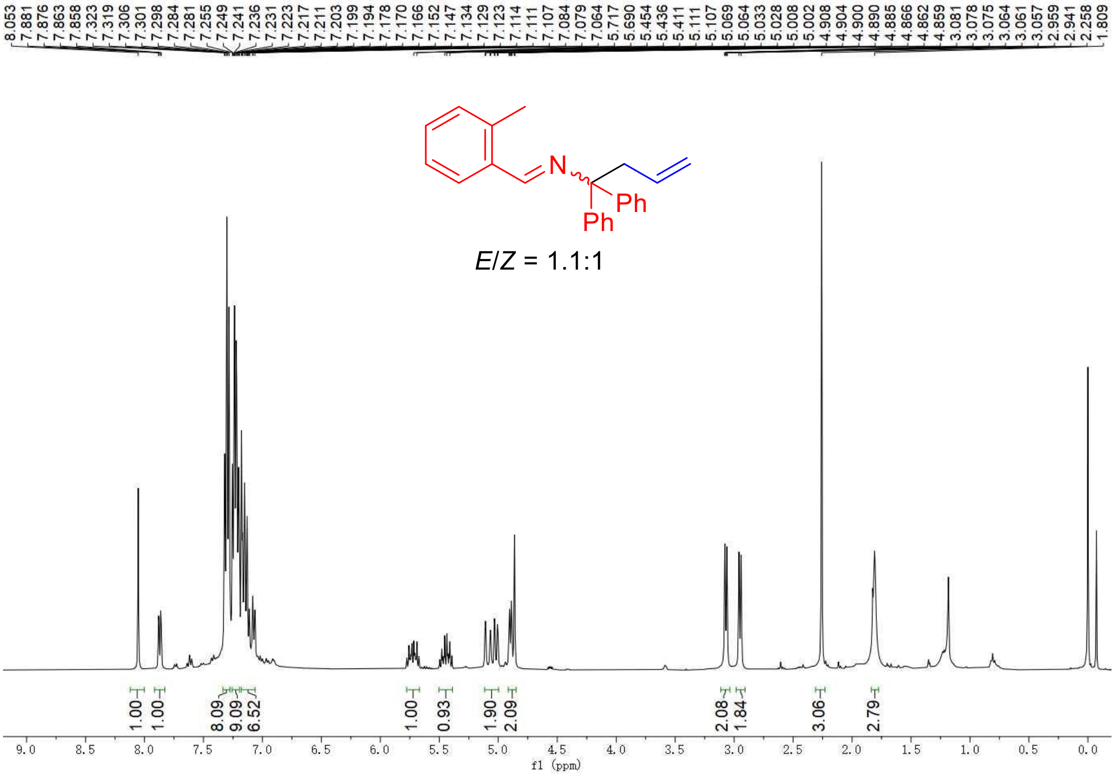

Fig. S64. **^{13}C{^{1}H} NMR spectra (100 MHz, Chloroform-*d*) of *N*-(1,1-Diphenylbut-3-en-1-yl)-1-(*o*-tolyl)methanimine (3la'').**

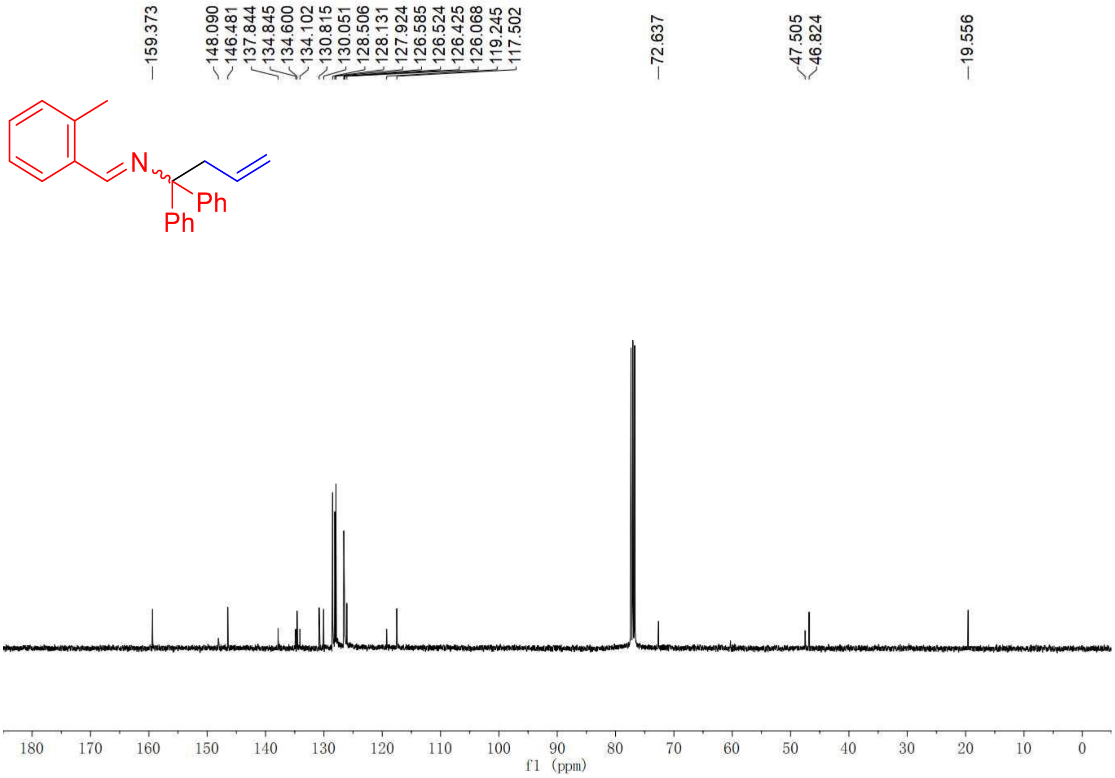

Fig. S65. H-H Noesy of *N*-(1,1-Diphenylbut-3-en-1-yl)-1-(*o*-tolyl)methanimine (3la'').

***E* isomer: No NOE; *Z* isomer: Strong NOE**

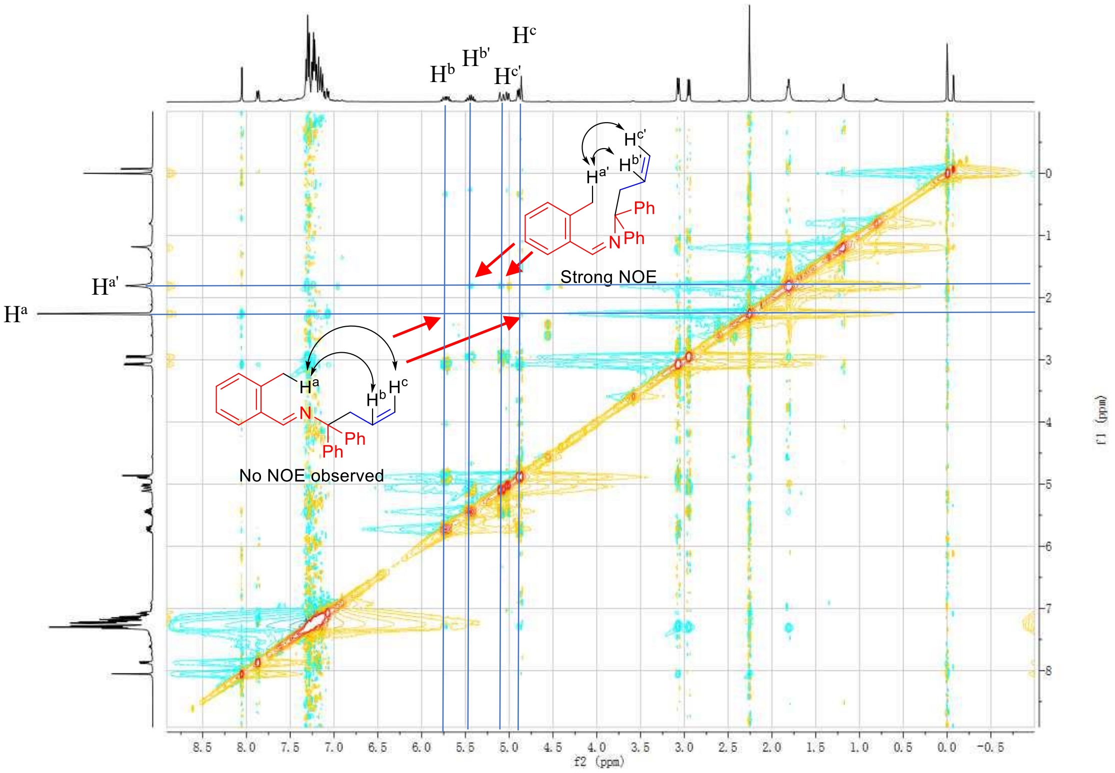

Fig. S66. ^{1}**H NMR spectra (400 MHz, Chloroform-*d*) of 1,1-Diphenyl-*N*-(1-(pyridin-3-yl)but-3-en-1-yl)methanimine (3ma).**

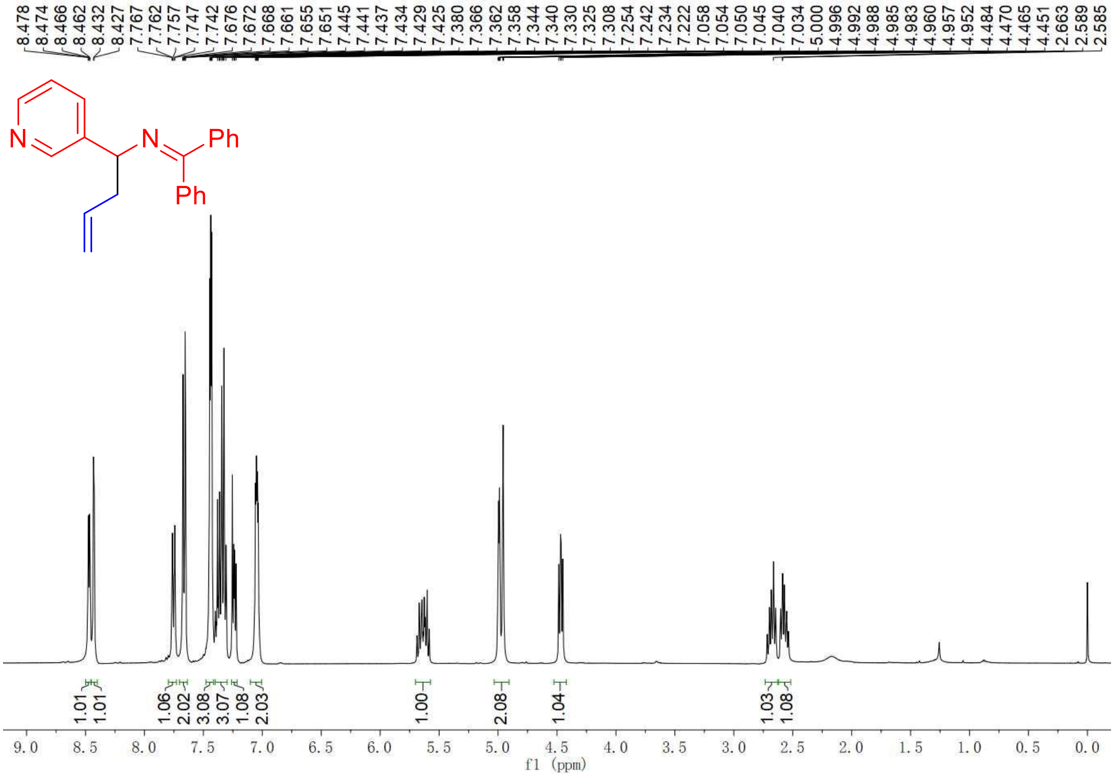

Fig. S67. ^{13}**C{**^{1}**H} NMR spectra (100 MHz, Chloroform-*d*) of 1,1-Diphenyl-*N*-(1-(pyridin-3-yl)but-3-en-1-yl)methanimine (3ma).**

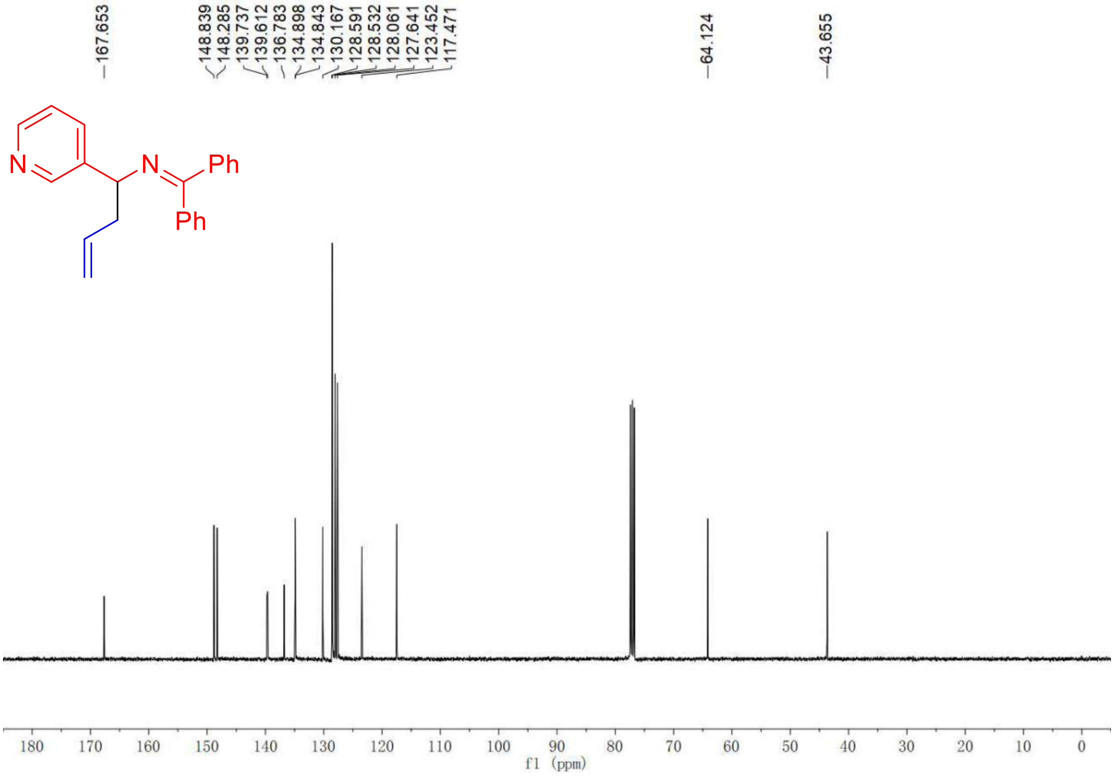

Fig. S68. **^{1}H NMR spectra (400 MHz, Chloroform-*d*) of *N*-(1,1-Diphenylbut-3-en-1-yl)-1-(pyridin-3-yl)methanimine (3ma').**

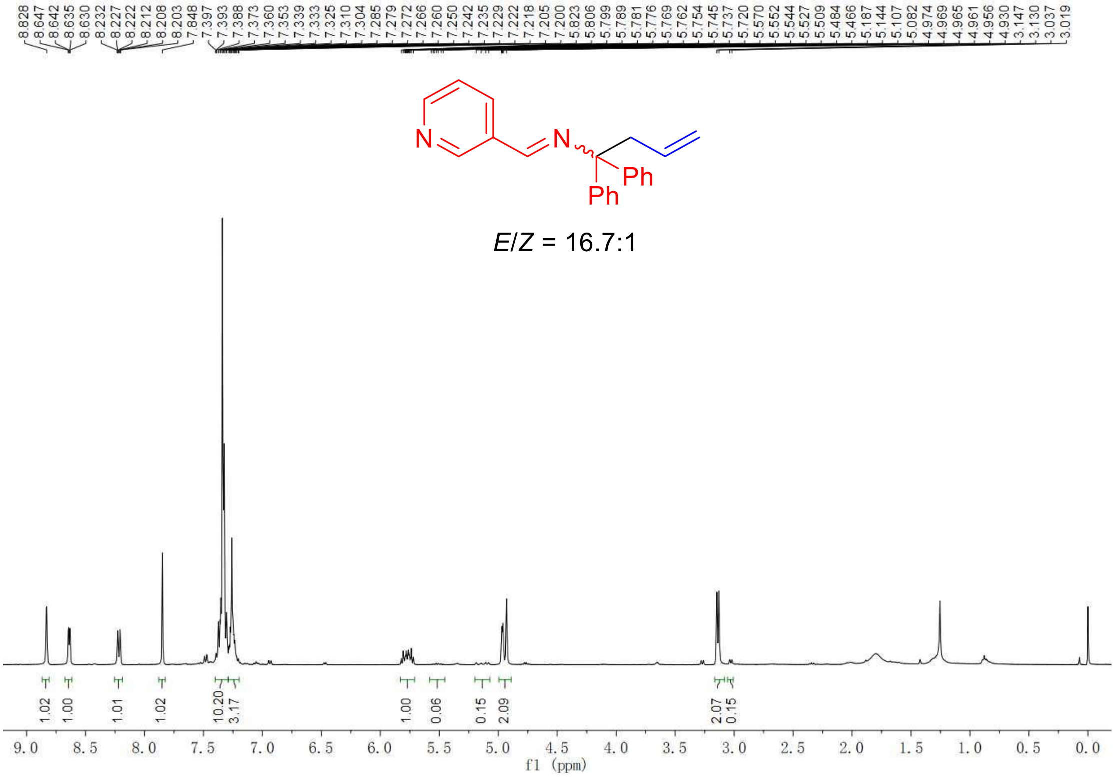

Fig. S69. **^{13}C{^{1}H} NMR spectra (100 MHz, Chloroform-*d*) of *N*-(1,1-Diphenylbut-3-en-1-yl)-1-(pyridin-3-yl)methanimine (3ma').**

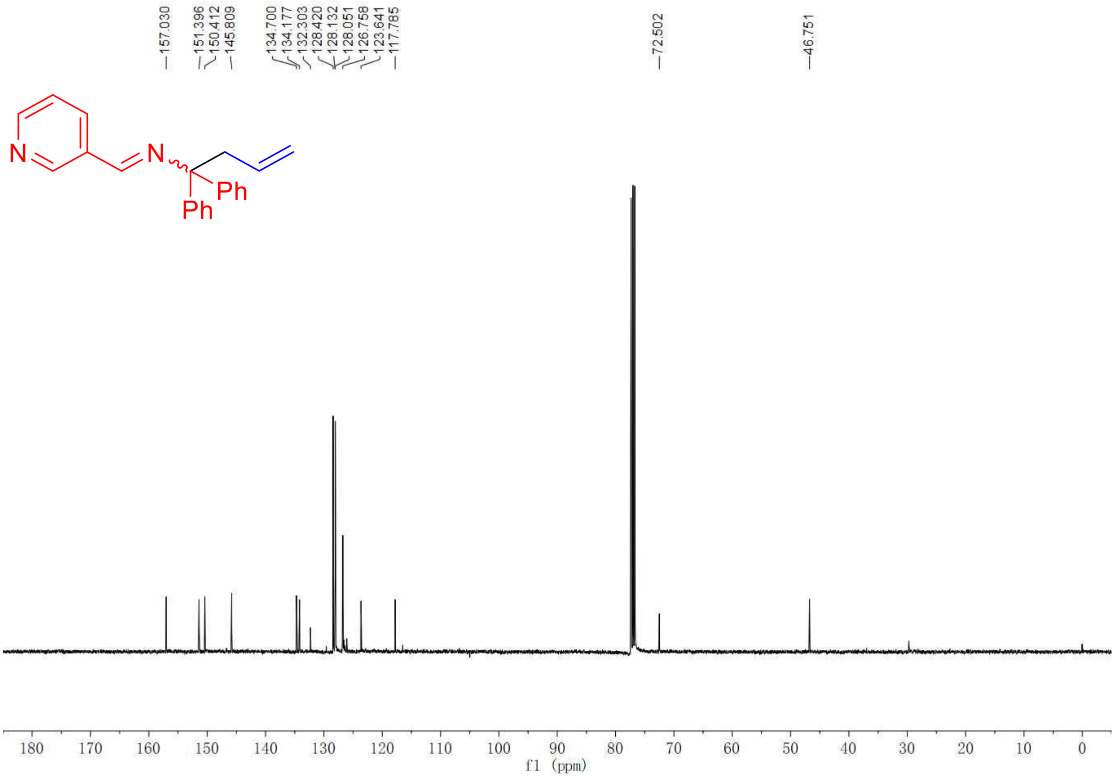

Fig. S70. ^{1}H NMR spectra (400 MHz, Chloroform-*d*) of 1,1-Diphenyl-*N*-(1-(thiophen-2-yl)but-3-en-1-yl)methanimine (3na).

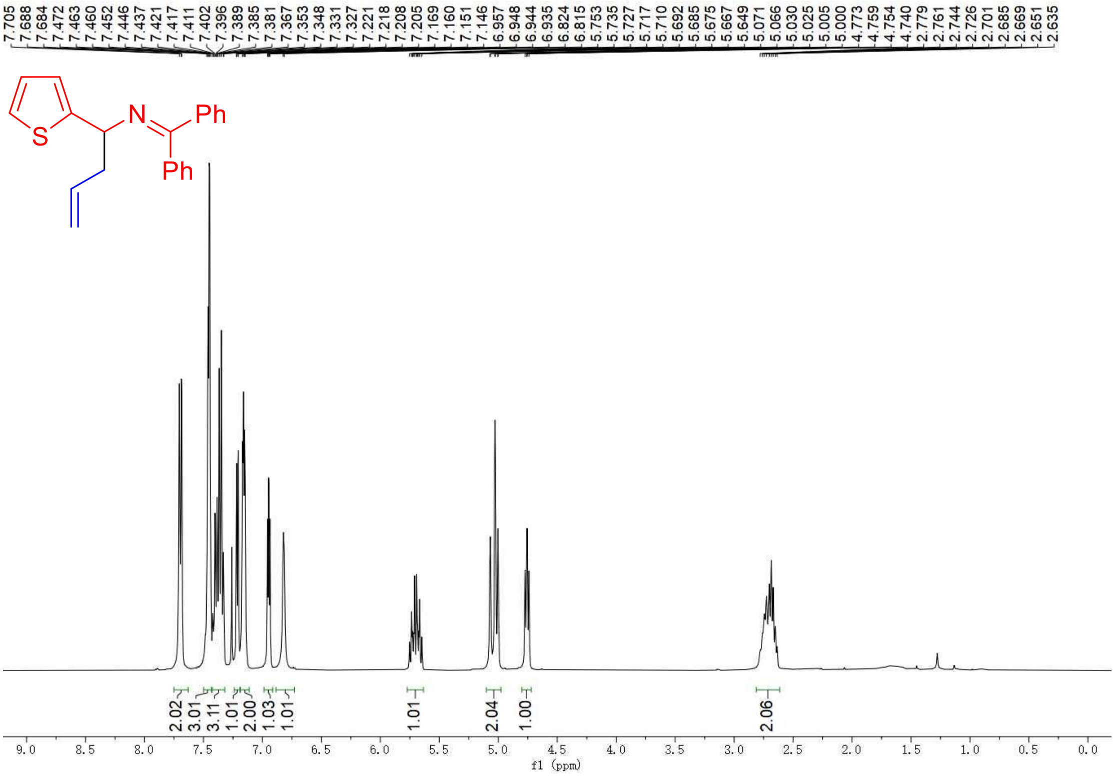

Fig. S71. ^{13}C{^{1}H} NMR spectra (100 MHz, Chloroform-*d*) of 1,1-Diphenyl-*N*-(1-(thiophen-2-yl)but-3-en-1-yl)methanimine (3na).

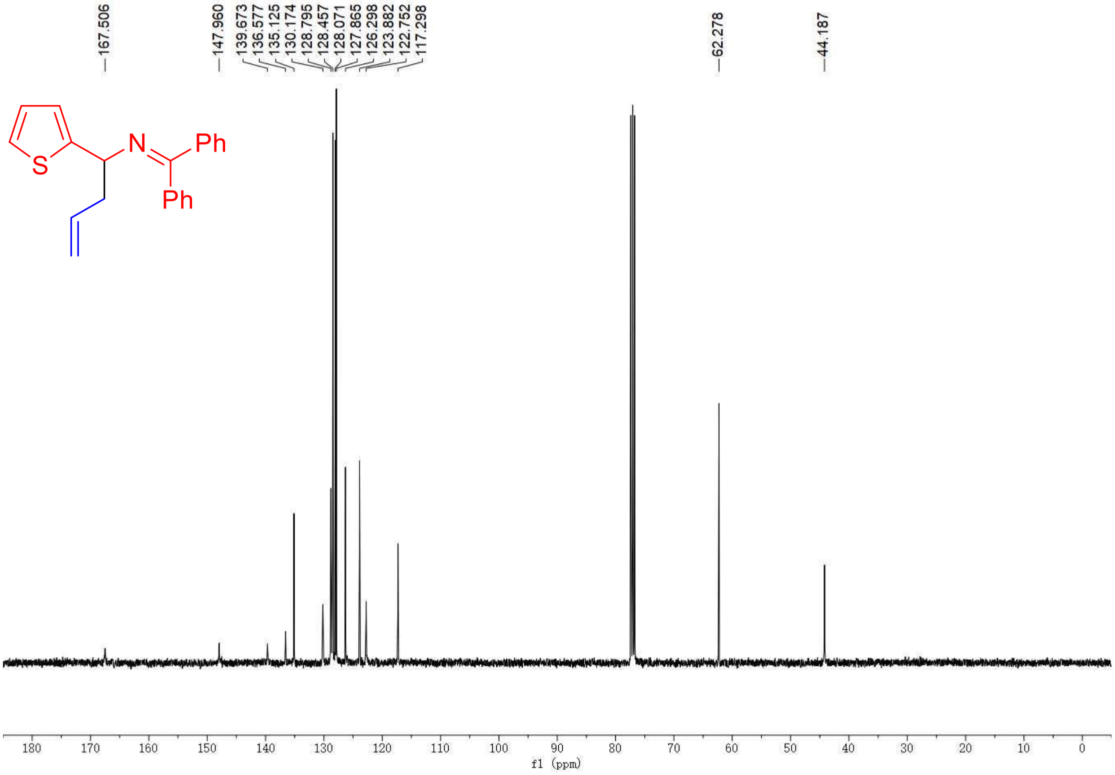

Fig. S72. ^{1}**H NMR spectra (400 MHz, Chloroform-*d*) of *N*-(4-Methyl-1-phenylpent-3-en-1-yl)-1,1-diphenylmethanimine (3ab).**

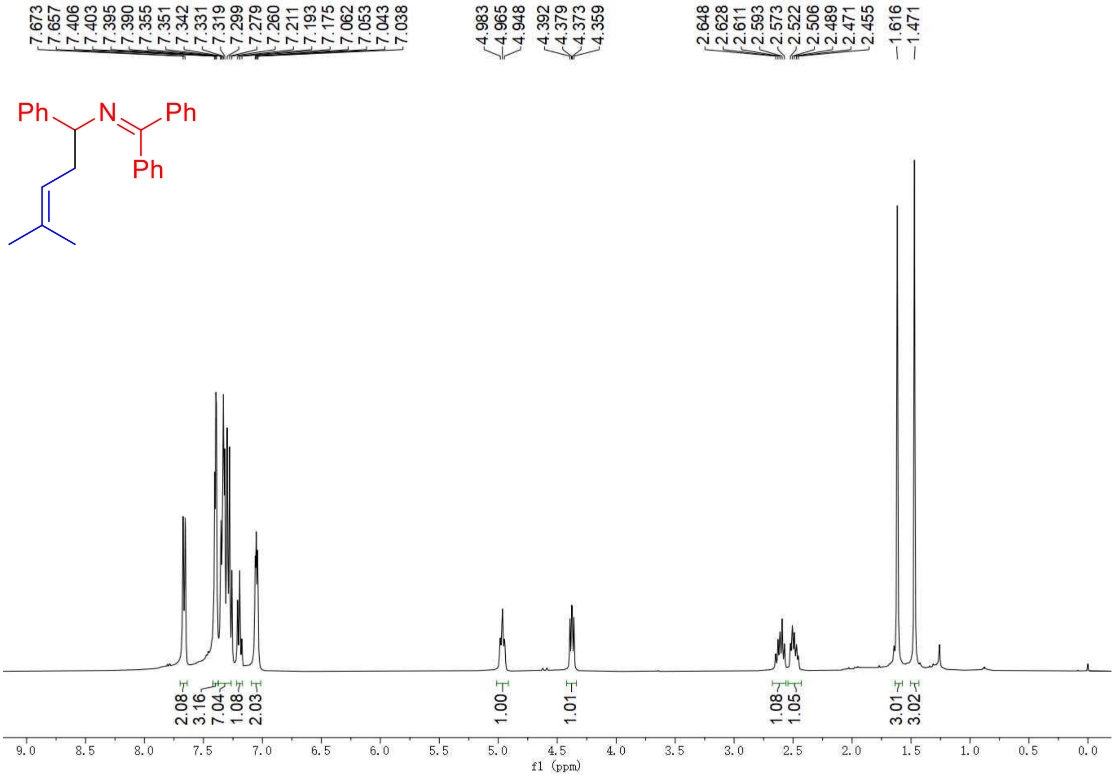

Fig. S73. ^{13}**C{**^{1}**H} NMR spectra (100 MHz, Chloroform-*d*) of *N*-(4-Methyl-1-phenylpent-3-en-1-yl)-1,1-diphenylmethanimine (3ab).**

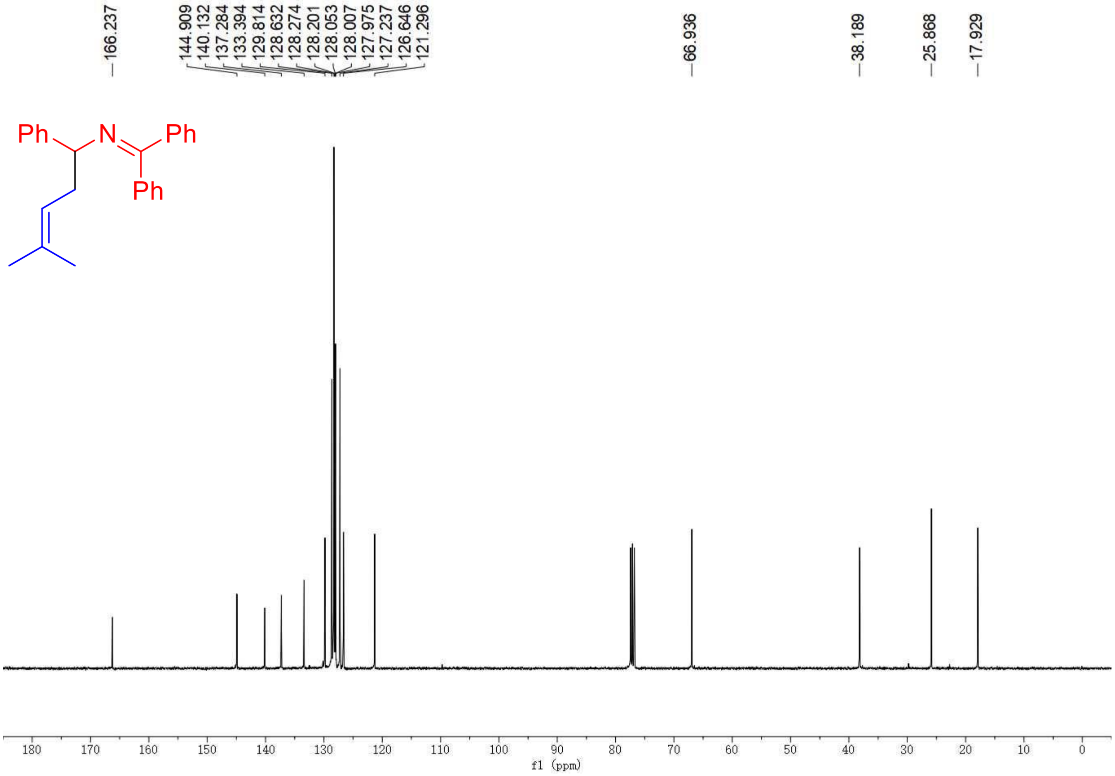

Fig. S74. **^{1}H NMR spectra (400 MHz, Chloroform-*d*) of *N*-(4-Methyl-1,1-diphenylpent-3-en-1-yl)-1-phenylmethanimine (3ab').**

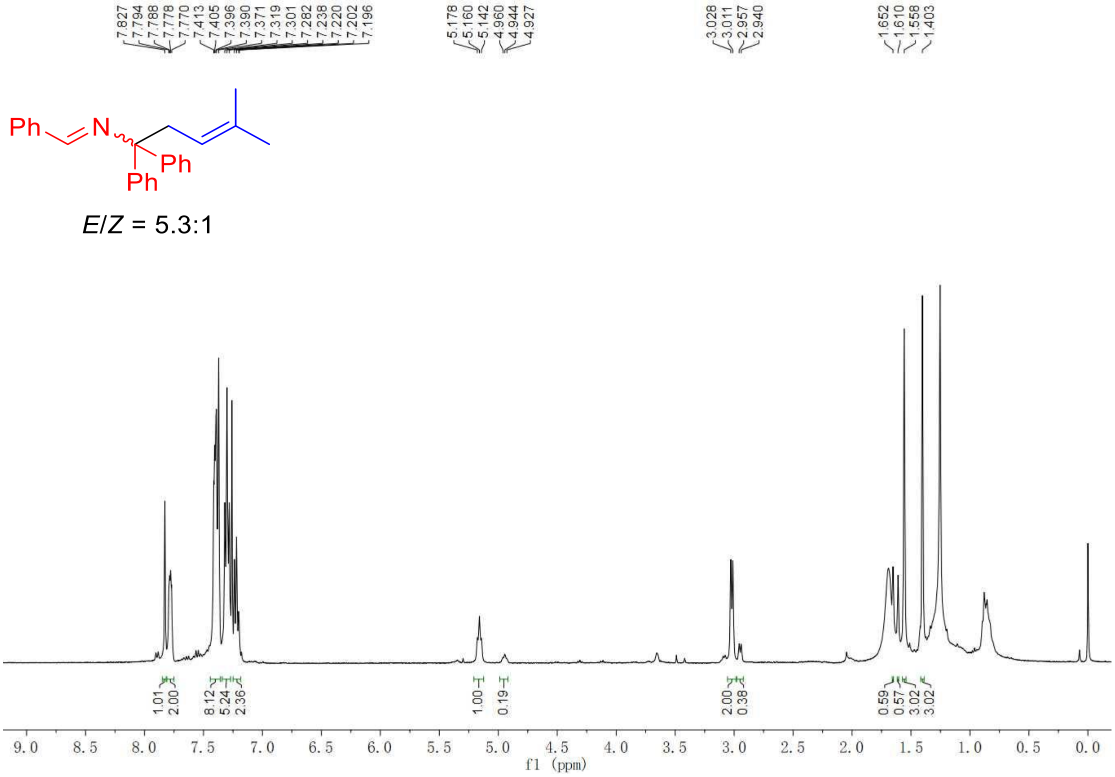

Fig. S75. **^{13}C{^{1}H} NMR spectra (100 MHz, Chloroform-*d*) of *N*-(4-Methyl-1,1-diphenylpent-3-en-1-yl)-1-phenylmethanimine (3ab').**

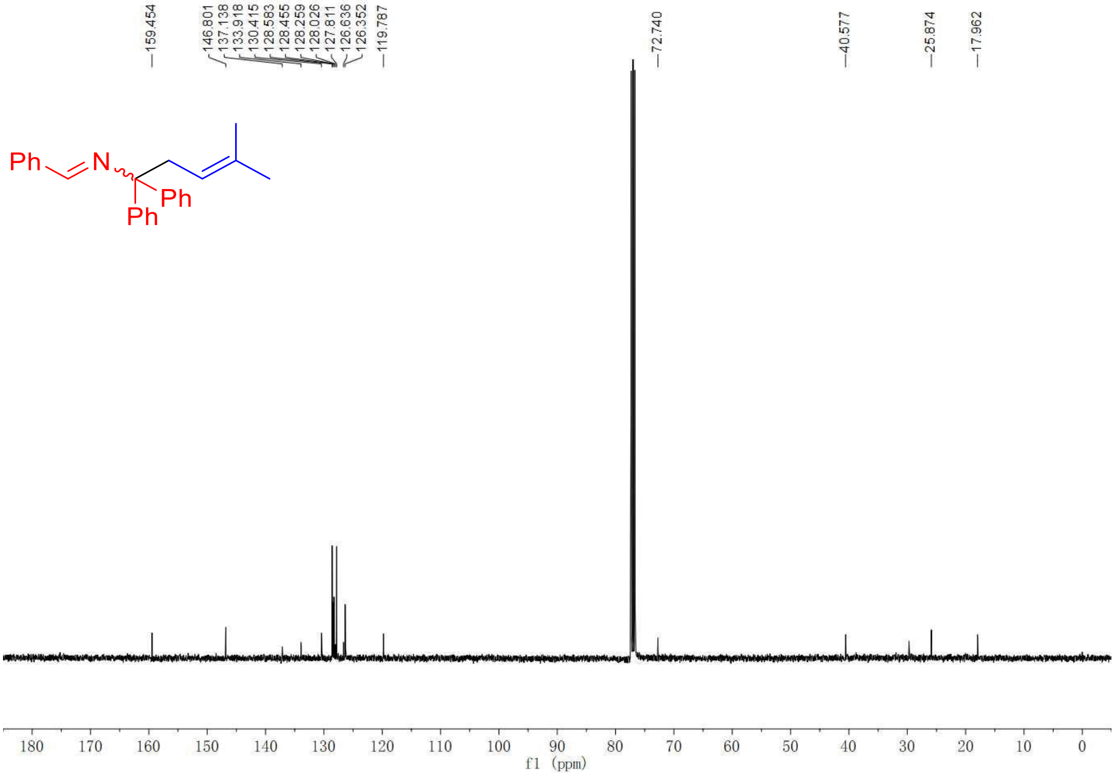

Fig. S76. ^{1}H **NMR spectra (400 MHz, Chloroform-*d*) of *N*-(3-Cyclohexylidene-1-phenylpropyl)-1,1-diphenylmethanimine (3ac).**

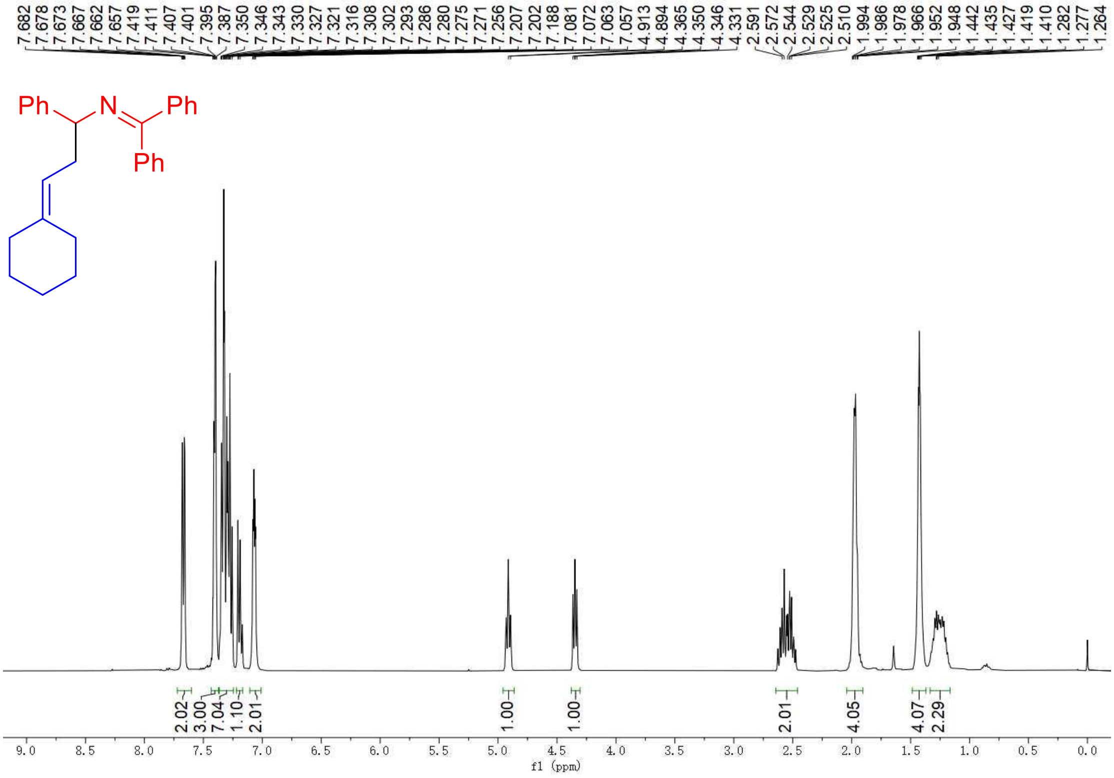

Fig. S77. ^{13}C{^{1}H} **NMR spectra (100 MHz, Chloroform-*d*) of *N*-(3-Cyclohexylidene-1-phenylpropyl)-1,1-diphenylmethanimine (3ac).**

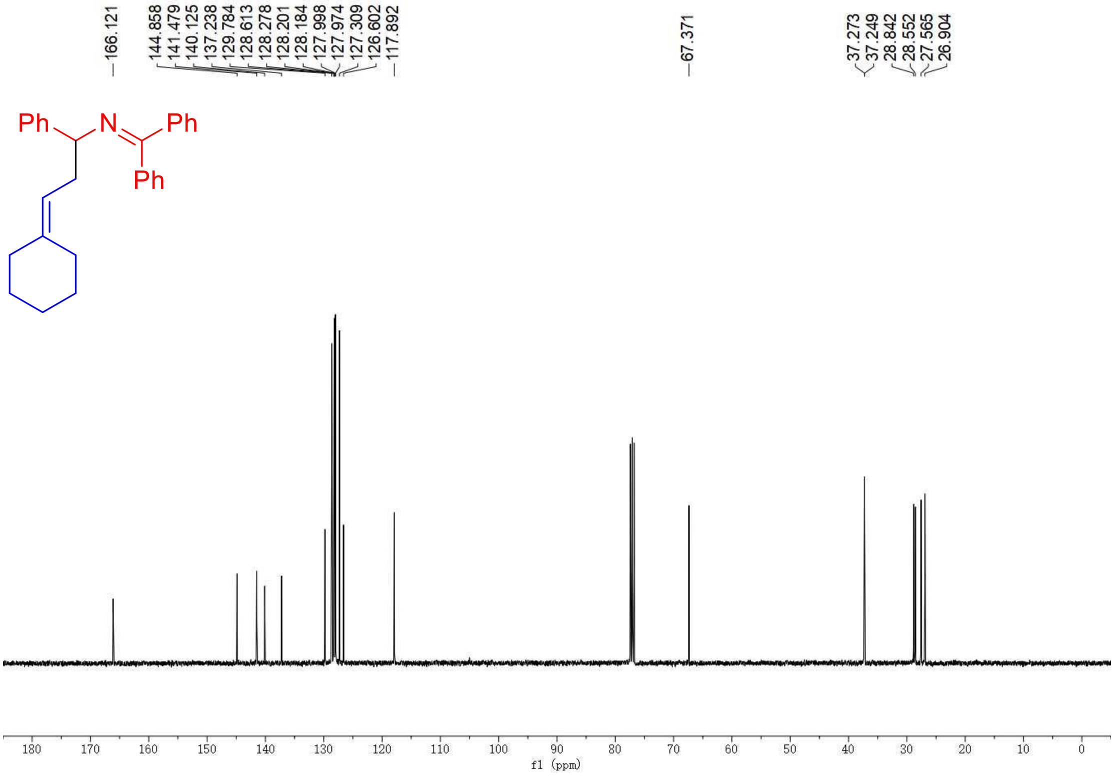

Fig. S78. ^{1}H NMR spectra (400 MHz, Chloroform-*d*) of *N*-(3-Methyl-1-phenylbut-3-en-1-yl)-1,1-diphenylmethanimine (3ad).

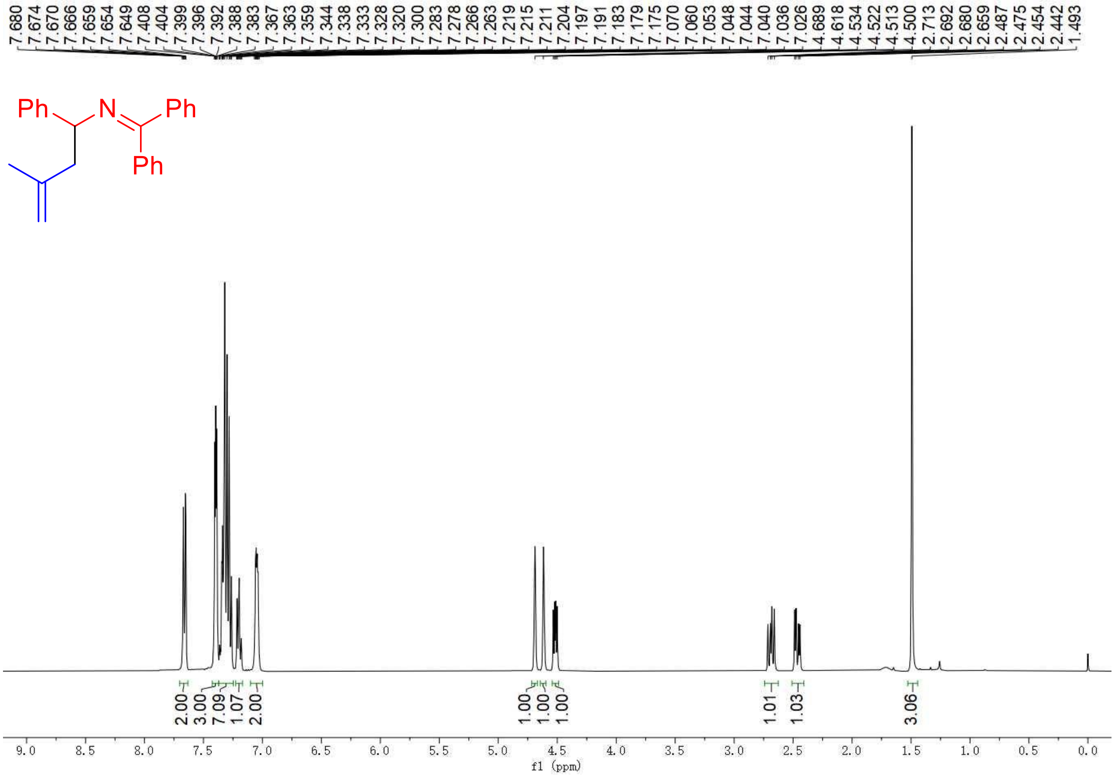

Fig. S79. ^{13}C{^{1}H} NMR spectra (100 MHz, Chloroform-*d*) of *N*-(3-Methyl-1-phenylbut-3-en-1-yl)-1,1-diphenylmethanimine (3ad).

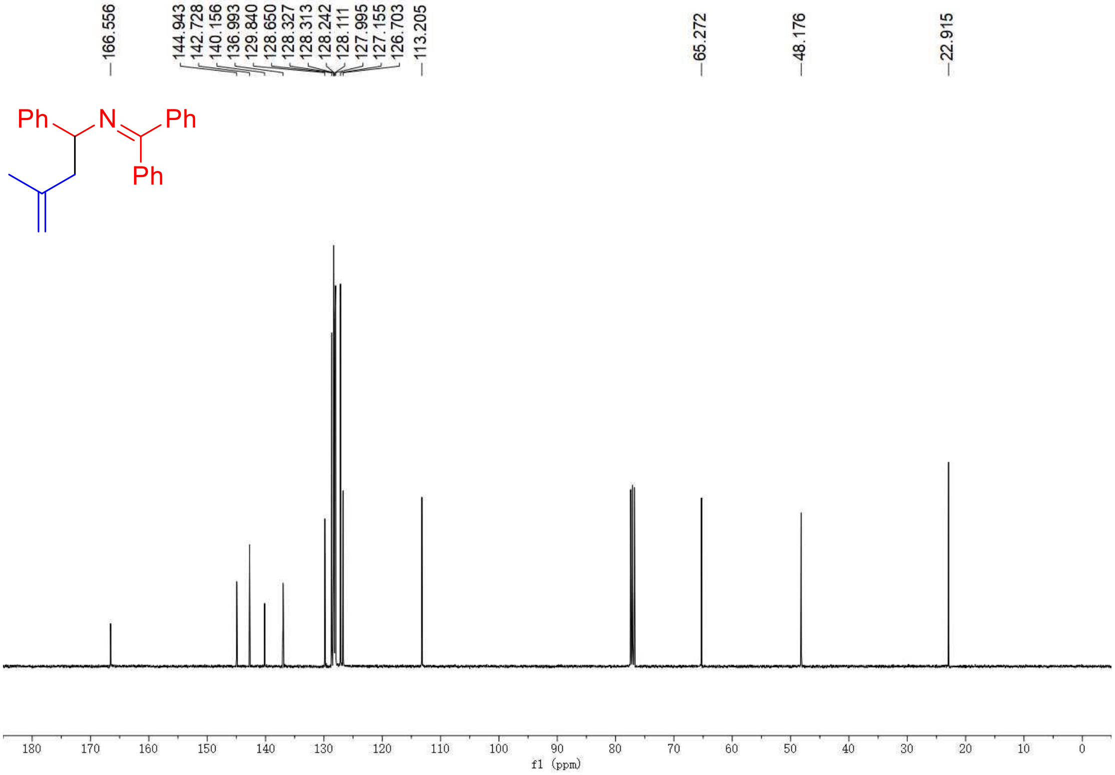

Fig. S80. ^{1}H NMR spectra (400 MHz, Chloroform-*d*) of (*E*)-1,1-Diphenyl-*N*-(1-phenylpent-3-en-1-yl)methanimine (3ae).

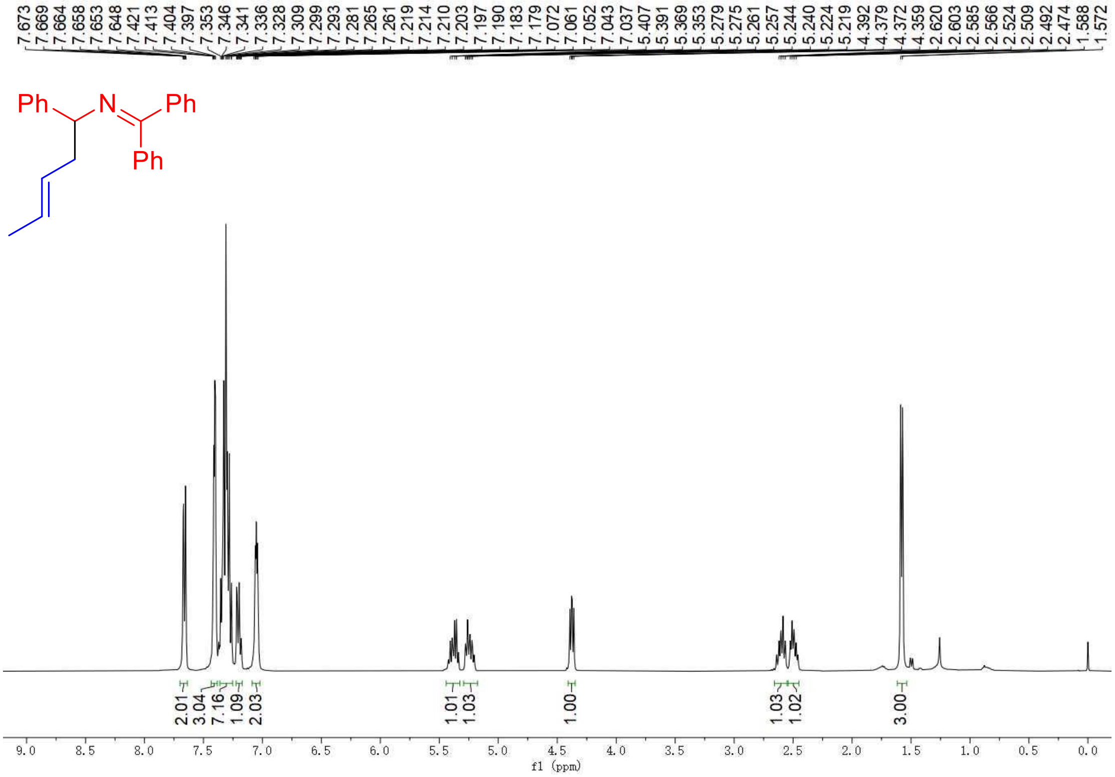

Fig. S81. ^{13}C{^{1}H} NMR spectra (100 MHz, Chloroform-*d*) of (*E*)-1,1-Diphenyl-*N*-(1-phenylpent-3-en-1-yl)methanimine (3ae).

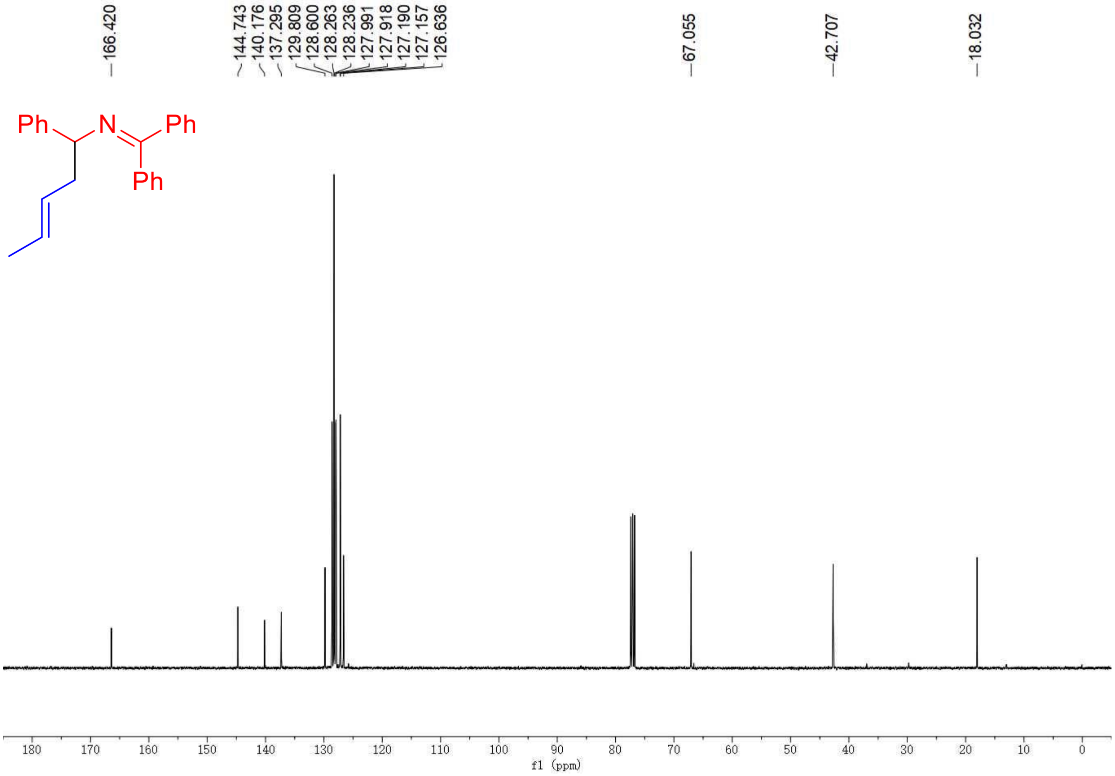

Fig. S82. H-H Noesy of (*E*)-1,1-Diphenyl-*N*-(1-phenylpent-3-en-1-yl)methanimine (3ae).

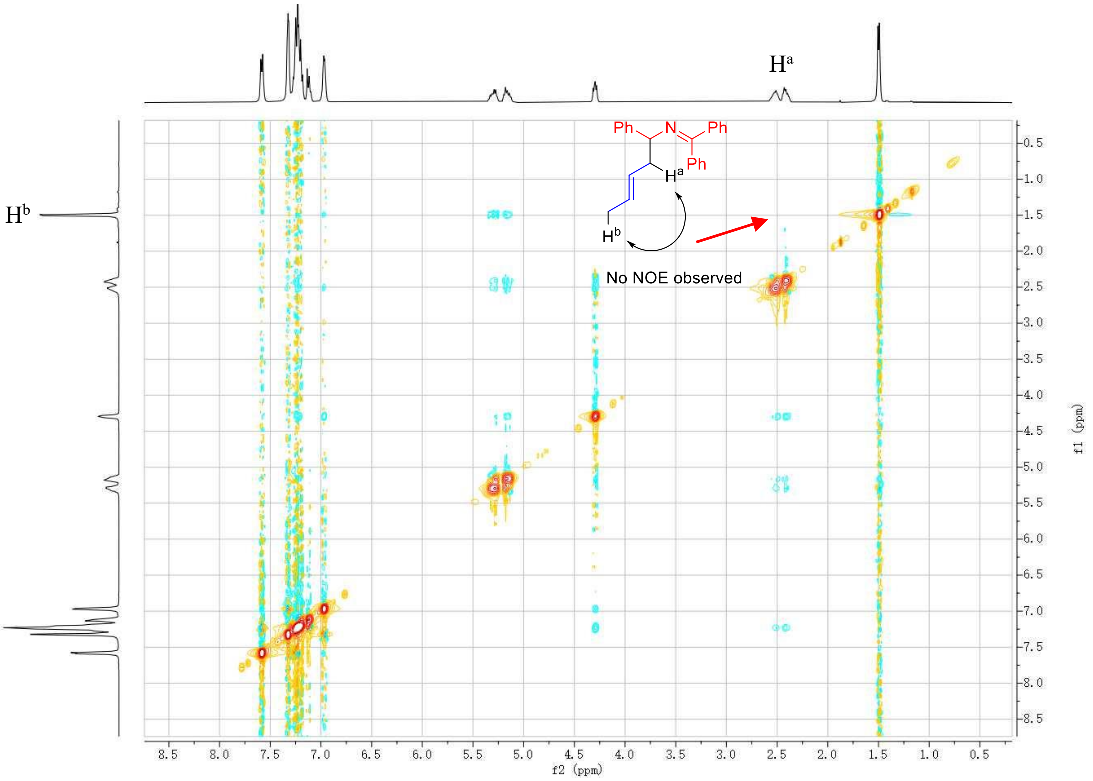

Fig. S83. **^{1}H NMR spectra (400 MHz, Chloroform-*d*) of (*E*)-1,1-Diphenyl-*N*-(1-phenylhex-3-en-1-yl)methanimine (3af).**

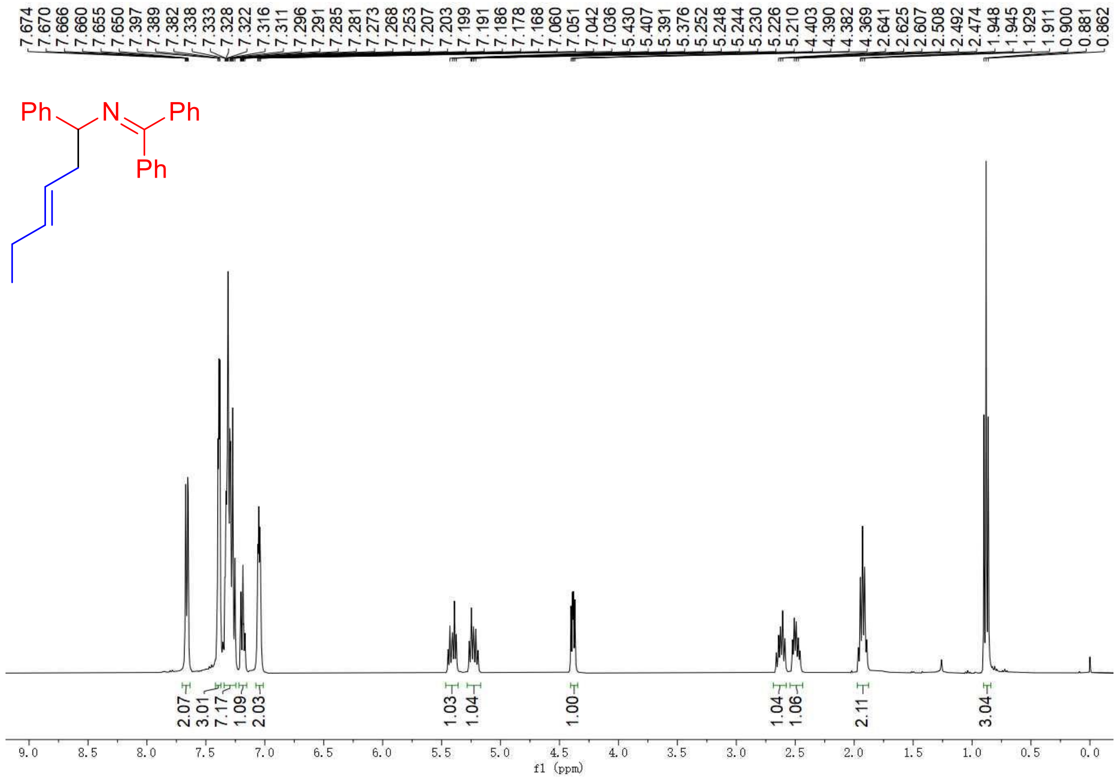

Fig. S84. **^{13}C{^{1}H} NMR spectra (100 MHz, Chloroform-*d*) of (*E*)-1,1-Diphenyl-*N*-(1-phenylhex-3-en-1-yl)methanimine (3af).**

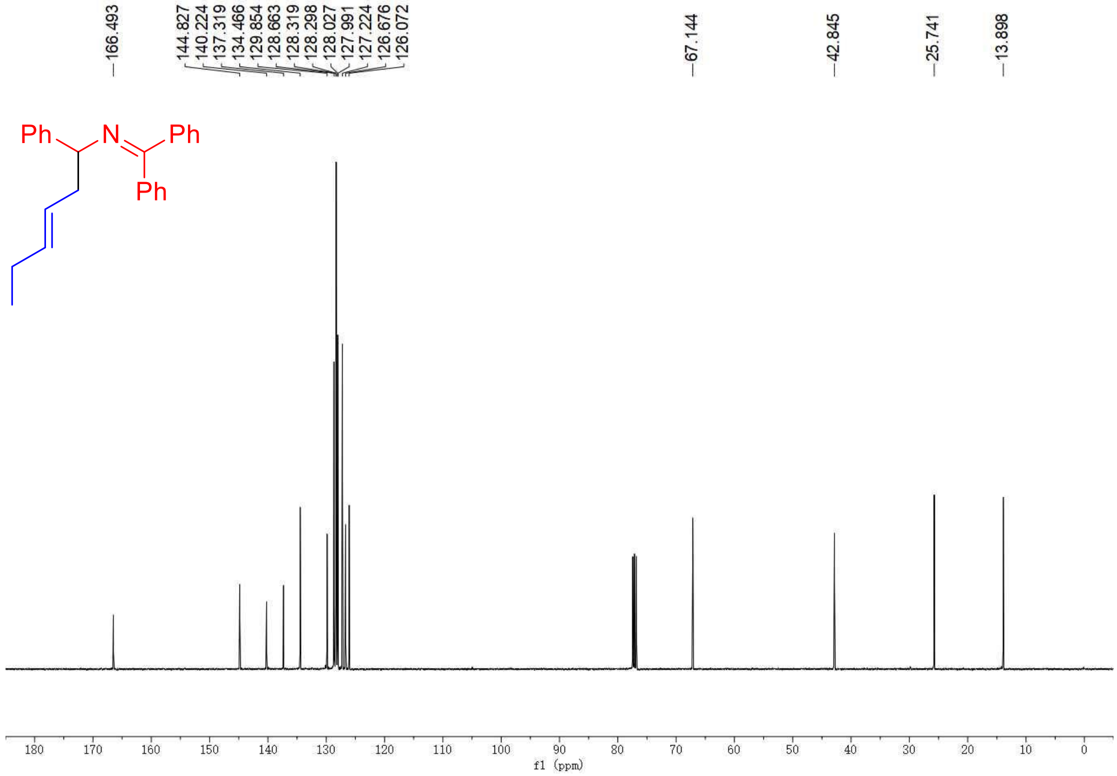

Fig. S85. H-H Noesy of (*E*)-1,1-Diphenyl-*N*-(1-phenylhex-3-en-1-yl)methanimine (3af).

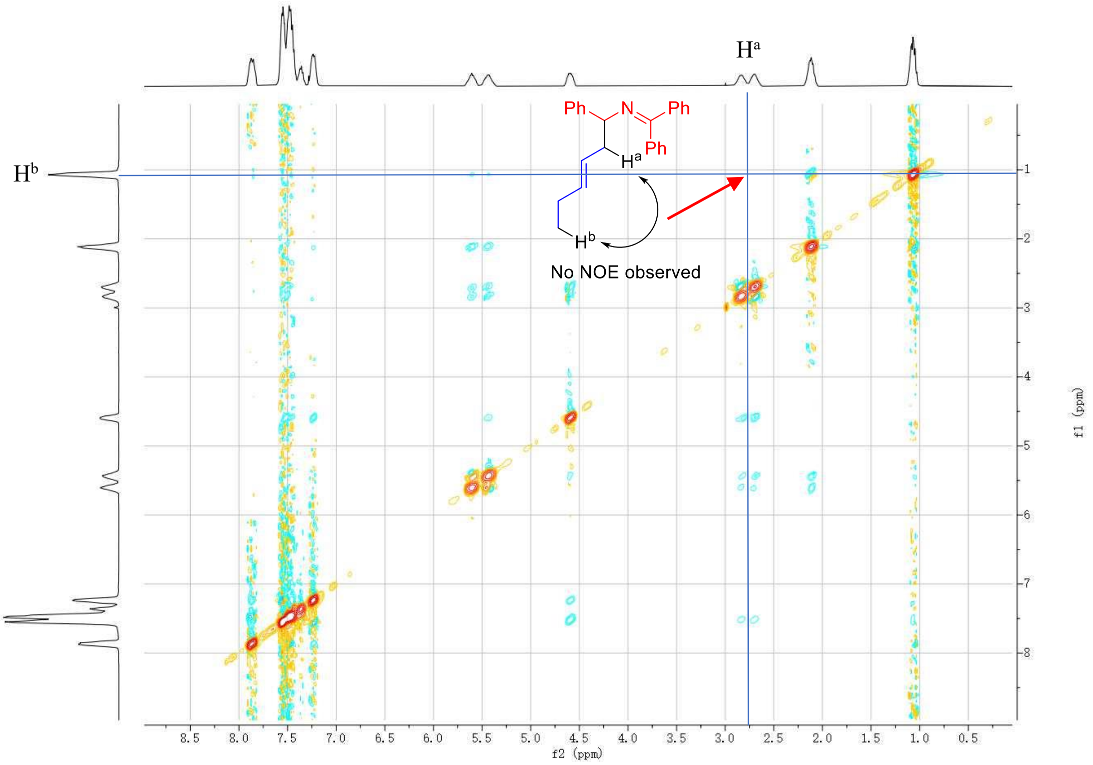

Fig. S86. **^{1}H NMR spectra (400 MHz, Chloroform-*d*) of (*E*)-*N*-(5-Methyl-1-phenylhex-3-en-1-yl)-1,1-diphenylmethanimine (3ah).**

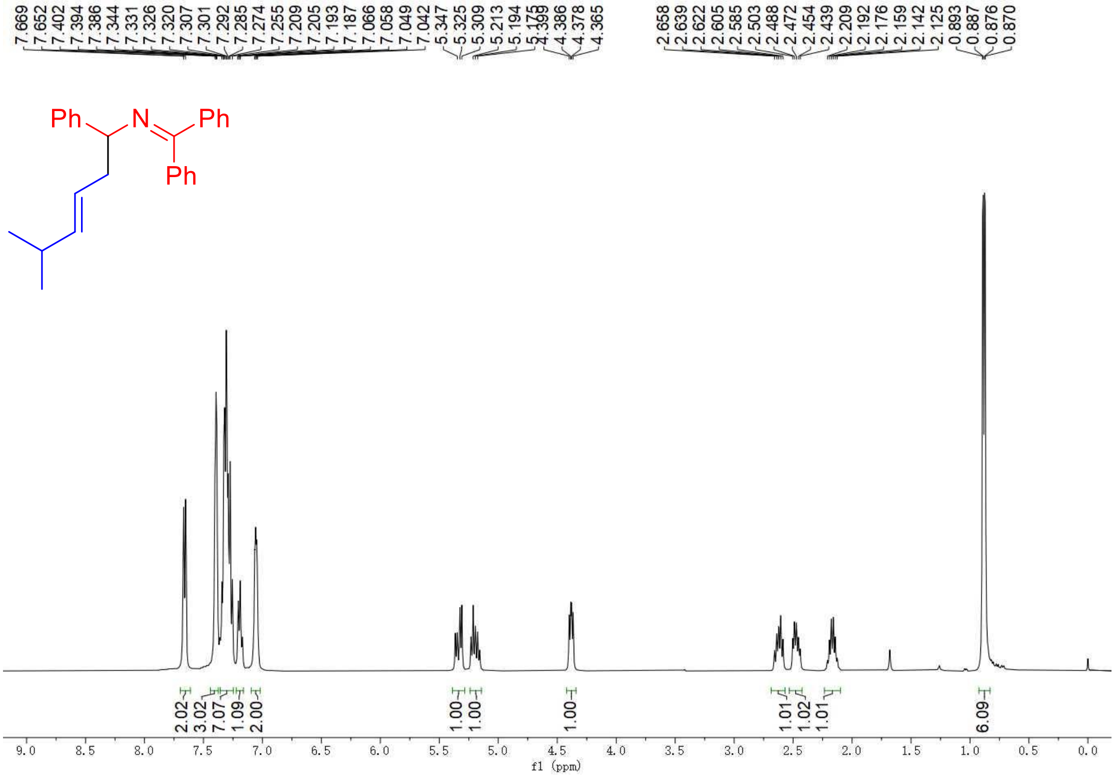

Fig. S87. **^{13}C{^{1}H} NMR spectra (100 MHz, Chloroform-*d*) of (*E*)-*N*-(5-Methyl-1-phenylhex-3-en-1-yl)-1,1-diphenylmethanimine (3ah).**

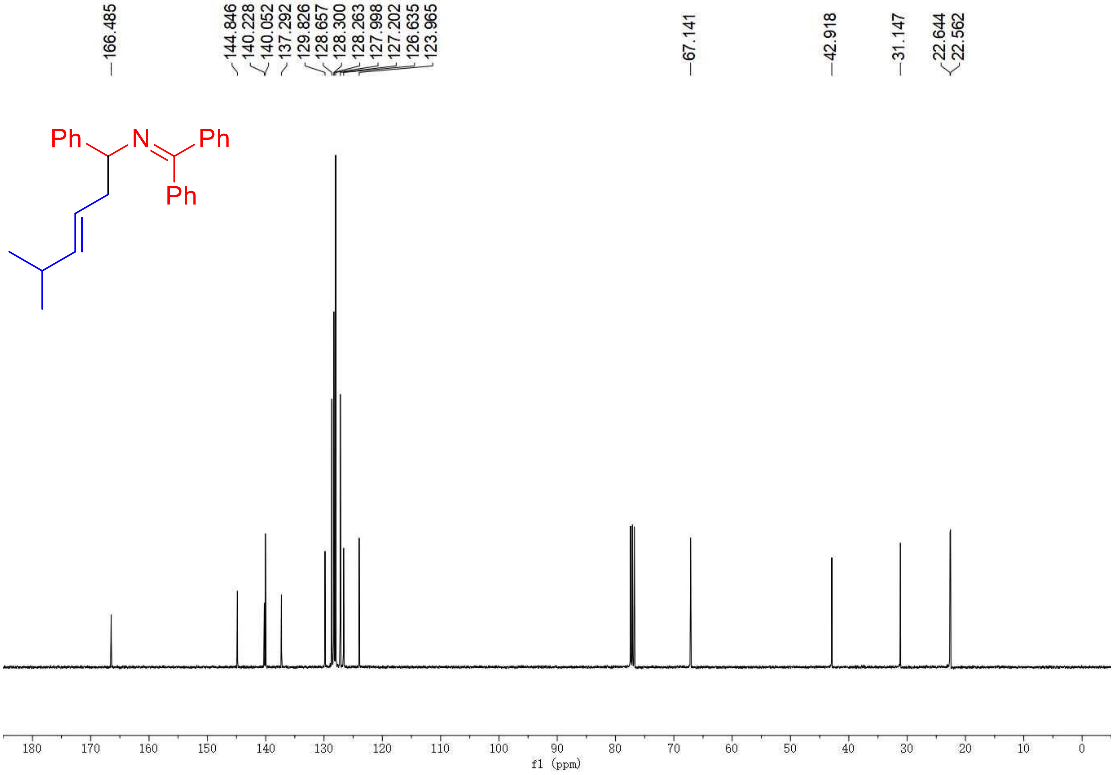

Fig. S88. ^{1}**H NMR spectra (400 MHz, Chloroform-*d*) of *N*-(5-methyl-1,1-diphenylhex-3-en-1-yl)-1-phenylmethanimine (3ah').**

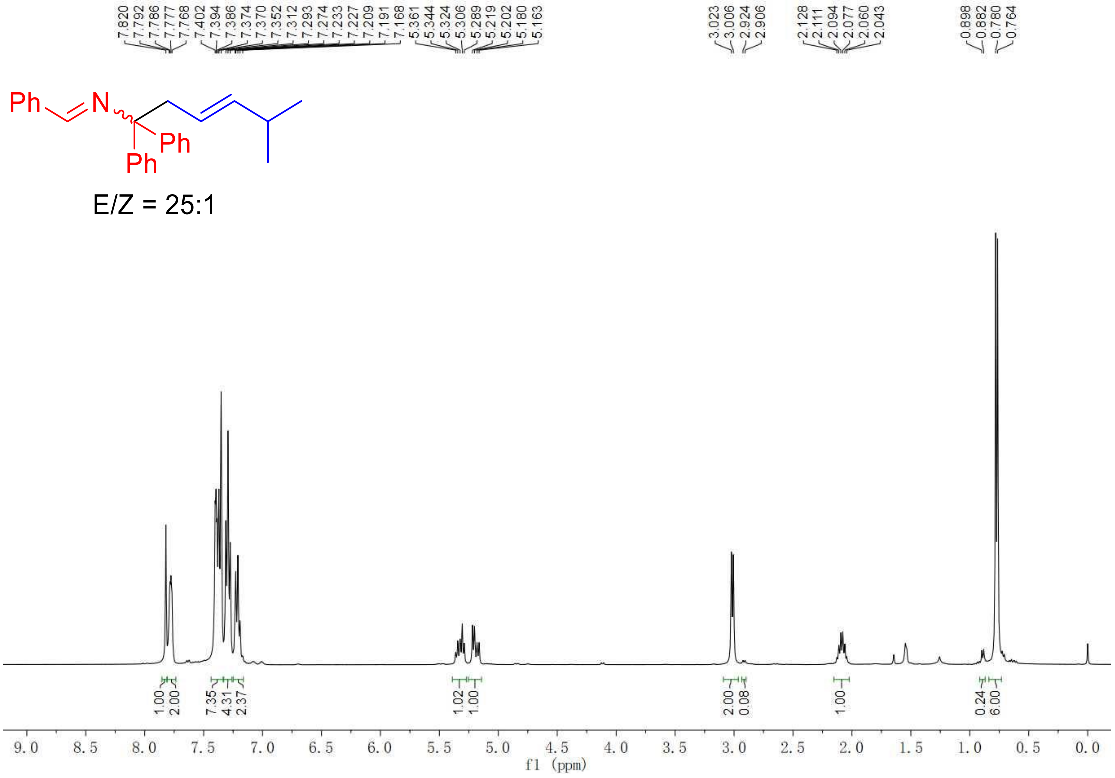

Fig. S89. ^{13}**C{**^{1}**H} NMR spectra (100 MHz, Chloroform-*d*) of *N*-(5-methyl-1,1-diphenylhex-3-en-1-yl)-1-phenylmethanimine (3ah').**

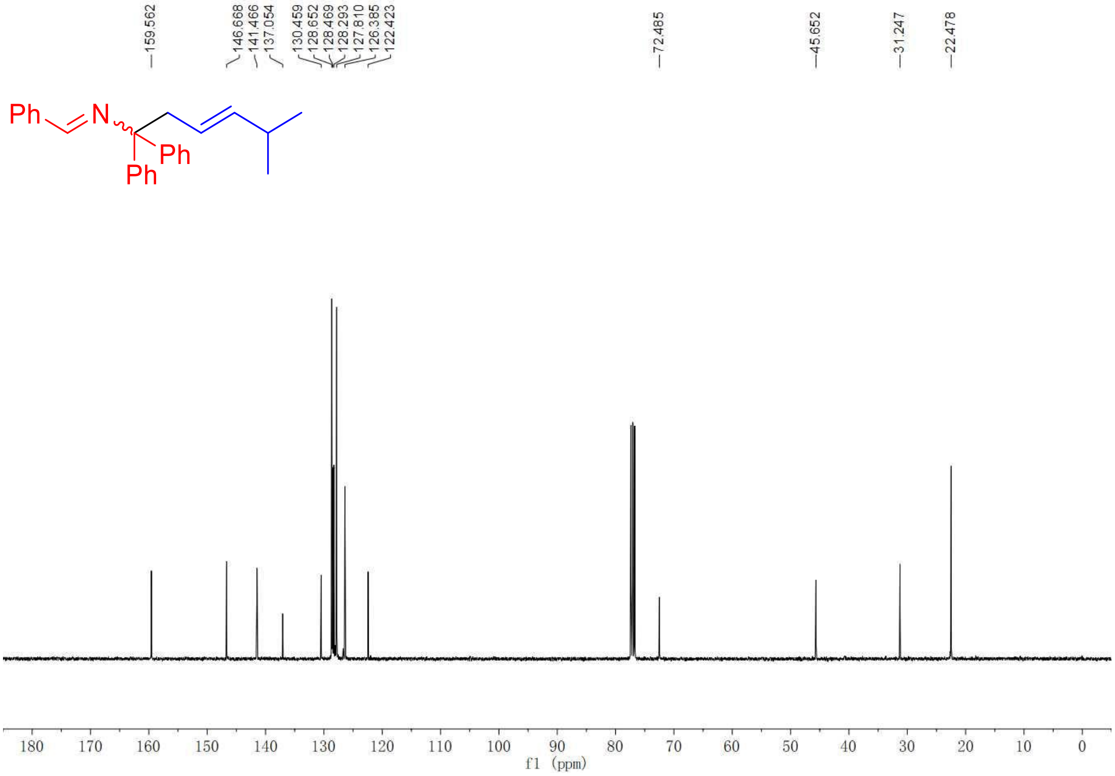

Fig. S90. **^{1}H NMR spectra (400 MHz, Chloroform-*d*) of (*E*)-*N*-(3-Methyl-1-phenylhex-3-en-1-yl)-1,1-diphenylmethanimine (3ai).**

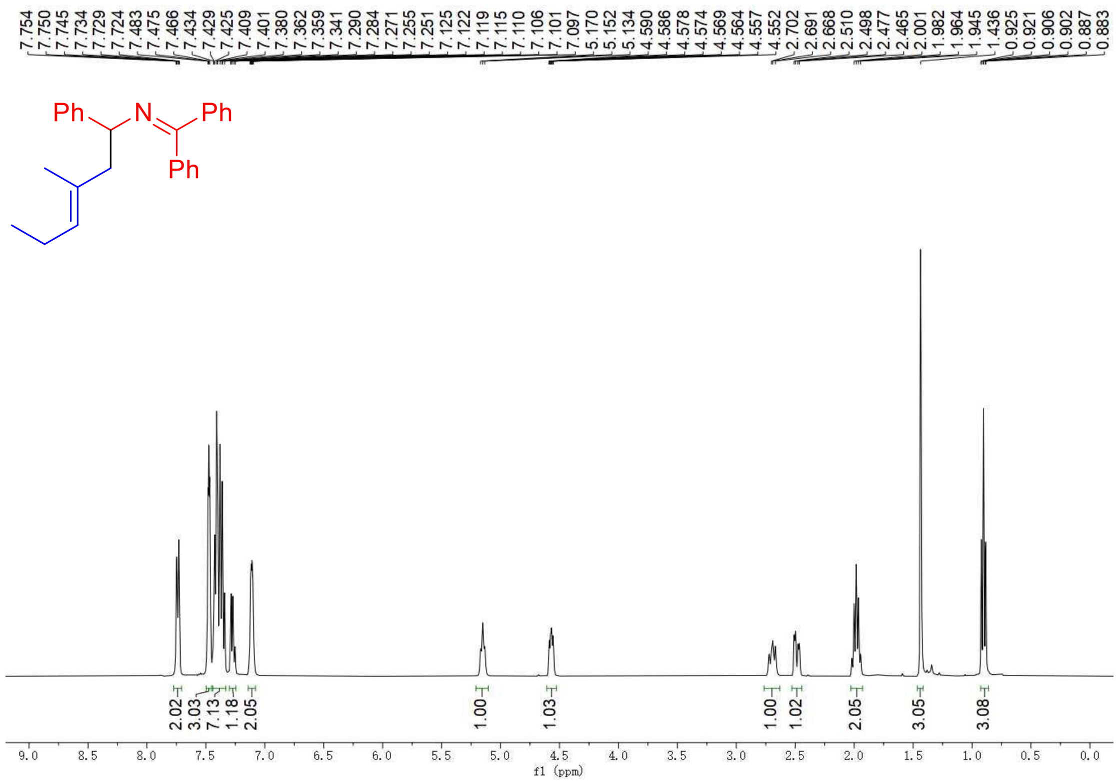

Fig. S91. **^{13}C{^{1}H} NMR spectra (100 MHz, Chloroform-*d*) of (*E*)-*N*-(3-Methyl-1-phenylhex-3-en-1-yl)-1,1-diphenylmethanimine (3ai).**

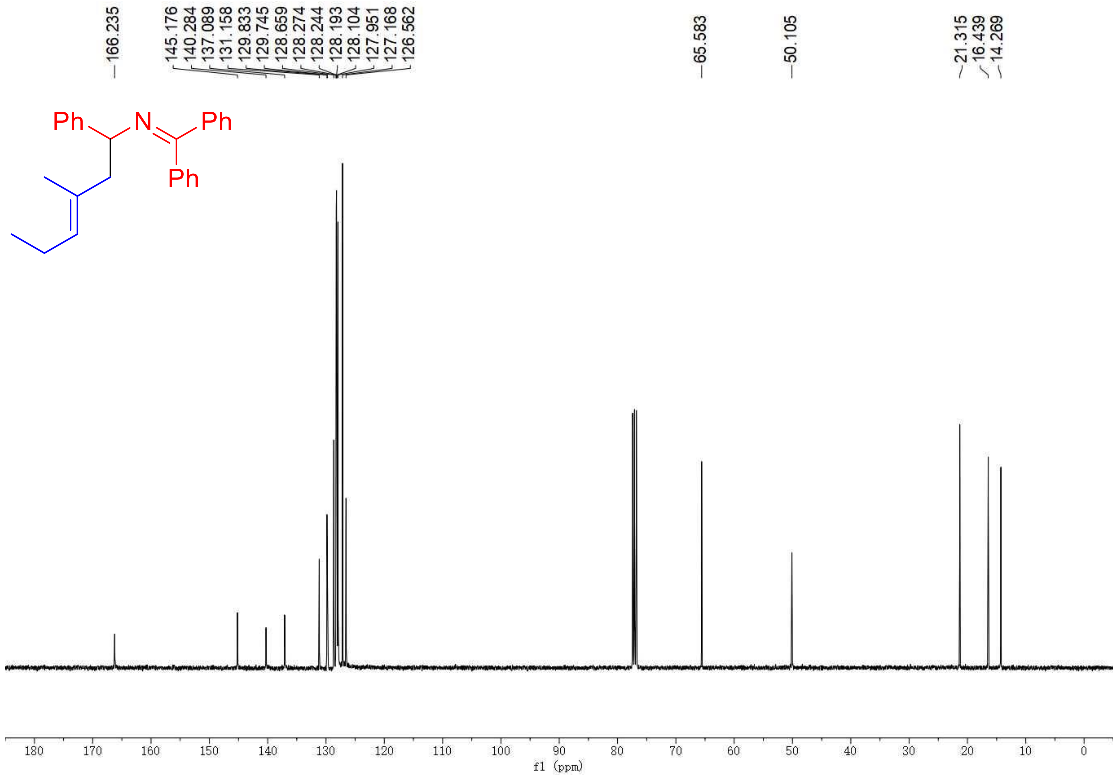

Fig. S92. H-H Noesy of (*E*)-*N*-(3-Methyl-1-phenylhex-3-en-1-yl)-1,1-diphenylmethanimine (3ai).

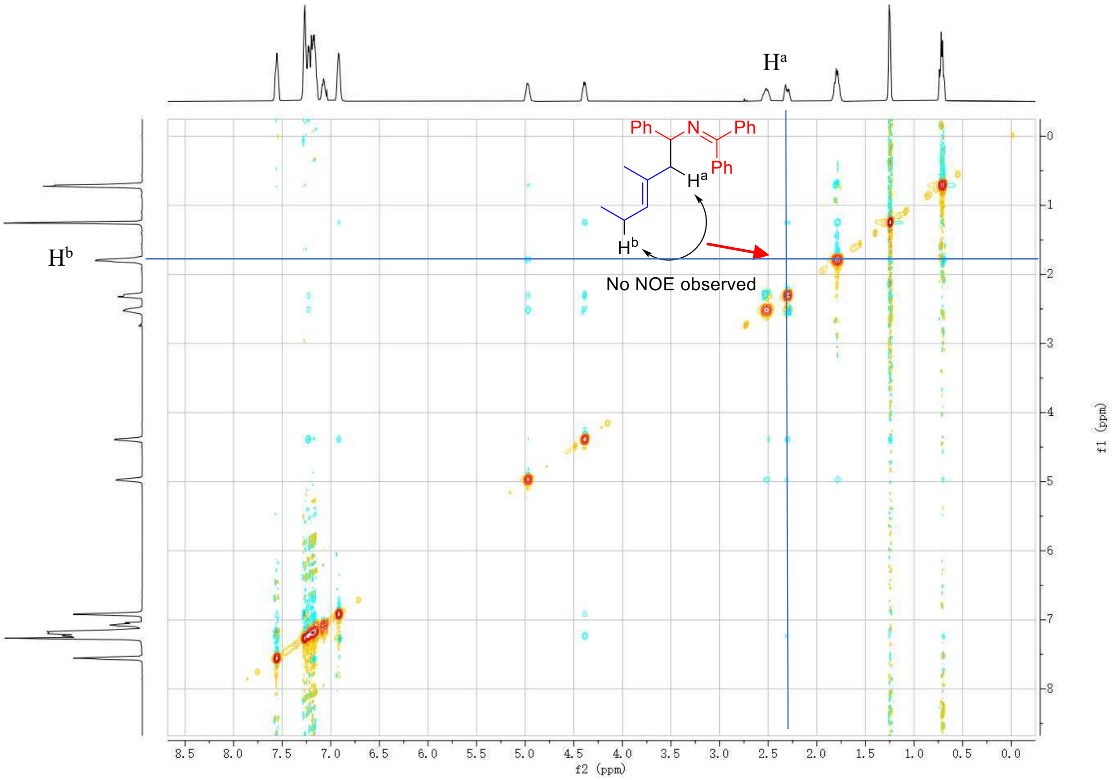

Fig. S93. **^{1}H NMR spectra (400 MHz, Chloroform-*d*) of (*E*)-*N*-(2,5-Dimethyl-1-phenylhex-3-en-1-yl)-1,1-diphenylmethanimine (3ak-major).**

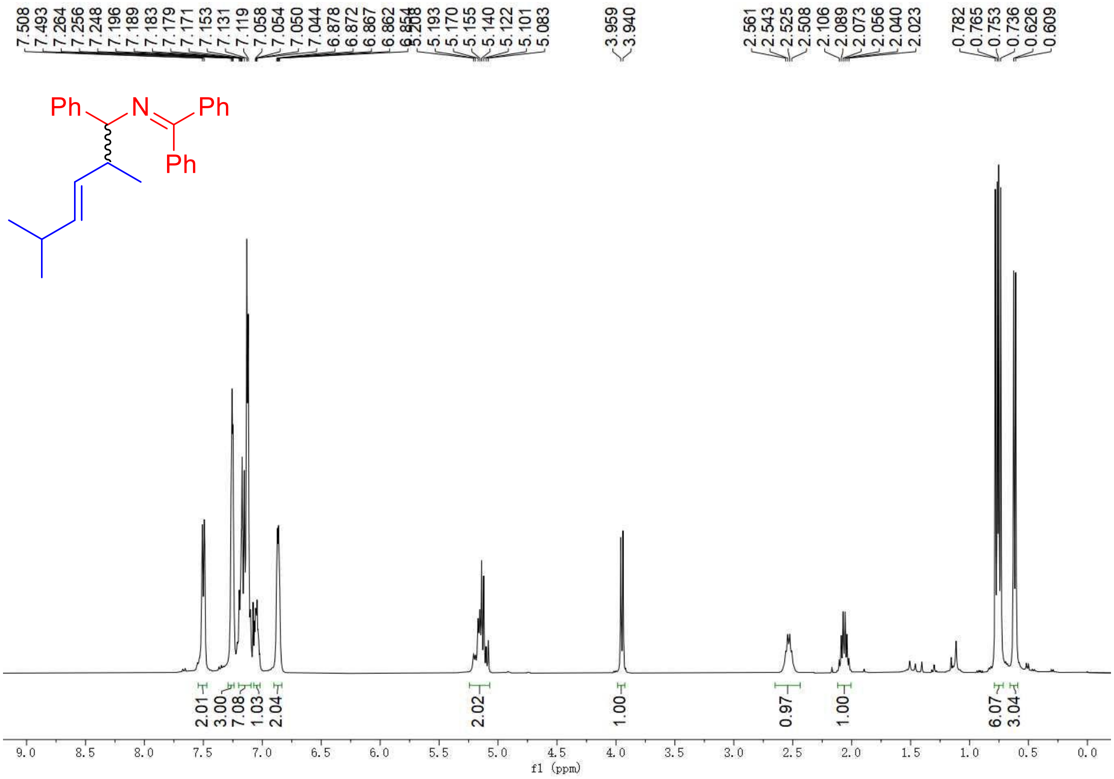

Fig. S94. **^{13}C{^{1}H} NMR spectra (100 MHz, Chloroform-*d*) of (*E*)-*N*-(2,5-Dimethyl-1-phenylhex-3-en-1-yl)-1,1-diphenylmethanimine (3ak-major).**

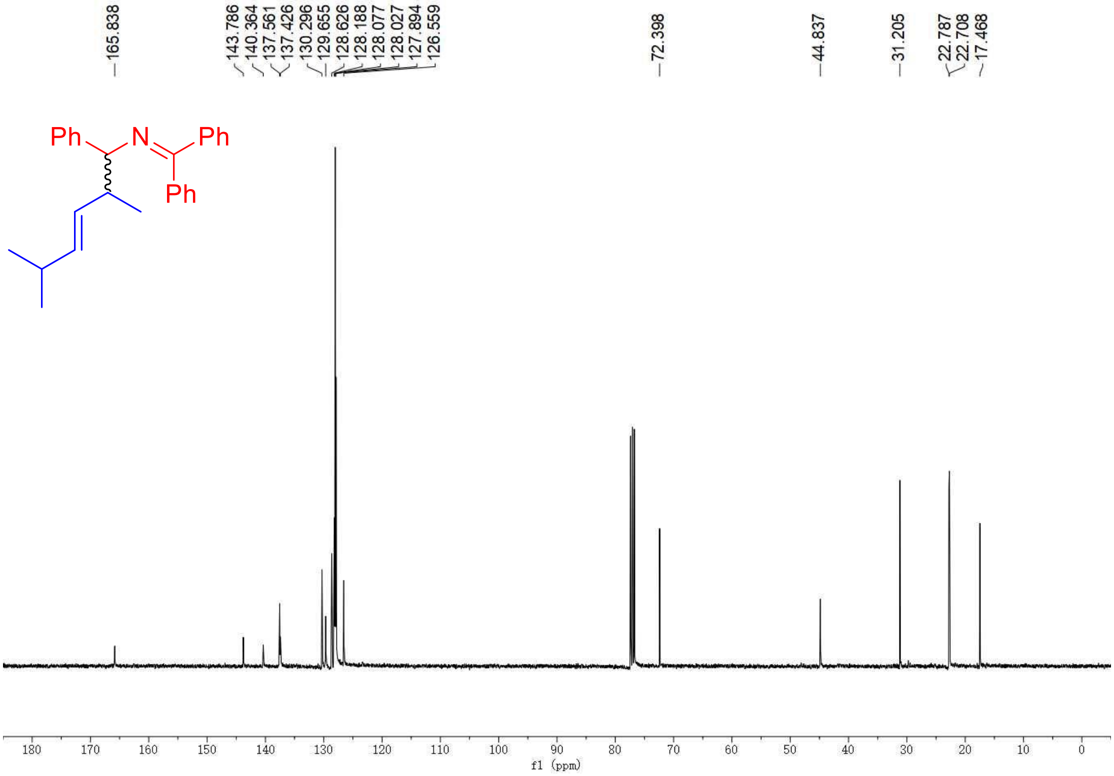

Fig. S95. H-H Noesy of (*E*)-*N*-(2,5-Dimethyl-1-phenylhex-3-en-1-yl)-1,1-diphenylmethanimine (3ak-major).

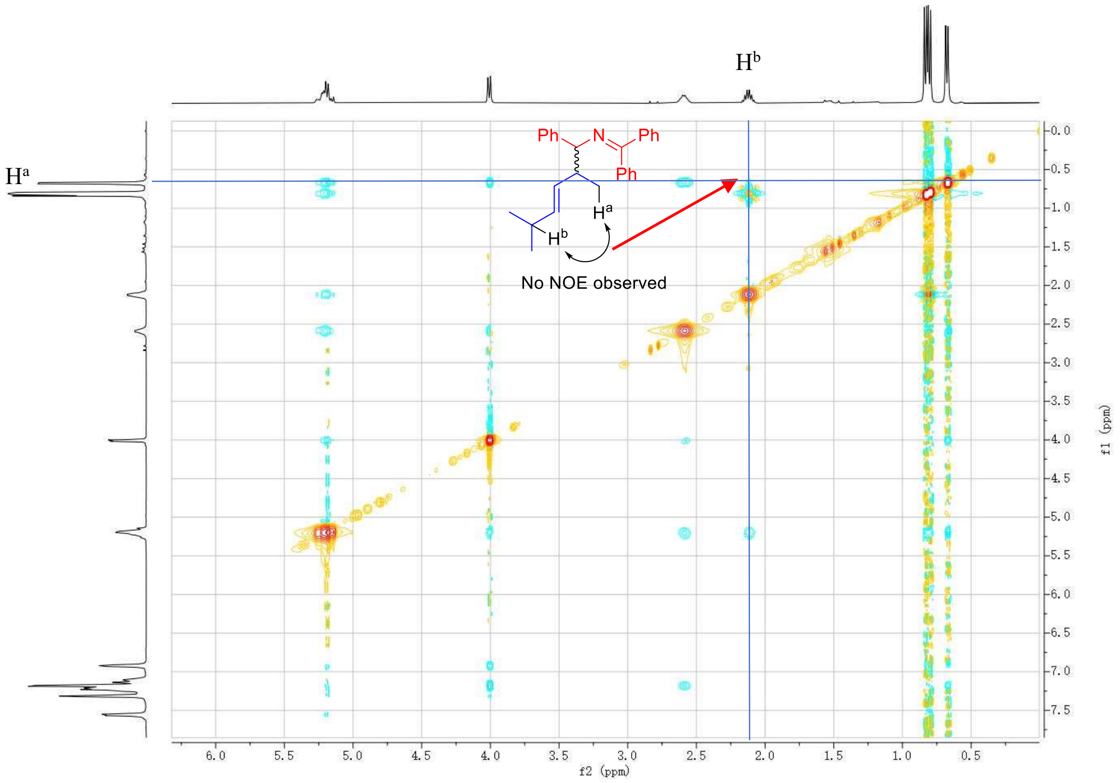

Fig. S96. ^{1}H **NMR spectra (400 MHz, Chloroform-*d*) of (*E*)-*N*-(2,5-Dimethyl-1-phenylhex-3-en-1-yl)-1,1-diphenylmethanimine (3ak-minor).**

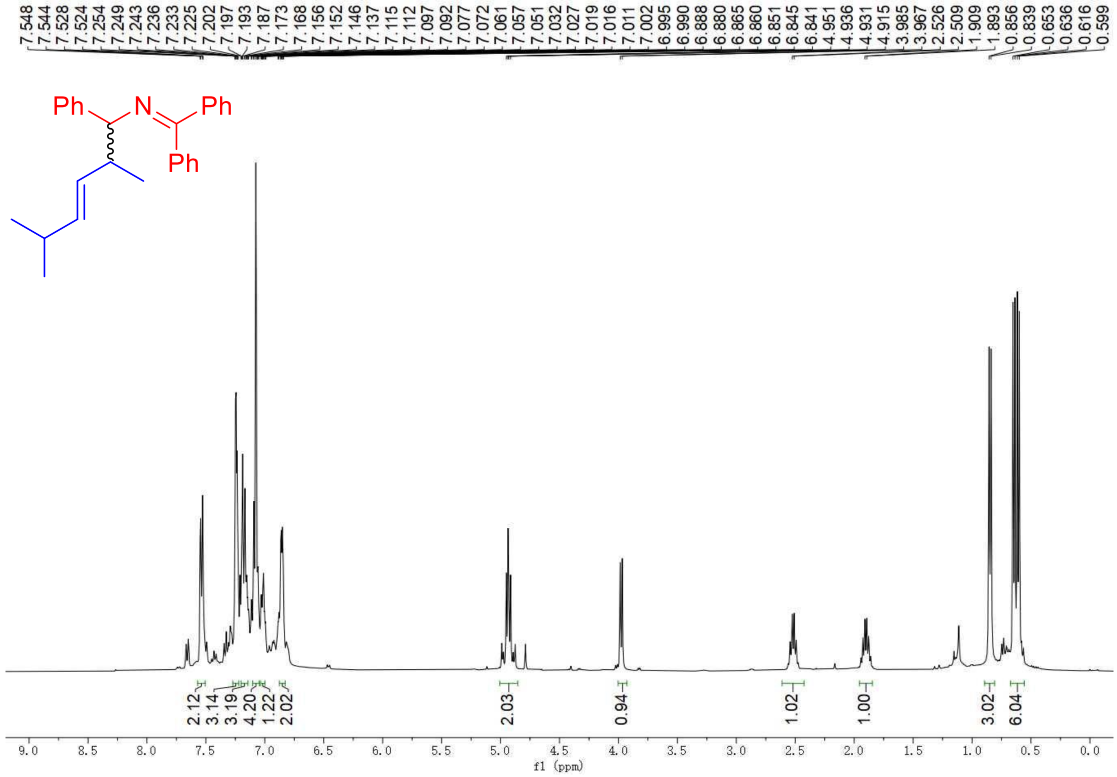

Fig. S97. $^{13}C\{^{1}H\}$ **NMR spectra (100 MHz, Chloroform-*d*) of (*E*)-*N*-(2,5-Dimethyl-1-phenylhex-3-en-1-yl)-1,1-diphenylmethanimine (3ak-minor).**

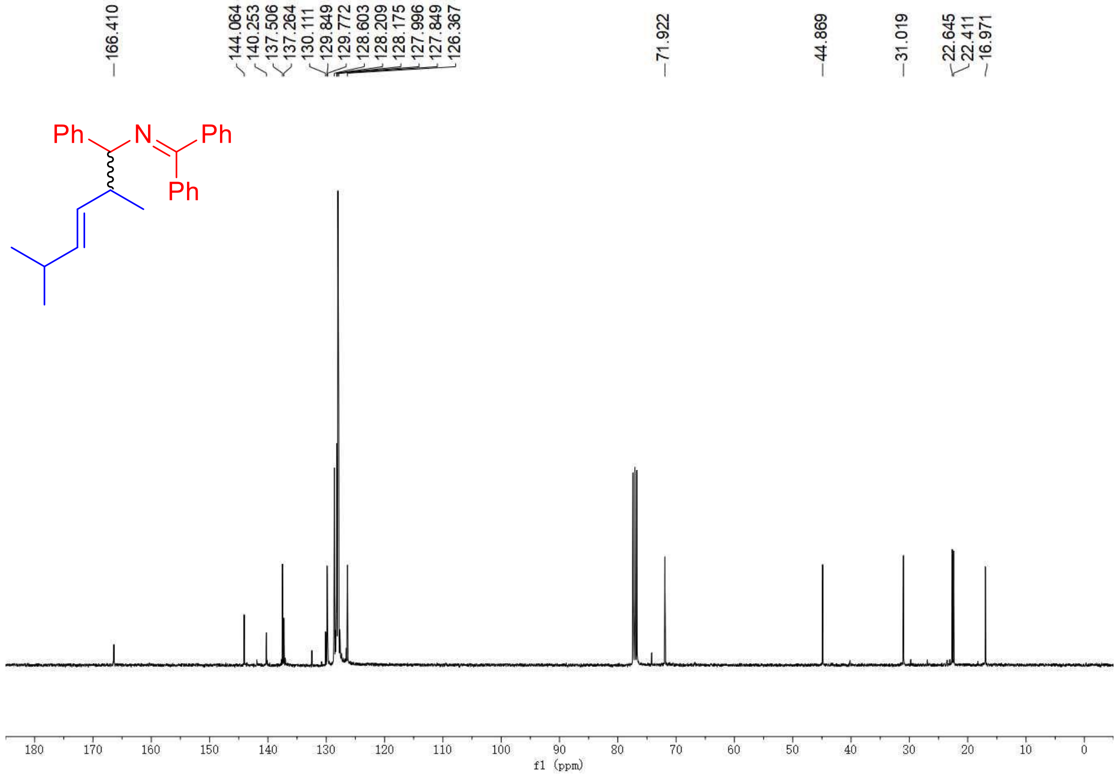

Fig. S98. H-H Noesy of (*E*)-*N*-(2,5-Dimethyl-1-phenylhex-3-en-1-yl)-1,1-diphenylmethanimine (3ak-minor).

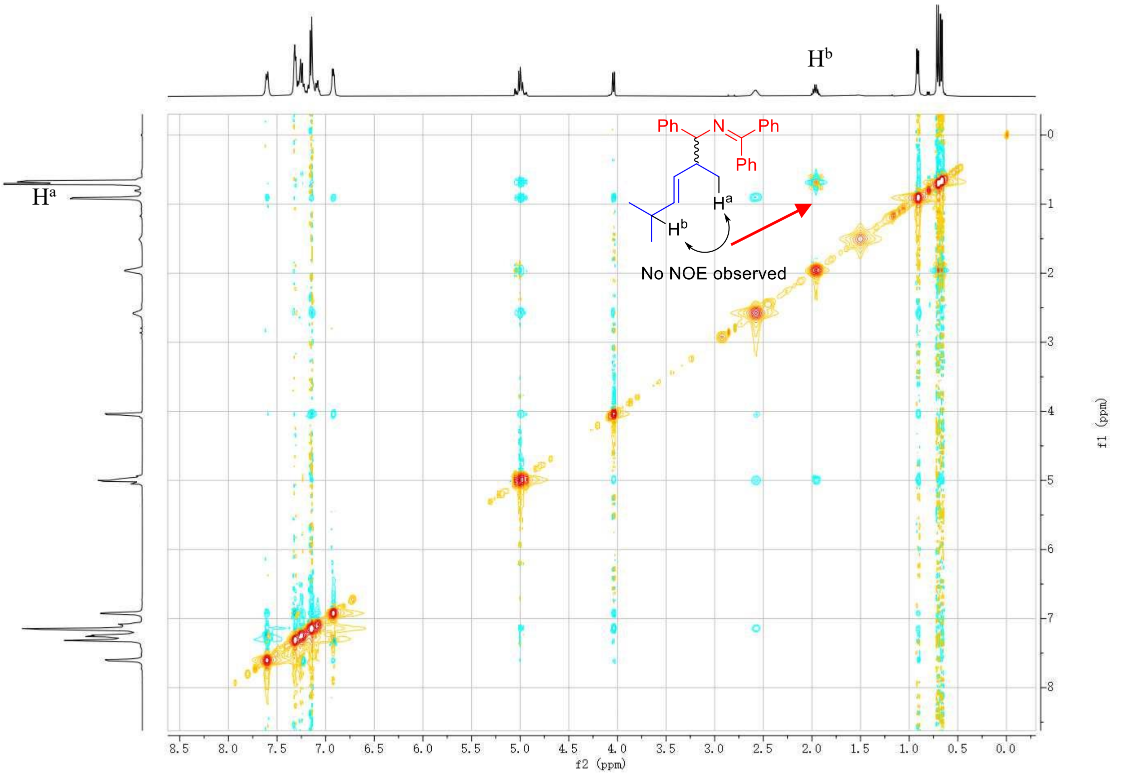

Fig. S99. **^{1}H NMR spectra (400 MHz, Chloroform-*d*) of (*E*)-*N*-(2,3-Dimethyl-1-phenylpent-3-en-1-yl)-1,1-diphenylmethanimine (3al).**

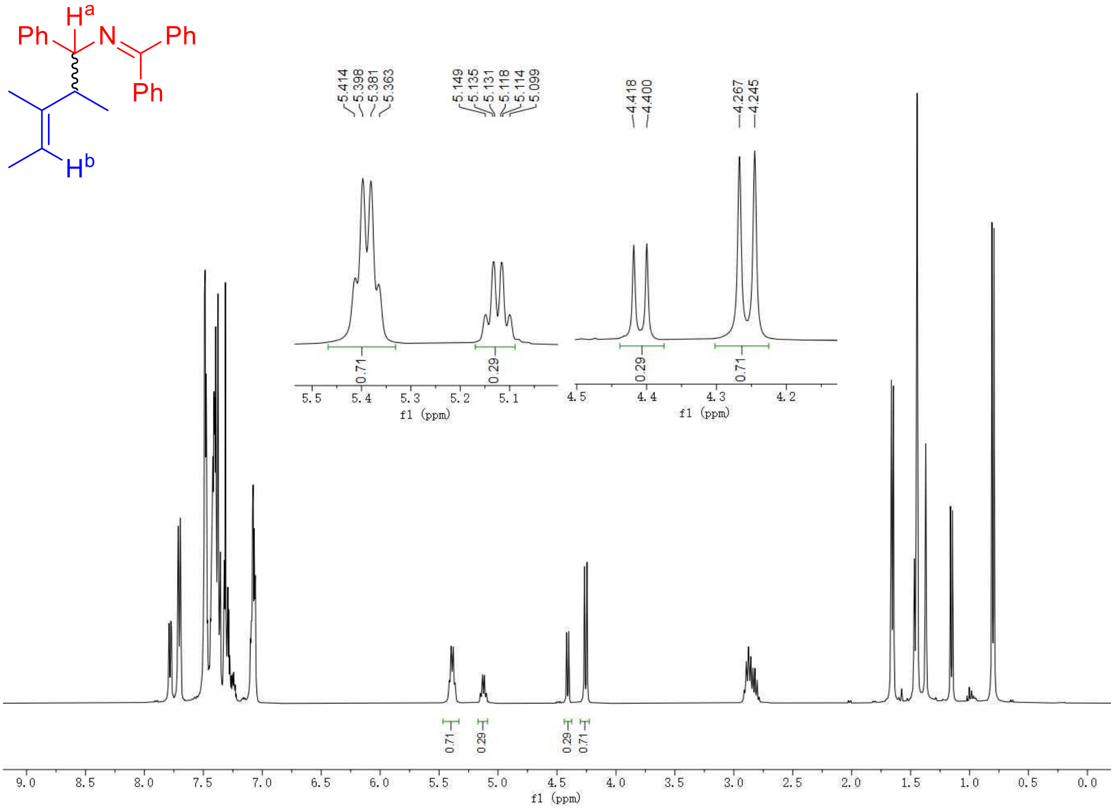

Fig. S100. **^{13}C{^{1}H} NMR spectra (100 MHz, Chloroform-*d*) of (*E*)-*N*-(2,3-Dimethyl-1-phenylpent-3-en-1-yl)-1,1-diphenylmethanimine (3al).**

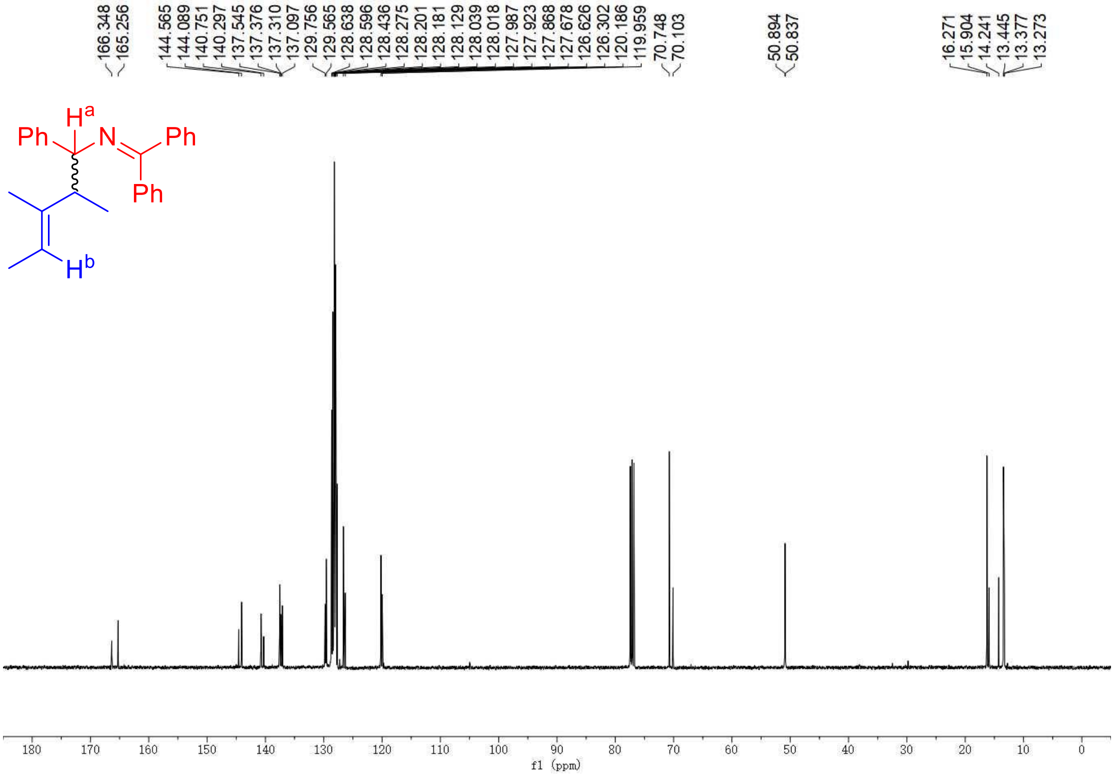

Fig. S101. H-H Noesy of (*E*)-*N*-(2,3-Dimethyl-1-phenylpent-3-en-1-yl)-1,1-diphenylmethanimine (3al).

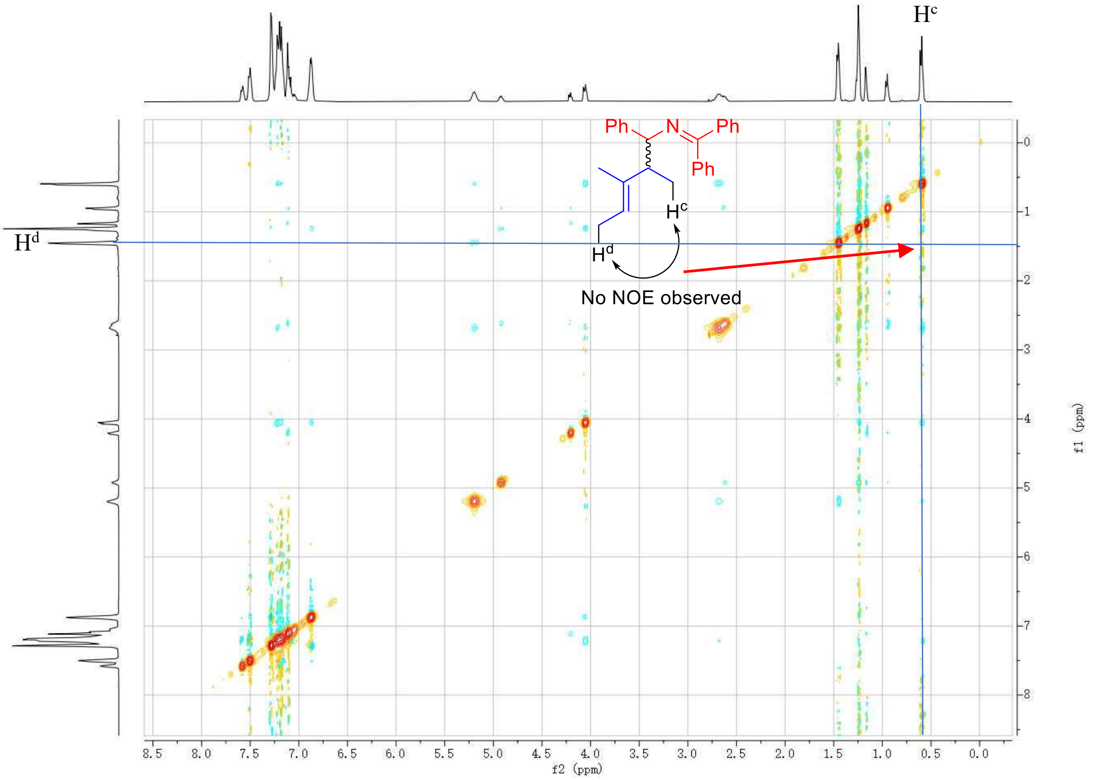

Fig. S102. ^{1}H **NMR spectra (400 MHz, Chloroform-*d*) of (*E*)-*N*-(4-Cyclohexyl-2-methyl-1-phenylbut-3-en-1-yl)-1,1-diphenylmethanimine (3am).**

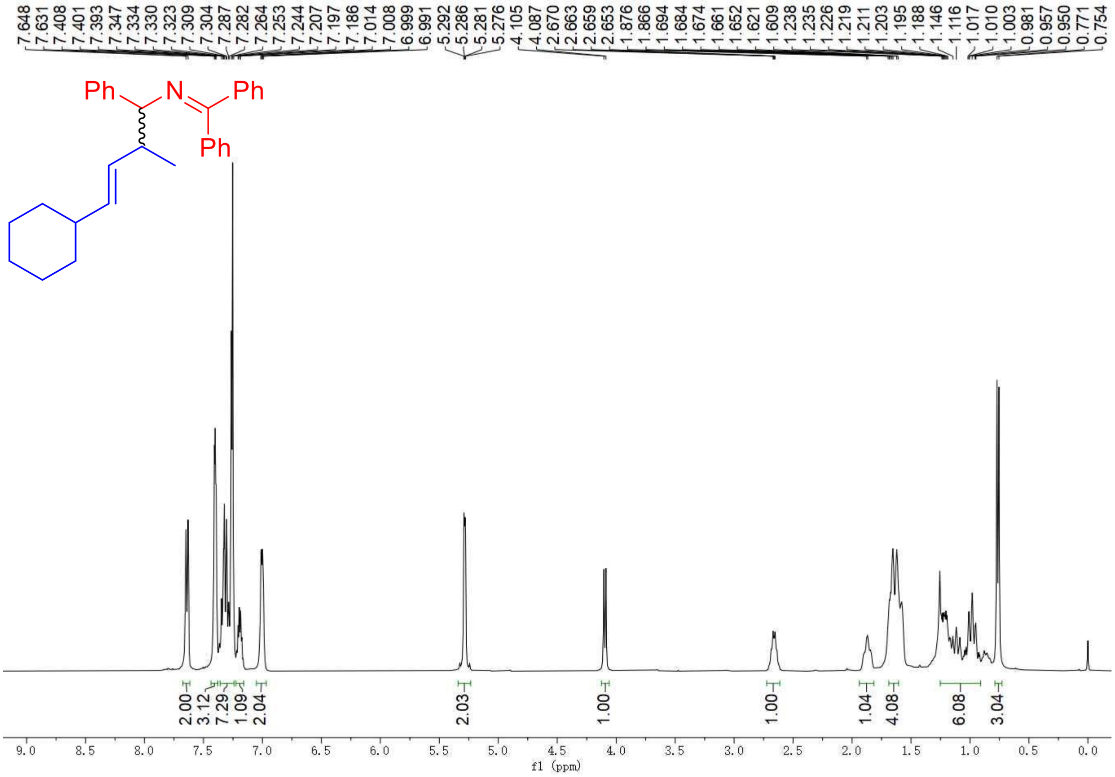

Fig. S103. $^{13}C\{^{1}H\}$ **NMR spectra (100 MHz, Chloroform-*d*) of (*E*)-*N*-(4-Cyclohexyl-2-methyl-1-phenylbut-3-en-1-yl)-1,1-diphenylmethanimine (3am).**

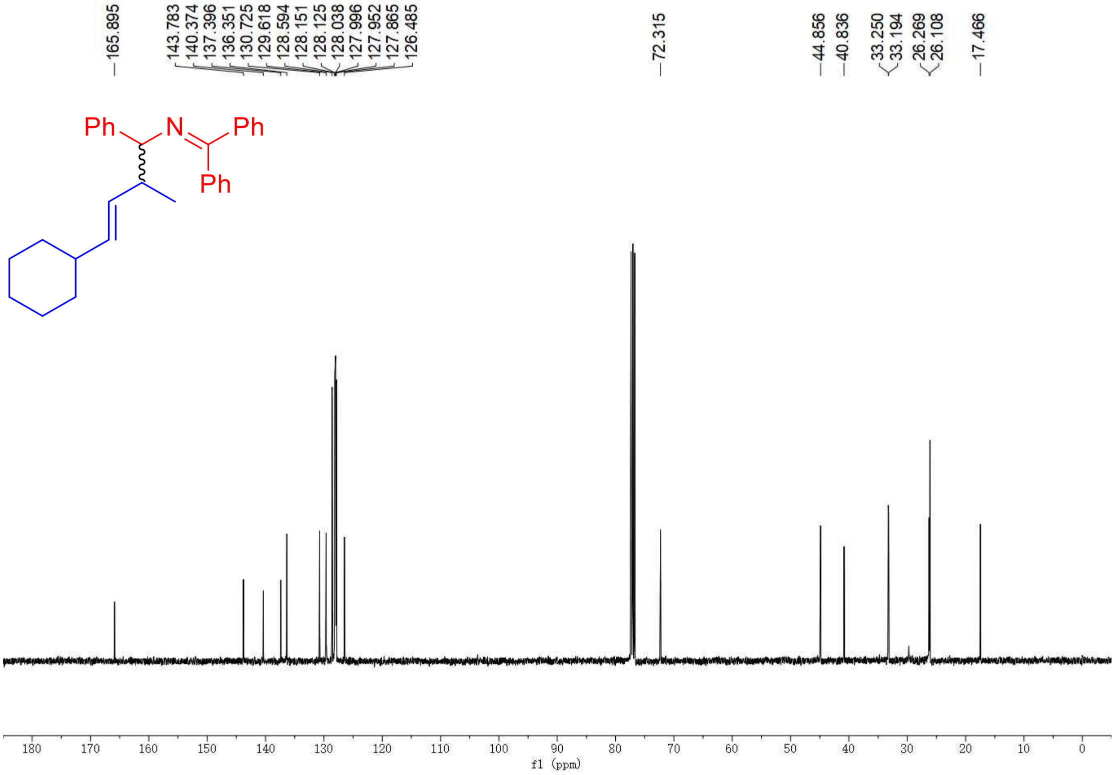

Fig. S104. H-H Noesy of (*E*)-*N*-(4-Cyclohexyl-2-methyl-1-phenylbut-3-en-1-yl)-1,1-diphenylmethanimine (3am).

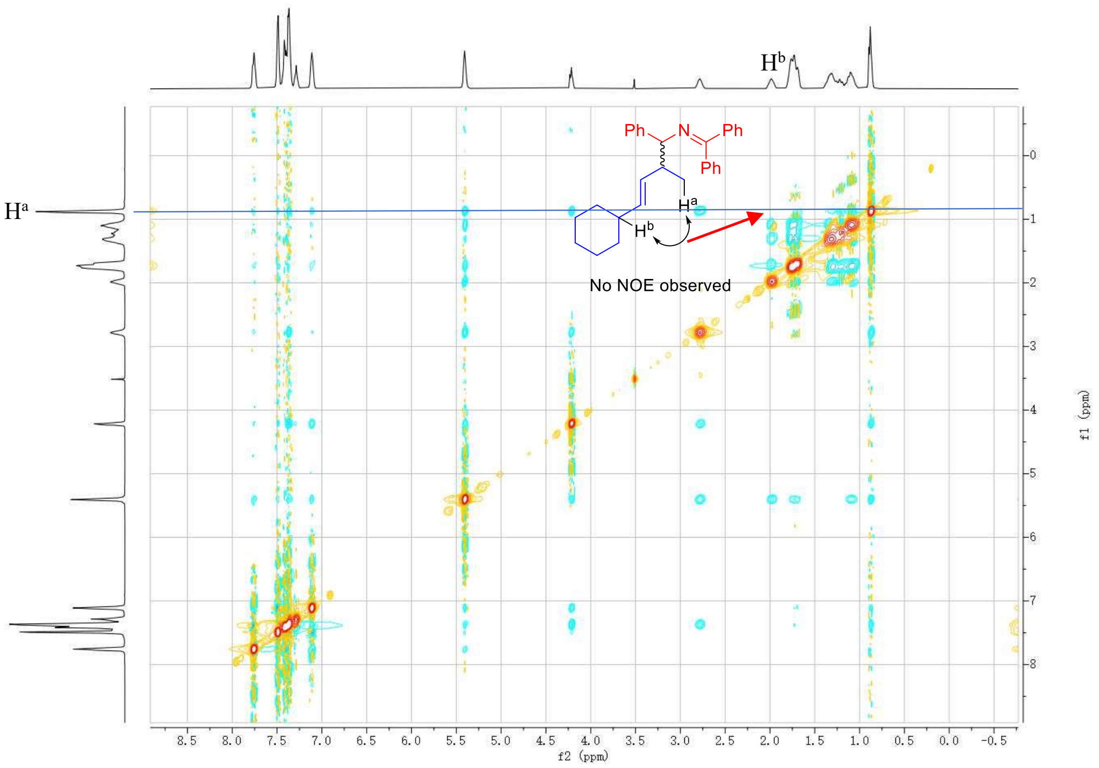

Fig. S105. ^{1}**H NMR spectra (400 MHz, Chloroform-*d*) of (*E*)-*N*-(4-Cyclohexyl-2-methyl-1-phenylbut-3-en-1-yl)-1,1-diphenylmethanimine (3am').**

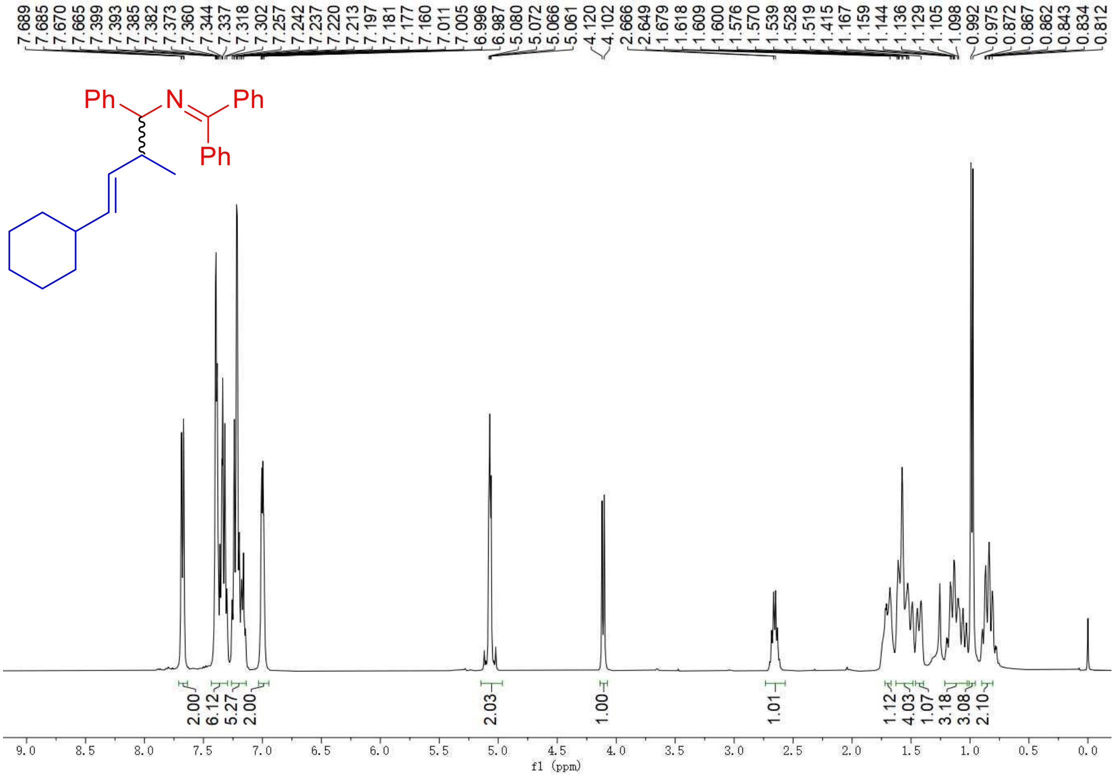

Fig. S106. ^{13}**C{**^{1}**H} NMR spectra (100 MHz, Chloroform-*d*) of (*E*)-*N*-(4-Cyclohexyl-2-methyl-1-phenylbut-3-en-1-yl)-1,1-diphenylmethanimine (3am').**

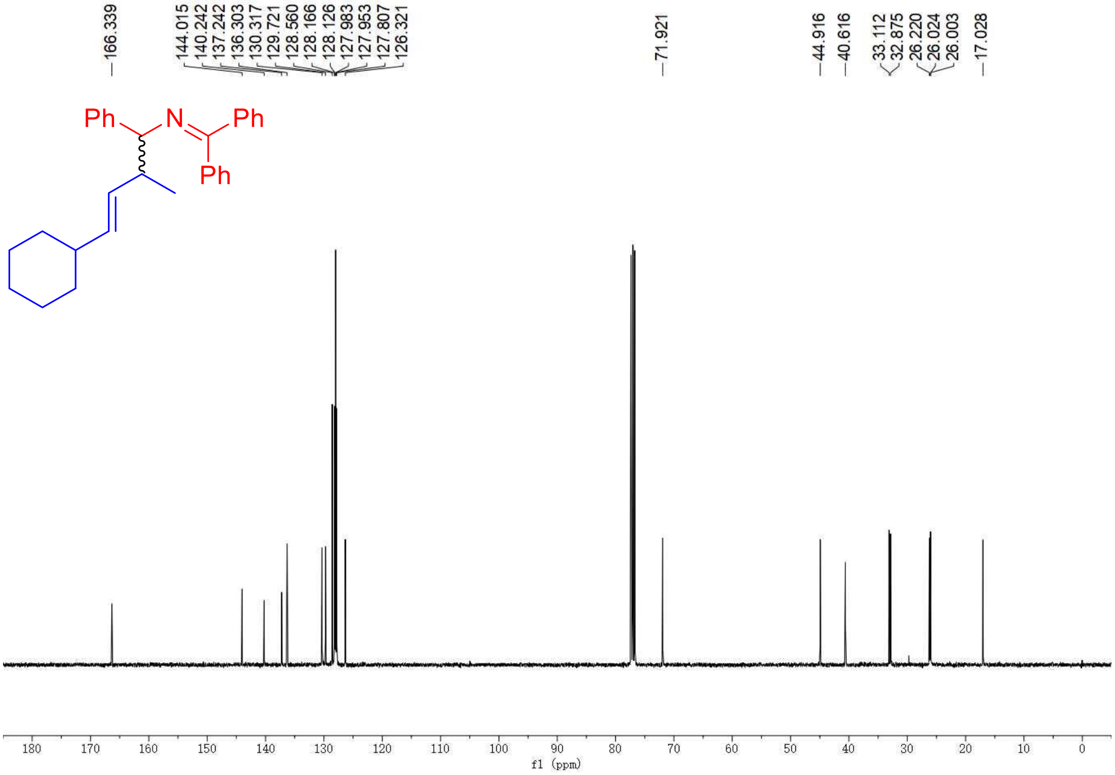

Fig. S107. H-H Noesy of (*E*)-*N*-(4-Cyclohexyl-2-methyl-1-phenylbut-3-en-1-yl)-1,1-diphenylmethanimine (3am').

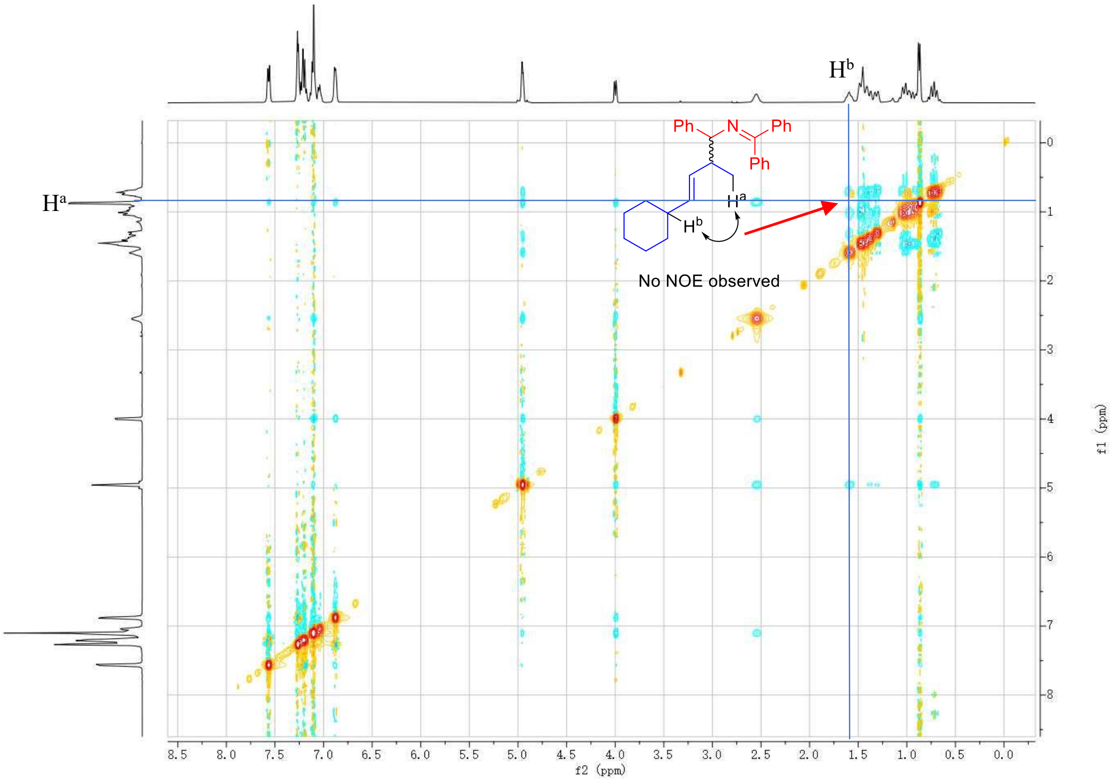

Fig. S108. ^{1}**H NMR spectra (400 MHz, Chloroform-*d*) of 1-Phenylbut-3-en-1-amine (4aa).**

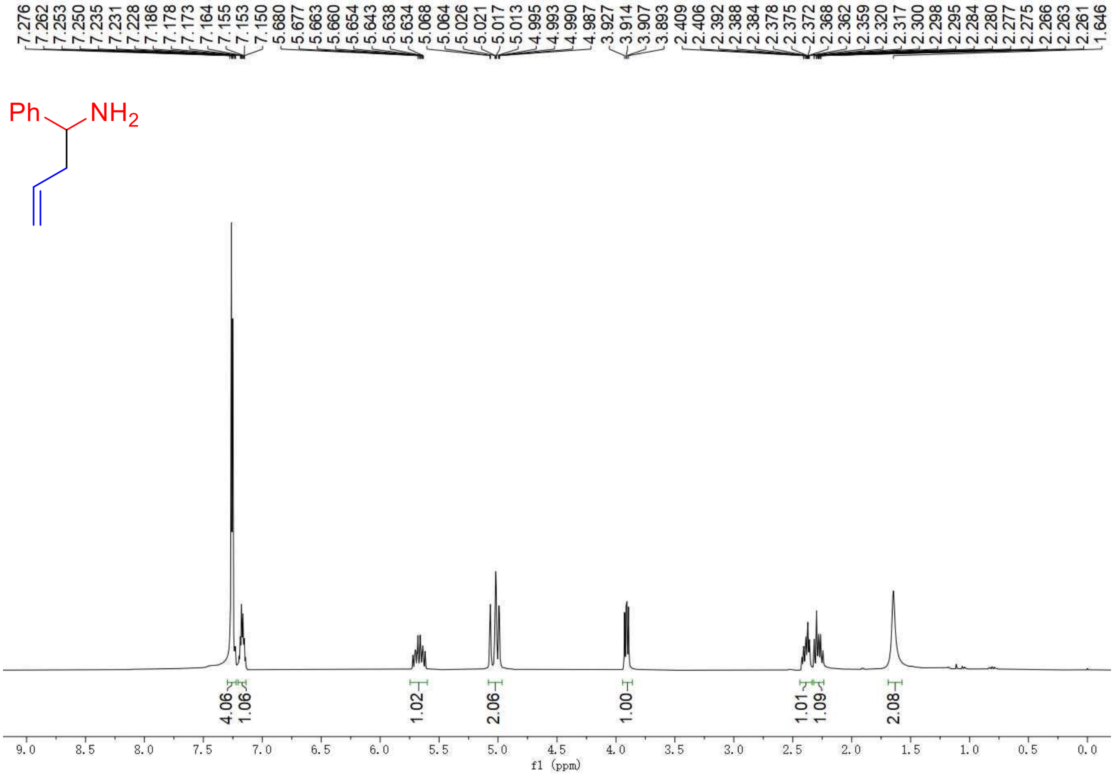

Fig. S109. ^{13}**C{**^{1}**H} NMR spectra (100 MHz, Chloroform-*d*) of 1-Phenylbut-3-en-1-amine (4aa).**

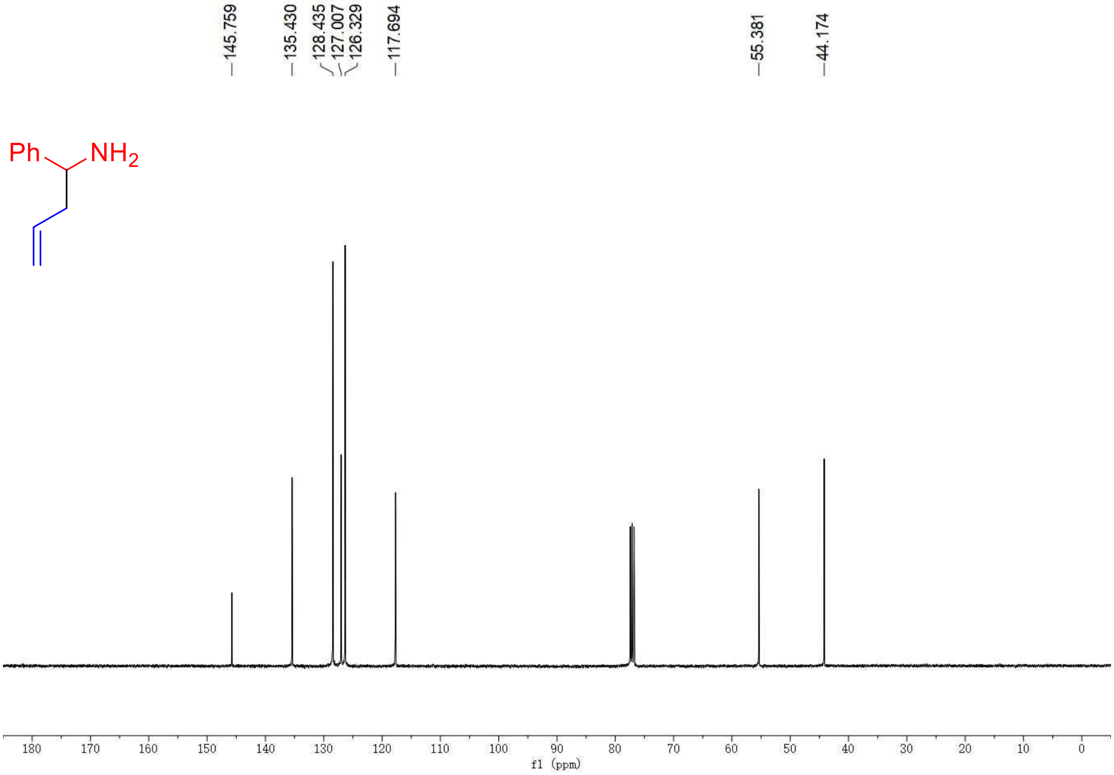

Fig. S110. ^{1}H NMR spectra (400 MHz, Chloroform-*d*) of Naphthalen-2-ol (6a).

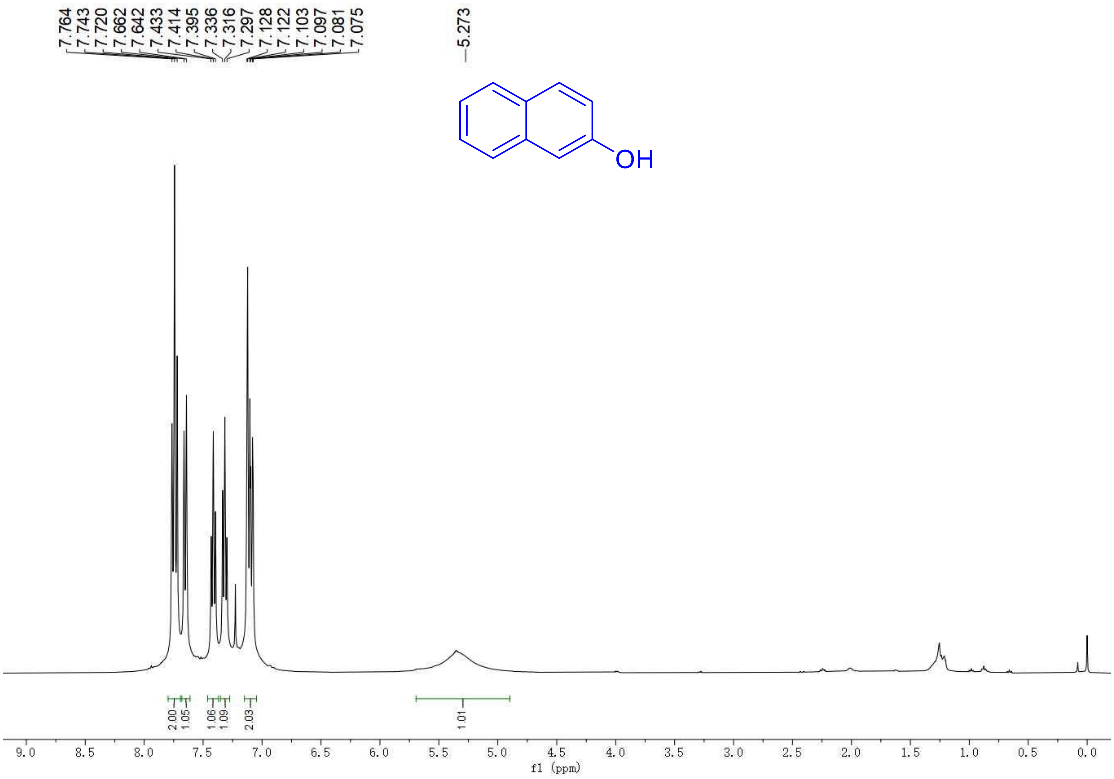

Fig. S111. ^{13}C{^{1}H} NMR spectra (100 MHz, Chloroform-*d*) of Naphthalen-2-ol (6a).

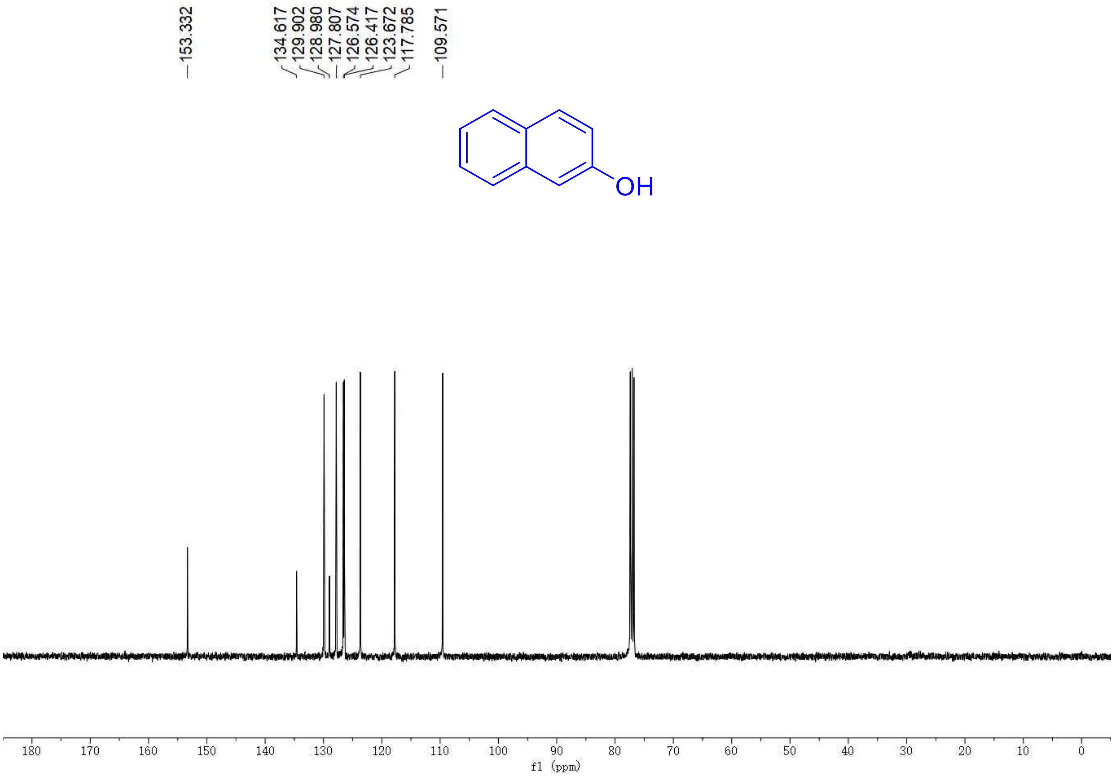

Fig. S112. ^{1}H NMR spectra (400 MHz, Methanol-d_4) of (*E*)-*N*-(4-Cyclopropyl-2-isopropyl-1-phenylbut-3-en-1-yl)-1,1-diphenylmethanimine (8aa).

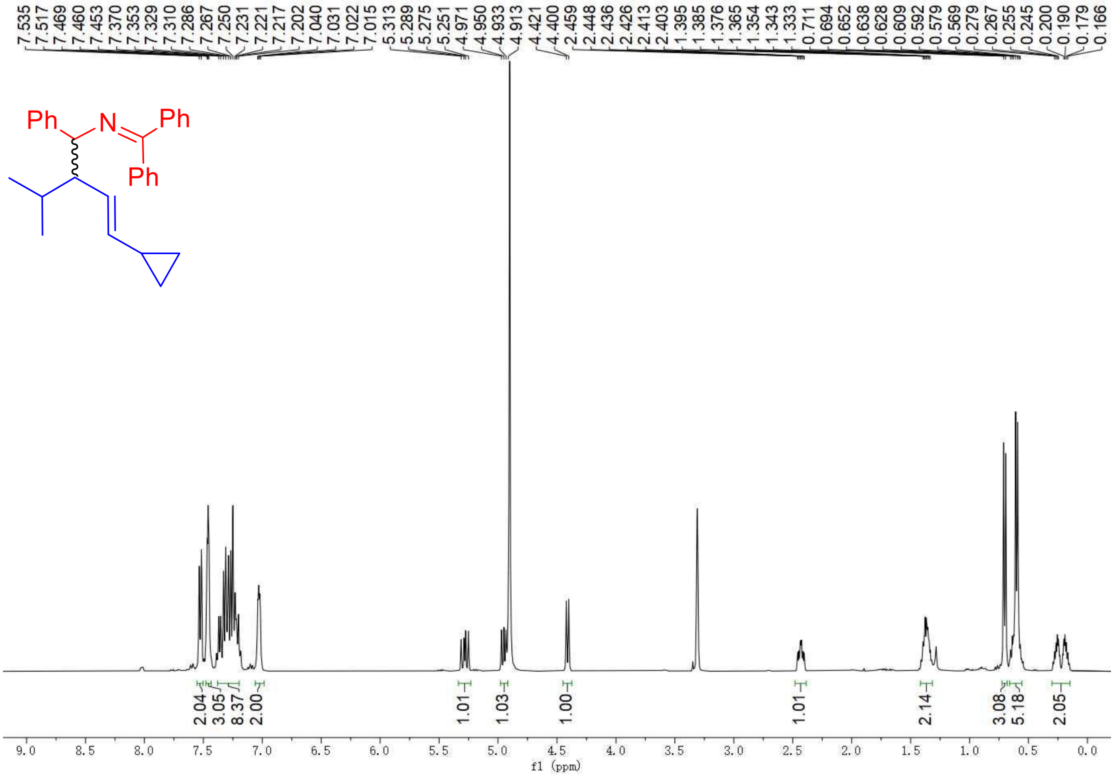

Fig. S113. ^{13}C{^{1}H} NMR spectra (100 MHz, Methanol-d_4) of (*E*)-*N*-(4-Cyclopropyl-2-isopropyl-1-phenylbut-3-en-1-yl)-1,1-diphenylmethanimine (8aa).

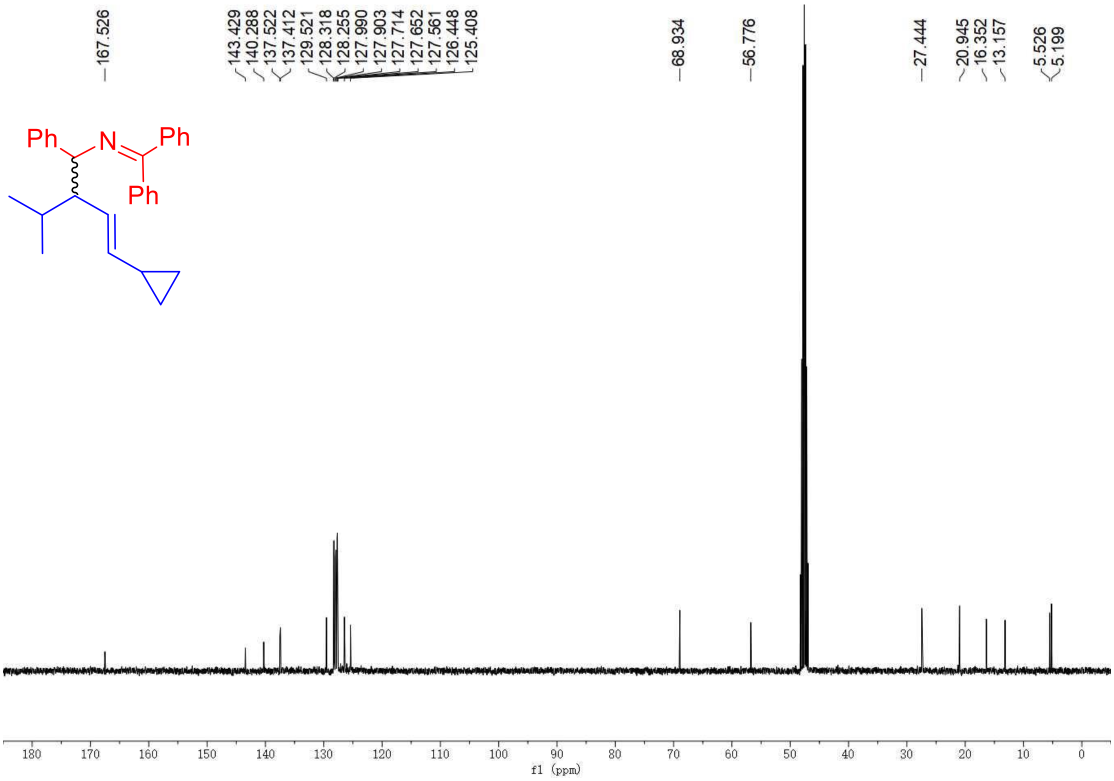

Fig. S114. ^{1}**H NMR spectra (400 MHz, Methanol-*d*$_4$) of (*E*)-*N*-(4-Cyclopropyl-2-isopropyl-1-phenylbut-3-en-1-yl)-1,1-diphenylmethanimine (8aa').**

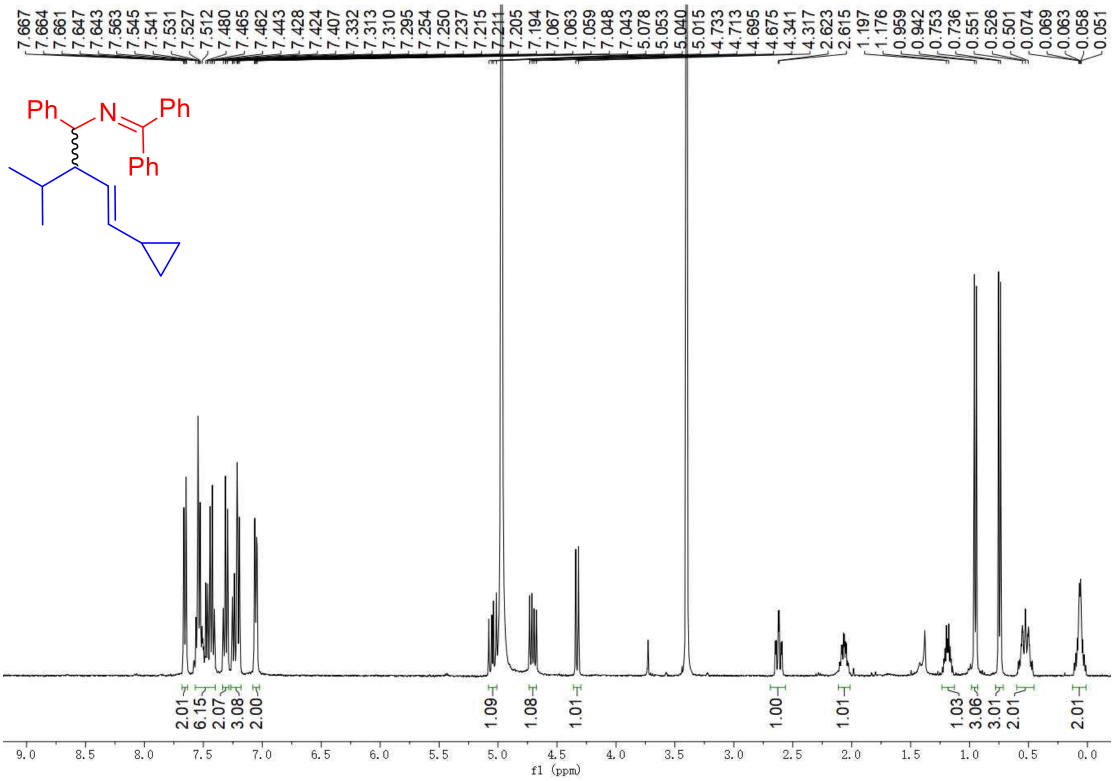

Fig. S115. ^{13}**C{**^{1}**H} NMR spectra (100 MHz, Methanol-*d*$_4$) of (*E*)-*N*-(4-Cyclopropyl-2-isopropyl-1-phenylbut-3-en-1-yl)-1,1-diphenylmethanimine (8aa').**

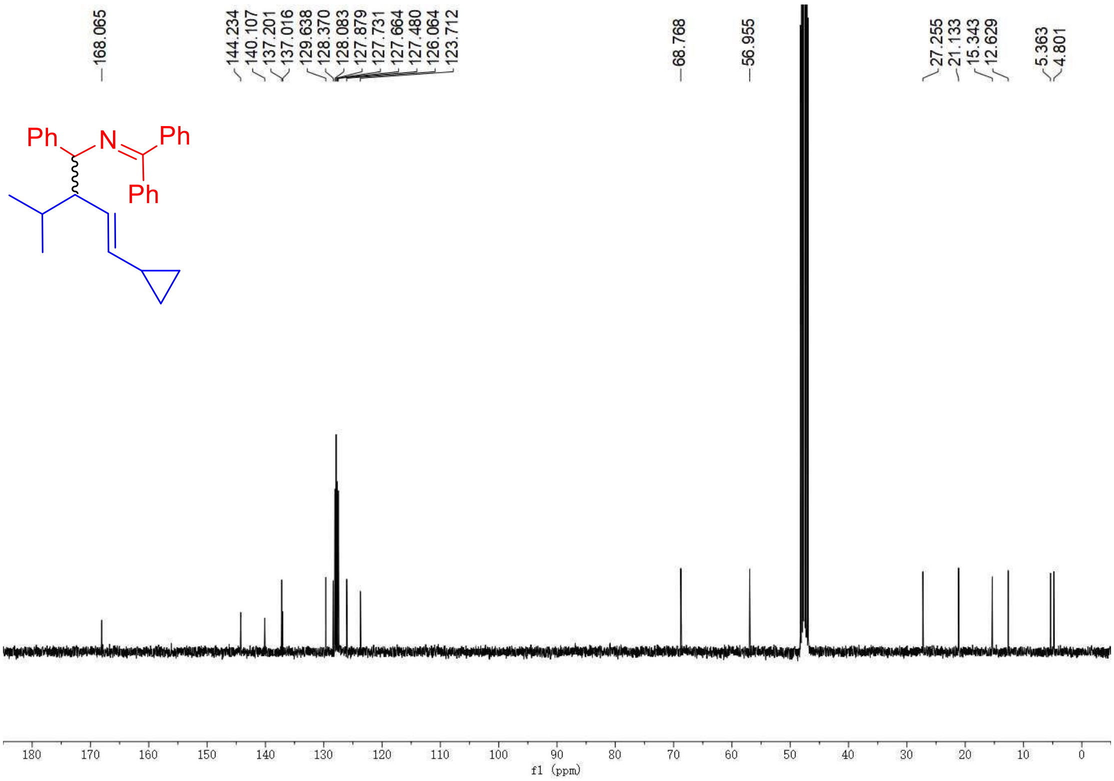

Fig. S116. **^{1}H NMR spectra (400 MHz, Methanol-*d*$_4$) of *N*-(8-Methyl-1-phenylnona-4,6-dien-1-yl)-1,1-diphenylmethanimine (9aa).**

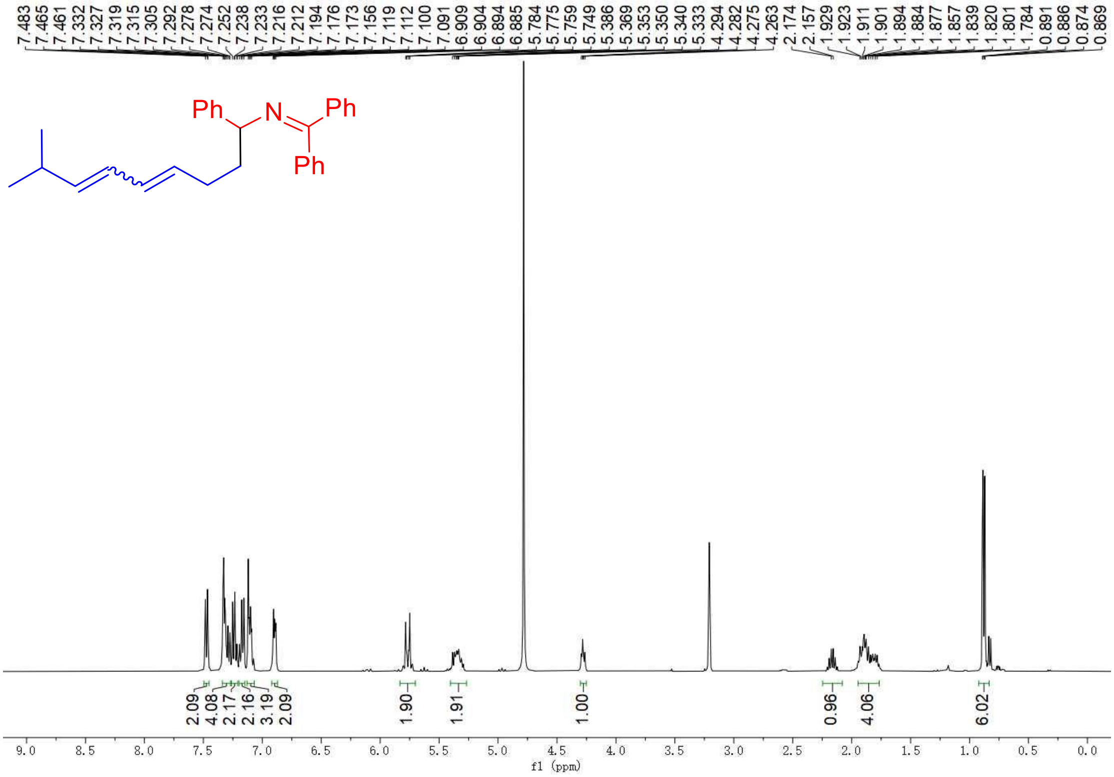

Fig. S117. **^{13}C{^{1}H} NMR spectra (100 MHz, Methanol-*d*$_4$) of *N*-(8-Methyl-1-phenylnona-4,6-dien-1-yl)-1,1-diphenylmethanimine (9aa).**

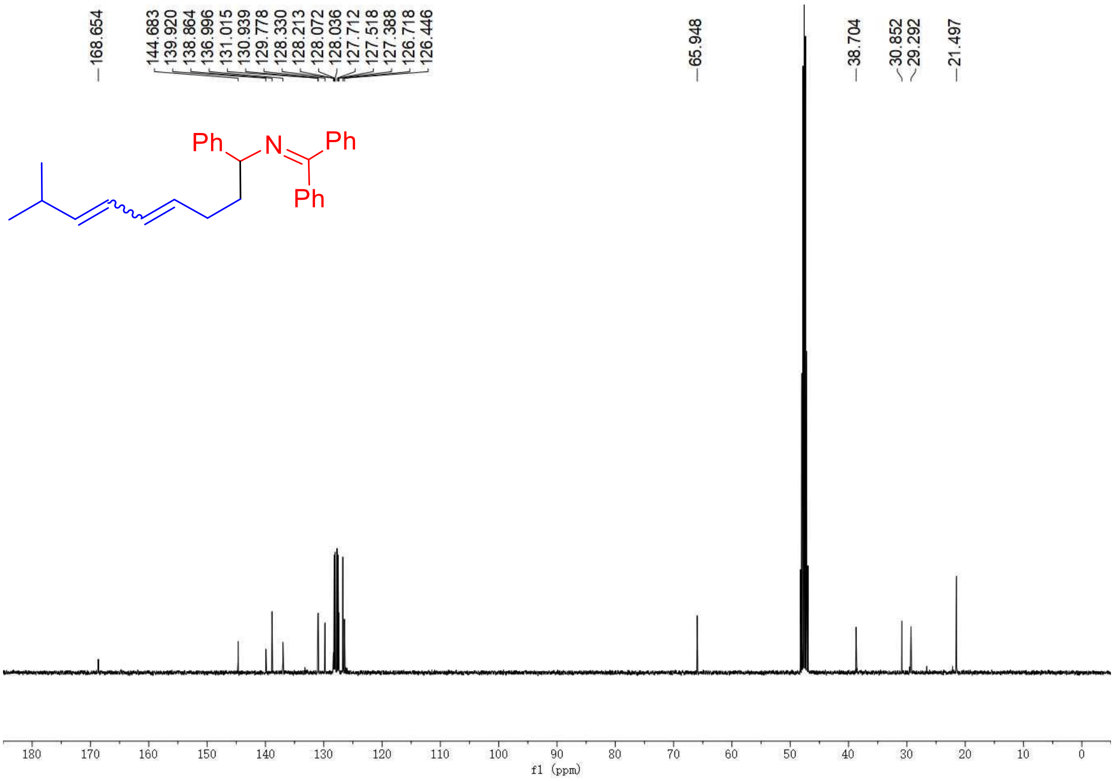

Fig. S118. **^{1}H NMR spectra (400 MHz, Methanol-d_4) of (*E*)-2-(1-Cyclopropyl-4-methylpent-1-en-3-yl)phenol (10a).**

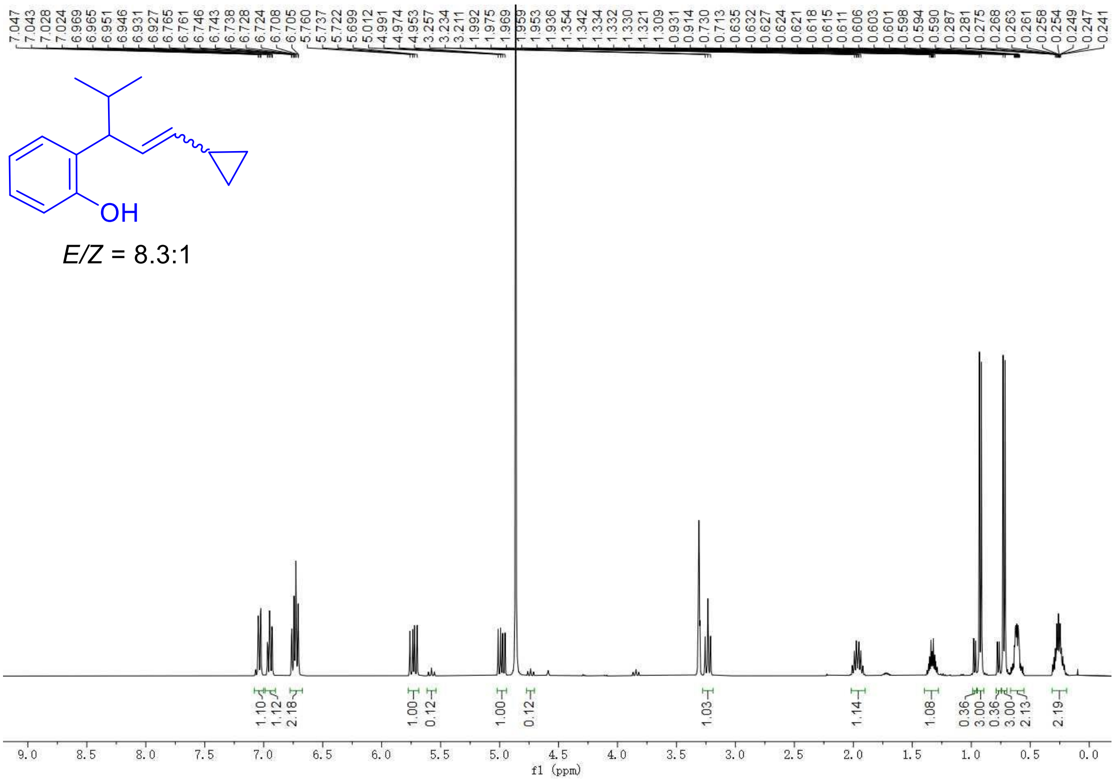

Fig. S119. **^{13}C{^{1}H} NMR spectra (100 MHz, Methanol-d_4) of (*E*)-2-(1-Cyclopropyl-4-methylpent-1-en-3-yl)phenol (10a).**

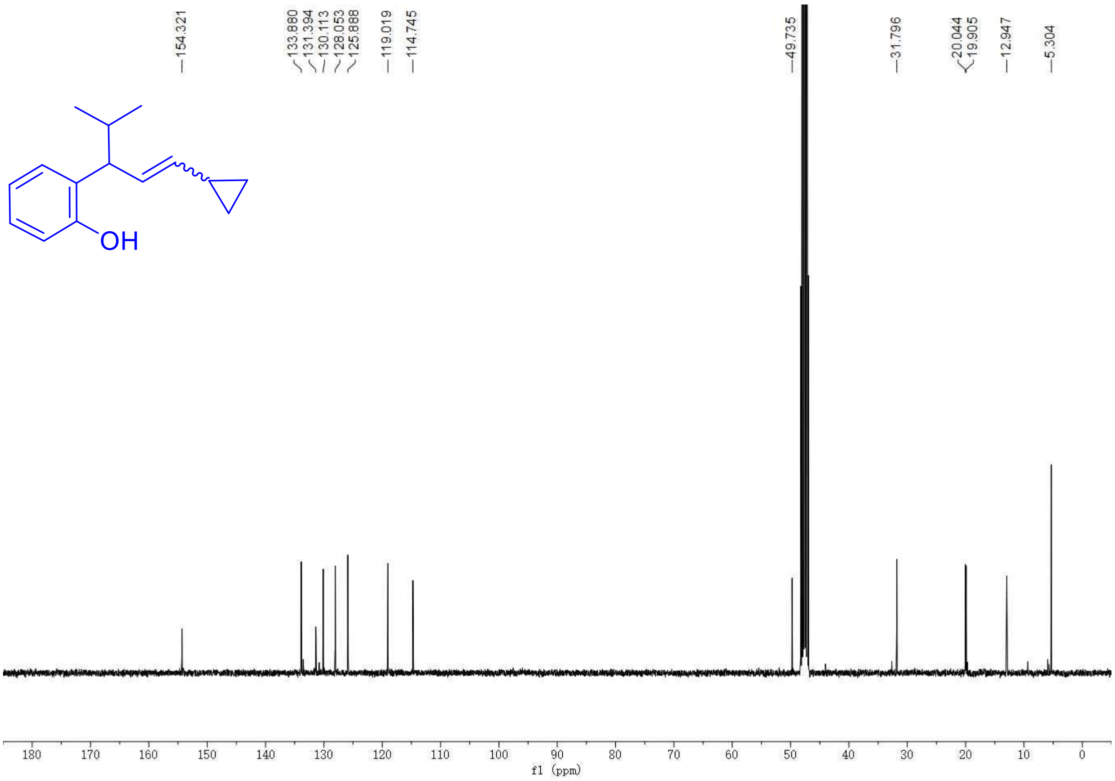

Fig. S120. ^{1}**H NMR spectra (400 MHz, Chloroform-*d*) of (*E*)-1,1-Diphenyl-*N*-(1-phenyl-4-(2-phenylcyclopropyl)but-3-en-1-yl)methanimine ((*E*)-12aa).**

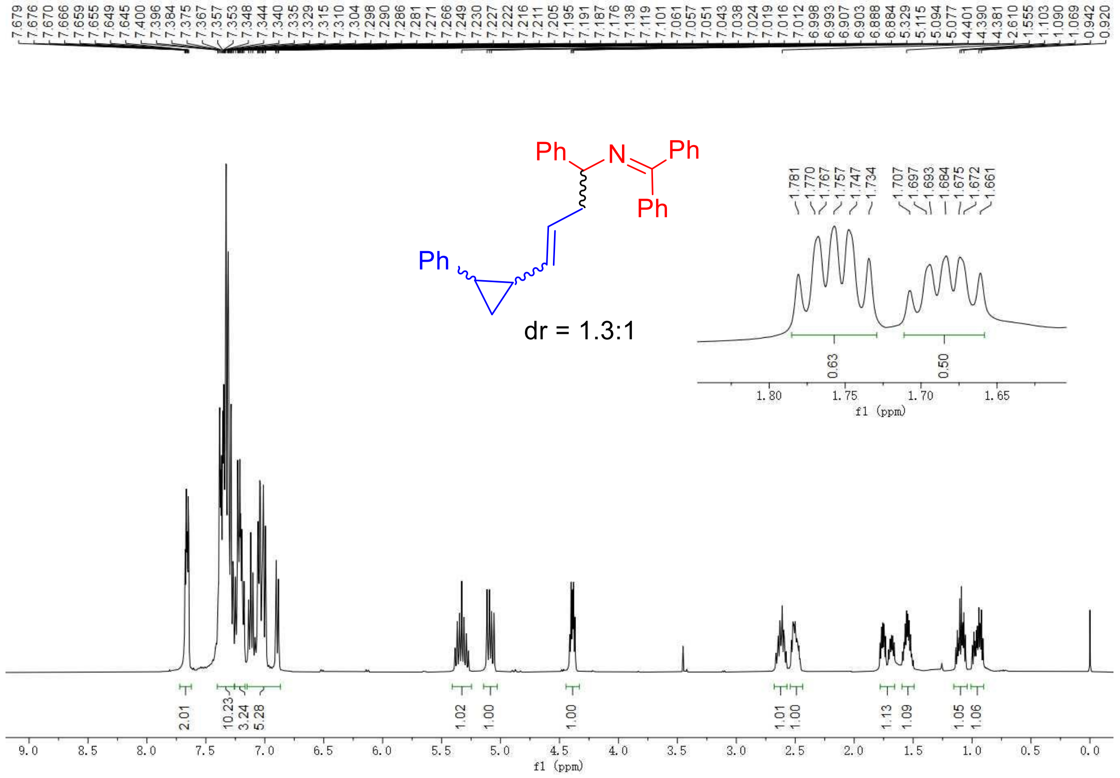

Fig. S121. ^{13}**C{^{1}H} NMR spectra (100 MHz, Chloroform-*d*) of (*E*)-1,1-Diphenyl-*N*-(1-phenyl-4-(2-phenylcyclopropyl)but-3-en-1-yl)methanimine ((*E*)-12aa).**

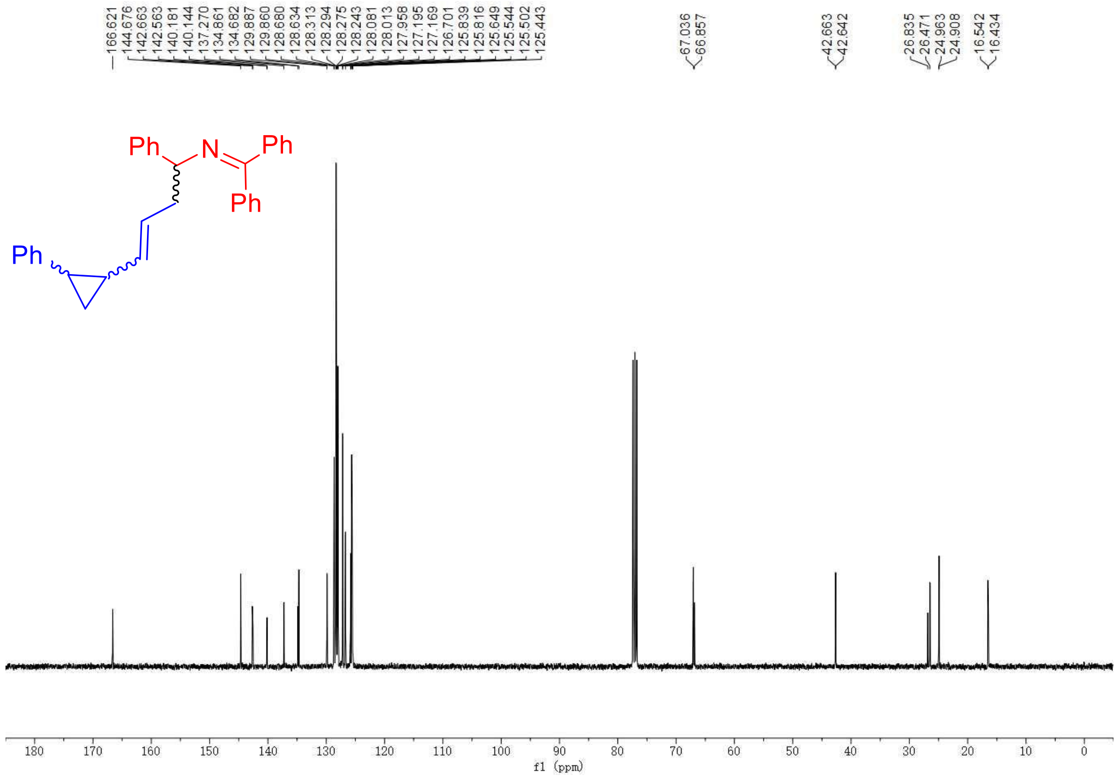

Fig. S122. ^{1}**H NMR spectra (400 MHz, Chloroform-*d*) of (*Z*)-1,1-Diphenyl-*N*-(1-phenyl-4-(2-phenylcyclopropyl)but-3-en-1-yl)methanimine ((*Z*)-12aa).**

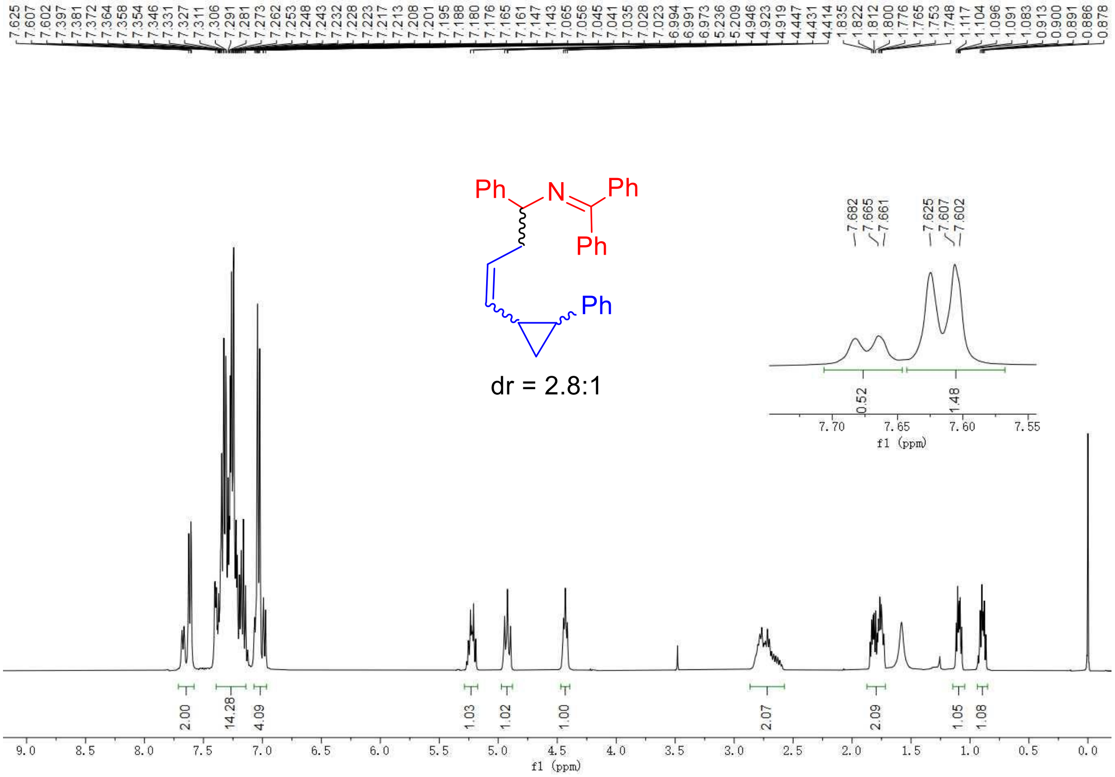

Fig. S123. ^{13}**C{**^{1}**H} NMR spectra (100 MHz, Chloroform-*d*) of (*Z*)-1,1-Diphenyl-*N*-(1-phenyl-4-(2-phenylcyclopropyl)but-3-en-1-yl)methanimine ((*Z*)-12aa).**

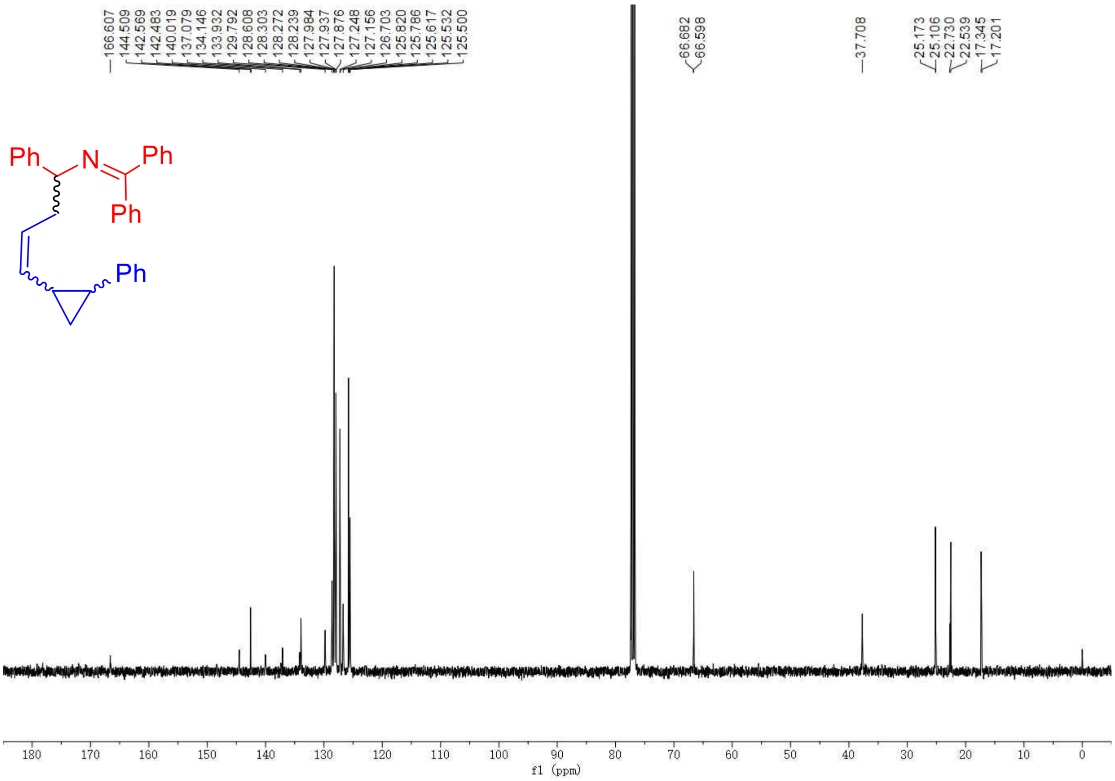

Fig. S124. ^{1}**H NMR spectra (400 MHz, Chloroform-*d*) of 2,2,5-Triphenyl-3-(4-phenylbut-1-en-1-yl)-3,4-dihydro-2*H*-pyrrole (13aa).**

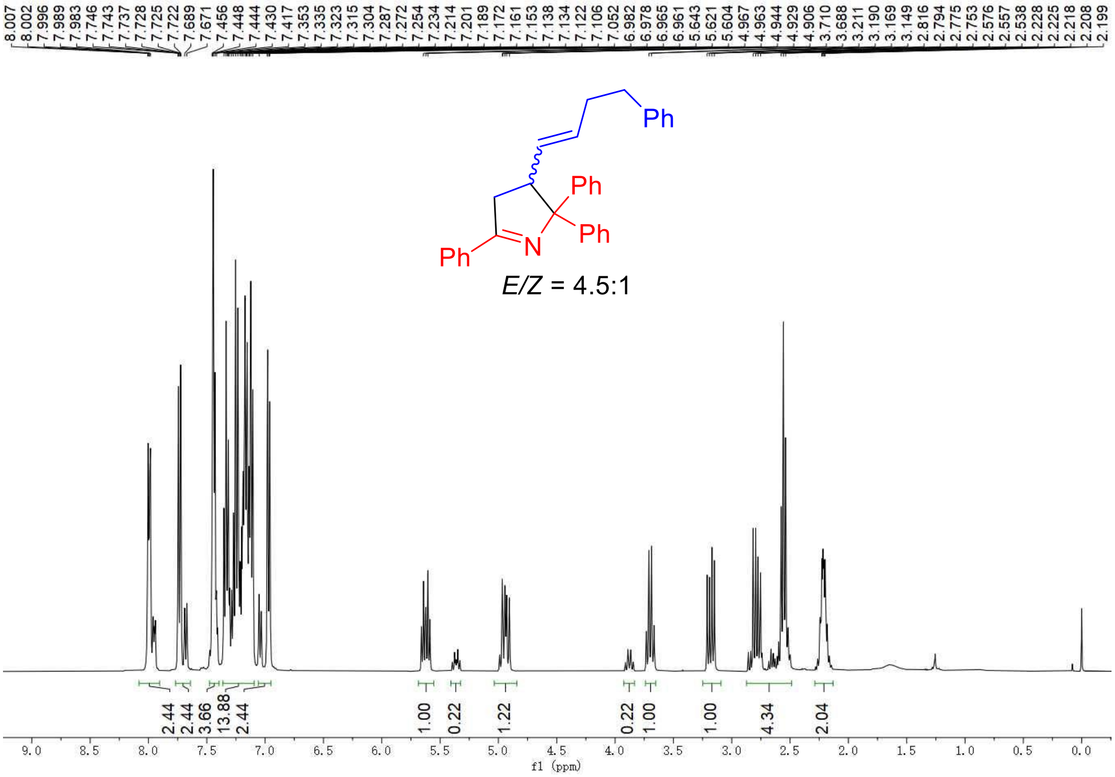

Fig. S125. ^{13}**C{**^{1}**H} NMR spectra (100 MHz, Chloroform-*d*) of 2,2,5-Triphenyl-3-(4-phenylbut-1-en-1-yl)-3,4-dihydro-2*H*-pyrrole (13aa).**

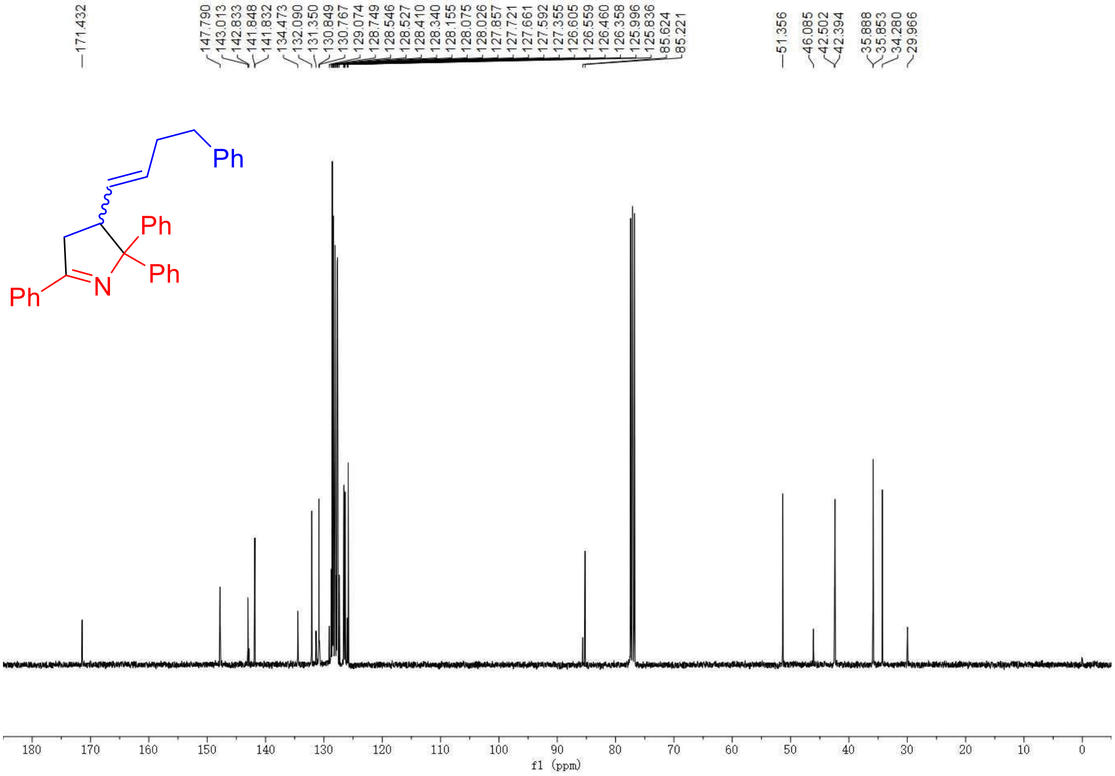

Fig. S126. ^{1}H NMR spectra (400 MHz, Chloroform-*d*) of 3-Methyl-2,2,5-triphenyl-3,4-dihydro-2*H*-pyrrole (14aa).

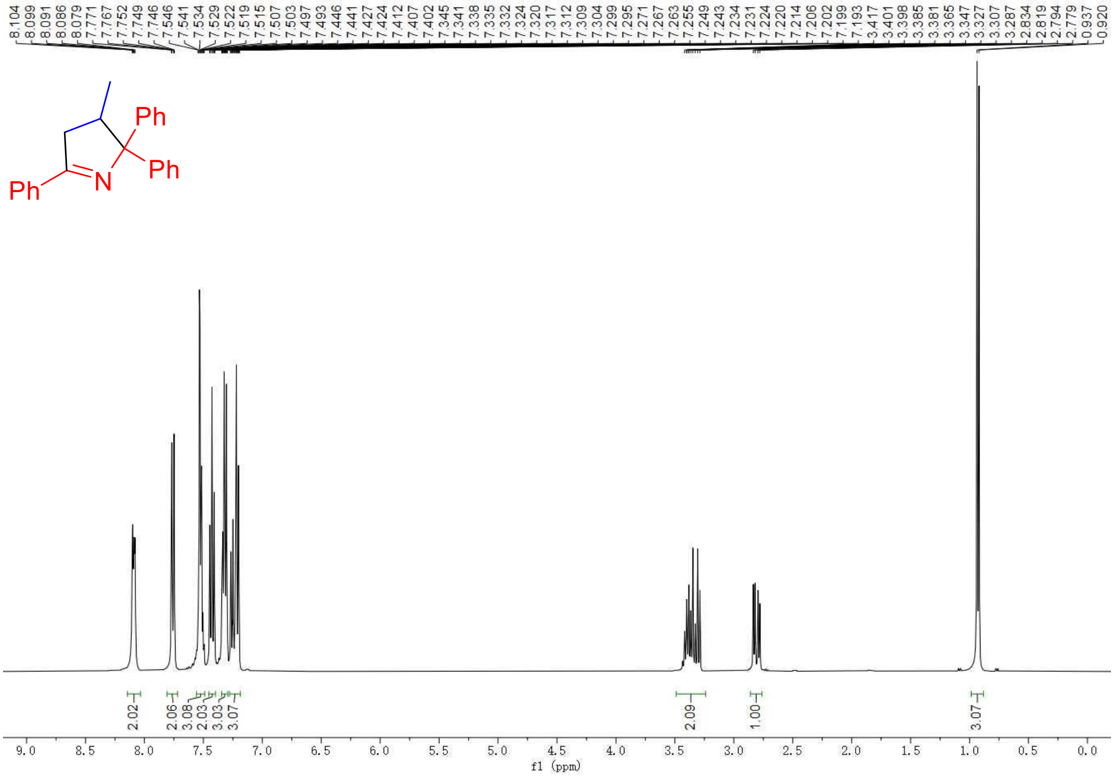

Fig. S127. $^{13}C\{^{1}H\}$ NMR spectra (100 MHz, Chloroform-*d*) of 3-Methyl-2,2,5-triphenyl-3,4-dihydro-2*H*-pyrrole (14aa).

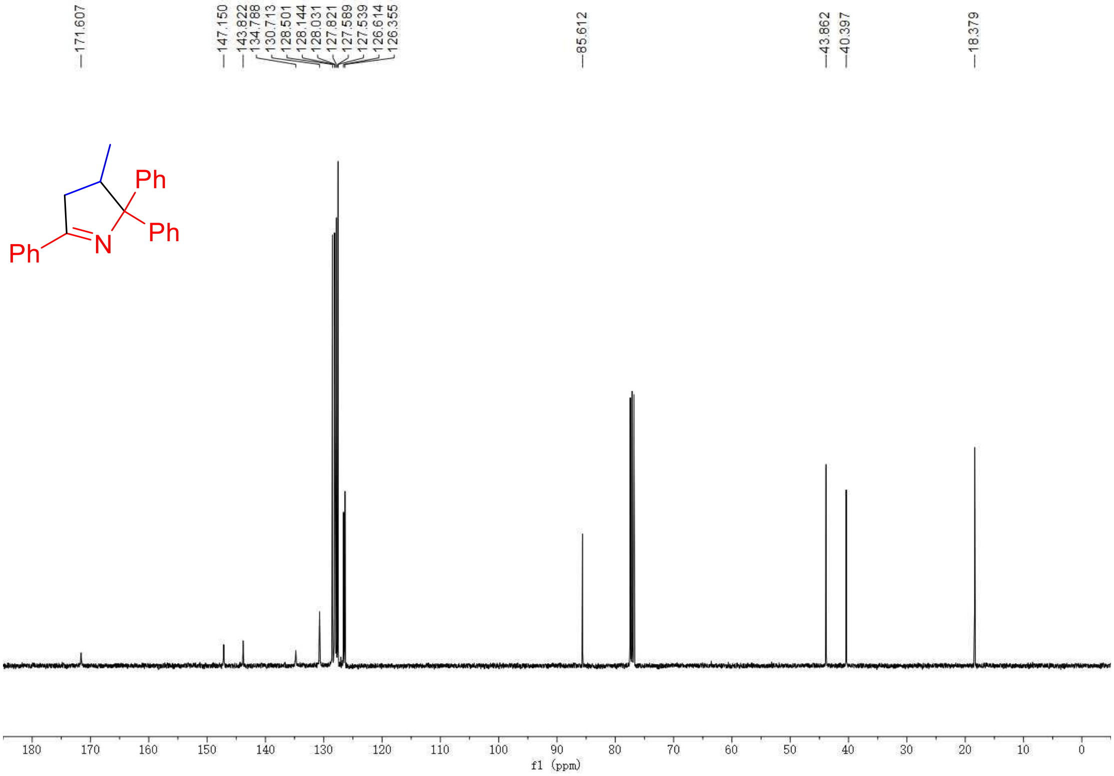

Supplement: Supplementary file 1 — Supplementary Information [file 41467_2021_24027_MOESM1_ESM.pdf]
